# Transcriptional profile of sweet orange in response to chitosan and salicylic acid

**DOI:** 10.1186/s12864-015-1440-5

**Published:** 2015-04-12

**Authors:** Danila Souza Oliveira Coqueiro, Alessandra Alves de Souza, Marco Aurélio Takita, Carolina Munari Rodrigues, Luciano Takeshi Kishi, Marcos Antonio Machado

**Affiliations:** Laboratório de Biotecnologia, Centro de Citricultura Sylvio Moreira, IAC, Cordeirópolis, São Paulo Brasil; Universidade Federal da Bahia, UFBA, Vitória da Conquista, Bahia Brasil; Universidade Federal de São Carlos, São Carlos, São Paulo Brasil

**Keywords:** Citrus, Gene expression, Metabolic pathway, RNA-seq

## Abstract

**Background:**

Resistance inducers have been used in annual crops as an alternative for disease control. Wood perennial fruit trees, such as those of the citrus species, are candidates for treatment with resistance inducers, such as salicylic acid (SA) and chitosan (CHI). However, the involved mechanisms in resistance induced by elicitors in citrus are currently few known.

**Results:**

In the present manuscript, we report information regarding the transcriptional changes observed in sweet orange in response to exogenous applications of SA and CHI using RNA-seq technology. More genes were induced by SA treatment than by CHI treatment. In total, 1,425 differentially expressed genes (DEGs) were identified following treatment with SA, including the important genes *WRKY5*0, *PR2,* and *PR9*, which are known to participate in the salicylic acid signaling pathway, and genes involved in ethylene/Jasmonic acid biosynthesis (*ACS1*2, *AP2* domain-containing transcription factor, and *OPR3*). In addition, SA treatment promoted the induction of a subset of genes involved in several metabolic processes, such as redox states and secondary metabolism, which are associated with biotic stress. For CHI treatment, there were 640 DEGs, many of them involved in secondary metabolism. For both SA and CHI treatments, the auxin pathway genes were repressed, but SA treatment promoted induction in the ethylene and jasmonate acid pathway genes, in addition to repressing the abscisic acid pathway genes. Chitosan treatment altered some hormone metabolism pathways. The DEGs were validated by quantitative Real-Time PCR (qRT-PCR), and the results were consistent with the RNA-seq data, with a high correlation between the two analyses.

**Conclusions:**

We expanded the available information regarding induced defense by elicitors in a species of *Citrus* that is susceptible to various diseases and identified the molecular mechanisms by which this defense might be mediated.

**Electronic supplementary material:**

The online version of this article (doi:10.1186/s12864-015-1440-5) contains supplementary material, which is available to authorized users.

## Background

Citrus is one of the most important crops around the world. Brazil is currently the largest producer of sweet oranges and is the largest producer and exporter of freezer concentrate and not-from-concentrate orange juice [1]. However, one of the limiting factors that threatens the growth and productivity of citrus production are citrus diseases. The integrated management of plant (IMP) diseases advocates alternate technologies, such as biological control and genetic resistance, to reduce the deleterious effects of pathogens. One strategy that may contribute to disease reduction is the use of elicitors to improve the natural resistance of the plant. Among the compounds that have been used to control pathogens are salicylic acid (SA) and chitosan (CHI).

SA is a phenolic compound produced by plants, and its biosynthesis and signaling pathways have been well characterized, demonstrating its important role as a signal involved in the plant defense against pathogens [2]. The biosynthesis of SA may culminate in the expression of resistance genes that promote systemic acquired resistance (SAR). During infection, SA accumulates at the site of pathogen penetration, acting in the hypersensitivity reaction, and is also distributed to other parts of the plants as a mobile signal, as methyl salicylate, to induce a range of defense responses [3]. Many plants are not able to deploy these mechanisms effectively. Studies on the exogenous application of SA in plants have revealed that it may induce systemic resistance and promote the accumulation of pathogenesis-related (PR) proteins [4]. The effectiveness of SA treatment has been verified against diseases caused by virus [5], fungi [6,7], and bacteria [8,9]. The exogenous application of SA has been shown to induce *PR1* mRNA and to reduce the systemic multiplication of the Alfalfa mosaic virus (A1MV) [5]. The potential for the exogenous application of SA to increase PR protein expression in bean plants and to reduce local lesions caused by A1MV was also demonstrated [5]. SA treatment promoted the resistance of asparagus against *Fusarium oxysporum* f. sp. *asparagi*, with increases in the levels of peroxidases, phenylalanine ammonia-lyase, and lignifications [6]. Similar results were obtained in tomato plants, where the application of SA to the roots reduced vascular browning caused by *F. oxysporum f. sp. lycopersici* and increased the levels of peroxidases, phenylalanine ammonia-lyase activities and the endogenous accumulation of free SA, showing that the root system might assimilate and distribute SA throughout the plant [7]. SA treatment was also effective in inducing several PR proteins in grapevine leaves [10]. In addition to demonstrating the effectiveness of SA applications against virus and fungi, studies have reported the effects of exogenous applications of SA for the control of bacterial diseases. There was an increase in the resistance of tobacco against *Erwinia carotovora* following SA treatment, which promoted reductions in disease symptoms and bacterial multiplication [11]. A recent study demonstrated the potential for SA treatment to attenuate the symptoms of citrus canker in sweet orange [*Citrus sinensis* (L.) Osbeck] by measuring the enzyme activities of phenylalanine ammonia-lyase and β-1,3-glucanase, as well as the mRNA levels of *CsCHI* and *CsPR4* [12].

Chitosan, a β-1,4-linked glucosamine, is a deacetylated derivative of chitin and has a double effect: it is antimicrobial and it activates several plant defense mechanisms during host-pathogen interactions, such as the hypersensitivity reaction, callose deposition, lignification, synthesis of abscisic acid, phytoalexins, and pathogenesis-related proteins [13-17]. In grapevine, CHI treatment was effective against powdery mildew, reducing disease severity and increasing the polyphenol content [18]. A recent study showed that CHI can act on the phenylpropanoid pathway, increasing the levels of phenolic compounds in tomato plants and contributing to the reduction of bacterial spots [19]. In fruit trees, much of the research conducted on CHI treatments has been focused on post-harvest treatment, due to the ability of this polysaccharide to form a semi-permeable biofilm that modifies the atmosphere and reduces losses due to perspiration and dehydration, thus increasing the shelf life of fruits. Furthermore, CHI treatment may lead to the induction of resistance in fruits [20,21].

Previously, researchers evaluated the induction of resistance in plants by analyzing individual mechanisms involved in the stress response. However, these strategies contribute little to the comprehension of the defense-related mechanisms promoted by elicitors of resistance. Large-scale studies of gene expression have been increasingly conducted to assess the effects of elicitors on plant metabolism. The transcriptional profile of sorghum following exogenous applications of SA showed the induction of several defense genes, such as numerous PR genes and members of the phenylpropanoid and jasmonic acid (JA) pathway, showing patterns of synergistic effects between SA and JA, as well as mutual antagonism for the regulation of some genes [22]. Studies conducted by RNA-seq to describe the transcriptome in *Taxus chinensis* in response to the exogenous application of methyl jasmonate (MeJA) revealed thein duction of JA biosynthesis/JA signaling pathway/defense responses [23]. For CHI-treated *Arabidopsis thaliana* that was challenged with *Botrytis cinerea*, the transcriptome profile showed that the polysaccharide was able to induce camalexin biosynthesis genes through of the CERK1-independent pathway [24]. To the best of our knowledge, there are no previous studies showing the effects of CHI treatment in sweet orange. Although studies examining the exogenous application of SA on citrus are available, these studies lack the information regarding the changes in the general profile that are caused by the elicitor*.* To provide a large-scale study of gene expression in citrus treated with SA and CHI, and considering the important role, these elicitors have played in inducing defense mechanisms in several species, in the present study we aimed to evaluate the changes in the transcript pattern in sweet orange plants induced by these elicitors. The Illumina platform has been widely used to generate transcriptional profiles though RNA-seq, providing greater accuracy in measuring the levels of transcript. Using this method, we observed important changes mediated by elicitors in the defense response of sweet orange.

## Results and discussion

In a preliminary experiment, leaves of sweet orange cv. Pera were sprayed with SA and CHI at different concentrations to test transcriptional induction of key genes of the SA and ethylene response pathway. Leaves were chosen because the most important Citrus diseases affect the aerial part of the plants and none of the treatments was shown to be phytotoxic. In addition, the results revealed that the best concentrations for CHI and SA were 4 mg/mL and 2.5 mM, respectively. The interval of 48 hours was the most appropriate for testing the response to CHI while for SA it was 24 hours (data not shown). These conditions were used for the RNA-seq experiment setup.

### Transcriptome profiling

To contribute to the understanding of how SA and CHI-treatments promote changes in transcript expression in sweet orange, transcriptional profiles of leaf samples treated with elicitors were generated using RNA-seq. Total RNA was extracted from elicitor-treated sweet orange plants and mock-treated plants (ethanol solution 10% or HCl 0.05 N, pH 5.6 for SA and chitosan, respectively), and then four cDNA libraries were created for sequencing with Illumina technology.

**Figure 1:**
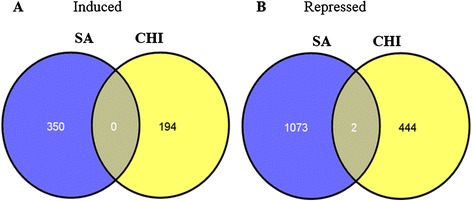
Venn diagrams showing the number of genes with significantly altered expression levels (P≤ 0.05 and ≤ 1 fold change). Non-overlapping numbers represent the number of genes unique to a particular treatment (CHI or SA). Overlapping numbers represent the number of mutual genes between treatments. Panel **A** represents the induced transcripts and Panel **B** the repressed ones.

Between 11 and 15 million 31-nt paired end were generated from leaves that received different treatments (see Additional file 1: Table S1 and Additional file 2: Table S2). The Table 1 provides information about sequencing quality. The reads were aligned with the *Citrus clementina* reference genome and comparisons were made between SA- and E-treated plants (mock) and between CHI- and H-treated plants (mock) (see Additional file 1: Table S1 and Additional file 2: Table S2). The numbers of transcripts with significantly altered expression levels (*P* ≤ 0.05) following treatment with elicitors, based on the Cuffdiff analysis, are shown in Table 1. Compared to controls, more down-regulated genes were identified than up-regulated genes in plants treated with elicitors. Among the genes significantly induced by the treatments, 350 were unique to SA-treated plants, and 194 were unique to CHI-treated plants. Among the genes that were significantly repressed, 1,073 were unique to SA-treated plants, and 444 were unique to CHI-treated plants (Figure 1). Only two genes were coregulated by SA and CHI treatments (encoding disease resistance family protein/leucine rich repeats (LRR) family protein, and hypothetical protein), and these genes were corepressed. Between the two treatments, SA treatment altered the mRNA levels of a substantially greater number of genes compared with CHI treatment (Figure 1).

**Table 1:** **RNA-seq raw data and number of differentially expressed transcripts**

**Treatment**	**Number of reads**	**CG (%)** ^**†**^	**Q (20%)** ^**‡**^	**Transcripts with changed expression** ^**§**^
CHI	11,691,216	43.39	92.68	640
H*	13,253,654	43.36	92.56	-
SA	14,833,464	43.35	93.92	1,425
E*	12,279,260	43.64	92.34	-

*H and E represent the controls for CHI and SA, respectively.

^**†**^CG represents the quantity of CG bases in the sequences.

^**‡**^Corresponds to sequences with Phred quality > 20, which was higher than 92% for all RNA-seq libraries.

^§^Statistical significance (*P* ≤ 0.05).

All transcripts (up- and down-regulated) were obtained by RNA-seq after treatment with elicitors when compared to controls, according to Cuffdiff analysis.

All of the differentially up- and down-regulated genes were functionally categorized based on Gene Ontology (GO), in biological process, cellular component, and molecular function (level 2) (Table 2), and visualizations of the functional groups that were significantly altered by the treatments were generated by PageMan [25] and MapMan 3.5.1R2 [26] software.

**Table 2:** **Functional categorization of up- and down-regulated genes after treatments with SA and CHI**

Categories*	**Name**	**Number of transcripts**			
		**UP**_**SA**_	**DOWN**_**SA**_	**UP**_**CHI**_	**DOWN**_**CHI**_
Biological process	Metabolic process	179	122	81	221
	Cellular process	161	137	79	229
	Response to stimulus	68	59	20	70
	Biological regulation	47	49	28	93
	Localization	42	30	16	47
	Multicellular organismal process	25	20	8	44
	Developmental process	23	22	8	44
	Multi-organism process	17	7	4	13
	Cellular component organization	15	16	8	25
	Signaling	9	17	10	18
	**Total annotations**	**586**	**479**	**262**	**804**
Cellular component	Cell	196	142	102	283
	Organelle	140	98	73	197
	Macromolecular complex	42	12	22	40
	Membrane-enclosed lumen	9	4	4	11
	Extracellular region	8	3	3	4
	**Total annotations**	**395**	**259**	**204**	**535**
Molecular function	Binding	157	140	77	281
	Catalytic activity	149	123	74	217
	Transporter activity	25	25	7	33
	Electron carrier activity	15	3	15	5
	Structural molecule activity	14	2	7	4
	Molecular transducer activity	11	9	7	17
	Enzyme regulator activity	8	6	3	2
	Antioxidant activity	5	0	2	3
	Transcription regulator activity	1	5	6	23
	**Total annotations**	**385**	**313**	**198**	**585**

*Individual gene products may be assigned to more than one functional category.

The differentially expressed genes were distributed into 10 biological processes, five cellular components and nine molecular functions. Within each category, the greatest number of GO annotated genes were associated with metabolic processes (28.3% for both SA and CHI treatments), cellular processes (27% for SA treatment and 28.9% for CHI treatment), cell (51.4% for SA treatment and 52.1% for CHI treatment), and binding (42.6% for SA treatment and 45.7% for CHI treatment) (Table 2).

For SA treatment, nearly 12% of the altered genes were considered to be genes that change expression in response to stimulus, and for CHI treatment that number was nearly 8% (Table 2). The transcripts most strongly up-regulated by SA treatment were those that encoded peroxidase superfamily proteins, which are involved in the response to oxidative stress during the defense response and the deposition of lignin, 2-oxoglutarate (2OG) and Fe(II)-dependent oxygenase superfamily proteins that are involved in the biosynthetic process of flavonoids, and β-1,3-glucanase, which has antimicrobial properties (see Additional file 3: Table S3, Additional file 4: Table S4-1, Additional file 5: Table S4-2). For CHI treatment, the most strongly up-regulated transcripts were those that encodes members of the tetratricopeptide repeat-like superfamily, the glycosyltransferase family, and the 2OG and Fe(II)-dependent oxygenase superfamily (see Additional file 6: Table S5, Additional file 7: Table S6-1, and Additional file 8: Table S6-2).

### Modulated metabolic process induced by exogenous SA and CHI

Many studies have shown that applications of exogenous SA and CHI increase the defense response in several plants [5-21]. SA is required for SAR and plays an important role in defense signaling. In a study performed in *Citrus sinensis* Osbeck it was demonstrated that SA treatment was able to enhance resistance against *Xanthomonas axonopodis* pv. *citri* elevating the activities of phenylalanine ammonia-lyase and glucanase, and the mRNA levels of *CsCHI* and *CsPR4A* [12]. However, studies of the large-scale transcriptional responses promoted by SA and CHI treatments have not yet been reported in citrus. Our results provided a broader vision of the mechanisms involved in response induced by SA treatment and were consistent with the mechanisms discussed in previous studies, demonstrating that exogenous SA application increased the expression levels of genes involved in the defense response. However, CHI treatment did not promote considerable changes in the expression levels of genes involved in plant defense metabolism.

**Figure 2:**
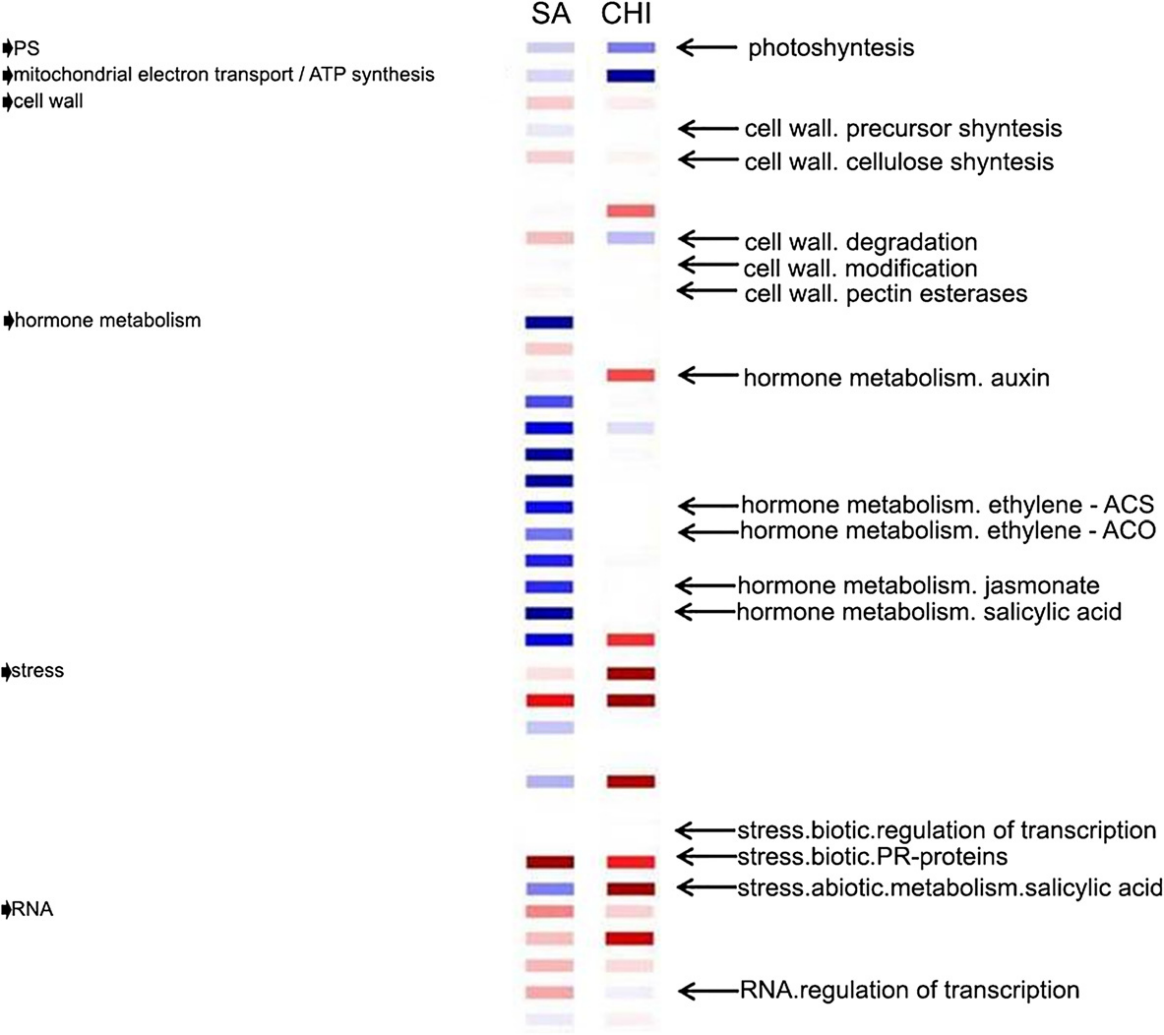
A comparative PageMan display of modulated pathways in sweet orange plants submitted to SA and CHI treatments. The fold changes of gene expression levels were input into PageMan and subjected to a Wilcoxon test. Pathways that were significantly up-regulated are colored in blue, and those colored in red were significantly down-regulated. Pathways without significant changes are in white. The names of pathways of interest are indicated on the right panel. PS, photosynthesis; CHO, carbohydrate.

Genes with putative roles in photosynthesis, cell wall synthesis/degradation/modification, hormone metabolism, the regulation of oxidative states, and transcriptional regulation showed distinctive patterns of regulation, as shown in Figure 2, which was obtained using PageMan software.

**Figure 3:**
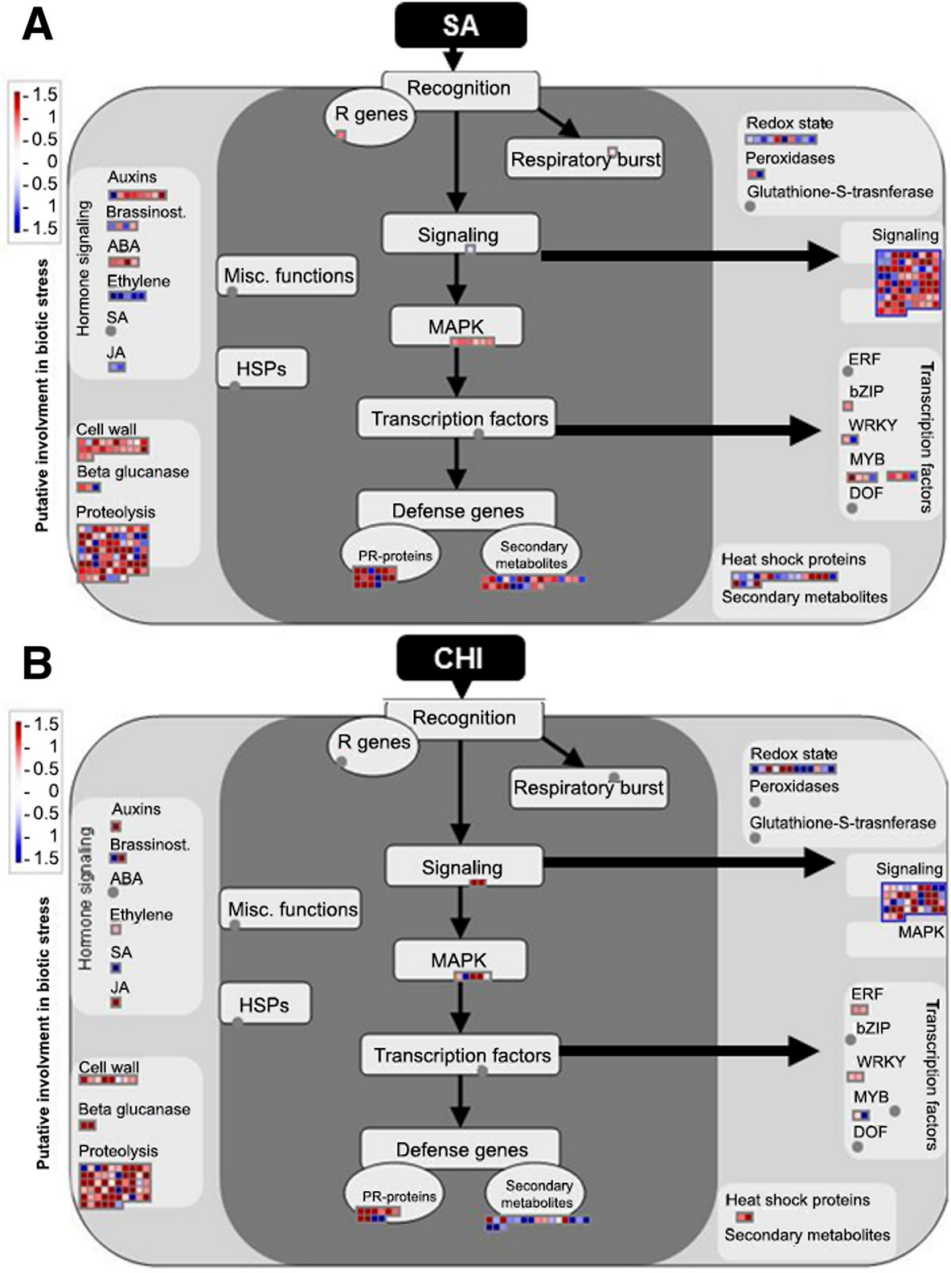
Differentially expressed transcripts related to stress responses. Treatment-modulated stress responses were evaluated in SA-treated plants (**A**) and CHI-treated plants (**B**). The fold change of gene expression levels were analyzed using MapMan. Small red and blue squares represent up- and down-regulated genes, respectively.

Several subsets of genes encoding receptor-like kinases, signaling proteins, transcriptional factors, oxidative stress response elements, secondary metabolism factors, and phytohormone-responsive genes were identified (Figure 3). Notably, a greater number of genes were modulated by SA treatment than by CHI treatment in all of the identified pathways. The functions of the genes described below are based on homology to genes of known functions from other organisms, primarily *A. thaliana*, with the help of MapMan software.

#### Genes associated with defense signaling/activation and redox state

A sequence of molecular events leads to the establishment of plant defense mechanisms, and the first step of this sequence is the recognition of the elicitor molecule by a specific receptor. Our results have shown that several receptor-like kinases were observed among the differentially expressed genes, with a greater number of genes that contain a LRR sequence being identified in SA-treated plans than in CHI-treated plants. Of these LRR containing genes, only one was considered up-regulated in SA-treated plants, where as two were considered up-regulated in CHI-treated plants. One member of the receptor-like kinase (RLK) family was induced only by SA treatment, while the receptor kinase associated to extensin, which participate of the primary cell wall of plants, was repressed in both treatment groups (see Additional file 3: Table S3 and Additional file 6: Table S5). The phosphoinositide-specific phospholipase C (PLC) pathway may respond to hormones or pathogen elicitors by releasing cytosolic Ca^2+^ [27]. The gene encoding the plasma membrane protein PLC4 was also induced by both SA and CHI treatments.

Our results suggest that in sweet orange exogenous SA might be activating RLK receptors. In *A. thaliana*, it was observed that the exogenous application of SA increased the expression levels of *RLK* genes, which exhibit a TTGAC sequence in their promoter regions that appear to be related to plant defense genes [28]. Thus, it is possible that exogenous SA might be recognized by RLK family members and might be able to increase the expression levels of various RLKs in citrus. The exact CHI recognition mechanisms in plants are not completely understood. Studies have suggested that CHI can be recognized by chitin receptors, such as CERK [29] however, in *A. thaliana*, it was observed that the perception of CHI occurred through a CERK1-independent pathway [24]. In our results, the CERK gene was not represented in the profile of CHI-treated plants, suggesting that the CHI is recognized by CERK independent mechanisms.

Another signaling factor that appeared to be involved in SA treatment but was not observed in CHI treatment was the repression of the *GLB1* gene (GLNB1-like protein), which is responsible for encoding a haemoglobin that oxidizes nitric oxide (NO) [30]. The suppression of *GLB1* suggests that SA treatment can favor the accumulation of NO in citrus, as has been shown in *A. thaliana* [31]. Just as studies have demonstrated that SA produces NO, it is also possible that NO stimulates SA accumulation [32,33]. Both signaling molecules, SA and NO, are well known to play important roles in the activation of plant defense after elicitation. However, the interrelationship between these two signaling molecules and their pathways is not understood. It is likely that SA and NO promote the hypersensitive response and pathogen death [34].

Because elicitors are recognized and accumulate in cells, they may trigger diverse processes, such as redox regulation. Oxidative stress occurs when there are changes in environmental conditions, causing the cell to produce reactive oxygen species (ROS), which result in the oxidation of cellular components, alter metabolic activities and affect organelle integrity. Thus, balancing these ROS through the activation of ROS responsive regulatory genes is required [35]. Several proteins play a critical role in redox in plants, including thioredoxins, superoxide dismutase, and glutaredoxin. Thioredoxins were induced by SA and CHI treatments. These proteins may participate in the conversion of *NRP1* (SA-induced nonexpresser of *PR* genes 1) to a monomer when the plant is elicited, by a pathogen or by SA treatment [36]. CSD2 (copper/zinc superoxide dismutase 2), which was induced by both treatments as determined by RNA-seq and by qRT-PCR (Tables 3 and 4), may promote the dismutation of superoxide radicals (O_2_^−^) in H_2_O_2_, which is part of the programmed cell death of plant cells and has been correlated with disease resistance [37]. The enhanced expression of superoxide dismutase by SA and CHI treatments has been reported in *A. thaliana*. Kliebenstein et al. [38] have shown that treatment with the SA analogs 2,6-dichloroisonicotinic acid and benzothiadiazole S-methylester (BTH) induced two CuZnSOD proteins, which is consistent with the differential expression of *CSD2* in SA-treated plants observed in our results. Similarly, the protective effect of CHI against brown rot in peaches was associated with the induction of superoxide dismutase activity [39]. Therefore, SA and CHI appear to act in plant protection by contributing to programmed cell death during the important event of disease resistance.

**Table 3:** **Defense-genes up- and down-regulated in sweet orange in response to SA-treatment (** ***P*** **< 0.01)**

**SA**			
**Gene ID**	**Gene name/function**	**RNA-seq** ^*^	**qRT-PCR** ^*^
AT1G19220	ARF19/ auxin response factor 19	−2.67	−1.39
AT3G12500	HCHIB/ basic chitinase	−2.13	NA^‡^
AT5G14420	RGLG2/ RING domain ligase 2	−1.89	−1.92
AT3G62980	TIR1/ F-box/RNI-like superfamily protein	−1.19	−1.17
AT1G75580	SAUR-like auxin-responsive protein family	−1.12	−1.85
AT5G17820	Peroxidase superfamily protein	3.19	1.09
AT2G28190	CSD2/copper/zinc superoxide dismutase 2	2.27	1.04
AT3G57260	BGL2/beta-1,3-glucanase 2	2.06	1.14
AT4G10490	2-oxoglutarate (2OG) and Fe(II)-dependent oxygenase superfamily protein	1.80	3.07
AT2G32440	ACS12/ 1-aminocyclopropane-1-carboxylate synthase	1.54	NA
AT5G26170	WRKY50/WRKY DNA-binding protein 50	1.31	1.42
AT5G23960	TPS21/terpene synthase 21	1.05	NA
AT2G06050	OPR3/12-oxophytodienoate reductase 3	1.03	NA
AT1G05010	1-aminocyclopropane-1-carboxylate oxidase	0.9	0.85

^*^r = 0.85.

^**‡**^not assessed.

**Table 4:** **Defense-genes up- and down-regulated in sweet orange in response to CHI-treatment (** ***P*** **< 0.01)**

**CHI**			
**Gene ID**	**Gene name/function**	**RNA-seq** ^**†**^	**qRT-PCR** ^**†**^
AT5G60450	ARF4/auxin response factor 4	−227.07^**^	NA^**‡**^
AT3G12500	HCHIB/basic chitinase	−226.22	NA
AT5G62000	ARF2/auxin response factor 2	−182.94	−1.81
AT3G61415	EBF1 - protein binding/ubiquitin-protein ligase	−134,34	−1,36
AT2G25490	SK21/SKP1-like 21-SCF ubiquitin ligase complex	−135.59	−2.56
AT5G17420	Cellulose synthase	−160.14	−1.67
AT2G02560	CAND1/cullin-associated and neddylation dissociated	−198.86	NA
AT5G49330	MYB111/myb domain protein 111	175.1	NA
AT3G50740	UDP-glucosyltransferase 72 E1	158.32	NA
AT3G11480	BSMT1/S-adenosyl-L-methionine-dependent methyltransferases superfamily protein	158.09	NA
AT4G13400	2-oxoglutarate (2OG) and Fe(II)-dependent oxygenase superfamily protein	153.90	1.31
AT4G34050	CCoAOMT1/S-adenosyl-L-methionine-dependent methyltransferases superfamily protein	111.50	NA
AT2G28190	CSD2/copper/zinc superoxide dismutase 2	104.85	1.30

^**†**^r = 0.96.

^**‡**^not assessed.

^**^ = Important genes modulated by chitosan based on the transcript profiling and considering the study of Povero et al. [24].

#### Genes associated with the cell wall and secondary metabolism

Under the conditions used in this study, most of the genes associated with cell wall related-pathways were repressed by the treatments (e.g., any cellulose synthase, expansin, extensin, and UDP-arabinose 4-epimerase – see Additional file 4, Additional file 5 and Additional file 6), with more genes being represented in the profile for SA-treated plants. Expansin is involved in cell wall modification. This enzyme is required for wall relaxation during plant cell enlargement [40]. Proteins that loosen the cell wall play key roles in plant growth, however, this process may also make the plant vulnerable to attack by pathogens [41]. Thus, these data support the idea that expansin is negatively regulated by SA treatment. The auxin hormone, which has been implicated in disease susceptibility, induces the expression of expansins [41]. The suppression of auxin signaling by microRNA in *A. thaliana* resulted in the restriction of *Pseudomonas syringae* growth, indicating that the repression of auxin signaling is part of the plant-induced immune response [42]. Additionally, *A. thaliana* treated with the SA analog BTH showed the global repression of auxin-related genes, including the TIR1 receptor, indicating that the inhibition of auxin responses is a part of the SA-mediated disease-resistance mechanism [43]. We have shown that the sweet orange response to SA treatment appears to be functionally similar to that observed in *A. thaliana*. The factor that corroborates our results with those reported in previous studies is that expansins are induced by auxin, and this pathway was repressed in the present study. Other components of the auxin pathway were repressed and are described below. Among the few genes involved in cell wall metabolism with altered expression in the CHI treatment, genes involved in cellulose synthesis (e.g. cellulose synthase) were repressed, which was confirmed by qRT-PCR (Table 4).

It is well known that the treatment of plants with elicitors promotes the accumulation of defensive secondary metabolites. RNA-seq analysis showed that the expression levels of genes encoding enzymes associated with lignin synthesis, flavonoids, chalcones, and isoprenoid were altered in response to SA and/or CHI treatment (see Additional file 3: Table S3 and Additional file 6: Table S5). We observed that more than 60% of the differentially expressed genes in this group were repressed by treatments, including anthocyanins, chalcones, and 4-coumaroyl-CoA synthase. However, the 2OG-Fe(II) oxygenase family protein was induced by both SA and CHI treatments, which was confirmed by qRT-PCR (Tables 3 and 4). In addition, the genes *DMR6* (downy mildew resistant 6) and terpene synthase were observed to be induced by SA treatment. For CHI treatment, the induction of caffeoyl-CoA 3-O-methyltransferase, coniferyl-alcohol glucosyltransferase, and flavonoid 3'-monooxygenase was observed. The 2OG-Fe (II) oxygenase protein uses a dioxygen molecule to catalyze the 2OG and Fe (II)-dependent oxidation of an organic substrate. In plants, the 2OG-Fe (II) oxygenase protein participates in the synthesis of diverse compounds, such as flavones. ACC oxidase (1-aminocyclopropane-1-carboxylate oxidase), which is involved in the biosynthesis of ethylene (ET) and was represented in the profile of SA-treated plants, belongs to this family [44]. DMR6 is also a member of the 2OG-Fe(II) oxygenase superfamily of oxidoreductases and has been considered an enzyme that is activated during various defense responses, which can be activated by pathogen stimulation or can be chemically induced. Van Damme et al. [45] analyzed several dmr6 mutants in *A. thaliana* and observed that *DMR6* expression was sensitive to SA analogous. Interestingly, *DMR6* was observed to be induced to higher levels during incompatible interactions than during compatible interactions. During incompatible interactions, the induction of genes dependent on SA is observed. Therefore, the authors suggest that *DMR6* expression can be considered to be SAR-induced. Our results also indicated the participation of this gene in response to SA treatment in sweet orange.

A gene that is potentially involved with terpenoids was also observed to be regulated by treatments and is involved in secondary metabolism. The terpene synthases, which were induced by SA treatment but not by CHI treatment, are involved in the synthesis of various terpene molecules, which may serve as plant defenses against herbivores and pathogens [46-48]. However, we observed that CHI treatment induced important genes involved in phenylpropanoids metabolism, such as the caffeoyl CoA O-methyltransferases (see Additional file 6: Table S5, Additional file 7: Table S6-1, and Additional file 8: Table S6-2), which have been implicated in lignin biosynthesis and were not represented in the profile of SA-treated plants. The participation of CHI in phenylpropanoid metabolism has also been observed in tomato plants [19].

#### Transcriptional factors and hormone metabolism

**Figure 4:**
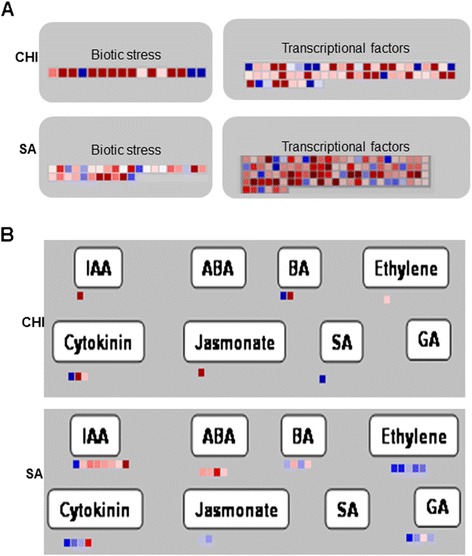
MapMan screenshots showing the expression of treatments-modulated genes. (**A**) Genes associated with biotic stress, abiotic stress and transcriptional factors, (**B**) differential expression of hormone-related transcripts in sweet orange submitted to SA and CHI treatments. The fold changes are indicated as gradients between red (down-regulated) and blue (up-regulated). Each point represents a transcript. IAA, indole-3-acetic acid; ABA, abscisic acid; BA,benzyladenine; SA, salicylic acid; GA, gibberellic acid.

Transcriptional factors were more strongly represented in the profile of SA-treated plants than in CHI-treated plants. A total of 106 regulated transcription factors were identified in SA-treated plants, and 59 were identified in CHI-treated plants (Figure 4A), including members of the *WRKY*, *ARF*, *MYB*, *bHLH*, *bZIP,* and *AP2/ERF* families (Additional file 6: Table S5). Most of these transcripts were down-regulated in both treatments (Figure 4A). Both treatments promoted the repression of *ARF* members involved in the response to auxin, which was confirmed by qRT-PCR (Tables 3 and 4). *TIR1* (transport inhibitor response 1) was also repressed by SA treatment, which was also confirmed by qRT-PCR (Table 3). *WRKY* factors, which are known to be involved in biotic stress and to participate in defense responses, showed increased expression levels in SA-treated plants, with *WRKY50* being up-regulated, which was confirmed by qRT-PCR; however, *WRKY2* and *WRKY4* were repressed in CHI-treated plants. Unsurprisingly, SA treatment has the potential to induce the transcription factors in the *WRKY* family [49] (Figure 3A). The WRKY proteins form a large family of transcription factors that bind w-box elements and have the potential to differentially regulate the expression of a variety of target genes [50]. The *WRKY* family members appear to play regulatory roles in responses against biotic and abiotic stress [51]. After an increase in the SA concentration in the cell cytosol, the *WRKY* family activates defense responses through its downstream components, which promote the expression of defense genes, such as the *PR* genes [49]. The induction of *WRKY* genes by SA treatment or BTH treatment has been demonstrated in other plants [52-55].

For hormone metabolism, the most differentially represented transcripts in the profile of SA-treated plants were involved in the auxin pathway, and 87.5% of these were down-regulated (Figure 4B), including *TIR1*, *ARF19,* and *BIG* transcriptional factors (Table 2). For CHI-treated plants, only nine genes were related to hormone metabolism, with most being down-regulated. We observed that the exogenous application of SA promoted an increase in the typical endogenous SA response because this hormone is able to attenuate auxin signaling, and reciprocally, the activation of the auxin pathway suppresses SA biosynthesis [56,57]. Genes involved in abscisic acid (ABA) biosynthesis and signaling were also repressed by SA treatment, such as *ELD1*, *AREB3,* and *HVA22K* (see Additional file 3: Table S3), which were not represented in the profile of CHI-treated plants. The activation of this pathway promotes disease susceptibility to several pathogens, but this pathway is important for abiotic stress resistance [58,59]. The MapMan analyses showed no genes in the SA pathway, but closer analyses showed that several components of the SA pathway were identified, such as *WRKY* family members and SA-dependent *PR* genes (Table 3 and Additional file 3: Table S3). A putative S-adenosylmethionine-dependent methyltransferase, which promotes the methylation of SA for the conversion into MeSA and is implicated in several aspects of plant defense signaling, was induced by CHI treatment (Table 4 and Additional file 3: Table S3).

Interestingly, genes involved in ET metabolism were up-regulated by SA treatment (Figure 4B), such as enzymes involved in synthesis and signal transduction (Table 3). In SA-treated plants, 15.6% of the up-regulated transcripts that were related to hormone metabolism were involved in ET metabolism, such as the ACS enzyme and AP2 domain-containing transcription factor (*ERF105*). Additionally, OPR3 (12-oxophytodienoate reductase 3) and proteinase inhibitors were up-regulated in SA-treated plants. The OPR3 enzyme is responsible for the conversion of JA from 12-oxophytodienoic acid, and proteinase inhibitors are produced by JA pathway signaling [60].

The hormone interactions that occur during the defense response in plants are complex, but it has been well demonstrated that SA and the auxin pathway interact antagonistically [61,62]. Similarly, ABA is able to antagonize the JA-ET signaling networks [62], and there have been data to suggest both positive and negative regulatory interactions between the ET and SA signaling pathways [63]. Our results showed that SA-treatment promotes an antagonism between SA and auxin and between SA and ABA because the repression of characteristic genes of the auxin and ABA pathways was observed. However, genes that participate in the SA pathway and the ET and JA biosynthesis pathways were induced, which is contrary to the idea that SA should suppress ET/JA synthesis. Several studies have suggested a mutual antagonism between SA and JA [64-68]. In contrast to this paradigm, some evidence has demonstrated strong positive interactions between the JA and SA pathways [68,69]. Sorghum treated with SA and other elicitors demonstrated the induction of genes belonging to the octadecanoic acid pathway of JA synthesis, and an increase in JA content was observed [22]. In tobacco and *A. thaliana*, there were indications that SA and JA act synergistically, suggesting that the two pathways regulate defense-related genes together [68,69]. We suggest that, in sweet orange, the three pathways (SA, ET, and JA) somehow interact early to promote an increase in the defense mechanism because we observed the induction of genes involved in ET and JA synthesis (*ACS12* and *OPR3*, respectively) and in the SA pathway (*WRKY*, *PR2*, and *PR9*).

Our analysis demonstrated that the interaction among hormonal pathways promoted by exogenous SA occur together with cellular redox signaling because many hormones produce ROS [63]. A similar interaction has been described previously, and thioredoxins have been linked to the SA signaling cascade. During the SAR, the oligomeric cytosolic protein NPR1 forms monomers [70] to interact with nuclear *TAG* transcription factors, a process that requires thioredoxins [36]. For CHI treatment, we observed fewer transcriptional changes when compared to SA treatment.

#### PR genes

Among the many components that are altered as a result of a plant's defensive response are the *PR* genes, which may accumulate locally or systemically and are associated with the development of SAR. The induction of resistance may culminate with the expression of *PR* genes, and both CHI and SA treatments have been demonstrated to induce these genes in other plants (Figure 3) [5,7,9,71-73]. The regulation of a number of genes encoding putative PR proteins was observed, such as trypsin and protease inhibitor/Kunitz family proteins, several disease resistance proteins (CC-NBS-LRR class), peroxidase (*PR9*) and glucanase (*PR2*), as well as a number of genes that were annotated as “pathogenesis-related” but not possessing homology to known PR genes (Additional file 3: Table S3 and Additional file 6: S5). Peroxidases and glucanases appeared to be induced by SA treatment, as assessed by RNA-seq and qRT-PCR (Table 3; Figure 2), but not by CHI treatment. The peroxidases are important for catalyzing lignin deposition in the cell wall, an important event in the primary defense against pathogens. Similarly, β-1,3-glucanase is a member of a family with antimicrobial properties [74]. Our results suggest that the expression of *PR* genes following the application of SA may contribute to increased plant resistance against pathogens in sweet orange. Thus, additional studies are being conducted to evaluate the behavior of these genes in treated plants with SA and changelled with pathogens, and also to evaluate other plant tissue.

## Conclusions

Using Illumina sequencing technology, we investigated the transcriptome of plants treated with SA and CHI and identified differentially expressed genes, with more genes being influenced by SA treatment than by CHI treatment. Analysis of the annotated genes showed significant increases in the expression levels of genes involved in signaling, biotic stress (redox state, secondary metabolism, *PR* genes) and hormonal interaction, especially for SA treatment. SA treatment altered the expression of nearly twice the number of genes when compared to CHI treatment. We observed responses typical of endogenous SA, such as an increase in *WRKY* transcriptional factors (in this case, *WRKY50*) and *PR* genes. Interestingly, our results did not show the suppression of ET/JA synthesis after the exogenous application of SA, but we did observe the suppression of the auxin pathway and the ABA pathway. Although CHI treatment promoted fewer changes in the transcriptional profile compared to SA treatment, it is possible that the elicitors promote the “priming phenomenon”; in this case, an increase in the capacity for the rapid and effective activation of defense mechanisms occurs only after contact with the pathogen, which involves *PR* genes, an oxidative burst, cell wall lignification, and the secretion of phytoalexins [75]. Thus, we expanded the knowledge regarding defense induced by elicitors in a species of citrus that is susceptible to various diseases as well as the molecular mechanisms mediated by SA/CHI treatments. Additional studies are being conducted to evaluate the effects of these compounds against citrus pathogens in leaves and other plant tissues.

## Methods

### Plant materials and treatment with elicitors

Seven-month-old sweet orange cv. Pera (*Citrus sinensis*) plants grafted onto Rangpur lime were selected from a uniform population and used in the experiment. SA (Sigma Aldrich Chemicals) was dissolved in 10% ethanol and the CHI was prepared in 0.05 N hydrochloric acid, and the pH was adjusted to 5.6 with NaOH before use. The concentrations were determined in a preliminary experiment in which plants healthy were treated as follows: SA at concentrations of 0, 1.25, 2.5 and 5 mM, and CHI 0, 1, 2 and 4 mg/ml. Solutions of 10% ethanol (E) and 0.05 N hydrochloric acid (H), pH 5.6, were used as controls (mock) for SA and CHI, respectively. After 1, 12, 24 and 48 hours of spraying elicitors until point of running off the leaves, leaf samples were collected, immediately placed in liquid nitrogen and stored at −80°C until extraction of RNA for analysis of expression of four genes related to resistance (*NPR1*-3 *WRKY70*, *PR1* and *PR4*) by qRT-PCR. These genes were selected because it is known that their orthologues participate in pathways regulated by SA or ethylene in Arabidopsis [49,61].

After determine the best conditions (concentration and intervals) which promoted higher expression of these defense genes, was conduced the experiment to evaluate the transcriptome. Three plants were used for each treatment and were sprayed with the treatment solutions. Young leaf samples (two leaves per plant) from the three replicates were harvested 24 h post-treatment for 2.5 mM SA and 48 h post-treatment for 4 mg/mL CHI, immediately frozen in liquid nitrogen, and stored at −80°C for RNA extraction.

### RNA extraction and RNA-seq preparation

Total RNA was extracted from 100 mg of fresh tissue from each replicate with Tri Reagent (Life Technologies, Foster City, CA), according to the manufacturer’s protocol. The total RNA was treated with RNase-free DNase (Qiagen, Maryland, USA), and then the replicates from each treatment were combined in a pool (10 μg) according to Venturini et al. (2013) [76] and sent to Macrogen (South Korea) for mRNA purification, cDNA library construction and sequencing, using the *Genome Analyzer IIx platform* (Illumina/Solexa technology).

#### Sequence analysis

The raw data from the RNA-seq in Fasta format and with a quality score of Phred ≥ 20 were indexed, trimmed, and aligned. The transcripts were mapped against the whole reference genome of *Citrus clementina* [77] by using TopHat software [78]. An initial consensus of the exon sequences was extracted from the mapped reads and was used to measure the relative abundance of transcripts, with Cufflink software [79]. A quantitative evaluation of the transcripts was used to calculate the levels of differential expression between the treatment and control groups and their levels of significance using Cuffdiff software [79]. These softwares are commonly used for differential expression analysis for RNA-seq samples [80]. The differentially expressed transcripts (*P* ≤ 0.05 and ≥ 1 fold change) were annotated and categorized automatically on GO (Gene Ontology - https://www.blast2go.com/). The functions of the identified genes were validated using BLASTx data from *A. thaliana.* PageMan [25] and MapMan 3.5.1R2 [26] softwares were used to visualize any functional classes that were significantly altered by the treatments.

To validate the mRNA abundance of 15 genes found to be significantly regulated by the compounds during the RNA-seq analysis, qRT-PCR was also performed. cDNAs were generated using the same RNA samples as those used for the RNA-seq experiment. For each sample, 1 μg of total RNA was used with the RevertAid™ H Minus First Strand cDNA Synthesis Kit, according to the manufacturer’s protocol (Fermentas, USA). The cDNA was diluted in RNase-free water (1:25) and stored at −80°C until used for qRT-PCR analysis. The qRT-PCR assay was performed with three technical replicates using the Fast SYBR Green Master Mix (Life Tecnhologies, Foster City, CA) on an ABI 7500 Real Time PCR system in a total volume of 20 μL. The PCR cycle consisted of one 20 s cycle at 95°C, followed by 40 cycles at 95°C for 3 s and 60°C for 30 s. All amplified products were subjected to melt curve analysis. A negative control without a cDNA template was run with all analyses to evaluate the overall specificity. The reference genes ubiquitin and cyclophilin were used to normalize the total amount of cDNA in each reaction. These genes were the most stable, as was also reported by Rodrigues et al. [80]. Then, amplification efficiency and relative gene expression levels were calculated using *Miner* tool [81] and GenEx 4.3.5. To assess the correlation between different analyses, Pearson correlations were calculated using Bioestat 5.0 [82] to compare the gene expression levels measured by RNA-seq and qRT-PCR. To assess the significant differences between treatments, Student’s t-test for independent samples was calculated using Bioestat 5.0. Gene-specific primers were designed using Primer premier 5.0 software and were synthesized by Exxtend (São Paulo, Brazil). Detailed information regarding the selected genes can be found in Additional file 9: Table S7.

### Availability of supporting data

The data sets supporting the results of this article are available in the NCBI Sequence Read Archive repository, Bioproject PRJNA261357 in http://www.ncbi.nlm.nih.gov/bioproject/?term=PRJNA261357, SRR1581242, SRR1581243, SRR1581244, and SRR1581245.

## Additional files

Additional file 1: Table S1. Differential expression analysis in SA-treated plants. Additional file 2: Table S2. Differential expression analysis in CHI-treated plants. Additional file 3: Table S3. Biological processes altered by SA treatment. Additional file 4: Table S4-1. Differentially expressed genes that were upregulated in SA-treated plants. Additional file 5: Table S4-2. Differentially expressed genes that were downregulated in SA-treated plants. Additional file 6: Table S5. Biological processes altered by CHI treatment. Additional file 7: Table S6-1. Differentially expressed genes that were upregulated in CHI-treated plants. Additional file 8: Table S6-2. Differentially expressed genes that were downregulated in CHI-treated plants. Additional file 9: Table S7. Primers used for validation in qRT-PCR.

## Abbreviations

SA: Salicylic acid
CHI: Chitosan
DEGs: Differentially expressed genes
qRT-PCR: Quantitative Real-Time PCR
IMP: Integrated management of plant
SAR: Systemic acquired resistance
PR: Pathogenesis-related
A1MV: Alfalfa mosaic virus
JA: Jasmonic acid
MeJA: Methyl jasmonate
2OG: 2-oxoglutarate
LRR: Leucine rich repeats
RLK: Receptor-like kinase
PLC: Phosphoinositide-specific phospholipase C
NO: Nitric oxide
ROS: Reactive oxygen species
BTH: Benzothiadiazole S-methylester
ET: Ethylene
ABA: Abscisic acid
GO: Gene Ontology
