# Supplementary material for: Transcriptional profile of sweet orange in response to chitosan and salicylic acid
Source: BMC Genomics. 2015 Apr 12;16(1):288. doi: 10.1186/s12864-015-1440-5 (PMC4415254; doi:10.1186/s12864-015-1440-5)
Supplement: Additional file 4: Table S4-1. — Differentially expressed genes that were upregulated in SA-treated plants. [file 12864_2015_1440_MOESM4_ESM.docx]

**Table S4-1** Differentially expressed genes that were up-regulated in SA-treated plants.

| **Locus** | **Log2**  **(fold_change)*** | **p_value** | **ID**** | ***Arabidopsis thaliana****** | **Gene** | **Description** |
| --- | --- | --- | --- | --- | --- | --- |
| 1. clementina_scaffold_19:2869666-2873408 | 4.07726 | 9.11453e-06 | clementine0.9_028869m | AT4G20980.1 |  | Eukaryotic translation initiation factor 3 subunit 7 (eIF-3) |
| 1. clementina_scaffold_65:649467-651972 | 3.58636 | 0.00129205 | clementine0.9_022066m | AT1G12880.1 | NUDT12 | nudix (nucleoside diphosphate linked moiety ) hydrolase homolog 12 |
| 1. clementina_scaffold_16:256470-259319 | 3.51525 | 6.58828e-09 | clementine0.9_008114m | AT5G19500.1 |  | Tryptophan/tyrosine permease |
| 1. clementina_scaffold_3:3540417-3541811 | 3.19388 | 7.31653e-07 | clementine0.9_015054m | AT5G17820.1 |  | Peroxidase superfamily protein |
| 1. clementina_scaffold_7:2099863-2100791 | 3.00814 | 0.00237499 | clementine0.9_026555m | AT5G02580.1 |  | Plant protein 1589 of unknown function |
| 1. clementina_scaffold_4:4736629-4744896 | 2.96532 | 0.000500128 | clementine0.9_032356m | AT4G25050.1 | ACP4 | acyl carrier protein 4 |
| 1. clementina_scaffold_15:2577520-2580444 | 2.72279 | 0 | clementine0.9_015878m | AT1G61800.1 | GPT2 | glucose-6-phosphate/phosphate translocator 2 |
| 1. clementina_scaffold_19:3055984-3060307 | 2.70432 | 0 | clementine0.9_007959m | AT4G04955.1 | ALN | allantoinase |
| 1. clementina_scaffold_70:224998-226108 | 2.52697 | 4.8232e-05 | clementine0.9_027700m | AT1G64830.1 |  | Eukaryotic aspartyl protease family protein |
| 1. clementina_scaffold_38:907804-908722 | 2.51936 | 0.00220683 | clementine0.9_021048m | AT1G17860.1 |  | Kunitz family trypsin and protease inhibitor protein |
| 1. clementina_scaffold_111:585867-588096 | 2.49997 | 0.00166301 | clementine0.9_026758m | AT3G61110.1 | RS27A | ribosomal protein S27 |
| 1. clementina_scaffold_15:4458333-4462236 | 2.48595 | 0.00220282 | clementine0.9_020269m | AT5G20920.1 | EIF2 BETA | eukaryotic translation initiation factor 2 beta subunit |
| 1. clementina_scaffold_27:3167229-3169873 | 2.44192 | 0.000467853 | clementine0.9_024961m | AT2G33370.1 |  | Ribosomal protein L14p/L23e family protein |
| 1. clementina_scaffold_1:6735068-6736006 | 2.26847 | 0.0021337 | clementine0.9_020612m | AT2G28190.1 | CSD2 | copper/zinc superoxide dismutase 2 |
| 1. clementina_scaffold_29:2049389-2050425 | 2.25556 | 1.2772e-12 | clementine0.9_025307m |  |  |  |
| 1. clementina_scaffold_2:292394-293611 | 2.19064 | 0.000859495 | clementine0.9_018843m | AT3G09250.1 |  | Nuclear transport factor 2 (NTF2) family protein |
| 1. clementina_scaffold_152:97-1429 | 2.17642 | 9.76996e-15 | clementine0.9_031181m |  |  |  |
| 1. clementina_scaffold_27:1754231-1755170 | 2.16302 | 0.00220864 | clementine0.9_025823m |  |  |  |
| 1. clementina_scaffold_8:1681144-1681204 | 2.15813 | 3.09891e-09 | clementine0.9_024036m | AT1G07400.1 |  | HSP20-like chaperones superfamily protein |
| 1. clementina_scaffold_3:152928-156527 | 2.14165 | 8.67581e-06 | clementine0.9_002014m | AT2G44950.1 | HUB1 | histone mono-ubiquitination 1 |
| 1. clementina_scaffold_7:140213-141706 | 2.13645 | 4.4315e-07 | clementine0.9_026895m | ATCG00140.1 | ATPH | ATP synthase subunit C family protein |
| 1. clementina_scaffold_29:2073934-2075735 | 2.10833 | 0.00244485 | clementine0.9_018640m | AT3G29270.1 |  | RING/U-box superfamily protein |
| 1. clementina_scaffold_66:1159162-1159277 | 2.0864 | 9.21841e-10 | clementine0.9_035745m | AT3G28890.1 | RLP43 | receptor like protein 43 |
| 1. clementina_scaffold_13:2533557-2535238 | 2.05923 | 5.09103e-06 | clementine0.9_014533m | AT3G57260.1 | BGL2 | beta-1.3-glucanase 2 |
| 1. clementina_scaffold_14:1258079-1258989 | 2.04303 | 1.19038e-05 | clementine0.9_020626m | AT1G17860.1 |  | Kunitz family trypsin and protease inhibitor protein |
| 1. clementina_scaffold_100:816744-818899 | 2.02704 | 5.34486e-10 | clementine0.9_012490m |  |  |  |
| 1. clementina_scaffold_55:101002-102221 | 1.96312 | 0.000468369 | clementine0.9_008552m | AT3G50930.1 | BCS1 | cytochrome BC1 synthesis |
| 1. clementina_scaffold_32:1685474-1687324 | 1.95596 | 1.32871e-12 | clementine0.9_017427m | AT3G16150.1 |  | N-terminal nucleophile aminohydrolases (Ntn hydrolases) superfamily protein |
| 1. clementina_scaffold_116:38094-41105 | 1.92677 | 0.00210374 | clementine0.9_010612m | AT1G73740.1 |  | UDP-Glycosyltransferase superfamily protein |
| 1. clementina_scaffold_89:419608-420688 | 1.88551 | 0.00206933 | clementine0.9_026190m |  |  |  |
| 1. clementina_scaffold_33:1366464-1367531 | 1.83069 | 8.94604e-06 | clementine0.9_026469m | AT1G52240.2 | ROPGEF11 | RHO guanyl-nucleotide exchange factor 11 |
| 1. clementina_scaffold_1:11563032-11566277 | 1.79769e+308 | 0.00217184 | clementine0.9_031776m | AT4G09020.1 | ISA3 | isoamylase 3 |
| 1. clementina_scaffold_1:4246404-4250120 | 1.79769e+308 | 1.73966e-05 | clementine0.9_017178m | AT5G45390.1 | CLPP4 | CLP protease P4 |
| 1. clementina_scaffold_1:7221807-7224549 | 1.79769e+308 | 0.00174334 | clementine0.9_036017m | AT4G27220.1 |  | NB-ARC domain-containing disease resistance protein |
| 1. clementina_scaffold_10:3131378-3136731 | 1.79769e+308 | 0.00121359 | clementine0.9_011479m | AT2G03620.1 | MGT3 | magnesium transporter 3 |
| 1. clementina_scaffold_13:1109960-1112264 | 1.79769e+308 | 0.0018773 | clementine0.9_008352m | AT3G48150.1 | APC8 | anaphase-promoting complex subunit 8 |
| 1. clementina_scaffold_135:195577-198271 | 1.79769e+308 | 0.00164546 | clementine0.9_020835m | AT1G25260.1 |  | Ribosomal protein L10 family protein |
| 1. clementina_scaffold_138:253088-256254 | 1.79769e+308 | 0.00285783 | clementine0.9_005552m | AT1G59740.1 |  | Major facilitator superfamily protein |
| 1. clementina_scaffold_147:66792-67786 | 1.79769e+308 | 0.000320121 | clementine0.9_014263m | AT4G10490.1 |  | 2-oxoglutarate (2OG) and Fe(II)-dependent oxygenase superfamily protein |
| 1. clementina_scaffold_15:3114907-3119621 | 1.79769e+308 | 3.77016e-05 | clementine0.9_031858m | AT2G34660.1 | MRP2 | multidrug resistance-associated protein 2 |
| 1. clementina_scaffold_19:61680-64330 | 1.79769e+308 | 0.00230812 | clementine0.9_007608m | AT4G17180.1 |  | O-Glycosyl hydrolases family 17 protein |
| 1. clementina_scaffold_2:5184711-5187403 | 1.79769e+308 | 0.000630598 | clementine0.9_010395m | AT3G02875.1 | ILR1 | Peptidase M20/M25/M40 family protein |
| 1. clementina_scaffold_2:407124-409059 | 1.79769e+308 | 9.30005e-05 | clementine0.9_010238m | AT5G62690.1 | TUB2 | tubulin beta chain 2 |
| 1. clementina_scaffold_20:907084-909266 | 1.79769e+308 | 0.00236046 | clementine0.9_009246m | AT3G26410.1 | TRM11 | methyltransferases;nucleic acid binding |
| 1. clementina_scaffold_22:1661791-1664594 | 1.79769e+308 | 0.00269081 | clementine0.9_012259m | AT5G42520.1 | BPC6 | basic pentacysteine 6 |
| 1. clementina_scaffold_22:1945951-1947225 | 1.79769e+308 | 0.00238051 | clementine0.9_004216m | AT1G75410.1 | BLH3 | BEL1-like homeodomain 3 |
| 1. clementina_scaffold_3:5369887-5373164 | 1.79769e+308 | 0.000850668 | clementine0.9_007770m | AT2G30780.1 |  | Tetratricopeptide repeat (TPR)-like superfamily protein |
| 1. clementina_scaffold_3:5722432-5724209 | 1.79769e+308 | 0.0024256 | clementine0.9_014388m | AT3G63110.1 | IPT3 | isopentenyltransferase 3 |
| 1. clementina_scaffold_34:253595-255756 | 1.79769e+308 | 0.000809786 | clementine0.9_014253m | AT1G21710.1 | OGG1 | 8-oxoguanine-DNA glycosylase 1 |
| 1. clementina_scaffold_34:2054446-2056347 | 1.79769e+308 | 0.00196032 | clementine0.9_027226m | AT5G41685.1 |  | Mitochondrial outer membrane translocase complex. subunit Tom7 |
| 1. clementina_scaffold_42:563633-566117 | 1.79769e+308 | 0.000422417 | clementine0.9_011268m | AT2G36950.1 |  | Heavy metal transport/detoxification superfamily protein |
| 1. clementina_scaffold_55:112165-114211 | 1.79769e+308 | 0.00136827 | clementine0.9_008995m | AT3G50930.1 | BCS1 | cytochrome BC1 synthesis |
| 1. clementina_scaffold_58:132483-137436 | 1.79769e+308 | 0.000808493 | clementine0.9_007823m | AT4G26270.1 | PFK3 | phosphofructokinase 3 |
| 1. clementina_scaffold_6:2887398-2888269 | 1.79769e+308 | 0.00197388 | clementine0.9_012078m | AT4G10490.1 |  | 2-oxoglutarate (2OG) and Fe(II)-dependent oxygenase superfamily protein |
| 1. clementina_scaffold_61:785918-786447 | 1.79769e+308 | 0.00128442 | clementine0.9_030420m | AT1G02230.1 | NAC004 | NAC domain containing protein 4 |
| 1. clementina_scaffold_80:293861-294458 | 1.79769e+308 | 0.00168522 | clementine0.9_007011m | AT5G46250.1 |  | RNA-binding protein |
| 1. clementina_scaffold_17:1206401-1209388 | 1.79381 | 0.000971548 | clementine0.9_002252m | AT3G14590.2 | NTMC2T6.2 | Calcium-dependent lipid-binding (CaLB domain) family protein |
| 1. clementina_scaffold_14:1122907-1124303 | 1.73999 | 8.85956e-08 | clementine0.9_015518m | AT1G19670.1 | CLH1 | chlorophyllase 1 |
| 1. clementina_scaffold_2:4532193-4536262 | 1.73409 | 7.63899e-05 | clementine0.9_015575m | AT5G06570.1 |  | alpha/beta-Hydrolases superfamily protein |
| 1. clementina_scaffold_8:3035566-3036317 | 1.70851 | 8.04968e-06 | clementine0.9_026130m | AT3G07450.1 |  | Bifunctional inhibitor/lipid-transfer protein/seed storage 2S albumin superfamily protein |
| 1. clementina_scaffold_6:1529530-1531010 | 1.67913 | 0.000872714 | clementine0.9_014789m | AT3G49340.1 |  | Cysteine proteinases superfamily protein |
| 1. clementina_scaffold_61:1416761-1419330 | 1.66481 | 4.82108e-05 | clementine0.9_008524m | AT2G32440.1 | KAO2 | ent-kaurenoic acid hydroxylase 2 |
| 1. clementina_scaffold_14:1226568-1227574 | 1.6434 | 7.26882e-09 | clementine0.9_020626m | AT1G17860.1 |  | Kunitz family trypsin and protease inhibitor protein |
| 1. clementina_scaffold_34:780802-782001 | 1.63894 | 3.79703e-05 | clementine0.9_015053m | AT1G31130.1 |  |  |
| 1. clementina_scaffold_10:2914898-2915636 | 1.63519 | 0.00013174 | clementine0.9_022359m |  |  |  |
| 1. clementina_scaffold_3:5108723-5109471 | 1.61359 | 1.69531e-09 | clementine0.9_026296m | AT2G30540.1 |  | Thioredoxin superfamily protein |
| 1. clementina_scaffold_1:10081153-10082166 | 1.60996 | 0.000281858 | clementine0.9_019994m | AT1G27730.1 | STZ | salt tolerance zinc finger |
| 1. clementina_scaffold_23:1963538-1964319 | 1.60286 | 1.31691e-08 | clementine0.9_026933m | AT3G49780.1 | PSK4 | phytosulfokine 4 precursor |
| 1. clementina_scaffold_21:502255-503503 | 1.54638 | 0.000165786 | clementine0.9_026022m | AT2G05540.1 |  | Glycine-rich protein family |
| 1. clementina_scaffold_6:3423952-3427734 | 1.544 | 1.99278e-05 | clementine0.9_034737m | AT5G51690.1 | ACS12 | 1-amino-cyclopropane-1-carboxylate synthase 12 |
| 1. clementina_scaffold_12:4213281-4213990 | 1.5408 | 5.65781e-05 | clementine0.9_024855m | AT4G23880.1 |  |  |
| 1. clementina_scaffold_66:1191208-1194315 | 1.53097 | 0.00193501 | clementine0.9_023918m |  |  |  |
| 1. clementina_scaffold_68:1095192-1095758 | 1.53 | 0.000175762 | clementine0.9_014419m | AT5G20400.1 |  | 2-oxoglutarate (2OG) and Fe(II)-dependent oxygenase superfamily protein |
| 1. clementina_scaffold_1:10156850-10158820 | 1.52871 | 0.000232078 | clementine0.9_014496m | AT3G01400.1 |  | ARM repeat superfamily protein |
| 1. clementina_scaffold_16:1795400-1798600 | 1.5139 | 0.000209758 | clementine0.9_007376m | AT5G24910.1 | CYP714A1 | cytochrome P450. family 714. subfamily A. polypeptide 1 |
| 1. clementina_scaffold_48:363710-365088 | 1.50906 | 1.25361e-06 | clementine0.9_015084m | AT3G12920.1 |  | SBP (S-ribonuclease binding protein) family protein |
| 1. clementina_scaffold_6:1595729-1596161 | 1.50143 | 0.00179186 | clementine0.9_031631m | AT3G54070.1 |  | Ankyrin repeat family protein |
| 1. clementina_scaffold_16:2933857-2934428 | 1.50087 | 1.59738e-07 | clementine0.9_003288m | AT3G56370.1 |  | Leucine-rich repeat protein kinase family protein |
| 1. clementina_scaffold_1:1701501-1702570 | 1.4861 | 0.000128548 | clementine0.9_020703m | AT3G54420.1 | EP3 | homolog of carrot EP3-3 chitinase |
| 1. clementina_scaffold_7:481908-483468 | 1.47496 | 0.00017765 | clementine0.9_004072m | AT2G37710.1 | RLK | receptor lectin kinase |
| 1. clementina_scaffold_8:867663-869125 | 1.46745 | 0.000776199 | clementine0.9_006081m | AT3G54140.1 | PTR1 | peptide transporter 1 |
| 1. clementina_scaffold_87:822237-823568 | 1.46641 | 0.000553588 | clementine0.9_009651m | AT3G55700.1 |  | UDP-Glycosyltransferase superfamily protein |
| 1. clementina_scaffold_1:1809188-1810583 | 1.45964 | 0.000644981 | clementine0.9_022372m | AT3G49340.1 |  | Cysteine proteinases superfamily protein |
| 1. clementina_scaffold_2:645216-646192 | 1.44988 | 5.67819e-08 | clementine0.9_022857m |  |  |  |
| 1. clementina_scaffold_25:1268363-1269908 | 1.44515 | 0.00208592 | clementine0.9_008439m | AT3G50740.1 | UGT72E1 | UDP-glucosyl transferase 72E1 |
| 1. clementina_scaffold_16:1354059-1355449 | 1.44394 | 6.92428e-08 | clementine0.9_017239m | AT4G01470.1 | TIP1;3 | tonoplast intrinsic protein 1;3 |
| 1. clementina_scaffold_8:4221245-4223843 | 1.43366 | 0.000135087 | clementine0.9_001060m | AT1G48410.1 | ago/01 | Stabilizer of iron transporter SufD / Polynucleotidyl transferase |
| 1. clementina_scaffold_4:1156347-1157005 | 1.42558 | 0.000306676 | clementine0.9_027664m | AT4G31985.1 |  | Ribosomal protein L39 family protein |
| 1. clementina_scaffold_3:4385848-4387099 | 1.42148 | 7.18419e-05 | clementine0.9_024644m | AT1G56300.1 |  | Chaperone DnaJ-domain superfamily protein |
| 1. clementina_scaffold_8:1307162-1317801 | 1.41506 | 1.46276e-05 | clementine0.9_031086m | AT2G29210.1 |  | splicing factor PWI domain-containing protein |
| 1. clementina_scaffold_24:3339075-3340095 | 1.39608 | 0.00161654 | clementine0.9_024472m | AT4G39700.1 |  | Heavy metal transport/detoxification superfamily protein |
| 1. clementina_scaffold_20:3343542-3344897 | 1.36854 | 0.000269469 | clementine0.9_036071m | AT2G23180.1 | CYP96A1 | cytochrome P450. family 96. subfamily A. polypeptide 1 |
| 1. clementina_scaffold_20:3050130-3052885 | 1.36058 | 7.56781e-05 | clementine0.9_009307m | AT5G14570.1 | NRT2.7 | high affinity nitrate transporter 2.7 |
| 1. clementina_scaffold_121:254619-255977 | 1.3562 | 1.12957e-06 | clementine0.9_026599m | AT2G44670.1 |  | Protein of unknown function (DUF581) |
| 1. clementina_scaffold_18:733357-739462 | 1.35594 | 6.59237e-06 | clementine0.9_015118m | AT5G24530.1 | DMR6 | 2-oxoglutarate (2OG) and Fe(II)-dependent oxygenase superfamily protein |
| 1. clementina_scaffold_6:2614452-2616014 | 1.35485 | 7.88403e-10 | clementine0.9_021972m | AT4G25200.1 | HSP23.6-MITO | mitochondrion-localized small heat shock protein 23.6 |
| 1. clementina_scaffold_4:3147363-3151380 | 1.35325 | 0.00108568 | clementine0.9_019894m | AT5G57150.1 |  | basic helix-loop-helix (bHLH) DNA-binding superfamily protein |
| 1. clementina_scaffold_6:1465247-1466327 | 1.34943 | 0.000655948 | clementine0.9_020285m | AT5G51190.1 |  | Integrase-type DNA-binding superfamily protein |
| 1. clementina_scaffold_53:593818-594870 | 1.33576 | 5.91633e-05 | clementine0.9_021807m | AT5G39670.1 |  | Calcium-binding EF-hand family protein |
| 1. clementina_scaffold_68:515338-517105 | 1.32741 | 5.60793e-07 | clementine0.9_007035m | AT3G02645.1 |  | Plant protein of unknown function (DUF247) |
| 1. clementina_scaffold_10:405692-408757 | 1.32394 | 6.114e-08 | clementine0.9_017290m | AT1G10350.1 |  | DNAJ heat shock family protein |
| 1. clementina_scaffold_1:11436463-11439082 | 1.31852 | 0.000462595 | clementine0.9_011370m | AT3G21110.1 | PUR7 | purin 7 |
| 1. clementina_scaffold_8:639956-642963 | 1.31341 | 0.000555938 | clementine0.9_005470m | AT5G58700.1 | PLC4 | phosphatidylinositol-speciwc phospholipase C4 |
| 1. clementina_scaffold_4:4224546-4225810 | 1.31332 | 0.000967513 | clementine0.9_024219m | AT5G26170.1 | WRKY50 | WRKY DNA-binding protein 50 |
| 1. clementina_scaffold_6:4983199-4984453 | 1.3065 | 1.25479e-06 | clementine0.9_025204m | AT1G01490.1 |  | Heavy metal transport/detoxification superfamily protein |
| 1. clementina_scaffold_1:6757785-6759316 | 1.30327 | 0.000587509 | clementine0.9_020612m | AT2G28190.1 | CSD2 | copper/zinc superoxide dismutase 2 |
| 1. clementina_scaffold_1:12244502-12245433 | 1.29627 | 3.75259e-05 | clementine0.9_024593m | AT4G08570.1 |  | Heavy metal transport/detoxification superfamily protein |
| 1. clementina_scaffold_13:1543278-1545527 | 1.29534 | 2.87475e-06 | clementine0.9_005506m | AT3G57150.1 | NAP57 | homologue of NAP57 |
| 1. clementina_scaffold_3:1343221-1345531 | 1.27756 | 0.00275254 | clementine0.9_033013m | AT2G45830.1 | DTA2 | downstream target of AGL15 2 |
| 1. clementina_scaffold_4:1327215-1330416 | 1.26204 | 0.00273185 | clementine0.9_004098m | AT2G25180.1 | RR12 | response regulator 12 |
| 1. clementina_scaffold_19:205914-211482 | 1.25912 | 0.000973087 | clementine0.9_005306m | AT5G47750.1 | D6PKL2 | D6 protein kinase like 2 |
| 1. clementina_scaffold_56:798180-799561 | 1.25149 | 4.14682e-05 | clementine0.9_032939m | AT1G23100.1 |  | GroES-like family protein |
| 1. clementina_scaffold_40:1985658-1986588 | 1.24528 | 0.000248151 | clementine0.9_035746m | ATCG00780.1 | RPL14 | ribosomal protein L14 |
| 1. clementina_scaffold_73:309911-312052 | 1.24049 | 0.000169373 | clementine0.9_028721m | AT1G07390.3 | RLP1 | receptor like protein 1 |
| 1. clementina_scaffold_2:630759-631676 | 1.23673 | 5.92999e-06 | clementine0.9_022857m |  |  |  |
| 1. clementina_scaffold_39:1208356-1210486 | 1.22351 | 5.41145e-12 | clementine0.9_013948m | AT2G38750.1 | ANNAT4 | annexin 4 |
| 1. clementina_scaffold_5:1449697-1451533 | 1.2234 | 0.00207954 | clementine0.9_034817m |  |  |  |
| 1. clementina_scaffold_76:985228-986610 | 1.2097 | 0.000712782 | clementine0.9_027461m | AT1G71400.1 | RLP12 | receptor like protein 12 |
| 1. clementina_scaffold_76:35374-36667 | 1.20925 | 0.00102871 | clementine0.9_014739m | AT1G68620.1 |  | alpha/beta-Hydrolases superfamily protein |
| 1. clementina_scaffold_33:1245260-1246718 | 1.20506 | 0.00222899 | clementine0.9_016825m | AT3G16050.1 | PDX1.2 | pyridoxine biosynthesis 1.2 |
| 1. clementina_scaffold_82:704210-708681 | 1.18624 | 4.36471e-05 | clementine0.9_017496m | AT5G20060.1 |  | alpha/beta-Hydrolases superfamily protein |
| 1. clementina_scaffold_2:652245-653405 | 1.18502 | 3.3525e-05 | clementine0.9_024080m |  |  |  |
| 1. clementina_scaffold_89:498306-500406 | 1.18315 | 0.00087468 | clementine0.9_029516m | AT1G35710.1 |  | Protein kinase family protein with leucine-rich repeat domain |
| 1. clementina_scaffold_1:1547977-1548587 | 1.17951 | 0.00175914 | clementine0.9_028785m | AT3G49670.1 | BAM2 | Leucine-rich receptor-like protein kinase family protein |
| 1. clementina_scaffold_29:674409-677857 | 1.17506 | 0.00176385 | clementine0.9_021356m | AT3G18580.1 |  | Nucleic acid-binding. OB-fold-like protein |
| 1. clementina_scaffold_14:1248463-1249606 | 1.16442 | 0.000283536 | clementine0.9_020424m | AT1G17860.1 |  | Kunitz family trypsin and protease inhibitor protein |
| 1. clementina_scaffold_95:628661-629626 | 1.16051 | 0.000103475 | clementine0.9_024362m | AT5G14030.1 |  | translocon-associated protein beta (TRAPB) family protein |
| 1. clementina_scaffold_56:156883-158491 | 1.15639 | 0.00223076 | clementine0.9_004661m | AT2G01730.1 | CPSF73-II | cleavage and polyadenylation specificity factor 73 kDa subunit-II |
| 1. clementina_scaffold_151:88790-90345 | 1.15173 | 0.00243989 | clementine0.9_026684m | AT2G44360.1 |  |  |
| 1. clementina_scaffold_10:2955190-2956758 | 1.14091 | 3.43626e-05 | clementine0.9_012925m | AT1G12060.1 | BAG5 | BCL-2-associated athanogene 5 - apoptose |
| 1. clementina_scaffold_63:1343548-1350328 | 1.13836 | 2.45096e-08 | clementine0.9_020138m | AT2G18950.1 | HPT1 | homogentisate phytyltransferase 1 |
| 1. clementina_scaffold_26:2927475-2929010 | 1.13054 | 2.12398e-05 | clementine0.9_017974m | AT2G17040.1 | NAC036 | NAC domain containing protein 36 |
| 1. clementina_scaffold_6:4955873-4958098 | 1.1304 | 2.87305e-05 | clementine0.9_003850m | AT5G52640.1 | HSP90.1 | heat shock protein 90.1 |
| 1. clementina_scaffold_2:3495787-3497662 | 1.12314 | 0.000210129 | clementine0.9_012628m | AT3G12750.1 | ZIP1 | zinc transporter 1 precursor |
| 1. clementina_scaffold_7:139080-140091 | 1.12222 | 0.00151027 | clementine0.9_021312m | AT5G59770.1 |  | Protein-tyrosine phosphatase-like. PTPLA |
| 1. clementina_scaffold_2:3176194-3178406 | 1.11974 | 0.00166753 | clementine0.9_004276m | AT1G03910.1 |  |  |
| 1. clementina_scaffold_126:56334-60496 | 1.11689 | 1.5762e-06 | clementine0.9_001984m | AT4G03230.1 |  | S-locus lectin protein kinase family protein |
| 1. clementina_scaffold_8:3927932-3932347 | 1.11641 | 7.4877e-10 | clementine0.9_020612m | AT2G28190.1 | CSD2 | copper/zinc superoxide dismutase 2 |
| 1. clementina_scaffold_4:6719012-6720755 | 1.10855 | 0.000358259 | clementine0.9_017480m | AT5G54630.1 |  | zinc finger protein-related |
| 1. clementina_scaffold_10:588736-590634 | 1.10552 | 1.20269e-08 | clementine0.9_016354m | AT1G70000.1 |  | myb-like transcription factor family protein |
| 1. clementina_scaffold_32:551465-558681 | 1.10523 | 0.00102954 | clementine0.9_006738m | AT1G72770.1 | HAB1 | homology to ABI1 |
| 1. clementina_scaffold_68:1118589-1121965 | 1.10454 | 0.00116189 | clementine0.9_033919m | AT1G74190.1 | RLP15 | receptor like protein 15 |
| 1. clementina_scaffold_3:2866194-2869954 | 1.10137 | 0.000392721 | clementine0.9_005293m | AT1G32900.1 |  | UDP-Glycosyltransferase superfamily protein |
| 1. clementina_scaffold_40:46599-50271 | 1.09988 | 4.92269e-05 | clementine0.9_020704m | AT4G29670.1 | ACHT2 | atypical CYS HIS rich thioredoxin 2 |
| 1. clementina_scaffold_34:1760168-1763591 | 1.09852 | 1.86149e-06 | clementine0.9_027469m | AT1G71710.2 |  | DNAse I-like superfamily protein |
| 1. clementina_scaffold_63:817438-819445 | 1.09795 | 0.000161718 | clementine0.9_009172m | AT5G10770.1 |  | Eukaryotic aspartyl protease family protein |
| 1. clementina_scaffold_7:6400491-6402074 | 1.09143 | 0.00146991 | clementine0.9_022187m | AT5G30495.1 |  | Fcf2 pre-rRNA processing protein |
| 1. clementina_scaffold_22:1639362-1641355 | 1.08981 | 0.00208332 | clementine0.9_008057m | AT4G31500.1 | CYP83B1 | cytochrome P450. family 83. subfamily B. polypeptide 1 |
| 1. clementina_scaffold_48:273406-277173 | 1.08464 | 1.02125e-07 | clementine0.9_024634m | AT5G53420.3 |  | CCT motif family protein |
| 1. clementina_scaffold_17:1245053-1247743 | 1.08261 | 0.000614499 | clementine0.9_021082m | AT2G34480.1 |  | Ribosomal protein L18ae/LX family protein |
| 1. clementina_scaffold_55:262390-268095 | 1.0768 | 2.56434e-07 | clementine0.9_007281m | AT4G39210.1 | APL3 | Glucose-1-phosphate adenylyltransferase family protein |
| 1. clementina_scaffold_7:5302896-5304247 | 1.07082 | 0.000244524 | clementine0.9_024116m | AT5G45860.1 | PYL11 | PYR1-like 11 - abscisic acid receptor PYL11 |
| 1. clementina_scaffold_12:3253474-3258781 | 1.06683 | 0.00144148 | clementine0.9_005822m | AT4G11560.1 |  | bromo-adjacent homology (BAH) domain-containing protein |
| 1. clementina_scaffold_3:8428977-8430814 | 1.06619 | 0.00163696 | clementine0.9_007355m | AT3G25180.1 | CYP82G1 | cytochrome P450. family 82. subfamily G. polypeptide 1 |
| 1. clementina_scaffold_6:1456547-1457813 | 1.06459 | 0.000988597 | clementine0.9_017800m | AT5G51190.1 |  | Integrase-type DNA-binding superfamily protein |
| 1. clementina_scaffold_35:470223-476351 | 1.06273 | 1.0003e-07 | clementine0.9_013848m | AT1G08450.1 | CRT3 | calreticulin 3 |
| 1. clementina_scaffold_4:5306842-5308773 | 1.06035 | 5.9357e-05 | clementine0.9_014799m | AT3G63010.1 | GID1B | alpha/beta-Hydrolases superfamily protein |
| 1. clementina_scaffold_74:814403-818290 | 1.05978 | 0.000234113 | clementine0.9_002139m | AT2G24030.1 |  | zinc ion binding;nucleic acid binding |
| 1. clementina_scaffold_2:4541761-4545231 | 1.05955 | 0.000412746 | clementine0.9_019395m | AT5G06570.1 |  | alpha/beta-Hydrolases superfamily protein |
| 1. clementina_scaffold_68:1099005-1104971 | 1.05907 | 0.00130845 | clementine0.9_003029m | AT1G15690.1 | AVP1 | Inorganic H pyrophosphatase family protein |
| 1. clementina_scaffold_24:1507707-1510068 | 1.05853 | 0.000103831 | clementine0.9_020217m | AT2G21870.1 | MGP1 | copper ion binding;cobalt ion binding;zinc ion binding |
| 1. clementina_scaffold_3:1479277-1480818 | 1.05545 | 0.00213047 | clementine0.9_026069m |  |  |  |
| 1. clementina_scaffold_49:896060-897075 | 1.05513 | 0.000289213 | clementine0.9_024002m | AT3G07600.1 |  | Heavy metal transport/detoxification superfamily protein |
| 1. clementina_scaffold_44:63540-64784 | 1.05283 | 0.000156375 | clementine0.9_016721m | AT4G05320.2 | UBQ10 | polyubiquitin 10 |
| 1. clementina_scaffold_14:948846-951100 | 1.05006 | 0.000350602 | clementine0.9_032775m | AT1G28150.1 |  |  |
| 1. clementina_scaffold_287:6545-9380 | 1.04916 | 2.73086e-07 | clementine0.9_004603m | AT3G12580.1 | HSP70 | heat shock protein 70 |
| 1. clementina_scaffold_12:3648596-3651770 | 1.04871 | 8.32484e-05 | clementine0.9_023765m | AT4G23880.1 |  |  |
| 1. clementina_scaffold_99:536331-539045 | 1.04715 | 1.07577e-06 | clementine0.9_006332m | AT5G23960.2 | TPS21 | terpene synthase 21 |
| 1. clementina_scaffold_64:344772-348601 | 1.04694 | 0.000113917 | clementine0.9_023000m | AT5G54750.1 |  | Transport protein particle (TRAPP) component |
| 1. clementina_scaffold_23:1809096-1811609 | 1.04678 | 2.26011e-05 | clementine0.9_009481m | AT5G67630.1 |  | P-loop containing nucleoside triphosphate hydrolases superfamily protein |
| 1. clementina_scaffold_46:754758-756498 | 1.04542 | 0.000136056 | clementine0.9_027298m | AT3G59540.1 |  | Ribosomal L38e protein family |
| 1. clementina_scaffold_12:839620-842606 | 1.04514 | 5.90553e-05 | clementine0.9_021557m | AT5G50375.2 | CPI1 | cyclopropyl isomerase |
| 1. clementina_scaffold_23:2653067-2655761 | 1.03919 | 0.000137239 | clementine0.9_013884m | AT4G37990.1 | ELI3-2 | elicitor-activated gene 3-2 |
| 1. clementina_scaffold_7:4021525-4024769 | 1.02971 | 0.000123317 | clementine0.9_022279m | ATCG00810.1 | RPL22 | ribosomal protein L22 |
| 1. clementina_scaffold_26:285775-287896 | 1.02949 | 0.00162383 | clementine0.9_005074m | AT2G15690.1 |  | Tetratricopeptide repeat (TPR)-like superfamily protein |
| 1. clementina_scaffold_22:928301-931988 | 1.02946 | 2.61339e-05 | clementine0.9_032756m | AT1G76130.1 | AMY2 | alpha-amylase-like 2 |
| 1. clementina_scaffold_6:6502238-6504011 | 1.02944 | 0.000350187 | clementine0.9_007484m | AT1G79360.1 | 2-Oct | organic cation/carnitine transporter 2 |
| 1. clementina_scaffold_22:2902124-2903992 | 1.02534 | 6.07142e-07 | clementine0.9_019308m | AT2G06050.1 | OPR3 | oxophytodienoate-reductase 3 |
| 1. clementina_scaffold_74:1108246-1115226 | 1.02448 | 0.00043865 | clementine0.9_013167m | AT3G52180.1 | SEX4 | dual specificity protein phosphatase (DsPTP1) family protein |
| 1. clementina_scaffold_3:7258044-7261636 | 1.02397 | 0.000366052 | clementine0.9_021676m | AT1G03950.1 | VPS2.3 | vacuolar protein sorting-associated protein 2.3 |
| 1. clementina_scaffold_56:117079-128383 | 1.01936 | 0.000906815 | clementine0.9_005046m | AT1G14830.1 | DL1C | DYNAMIN-like 1C |
| 1. clementina_scaffold_32:2390962-2394645 | 1.01832 | 0.0008923 | clementine0.9_014478m | AT5G42630.1 | ATS | Homeodomain-like superfamily protein |
| 1. clementina_scaffold_19:1389803-1391630 | 1.01721 | 0.000960883 | clementine0.9_025101m | AT2G34450.1 |  | HMG-box (high mobility group) DNA-binding family protein |
| 1. clementina_scaffold_3:7541270-7543188 | 1.00113 | 3.31717e-06 | clementine0.9_013433m | AT5G20190.1 |  | Tetratricopeptide repeat (TPR)-like superfamily protein |
| 1. clementina_scaffold_91:601568-603258 | 0.998879 | 0.00115288 | clementine0.9_011165m | AT5G33340.1 | CDR1 | Eukaryotic aspartyl protease family protein |
| 1. clementina_scaffold_20:679244-683220 | 0.995274 | 0.000768884 | clementine0.9_003178m |  |  |  |
| 1. clementina_scaffold_132:306073-307681 | 0.993887 | 0.000147254 | clementine0.9_027268m | AT4G21105.1 |  | cytochrome-c oxidases;electron carriers |
| 1. clementina_scaffold_2:6508343-6511119 | 0.992098 | 0.00026572 | clementine0.9_018490m | AT1G12520.1 | CCS | copper chaperone for SOD1 |
| 1. clementina_scaffold_32:512803-516266 | 0.990745 | 0.00150285 | clementine0.9_011561m | AT3G13920.1 | EIF4A1 | eukaryotic translation initiation factor 4A1 |
| 1. clementina_scaffold_21:1261132-1265976 | 0.990012 | 0.000315863 | clementine0.9_008208m | AT4G15440.1 | HPL1 | hydroperoxide lyase 1 |
| 1. clementina_scaffold_13:4530191-4533044 | 0.987917 | 0.000449891 | clementine0.9_025477m | AT5G58240.1 | FHIT | FRAGILE HISTIDINE TRIAD |
| 1. clementina_scaffold_12:2136636-2138731 | 0.984528 | 0.00102886 | clementine0.9_017112m | AT2G36330.1 |  | Uncharacterised protein family (UPF0497) |
| 1. clementina_scaffold_67:1007074-1014302 | 0.982787 | 4.43256e-05 | clementine0.9_016816m | AT1G30910.1 |  | Molybdenum cofactor sulfurase family protein |
| 1. clementina_scaffold_29:919439-921439 | 0.97626 | 0.000812684 | clementine0.9_024323m | AT5G08180.1 |  | Ribosomal protein L7Ae/L30e/S12e/Gadd45 family protein |
| 1. clementina_scaffold_21:1149547-1151147 | 0.975689 | 0.000904001 | clementine0.9_024879m | AT2G32180.1 | PTAC18 | plastid transcriptionally active 18 |
| 1. clementina_scaffold_27:3114649-3117272 | 0.975247 | 0.000100566 | clementine0.9_024837m | AT4G26740.1 | ATS1 | seed gene 1 |
| 1. clementina_scaffold_31:1964517-1968392 | 0.972974 | 1.29159e-11 | clementine0.9_015023m | AT3G20810.1 |  | 2-oxoglutarate (2OG) and Fe(II)-dependent oxygenase superfamily protein |
| 1. clementina_scaffold_44:123747-124910 | 0.972592 | 0.000312807 | clementine0.9_016737m | AT5G20620.1 | UBQ4 | ubiquitin 4 |
| 1. clementina_scaffold_141:184891-191499 | 0.970332 | 0.000508393 | clementine0.9_030197m | AT3G47570.1 |  | Leucine-rich repeat protein kinase family protein |
| 1. clementina_scaffold_88:181146-183204 | 0.965053 | 0.000183035 | clementine0.9_023543m | AT1G52590.1 |  | Putative thiol-disulphide oxidoreductase DCC |
| 1. clementina_scaffold_4:6809483-6812067 | 0.963008 | 0.000157557 | clementine0.9_020951m | AT1G50590.1 |  | RmlC-like cupins superfamily protein |
| 1. clementina_scaffold_3:8978916-8981975 | 0.961658 | 0.000802975 | clementine0.9_030066m | AT4G15880.1 | ESD4 | Cysteine proteinases superfamily protein |
| 1. clementina_scaffold_54:670168-672193 | 0.959573 | 0.00154291 | clementine0.9_001315m | AT2G01830.2 | WOL | CHASE domain containing histidine kinase protein-receptor de citocinina |
| 1. clementina_scaffold_1:6035520-6046136 | 0.95914 | 0.00116415 | clementine0.9_016545m | AT3G04880.1 | DRT102 | DNA-damage-repair/toleration protein (DRT102) |
| 1. clementina_scaffold_16:2168937-2172129 | 0.957476 | 0.0019987 | clementine0.9_020317m | AT3G10915.2 |  | Reticulon family protein |
| 1. clementina_scaffold_148:226789-227367 | 0.952105 | 0.000832956 | clementine0.9_000642m | AT3G28860.1 | ABCB19 | ATP binding cassette subfamily B19 |
| 1. clementina_scaffold_3:3654747-3658537 | 0.951579 | 0.0003127 | clementine0.9_022663m | AT4G01900.1 | GLB1 | GLNB1 homolog |
| 1. clementina_scaffold_7:5842367-5843238 | 0.950514 | 0.000487184 | clementine0.9_023886m | AT5G49480.1 | CP1 | Ca2+-binding protein 1 |
| 1. clementina_scaffold_3:4911757-4913241 | 0.94161 | 7.53729e-05 | clementine0.9_017738m | AT5G27690.1 |  | Heavy metal transport/detoxification superfamily protein |
| 1. clementina_scaffold_4:6491041-6493113 | 0.941368 | 9.49838e-06 | clementine0.9_007882m | AT3G26300.1 | CYP71B34 | cytochrome P450. family 71. subfamily B. polypeptide 34 |
| 1. clementina_scaffold_22:1400133-1400982 | 0.940176 | 2.41045e-06 | clementine0.9_026731m | AT1G75750.1 | GASA1 | GAST1 protein homolog 1 |
| 1. clementina_scaffold_31:578507-580606 | 0.931072 | 8.36839e-06 | clementine0.9_018923m | AT5G16010.1 |  | 3-oxo-5-alpha-steroid 4-dehydrogenase family protein |
| 1. clementina_scaffold_2:7730712-7739668 | 0.926413 | 6.57023e-06 | clementine0.9_014786m | AT2G35680.1 |  | Phosphotyrosine protein phosphatases superfamily protein |
| 1. clementina_scaffold_3:6040890-6042515 | 0.925683 | 0.00187379 | clementine0.9_016437m | AT4G02730.1 |  | Transducin/WD40 repeat-like superfamily protein |
| 1. clementina_scaffold_7:2111878-2113375 | 0.922731 | 0.00110334 | clementine0.9_014192m | AT2G37400.1 |  | Tetratricopeptide repeat (TPR)-like superfamily protein |
| 1. clementina_scaffold_13:4535806-4539042 | 0.922319 | 0.00224248 | clementine0.9_024784m | AT5G58240.1 | FHIT | FRAGILE HISTIDINE TRIAD |
| 1. clementina_scaffold_33:1268950-1270695 | 0.920715 | 2.41134e-05 | clementine0.9_026485m | AT3G16080.1 |  | Zinc-binding ribosomal protein family protein |
| 1. clementina_scaffold_7:4178251-4178822 | 0.917164 | 0.00180242 | clementine0.9_026646m | AT5G18850.1 |  |  |
| 1. clementina_scaffold_32:2139092-2142949 | 0.912485 | 0.000709432 | clementine0.9_016406m | AT5G23540.1 |  | Mov34/MPN/PAD-1 family protein |
| 1. clementina_scaffold_19:1200295-1202285 | 0.911349 | 0.00269721 | clementine0.9_021754m | AT1G30070.1 |  | SGS domain-containing protein |
| 1. clementina_scaffold_8:2802447-2804119 | 0.908883 | 0.00010284 | clementine0.9_020086m | AT1G56450.1 | PBG1 | 20S proteasome beta subunit G1 |
| 1. clementina_scaffold_7:6315245-6316860 | 0.907239 | 2.66557e-06 | clementine0.9_012665m | AT3G17390.1 | MTO3 | S-adenosylmethionine synthetase family protein |
| 1. clementina_scaffold_4:4131090-4135710 | 0.906887 | 0.00071562 | clementine0.9_007085m | AT2G26200.1 |  | S-adenosyl-L-methionine-dependent methyltransferases superfamily protein |
| 1. clementina_scaffold_21:824111-825670 | 0.904105 | 0.000586167 | clementine0.9_016110m | AT1G05010.1 | EFE | ethylene-forming enzyme |
| 1. clementina_scaffold_46:1584881-1586344 | 0.900411 | 0.000959605 | clementine0.9_026192m | AT4G14320.1 |  | Zinc-binding ribosomal protein family protein |
| 1. clementina_scaffold_128:73540-75681 | 0.896379 | 9.4387e-05 | clementine0.9_011431m | AT5G22060.1 | J2 | DNAJ homologue 2 |
| 1. clementina_scaffold_15:739259-740524 | 0.895714 | 0.0018652 | clementine0.9_016481m | AT3G18420.1 |  | Protein prenylyltransferase superfamily protein |
| 1. clementina_scaffold_136:13005-17911 | 0.89524 | 0.00124198 | clementine0.9_000924m | AT2G23460.1 | XLG1 | extra-large G-protein 1 |
| 1. clementina_scaffold_27:483034-483983 | 0.892245 | 0.00132127 | clementine0.9_022695m | AT3G22840.1 | ELIP1 | Chlorophyll A-B binding family protein |
| 1. clementina_scaffold_2:3501305-3503062 | 0.890312 | 0.000580934 | clementine0.9_019076m | AT3G12750.1 | ZIP1 | zinc transporter 1 precursor |
| 1. clementina_scaffold_2:1735822-1738457 | 0.888093 | 0.000735978 | clementine0.9_005386m | AT4G33070.1 |  | Thiamine pyrophosphate dependent pyruvate decarboxylase family protein |
| 1. clementina_scaffold_20:3893014-3896138 | 0.887261 | 6.12038e-05 | clementine0.9_014749m | AT2G20560.1 |  | DNAJ heat shock family protein |
| 1. clementina_scaffold_146:273968-275921 | 0.886002 | 0.000152788 | clementine0.9_017826m | AT5G23660.1 | MTN3 | homolog of Medicago truncatula MTN3 |
| 1. clementina_scaffold_9:1994003-1995511 | 0.881797 | 0.000558997 | clementine0.9_018554m | AT5G20190.1 |  | Tetratricopeptide repeat (TPR)-like superfamily protein |
| 1. clementina_scaffold_47:1614875-1617472 | 0.878327 | 0.000740688 | clementine0.9_008972m | AT1G71140.1 |  | MATE efflux family protein |
| 1. clementina_scaffold_7:1959200-1962164 | 0.877296 | 0.00210496 | clementine0.9_024034m | AT3G53620.1 | PPa4 | pyrophosphorylase 4 |
| 1. clementina_scaffold_91:401720-406257 | 0.873723 | 0.000963264 | clementine0.9_007502m | AT3G03520.1 | NPC3 | non-specific phospholipase C3 |
| 1. clementina_scaffold_26:2416846-2418539 | 0.872594 | 0.00023986 | clementine0.9_024427m | AT4G38580.1 | FP6 | farnesylated protein 6 |
| 1. clementina_scaffold_3:5446768-5454695 | 0.871768 | 0.000130811 | clementine0.9_030780m | AT2G48020.1 |  | Major facilitator superfamily protein |
| 1. clementina_scaffold_17:3316166-3317412 | 0.867668 | 0.00114759 | clementine0.9_027179m | AT5G41685.1 |  | Mitochondrial outer membrane translocase complex. subunit Tom7 |
| 1. clementina_scaffold_25:2300598-2301680 | 0.866888 | 8.89199e-05 | clementine0.9_022677m | AT2G18050.1 | HIS1-3 | histone H1-3 |
| 1. clementina_scaffold_3:8681092-8685661 | 0.863939 | 0.00126939 | clementine0.9_004419m | AT1G58030.1 | CAT2 | cationic amino acid transporter 2 |
| 1. clementina_scaffold_14:1472925-1474306 | 0.862244 | 0.00174859 | clementine0.9_003800m | AT1G65730.1 | YSL7 | YELLOW STRIPE like 7 |
| 1. clementina_scaffold_19:3459524-3461176 | 0.860952 | 0.0019754 | clementine0.9_021864m | AT4G05000.1 | VPS28-2 | Vacuolar protein sorting-associated protein VPS28 family protein |
| 1. clementina_scaffold_89:868480-869021 | 0.857579 | 0.00149823 | clementine0.9_027273m |  |  |  |
| 1. clementina_scaffold_10:4960174-4961763 | 0.851991 | 8.91866e-05 | clementine0.9_026215m | AT5G08290.1 | YLS8 | mRNA splicing factor. thioredoxin-like U5 snRNP |
| 1. clementina_scaffold_55:436059-437988 | 0.846126 | 0.00208491 | clementine0.9_016626m | AT4G39230.1 |  | NmrA-like negative transcriptional regulator family protein |
| 1. clementina_scaffold_51:71492-74951 | 0.845214 | 0.00139645 | clementine0.9_005986m | AT3G23990.1 | HSP60 | heat shock protein 60 |
| 1. clementina_scaffold_2:7843376-7847391 | 0.844326 | 0.00240064 | clementine0.9_020648m | AT4G17510.1 | UCH3 | ubiquitin C-terminal hydrolase 3 |
| 1. clementina_scaffold_2:7600742-7604856 | 0.843829 | 6.84864e-05 | clementine0.9_018028m | AT4G17420.1 |  | Tryptophan RNA-binding attenuator protein-like |
| 1. clementina_scaffold_25:1685848-1687459 | 0.843735 | 0.00251124 | clementine0.9_033620m | AT1G75250.1 | RL6 | RAD-like 6 |
| 1. clementina_scaffold_58:1366189-1375584 | 0.843134 | 0.000496529 | clementine0.9_035784m | AT4G29060.1 | emb2726 | elongation factor Ts family protein |
| 1. clementina_scaffold_3:1028965-1030310 | 0.84066 | 0.00287074 | clementine0.9_026368m | AT3G61113.1 |  | Ubiquitin related modifier 1 |
| 1. clementina_scaffold_2:4096641-4098365 | 0.839439 | 0.00215061 | clementine0.9_013907m | AT3G12050.1 |  | Aha1 domain-containing protein |
| 1. clementina_scaffold_8:813462-818710 | 0.834017 | 0.00289736 | clementine0.9_004655m | AT2G29630.1 | THIC | thiaminC |
| 1. clementina_scaffold_29:1512169-1513894 | 0.832996 | 0.00101667 | clementine0.9_002374m | AT2G41820.1 |  | Leucine-rich repeat protein kinase family protein |
| 1. clementina_scaffold_80:524036-527038 | 0.829435 | 0.00175745 | clementine0.9_024924m | AT5G38660.2 | APE1 | acclimation of photosynthesis to environment |
| 1. clementina_scaffold_82:349509-351319 | 0.824293 | 0.00271989 | clementine0.9_004490m | AT1G56130.1 |  | Leucine-rich repeat transmembrane protein kinase |
| 1. clementina_scaffold_26:1718992-1721969 | 0.821177 | 7.57377e-07 | clementine0.9_024657m | AT1G50020.1 |  |  |
| 1. clementina_scaffold_34:2294412-2298967 | 0.819743 | 0.000594611 | clementine0.9_003324m | AT1G71820.1 | SEC6 | SEC6 |
| 1. clementina_scaffold_22:2500062-2503949 | 0.816278 | 3.41093e-09 | clementine0.9_012203m | AT1G65910.1 | NAC028 | NAC domain containing protein 28 |
| 1. clementina_scaffold_6:4890038-4891799 | 0.812575 | 0.00179615 | clementine0.9_009496m | AT5G52540.1 |  | Protein of unknown function (DUF819) |
| 1. clementina_scaffold_22:2380547-2384242 | 0.809261 | 2.49584e-06 | clementine0.9_021611m | AT5G42960.1 |  |  |
| 1. clementina_scaffold_9:4211107-4213258 | 0.803457 | 0.00247432 | clementine0.9_014067m | AT1G16080.1 |  |  |
| 1. clementina_scaffold_106:537912-540218 | 0.801842 | 0.00120438 | clementine0.9_010420m |  |  |  |
| 1. clementina_scaffold_27:561768-564777 | 0.799601 | 0.000463557 | clementine0.9_009574m | AT3G22890.1 | APS1 | ATP sulfurylase 1 |
| 1. clementina_scaffold_12:2454751-2456551 | 0.788445 | 0.0028236 | clementine0.9_026699m | AT3G48140.1 |  | B12D protein |
| 1. clementina_scaffold_32:1160590-1165781 | 0.785416 | 0.000909392 | clementine0.9_000537m | AT1G72390.1 |  |  |
| 1. clementina_scaffold_63:1134858-1137280 | 0.784445 | 0.00250046 | clementine0.9_021479m | AT2G25110.1 | SDF2 | stromal cell-derived factor 2-like protein precursor |
| 1. clementina_scaffold_75:1148681-1149216 | 0.783966 | 0.00284387 | clementine0.9_032568m | AT1G66240.1 | ATX1 | homolog of anti-oxidant 1 |
| 1. clementina_scaffold_2:1374411-1378114 | 0.780307 | 0.000201378 | clementine0.9_027358m |  |  |  |
| 1. clementina_scaffold_71:1137593-1143376 | 0.777407 | 2.47112e-06 | clementine0.9_030300m | AT3G24503.1 | ALDH2C4 | aldehyde dehydrogenase 2C4 |
| 1. clementina_scaffold_98:513864-516279 | 0.776703 | 0.00127863 | clementine0.9_007987m | AT4G31500.1 | CYP83B1 | cytochrome P450. family 83. subfamily B. polypeptide 1 |
| 1. clementina_scaffold_1:6620780-6623528 | 0.772712 | 6.35527e-07 | clementine0.9_024343m | AT1G08830.1 | CSD1 | copper/zinc superoxide dismutase 1 |
| 1. clementina_scaffold_8:273431-275144 | 0.77072 | 0.000294896 | clementine0.9_019991m | AT1G07050.1 |  | CCT motif family protein |
| 1. clementina_scaffold_2:4557091-4561401 | 0.768345 | 0.00148 | clementine0.9_007761m | AT5G06560.1 |  | Protein of unknown function. DUF593 |
| 1. clementina_scaffold_8:1219181-1221376 | 0.766666 | 0.00168403 | clementine0.9_017400m | AT3G11400.1 | EIF3G1 | eukaryotic translation initiation factor 3G1 |
| 1. clementina_scaffold_39:1352174-1355553 | 0.765117 | 0.000733834 | clementine0.9_009620m | AT2G33500.2 |  | B-box type zinc finger protein with CCT domain |
| 1. clementina_scaffold_8:2580831-2584699 | 0.763165 | 0.000538748 | clementine0.9_012176m | AT3G07630.1 | ADT2 | arogenate dehydratase 2 |
| 1. clementina_scaffold_8:3006894-3008537 | 0.757536 | 0.00108369 | clementine0.9_031693m | AT5G60640.1 | PDIL1-4 | PDI-like 1-4 |
| 1. clementina_scaffold_12:4587161-4590930 | 0.756535 | 0.00216433 | clementine0.9_019409m | AT5G41960.1 |  |  |
| 1. clementina_scaffold_50:145053-149367 | 0.754871 | 0.00208003 | clementine0.9_013077m | AT5G20540.1 | BRXL4 | BREVIS RADIX-like 4 |
| 1. clementina_scaffold_4:3719929-3722692 | 0.737745 | 0.00289871 | clementine0.9_021970m | AT5G58110.1 |  | chaperone binding;ATPase activators |
| 1. clementina_scaffold_15:3435819-3440890 | 0.73364 | 0.000385427 | clementine0.9_008727m | AT5G63890.1 | HDH | histidinol dehydrogenase |
| 1. clementina_scaffold_67:987832-993477 | 0.728939 | 8.28195e-06 | clementine0.9_004780m | AT1G30900.1 | VSR6 | VACUOLAR SORTING RECEPTOR 6 |
| 1. clementina_scaffold_8:2512532-2515931 | 0.721895 | 6.3265e-05 | clementine0.9_019663m | AT1G07890.1 | APX1 | ascorbate peroxidase 1 |
| 1. clementina_scaffold_20:1771067-1777253 | 0.719579 | 0.00040702 | clementine0.9_009769m | AT1G08490.1 | CPNIFS | chloroplastic NIFS-like cysteine desulfurase |
| 1. clementina_scaffold_18:1781606-1785667 | 0.718882 | 0.000240914 | clementine0.9_019189m | AT5G24650.1 |  | Mitochondrial import inner membrane translocase subunit Tim17/Tim22/Tim23 family protein |
| 1. clementina_scaffold_12:801361-803953 | 0.718142 | 0.000256727 | clementine0.9_020927m | AT2G18110.1 |  | Translation elongation factor EF1B/ribosomal protein S6 family protein |
| 1. clementina_scaffold_1:11281415-11287021 | 0.717608 | 0.000184371 | clementine0.9_005145m | AT4G24620.1 | PGI1 | phosphoglucose isomerase 1 |
| 1. clementina_scaffold_33:2108178-2115946 | 0.716479 | 9.02298e-05 | clementine0.9_016849m | AT1G14520.1 | MIOX1 | myo-inositol oxygenase 1 |
| 1. clementina_scaffold_16:2787210-2790655 | 0.710771 | 0.000234842 | clementine0.9_019031m | AT2G40300.1 | FER4 | ferritin 4 |
| 1. clementina_scaffold_111:270553-272631 | 0.698468 | 0.00268462 | clementine0.9_019770m | AT3G58720.1 |  | RING/U-box superfamily protein |
| 1. clementina_scaffold_59:741772-744221 | 0.697985 | 0.000125015 | clementine0.9_024714m | AT4G13850.1 | GR-RBP2 | glycine-rich RNA-binding protein 2 |
| 1. clementina_scaffold_1:9785612-9788444 | 0.697268 | 0.000802088 | clementine0.9_023159m | AT1G09330.1 |  |  |
| 1. clementina_scaffold_2:4427120-4429098 | 0.68815 | 1.26333e-06 | clementine0.9_021954m | AT3G11940.1 | RPS5A | ribosomal protein 5A |
| 1. clementina_scaffold_82:212899-217300 | 0.688014 | 0.00209932 | clementine0.9_012715m | AT4G32140.1 |  | EamA-like transporter family |
| 1. clementina_scaffold_26:1186578-1189451 | 0.686392 | 0.000347044 | clementine0.9_024004m | AT3G43740.1 |  | Leucine-rich repeat (LRR) family protein |
| 1. clementina_scaffold_19:1536874-1539246 | 0.682264 | 0.000108074 | clementine0.9_025382m | AT1G29850.2 |  | double-stranded DNA-binding family protein |
| 1. clementina_scaffold_62:909870-911843 | 0.681654 | 0.000758647 | clementine0.9_003995m | AT1G65800.1 | RK2 | receptor kinase 2 |
| 1. clementina_scaffold_22:1982657-1985211 | 0.674616 | 0.00135515 | clementine0.9_018453m | AT1G04940.1 | TIC20 | translocon at the inner envelope membrane of chloroplasts 20 |
| 1. clementina_scaffold_4:5173147-5175144 | 0.670537 | 2.51287e-06 | clementine0.9_019565m | AT5G20720.1 | CPN20 | chaperonin 20 |
| 1. clementina_scaffold_15:4590062-4595147 | 0.670075 | 0.00184289 | clementine0.9_012232m | AT2G26560.1 | PLA2A | phospholipase A 2A |
| 1. clementina_scaffold_22:1310107-1314720 | 0.666056 | 2.53538e-05 | clementine0.9_002519m | AT4G24190.1 | SHD | Chaperone protein htpG family protein |
| 1. clementina_scaffold_12:851473-854657 | 0.660885 | 0.00131251 | clementine0.9_008458m | AT4G24830.1 |  | arginosuccinate synthase family |
| 1. clementina_scaffold_3:2749993-2754194 | 0.660457 | 0.000494015 | clementine0.9_021762m | AT4G00850.1 | GIF3 | GRF1-interacting factor 3 |
| 1. clementina_scaffold_20:363386-367048 | 0.657263 | 0.000239534 | clementine0.9_020972m | AT5G19855.1 |  | Chaperonin-like RbcX protein |
| 1. clementina_scaffold_26:483198-488159 | 0.649442 | 0.00133671 | clementine0.9_004187m | AT1G49730.1 |  | Protein kinase superfamily protein |
| 1. clementina_scaffold_39:1199010-1204681 | 0.644459 | 0.000264425 | clementine0.9_008256m | AT3G07530.1 |  |  |
| 1. clementina_scaffold_20:4020352-4024532 | 0.638119 | 0.00287988 | clementine0.9_020296m | AT4G28510.1 | PHB1 | prohibitin 1 |
| 1. clementina_scaffold_10:4901001-4908259 | 0.629384 | 0.00290308 | clementine0.9_020101m | AT3G19910.1 |  | RING/U-box superfamily protein |
| 1. clementina_scaffold_12:4438349-4441859 | 0.626895 | 0.000409234 | clementine0.9_031989m | AT5G42020.1 | BIP2 | Heat shock protein 70 (Hsp 70) family protein |
| 1. clementina_scaffold_15:3802260-3806163 | 0.615283 | 2.09442e-05 | clementine0.9_012331m | AT1G30620.1 | MUR4 | NAD(P)-binding Rossmann-fold superfamily protein |
| 1. clementina_scaffold_2:5353064-5354946 | 0.613264 | 0.000430462 | clementine0.9_025123m | AT3G52590.1 | UBQ1 | ubiquitin extension protein 1 |
| 1. clementina_scaffold_34:721555-725957 | 0.611814 | 0.00182578 | clementine0.9_009202m | AT1G21400.1 |  | Thiamin diphosphate-binding fold (THDP-binding) superfamily protein |
| 1. clementina_scaffold_56:949377-951315 | 0.609608 | 0.00138665 | clementine0.9_023216m | AT1G71000.1 |  | Chaperone DnaJ-domain superfamily protein |
| 1. clementina_scaffold_22:661023-668161 | 0.60457 | 0.00106794 | clementine0.9_002220m | AT5G03650.1 | SBE2.2 | starch branching enzyme 2.2 |
| 1. clementina_scaffold_39:1124328-1125310 | 0.603367 | 0.00138794 | clementine0.9_024157m | AT4G14270.1 |  |  |
| 1. clementina_scaffold_4:4419314-4421015 | 0.602429 | 0.00227636 | clementine0.9_021539m | AT5G21430.1 |  | Chaperone DnaJ-domain superfamily protein |
| 1. clementina_scaffold_16:1341287-1342834 | 0.60149 | 0.00117536 | clementine0.9_025459m | AT3G55280.1 | RPL23AB | ribosomal protein L23AB |
| 1. clementina_scaffold_2:830973-837175 | 0.600638 | 0.000253155 | clementine0.9_007325m | AT5G01220.1 | SQD2 | sulfoquinovosyldiacylglycerol 2 |
| 1. clementina_scaffold_33:1627607-1631467 | 0.600556 | 0.00207305 | clementine0.9_023518m | AT3G16250.1 | NDF4 | NDH-dependent cyclic electron flow 1 |
| 1. clementina_scaffold_8:766093-769004 | 0.599888 | 0.00116523 | clementine0.9_009656m | AT2G29670.1 |  | Tetratricopeptide repeat (TPR)-like superfamily protein |
| 1. clementina_scaffold_32:110999-113264 | 0.595104 | 0.00286212 | clementine0.9_000327m | AT3G21250.2 | MRP6 | multidrug resistance-associated protein 6 |
| 1. clementina_scaffold_6:5188053-5190211 | 0.593781 | 0.00275897 | clementine0.9_017725m | AT1G64090.1 | RTNLB3 | Reticulan like protein B3 |
| 1. clementina_scaffold_12:2463725-2466475 | 0.590723 | 0.00162214 | clementine0.9_022368m | AT3G48100.1 | RR5 | response regulator 5 |
| 1. clementina_scaffold_21:666147-669994 | 0.588197 | 0.00166696 | clementine0.9_010903m | AT1G04980.1 | PDIL2-2 | PDI-like 2-2 |
| 1. clementina_scaffold_44:561097-563586 | 0.587872 | 0.000600871 | clementine0.9_026960m | AT4G23710.1 | VAG2 | vacuolar ATP synthase subunit G2 |
| 1. clementina_scaffold_19:3175106-3181655 | 0.584159 | 0.000557627 | clementine0.9_005936m | AT4G21660.1 |  | proline-rich spliceosome-associated (PSP) family protein |
| 1. clementina_scaffold_10:1438799-1446327 | 0.58258 | 0.000692712 | clementine0.9_000452m | AT1G10760.1 | SEX1 | Pyruvate phosphate dikinase. PEP/pyruvate binding domain |
| 1. clementina_scaffold_7:1957892-1959090 | 0.57929 | 0.00131862 | clementine0.9_022164m | AT3G53620.1 | PPa4 | pyrophosphorylase 4 |
| 1. clementina_scaffold_12:3483063-3486474 | 0.561434 | 5.11979e-05 | clementine0.9_019214m | AT4G23470.1 |  | PLAC8 family protein |
| 1. clementina_scaffold_44:1465179-1472387 | 0.561411 | 0.000946295 | clementine0.9_011543m | AT4G34640.1 | SQS1 | squalene synthase 1 |
| 1. clementina_scaffold_78:28663-31447 | 0.545004 | 0.00107859 | clementine0.9_020903m | AT5G07020.1 |  | proline-rich family protein |
| 1. clementina_scaffold_132:431858-435342 | 0.542846 | 0.00197397 | clementine0.9_007760m | AT5G22620.1 |  | phosphoglycerate/bisphosphoglycerate mutase family protein |
| 1. clementina_scaffold_4:6361073-6366812 | 0.542806 | 0.000101007 | clementine0.9_004530m | AT4G26630.1 |  | DEK domain-containing chromatin associated protein |
| 1. clementina_scaffold_1:9411925-9413581 | 0.539707 | 5.42889e-05 | clementine0.9_026008m | AT1G56300.1 |  | Chaperone DnaJ-domain superfamily protein |
| 1. clementina_scaffold_22:3578652-3585930 | 0.530831 | 0.000131323 | clementine0.9_002996m | AT5G57035.1 |  | U-box domain-containing protein kinase family protein |
| 1. clementina_scaffold_8:4437290-4440552 | 0.514677 | 0.00130364 | clementine0.9_009396m | AT4G19810.1 |  | Glycosyl hydrolase family protein with chitinase insertion domain |
| 1. clementina_scaffold_91:805883-812330 | 0.504877 | 0.00289203 | clementine0.9_010095m | AT4G02600.1 | MLO1 | Seven transmembrane MLO family protein |
| 1. clementina_scaffold_2:5488404-5494432 | 0.496519 | 0.00105562 | clementine0.9_015095m | AT5G03880.1 |  | Thioredoxin family protein |
| 1. clementina_scaffold_1:5144859-5150797 | 0.481089 | 0.000678792 | clementine0.9_014785m | AT1G60680.1 |  | NAD(P)-linked oxidoreductase superfamily protein |
| 1. clementina_scaffold_2:7849228-7852358 | 0.47721 | 0.0016177 | clementine0.9_022021m | AT4G16800.1 |  | ATP-dependent caseinolytic (Clp) protease/crotonase family protein |
| 1. clementina_scaffold_128:99779-107159 | 0.475845 | 0.000983471 | clementine0.9_023124m | AT3G11780.1 |  | MD-2-related lipid recognition domain-containing protein / ML domain-containing protein |
| 1. clementina_scaffold_19:3370440-3376834 | 0.464702 | 0.00032328 | clementine0.9_011859m | AT1G11170.1 |  | Protein of unknown function (DUF707) |
| 1. clementina_scaffold_10:1216313-1219587 | 0.462527 | 0.00159192 | clementine0.9_006161m | AT1G60420.1 |  | DC1 domain-containing protein |
| 1. clementina_scaffold_4:7346111-7348361 | 0.438069 | 0.00179887 | clementine0.9_025680m | AT1G62040.2 | ATG8C | Ubiquitin-like superfamily protein |
| 1. clementina_scaffold_4:4074729-4078171 | 0.434377 | 0.000554757 | clementine0.9_010854m | AT3G01300.1 |  | Protein kinase superfamily protein |
| 1. clementina_scaffold_9:254708-260636 | 0.406528 | 0.000602795 | clementine0.9_016107m | AT5G25560.1 |  | CHY-type/CTCHY-type/RING-type Zinc finger protein |
| 1. clementina_scaffold_12:2825867-2829535 | 0.367192 | 0.00169518 | clementine0.9_010757m | AT1G69850.1 | NRT1:2 | nitrate transporter 1:2 |

*The fold change values (P ≤ 0.001) obtained from of each treated sample compared to Etanol control.

** Identification number of *Citrus clementina* transcripts present in the locus - http://www.phytozome.org/search.php

***Identification number of the *Arabidopsis thaliana* ortholog of up-regulated citrus gene in response to SA treatment (The Arabidopsis Genome Initiative).
