# Supplementary material for: Transcriptional profile of sweet orange in response to chitosan and salicylic acid
Source: BMC Genomics. 2015 Apr 12;16(1):288. doi: 10.1186/s12864-015-1440-5 (PMC4415254; doi:10.1186/s12864-015-1440-5)
Supplement: Additional file 5: Table S4-2. — Differentially expressed genes that were downregulated in SA-treated plants. [file 12864_2015_1440_MOESM5_ESM.docx]

**Table S4-2** Differentially expressed genes that were down-regulated in SA-treated plants.

| **Locus** | **Log2**  **(fold_change)*** | **p_value** | **ID**** | ***Arabidopsis thaliana****** | **Gene** | **Description** |
| --- | --- | --- | --- | --- | --- | --- |
| 1. clementina_scaffold_4:4407359-4411370 | -4.1822 | 0.00 | clementine0.9_027824m | AT5G19530.1 | ACL5 | S-adenosyl-L-methionine-dependent methyltransferases superfamily protein |
| 1. clementina_scaffold_10:3889884-3896933 | -4.04612 | 0.00063184 | clementine0.9_003189m | AT1G68690.1 |  | Protein kinase superfamily protein |
| 1. clementina_scaffold_9:2625862-2626416 | -3.96774 | 0.00 | clementine0.9_026873m |  |  |  |
| 1. clementina_scaffold_43:899931-905680 | -3.85815 | 0.000370373 | clementine0.9_007011m | AT5G46250.1 |  | RNA-binding protein |
| 1. clementina_scaffold_6:5912011-5914737 | -3.80733 | 0.0012992 | clementine0.9_000064m | AT5G53460.1 | GLT1 | NADH-dependent glutamate synthase 1 |
| 1. clementina_scaffold_3:2209186-2210086 | -3.72738 | 0.00168726 | clementine0.9_004590m | AT1G54115.1 | CCX4 | cation calcium exchanger 4 |
| 1. clementina_scaffold_16:2087637-2088678 | -3.70266 | 2.1579e-11 | clementine0.9_020714m | AT3G56360.1 |  |  |
| 1. clementina_scaffold_43:110865-111715 | -3.6618 | 0.00206964 | clementine0.9_000876m | AT3G14470.1 |  | NB-ARC domain-containing disease resistance protein |
| 1. clementina_scaffold_11:2449597-2450996 | -3.61624 | 0.000138221 | clementine0.9_035576m | AT5G17680.1 |  | disease resistance protein (TIR-NBS-LRR class). putative |
| 1. clementina_scaffold_3:4660419-4662918 | -3.50343 | 0.000243362 | clementine0.9_026114m | AT2G47640.2 |  | Small nuclear ribonucleoprotein family protein |
| 1. clementina_scaffold_40:396522-400301 | -3.37707 | 0.000567118 | clementine0.9_000362m | AT5G56890.1 |  | Protein kinase superfamily protein |
| 1. clementina_scaffold_15:3932666-3938737 | -3.37602 | 4.54498e-08 | clementine0.9_000078m | AT2G35630.1 | MOR1 | ARM repeat superfamily protein |
| 1. clementina_scaffold_63:892677-897044 | -3.36125 | 0.000242943 | clementine0.9_008184m | AT4G31780.1 | MGD1 | monogalactosyl diacylglycerol synthase 1 |
| 1. clementina_scaffold_15:3927433-3929613 | -3.35305 | 0.000121016 | clementine0.9_000078m | AT2G35630.1 | MOR1 | ARM repeat superfamily protein |
| 1. clementina_scaffold_1:10910939-10912099 | -3.25048 | 7.64414e-07 | clementine0.9_000019m | AT1G70320.1 | UPL2 | ubiquitin-protein ligase 2 |
| 1. clementina_scaffold_9:3362234-3366094 | -3.11213 | 0.000563971 | clementine0.9_001692m | AT4G27190.1 |  | NB-ARC domain-containing disease resistance protein |
| 1. clementina_scaffold_26:1755193-1758551 | -3.08693 | 5.96141e-05 | clementine0.9_000034m | AT1G50030.1 | TOR | target of rapamycin |
| 1. clementina_scaffold_88:581989-582568 | -3.03727 | 0.000333394 | clementine0.9_030049m | AT5G63020.1 |  | Disease resistance protein (CC-NBS-LRR class) family |
| 1. clementina_scaffold_3:9031601-9033958 | -3.00275 | 0.00181673 | clementine0.9_000933m | AT5G19820.1 | emb2734 | ARM repeat superfamily protein |
| 1. clementina_scaffold_25:465457-466856 | -3.00107 | 3.94997e-05 | clementine0.9_000098m | AT3G50370.1 |  |  |
| 1. clementina_scaffold_9:1633512-1636520 | -2.97029 | 0.00281907 | clementine0.9_011286m | AT1G10970.1 | ZIP4 | zinc transporter 4 precursor |
| 1. clementina_scaffold_2:2707555-2711922 | -2.95865 | 2.12097e-05 | clementine0.9_000615m | AT5G04930.1 | ALA1 | aminophospholipid ATPase 1 |
| 1. clementina_scaffold_1:12490910-12495382 | -2.94202 | 1.58695e-07 | clementine0.9_001888m | AT1G77760.1 | NIA1 | nitrate reductase 1 |
| 1. clementina_scaffold_38:1087958-1094293 | -2.9333 | 0.00228788 | clementine0.9_029560m | AT3G14460.1 |  | LRR and NB-ARC domains-containing disease resistance protein |
| 1. clementina_scaffold_35:1769194-1771370 | -2.93167 | 0.000988415 | clementine0.9_032510m | AT1G20960.1 | emb1507 | U5 small nuclear ribonucleoprotein helicase. putative |
| 1. clementina_scaffold_94:117401-119842 | -2.90868 | 0.00277305 | clementine0.9_000177m | AT4G00990.1 |  | Transcription factor jumonji (jmjC) domain-containing protein |
| 1. clementina_scaffold_1:6209640-6214471 | -2.88156 | 0.00124998 | clementine0.9_000227m | AT5G27970.1 |  | ARM repeat superfamily protein |
| 1. clementina_scaffold_109:152473-154152 | -2.85023 | 6.96602e-05 | clementine0.9_003825m | AT5G24320.2 |  | Transducin/WD40 repeat-like superfamily protein |
| 1. clementina_scaffold_95:2545-4814 | -2.84206 | 0.000907709 | clementine0.9_026758m | AT3G61110.1 | RS27A | ribosomal protein S27 |
| 1. clementina_scaffold_53:331536-336039 | -2.8373 | 0.000406967 | clementine0.9_002091m | AT1G69360.1 |  | Plant protein of unknown function (DUF863) |
| 1. clementina_scaffold_6:6632351-6635435 | -2.82175 | 2.33687e-05 | clementine0.9_000491m | AT1G79280.1 | NUA | nuclear pore anchor |
| 1. clementina_scaffold_1:12697129-12698714 | -2.80883 | 5.25633e-11 | clementine0.9_012258m | AT5G13930.1 | TT4 | Chalcone and stilbene synthase family protein |
| 1. clementina_scaffold_31:1187744-1189997 | -2.79334 | 0.000782686 | clementine0.9_000102m | AT3G07160.1 | GSL10 | glucan synthase-like 10 |
| 1. clementina_scaffold_54:200358-202078 | -2.79315 | 0.00173548 | clementine0.9_029676m | AT5G04290.1 | KTF1 | kow domain-containing transcription factor 1 |
| 1. clementina_scaffold_1:9642116-9647912 | -2.78762 | 1.19904e-14 | clementine0.9_032351m | AT4G27190.1 |  | NB-ARC domain-containing disease resistance protein |
| 1. clementina_scaffold_67:428746-431042 | -2.77463 | 0.000111655 | clementine0.9_000605m | AT5G03360.1 |  | DC1 domain-containing protein |
| 1. clementina_scaffold_106:15743-16440 | -2.7741 | 0.000884247 | clementine0.9_000267m | AT4G26090.1 | RPS2 | NB-ARC domain-containing disease resistance protein |
| 1. clementina_scaffold_10:3664984-3666772 | -2.75616 | 0.00206786 | clementine0.9_000265m | AT1G68890.1 |  | magnesium ion binding;thiamin pyrophosphate binding;hydro-lyases;catalytics;2-succinyl-5-enolpyruvyl-6-hydroxy-3-cyclohexene-1-carboxylic-acid synthases |
| 1. clementina_scaffold_1:10041246-10042888 | -2.71783 | 0.000545059 | clementine0.9_001620m | AT5G43130.1 | TAF4 | TBP-associated factor 4 |
| 1. clementina_scaffold_81:678864-679402 | -2.71358 | 0.00245691 | clementine0.9_034047m | AT1G06950.1 | TIC110 | translocon at the inner envelope membrane of chloroplasts 110 |
| 1. clementina_scaffold_7:4217733-4218335 | -2.70946 | 0.000534212 | clementine0.9_002727m | AT5G18830.3 | SPL7 | squamosa promoter binding protein-like 7 |
| 1. clementina_scaffold_87:664735-666083 | -2.69834 | 0.000134479 | clementine0.9_001317m | AT2G43160.1 |  | ENTH/VHS family protein |
| 1. clementina_scaffold_44:1383170-1384613 | -2.63367 | 0.000225713 | clementine0.9_009112m | AT1G12700.1 |  | ATP binding;nucleic acid binding;helicases |
| 1. clementina_scaffold_3:6917301-6918867 | -2.63168 | 0.00153364 | clementine0.9_003668m | AT1G06150.1 | EMB1444 | basic helix-loop-helix (bHLH) DNA-binding superfamily protein |
| 1. clementina_scaffold_9:4884157-4888813 | -2.62627 | 0.000819318 | clementine0.9_001044m | AT3G21180.1 | ACA9 | autoinhibited Ca(2+)-ATPase 9 |
| 1. clementina_scaffold_3:4000015-4001877 | -2.62426 | 0.00206791 | clementine0.9_015353m | AT2G16760.1 |  | Calcium-dependent phosphotriesterase superfamily protein |
| 1. clementina_scaffold_4:5165216-5168210 | -2.60661 | 0.00198966 | clementine0.9_000850m | AT1G19220.1 | ARF19 | auxin response factor 19 |
| 1. clementina_scaffold_16:1597050-1600228 | -2.59509 | 2.47965e-09 | clementine0.9_004610m | AT5G02500.1 | HSC70-1 | heat shock cognate protein 70-1 |
| 1. clementina_scaffold_49:1151357-1153310 | -2.59015 | 2.33627e-05 | clementine0.9_001985m | AT2G04270.2 | RNEE/G | RNAse E/G-like |
| 1. clementina_scaffold_90:419684-421045 | -2.58603 | 0.000516387 | clementine0.9_002268m | AT4G39530.1 |  | Tetratricopeptide repeat (TPR)-like superfamily protein |
| 1. clementina_scaffold_3:2475469-2477824 | -2.58119 | 0.000299667 | clementine0.9_000030m | AT2G46560.1 |  | transducin family protein / WD-40 repeat family protein |
| 1. clementina_scaffold_1:12437227-12441283 | -2.56969 | 4.17444e-13 | clementine0.9_000385m | AT1G77800.1 |  | PHD finger family protein |
| 1. clementina_scaffold_24:3529235-3530759 | -2.55746 | 0.00234275 | clementine0.9_032089m | AT3G14470.1 |  | NB-ARC domain-containing disease resistance protein |
| 1. clementina_scaffold_58:1064503-1065280 | -2.55746 | 0.00234275 | clementine0.9_001474m | AT2G20010.2 |  | Protein of unknown function (DUF810) |
| 1. clementina_scaffold_8:2427221-2428691 | -2.55596 | 9.72364e-06 | clementine0.9_001522m | AT5G58160.1 |  | actin binding |
| 1. clementina_scaffold_9:192744-196382 | -2.55094 | 0.00130433 | clementine0.9_000085m | AT2G25660.1 | emb2410 | embryo defective 2410 |
| 1. clementina_scaffold_7:55777-57353 | -2.54511 | 0.00128086 | clementine0.9_003606m | AT2G37840.1 |  | Protein kinase superfamily protein |
| 1. clementina_scaffold_3:2210398-2211639 | -2.53364 | 0.00259805 | clementine0.9_017684m | AT1G55230.1 |  | Family of unknown function (DUF716) |
| 1. clementina_scaffold_2:7503350-7504993 | -2.52482 | 0.000412832 | clementine0.9_000061m | AT4G17330.1 | G2484-1 | G2484-1 protein |
| 1. clementina_scaffold_3:8764417-8766180 | -2.52429 | 0.000712042 | clementine0.9_002507m | AT5G35910.1 |  | Polynucleotidyl transferase. ribonuclease H fold protein with HRDC domain |
| 1. clementina_scaffold_15:3083929-3088461 | -2.52221 | 1.41128e-05 | clementine0.9_031858m | AT2G34660.1 | MRP2 | multidrug resistance-associated protein 2 |
| 1. clementina_scaffold_7:5754551-5756113 | -2.50855 | 0.000225181 | clementine0.9_000839m | AT2G18790.1 | PHYB | phytochrome B |
| 1. clementina_scaffold_2:4702985-4705470 | -2.50822 | 0.00153009 | clementine0.9_003815m | AT5G22030.1 | UBP8 | ubiquitin-specific protease 8 |
| 1. clementina_scaffold_6:2527750-2527992 | -2.48375 | 0.00 | clementine0.9_016984m | AT5G23240.1 |  | DNAJ heat shock N-terminal domain-containing protein |
| 1. clementina_scaffold_12:3423125-3423421 | -2.4699 | 0.000157425 | clementine0.9_023740m | AT3G46780.1 | PTAC16 | plastid transcriptionally active 16 |
| 1. clementina_scaffold_31:279101-280893 | -2.44343 | 3.0909e-05 | clementine0.9_000071m | AT1G55350.1 | DEK1 | calpain-type cysteine protease family |
| 1. clementina_scaffold_118:251772-253895 | -2.44301 | 1.06717e-05 | clementine0.9_001548m | AT3G14470.1 |  | NB-ARC domain-containing disease resistance protein |
| 1. clementina_scaffold_12:1254617-1257438 | -2.41716 | 3.24964e-05 | clementine0.9_016789m | AT5G63160.1 | BT1 | BTB and TAZ domain protein 1 |
| 1. clementina_scaffold_3:5899834-5900394 | -2.4161 | 8.95909e-05 | clementine0.9_029132m | AT1G03160.1 | FZL | FZO-like |
| 1. clementina_scaffold_84:814571-817348 | -2.41009 | 7.86666e-06 | clementine0.9_002227m | AT5G61960.1 | ML1 | MEI2-like protein 1 |
| 1. clementina_scaffold_12:1886506-1887614 | -2.40828 | 1.00549e-06 | clementine0.9_001035m | AT3G48200.1 |  |  |
| 1. clementina_scaffold_74:980573-985464 | -2.40758 | 0.00238731 | clementine0.9_002684m | AT2G24030.1 |  | zinc ion binding;nucleic acid binding |
| 1. clementina_scaffold_151:43680-49448 | -2.40665 | 0.00196327 | clementine0.9_013268m | AT3G60070.1 |  | Major facilitator superfamily protein |
| 1. clementina_scaffold_22:1216723-1217744 | -2.39616 | 0.00195248 | clementine0.9_000638m | AT1G27940.1 | PGP13 | P-glycoprotein 13 |
| 1. clementina_scaffold_12:537778-540123 | -2.39029 | 0.00159813 | clementine0.9_000013m | AT4G17140.3 |  | pleckstrin homology (PH) domain-containing protein |
| 1. clementina_scaffold_59:1216058-1220093 | -2.38974 | 0.000415187 | clementine0.9_000177m | AT4G00990.1 |  | Transcription factor jumonji (jmjC) domain-containing protein |
| 1. clementina_scaffold_25:464697-465407 | -2.38753 | 0.00273201 | clementine0.9_011434m | AT3G06540.1 | REP | Rab escort protein |
| 1. clementina_scaffold_34:1416903-1417769 | -2.38753 | 0.00273201 | clementine0.9_005384m | AT1G43245.1 |  | SET domain-containing protein |
| 1. clementina_scaffold_1:6590011-6592789 | -2.37236 | 0.00181087 | clementine0.9_016542m | AT3G05010.1 |  | Protein of unknown function. transmembrane-40 |
| 1. clementina_scaffold_2:3688997-3693686 | -2.36845 | 0.00171385 | clementine0.9_000377m | AT4G16340.1 | SPK1 | guanyl-nucleotide exchange factors;GTPase binding;GTP binding |
| 1. clementina_scaffold_1:12436537-12437082 | -2.36611 | 7.98085e-05 | clementine0.9_000385m | AT1G77800.1 |  | PHD finger family protein |
| 1. clementina_scaffold_41:203362-204330 | -2.36548 | 6.83518e-05 | clementine0.9_035252m | AT2G30480.3 |  |  |
| 1. clementina_scaffold_3:8448214-8449125 | -2.36305 | 0.00172622 | clementine0.9_021402m | AT4G13830.2 | J20 | DNAJ-like 20 |
| 1. clementina_scaffold_12:5010441-5011672 | -2.35951 | 0.000587916 | clementine0.9_000742m | AT4G38120.1 |  | ARM repeat superfamily protein |
| 1. clementina_scaffold_3:6213840-6218500 | -2.35372 | 7.50511e-14 | clementine0.9_000779m | AT1G03080.1 |  | kinase interacting (KIP1-like) family protein |
| 1. clementina_scaffold_53:1136996-1138448 | -2.30303 | 0.000493669 | clementine0.9_001602m | AT3G47950.1 | HA4 | H(+)-ATPase 4 |
| 1. clementina_scaffold_4:3438197-3440748 | -2.28759 | 0.00143289 | clementine0.9_000984m | AT5G20730.2 | NPH4 | Transcriptional factor B3 family protein / auxin-responsive factor AUX/IAA-related |
| 1. clementina_scaffold_44:1063349-1066400 | -2.27051 | 7.73827e-08 | clementine0.9_001564m | AT2G21300.1 |  | ATP binding microtubule motor family protein |
| 1. clementina_scaffold_44:178721-183825 | -2.26909 | 0.00044241 | clementine0.9_006636m | AT2G37150.3 |  | RING/U-box superfamily protein |
| 1. clementina_scaffold_1:6145722-6147452 | -2.24035 | 0.00113824 | clementine0.9_001582m | AT1G54490.1 | XRN4 | exoribonuclease 4 |
| 1. clementina_scaffold_1:10539878-10542580 | -2.23397 | 0.00216029 | clementine0.9_003022m | AT1G58350.1 | ZW18 | Putative serine esterase family protein |
| 1. clementina_scaffold_17:2851547-2852284 | -2.23328 | 2.15107e-05 | clementine0.9_025703m | AT2G38870.1 |  | Serine protease inhibitor. potato inhibitor I-type family protein |
| 1. clementina_scaffold_3:1595153-1598307 | -2.23273 | 0.000234545 | clementine0.9_000049m | AT2G46020.2 | BRM | transcription regulatory protein SNF2. putative |
| 1. clementina_scaffold_120:352501-356408 | -2.22816 | 0.00207444 | clementine0.9_035024m | AT1G64790.1 | ILA | ILITYHIA |
| 1. clementina_scaffold_26:2365388-2368214 | -2.22622 | 0.000232389 |  |  |  |  |
| 1. clementina_scaffold_2:3543590-3545248 | -2.22229 | 8.17909e-06 | clementine0.9_010708m | AT3G22142.1 |  | Bifunctional inhibitor/lipid-transfer protein/seed storage 2S albumin superfamily protein |
| 1. clementina_scaffold_17:2945581-2946371 | -2.20771 | 4.66294e-14 | clementine0.9_025727m | AT2G38870.1 |  | Serine protease inhibitor. potato inhibitor I-type family protein |
| 1. clementina_scaffold_29:2196733-2197370 | -2.20752 | 0.000894825 | clementine0.9_003783m | AT1G18670.1 | IBS1 | Protein kinase superfamily protein |
| 1. clementina_scaffold_88:137298-142216 | -2.20647 | 2.49459e-06 | clementine0.9_031894m | AT4G34050.1 | CCoAOMT1 | S-adenosyl-L-methionine-dependent methyltransferases superfamily protein |
| 1. clementina_scaffold_20:3935354-3940843 | -2.20454 | 3.44156e-06 | clementine0.9_000180m | AT5G44180.1 |  | Homeodomain-like transcriptional regulator |
| 1. clementina_scaffold_108:7138-7879 | -2.20138 | 0.000524165 | clementine0.9_001869m | AT5G63020.1 |  | Disease resistance protein (CC-NBS-LRR class) family |
| 1. clementina_scaffold_35:1325902-1327256 | -2.20024 | 9.07523e-07 | clementine0.9_008514m | AT3G02750.1 |  | Protein phosphatase 2C family protein |
| 1. clementina_scaffold_64:189858-195745 | -2.19284 | 0.0003218 | clementine0.9_001787m | AT5G54730.1 | G18F | homolog of yeast autophagy 18 (ATG18) F |
| 1. clementina_scaffold_9:4081225-4089341 | -2.1913 | 1.66763e-06 | clementine0.9_021480m | AT1G80940.1 |  |  |
| 1. clementina_scaffold_10:4743628-4747162 | -2.18463 | 0.00290351 | clementine0.9_000781m | AT3G19960.1 | ATM1 | myosin 1 |
| 1. clementina_scaffold_54:568766-572113 | -2.17541 | 9.36041e-05 | clementine0.9_029099m | AT1G24706.2 | THO2 | THO2 |
| 1. clementina_scaffold_49:486436-487828 | -2.17413 | 0.000136441 | clementine0.9_030591m | AT4G27190.1 |  | NB-ARC domain-containing disease resistance protein |
| 1. clementina_scaffold_65:1364157-1369359 | -2.16262 | 0.00184021 | clementine0.9_030260m | AT1G32750.1 | HAF01 | HAC13 protein (HAC13) |
| 1. clementina_scaffold_3:8222687-8227259 | -2.16053 | 0.00195201 | clementine0.9_000075m | AT3G24870.1 |  | Helicase/SANT-associated. DNA binding protein |
| 1. clementina_scaffold_26:2702778-2704071 | -2.14242 | 0.00206731 | clementine0.9_001439m | AT5G58140.1 | PHOT2 | phototropin 2 |
| 1. clementina_scaffold_139:117371-120365 | -2.12776 | 0.000576753 | clementine0.9_000974m | AT1G10170.1 | NFXL1 | NF-X-like 1 |
| 1. clementina_scaffold_26:2938264-2939687 | -2.12494 | 3.92101e-08 | clementine0.9_015646m | AT3G12500.1 | HCHIB | basic chitinase |
| 1. clementina_scaffold_15:3088652-3093526 | -2.12309 | 1.82507e-06 | clementine0.9_031858m | AT2G34660.1 | MRP2 | multidrug resistance-associated protein 2 |
| 1. clementina_scaffold_10:988652-989576 | -2.11952 | 0.0025512 | clementine0.9_002470m | AT1G23870.1 | TPS9 | trehalose-phosphatase/synthase 9 |
| 1. clementina_scaffold_10:405692-408757 | -2.11751 | 0.000559316 | clementine0.9_017290m | AT1G10350.1 |  | DNAJ heat shock family protein |
| 1. clementina_scaffold_27:15001-15828 | -2.11 | 0.00248637 | clementine0.9_000264m | AT4G14920.1 |  | Acyl-CoA N-acyltransferase with RING/FYVE/PHD-type zinc finger protein |
| 1. clementina_scaffold_16:266859-267961 | -2.08861 | 0.000386324 | clementine0.9_006578m | AT1G28060.1 |  | Pre-mRNA-splicing factor 3 |
| 1. clementina_scaffold_3:7286573-7289057 | -2.0861 | 6.40733e-07 | clementine0.9_011122m | AT4G28550.1 |  | Ypt/Rab-GAP domain of gyp1p superfamily protein |
| 1. clementina_scaffold_125:200971-202948 | -2.07674 | 7.97127e-05 | clementine0.9_000525m | AT4G39850.1 | PXA1 | peroxisomal ABC transporter 1 |
| 1. clementina_scaffold_34:257038-257836 | -2.07443 | 1.12213e-07 | clementine0.9_006038m | AT1G21700.1 | SWI3C | SWITCH/sucrose nonfermenting 3C |
| 1. clementina_scaffold_11:3968958-3973325 | -2.07059 | 2.03277e-07 | clementine0.9_011903m | AT2G39130.1 |  | Transmembrane amino acid transporter family protein |
| 1. clementina_scaffold_10:5797964-5798653 | -2.0479 | 3.52593e-07 | clementine0.9_004805m | AT5G06740.1 |  | Concanavalin A-like lectin protein kinase family protein |
| 1. clementina_scaffold_1:10249069-10257093 | -2.04399 | 1.00612e-09 | clementine0.9_001347m | AT1G10130.1 | ECA3 | endoplasmic reticulum-type calcium-transporting ATPase 3 |
| 1. clementina_scaffold_37:1754063-1755464 | -2.04256 | 2.41711e-06 | clementine0.9_030382m | AT3G14470.1 |  | NB-ARC domain-containing disease resistance protein |
| 1. clementina_scaffold_3:7763507-7765001 | -2.02802 | 0.00248535 |  |  |  |  |
| 1. clementina_scaffold_37:1986545-1987481 | -2.02463 | 0.00259031 | clementine0.9_031140m |  |  |  |
| 1. clementina_scaffold_29:927548-928808 | -2.02461 | 0.00270344 | clementine0.9_010733m | AT1G73980.1 |  | Phosphoribulokinase / Uridine kinase family |
| 1. clementina_scaffold_6:6578395-6580243 | -2.01835 | 0.00135241 | clementine0.9_002500m | AT1G79350.1 | EMB1135 | RING/FYVE/PHD zinc finger superfamily protein |
| 1. clementina_scaffold_17:3821686-3825158 | -2.01711 | 0.000105268 | clementine0.9_028474m | AT1G78770.1 | APC6 | anaphase promoting complex 6 |
| 1. clementina_scaffold_4:3942377-3944317 | -2.01338 | 0.000470208 | clementine0.9_001097m | AT5G20280.1 | SPS1F | sucrose phosphate synthase 1F |
| 1. clementina_scaffold_8:45154-47634 | -2.01233 | 6.4467e-06 | clementine0.9_000170m | AT1G16710.1 | HAC12 | histone acetyltransferase of the CBP family 12 |
| 1. clementina_scaffold_2:3657512-3658951 | -2.01034 | 0.000211881 | clementine0.9_000014m | AT5G24740.1 |  | Protein of unknown function (DUF1162) |
| 1. clementina_scaffold_6:6608340-6610336 | -2.00989 | 0.000684168 | clementine0.9_011243m | AT3G12710.1 |  | DNA glycosylase superfamily protein |
| 1. clementina_scaffold_16:653579-654766 | -1.99894 | 0.00134197 | clementine0.9_000475m | AT5G02950.1 |  | Tudor/PWWP/MBT superfamily protein |
| 1. clementina_scaffold_79:369632-372727 | -1.98815 | 5.44853e-12 | clementine0.9_016289m | AT1G19150.1 | LHCA6 | photosystem I light harvesting complex gene 6 |
| 1. clementina_scaffold_33:1113160-1118563 | -1.98742 | 0.000462202 | clementine0.9_003379m | AT1G52320.2 |  |  |
| 1. clementina_scaffold_31:530059-531397 | -1.98665 | 1.88471e-09 | clementine0.9_014881m | AT1G15670.1 |  | Galactose oxidase/kelch repeat superfamily protein |
| 1. clementina_scaffold_118:228063-230178 | -1.97734 | 0.00067194 | clementine0.9_028955m | AT3G14470.1 |  | NB-ARC domain-containing disease resistance protein |
| 1. clementina_scaffold_33:1961840-1962796 | -1.96999 | 9.10676e-05 | clementine0.9_022514m | AT5G56550.1 | OXS3 | oxidative stress 3 |
| 1. clementina_scaffold_18:1493673-1494518 | -1.9667 | 0.000481404 | clementine0.9_004607m | AT5G54090.1 |  | DNA mismatch repair protein MutS. type 2 |
| 1. clementina_scaffold_27:913635-913979 | -1.95542 | 0.001038 | clementine0.9_015542m | AT5G09690.2 | MGT7 | magnesium transporter 7 |
| 1. clementina_scaffold_26:2597607-2600875 | -1.94308 | 0.00117444 | clementine0.9_001971m | AT2G16950.2 | TRN1 | transportin 1 |
| 1. clementina_scaffold_7:2904086-2905721 | -1.94079 | 0.000186934 | clementine0.9_034406m | AT4G27190.1 |  | NB-ARC domain-containing disease resistance protein |
| 1. clementina_scaffold_54:1051710-1055091 | -1.9365 | 0.00128672 | clementine0.9_004537m | AT2G01980.1 | SOS1 | sodium proton exchanger. putative (NHX7) (SOS1) |
| 1. clementina_scaffold_22:1348024-1348866 | -1.90481 | 9.13714e-12 | clementine0.9_023745m | AT4G36040.1 |  | Chaperone DnaJ-domain superfamily protein |
| 1. clementina_scaffold_18:3014364-3018403 | -1.89288 | 3.14784e-10 | clementine0.9_001060m | AT1G48410.1 | ago/01 | Stabilizer of iron transporter SufD / Polynucleotidyl transferase |
| 1. clementina_scaffold_3:8009373-8013876 | -1.89079 | 1.24345e-14 | clementine0.9_000552m | AT3G24715.1 |  | Protein kinase superfamily protein with octicosapeptide/Phox/Bem1p domain |
| 1. clementina_scaffold_10:1837550-1839712 | -1.89029 | 0.00115423 | clementine0.9_009938m | AT5G14420.1 | RGLG2 | RING domain ligase2 |
| 1. clementina_scaffold_1:8349566-8350092 | -1.88599 | 0.00174016 | clementine0.9_021584m | AT3G14470.1 |  | NB-ARC domain-containing disease resistance protein |
| 1. clementina_scaffold_108:299559-300406 | -1.88553 | 1.00669e-05 | clementine0.9_029016m | AT4G10780.1 |  | LRR and NB-ARC domains-containing disease resistance protein |
| 1. clementina_scaffold_10:4380153-4382142 | -1.88308 | 0.000362581 | clementine0.9_005771m | AT3G25800.1 | PP2AA2 | protein phosphatase 2A subunit A2 |
| 1. clementina_scaffold_16:3090496-3091865 | -1.87939 | 0.000232533 | clementine0.9_000817m | AT2G40130.2 |  | Double Clp-N motif-containing P-loop nucleoside triphosphate hydrolases superfamily protein |
| 1. clementina_scaffold_96:349485-352038 | -1.87554 | 2.13236e-05 | clementine0.9_004781m | AT5G17680.1 |  | disease resistance protein (TIR-NBS-LRR class). putative |
| 1. clementina_scaffold_12:1984243-1987167 | -1.87271 | 6.04949e-05 | clementine0.9_002445m | AT4G32180.2 | PANK2 | pantothenate kinase 2 |
| 1. clementina_scaffold_2:4335620-4336837 | -1.87025 | 0.00107438 | clementine0.9_014053m | AT2G44130.1 |  | Galactose oxidase/kelch repeat superfamily protein |
| 1. clementina_scaffold_47:1547172-1550286 | -1.86133 | 0.00154285 | clementine0.9_010596m | AT1G36730.1 |  | Translation initiation factor IF2/IF5 |
| 1. clementina_scaffold_99:643359-645493 | -1.85929 | 0.000438682 | clementine0.9_010717m | AT5G54590.2 | CRLK1 | Protein kinase superfamily protein |
| 1. clementina_scaffold_29:1563828-1568241 | -1.85724 | 4.91749e-05 | clementine0.9_028323m | AT5G61040.1 |  |  |
| 1. clementina_scaffold_47:808234-808885 | -1.84851 | 0.00212158 | clementine0.9_015549m | AT5G37930.1 |  | Protein with RING/U-box and TRAF-like domains |
| 1. clementina_scaffold_33:516709-519453 | -1.84744 | 0.00109214 | clementine0.9_003361m | AT1G80000.1 |  | CASC3/Barentsz eIF4AIII binding |
| 1. clementina_scaffold_13:2801527-2803827 | -1.84342 | 0.000778374 | clementine0.9_000279m | AT3G57300.1 | INO80 | INO80 ortholog |
| 1. clementina_scaffold_20:119760-120004 | -1.8396 | 0.000269202 | clementine0.9_001658m | AT3G58650.1 |  |  |
| 1. clementina_scaffold_16:4150483-4155334 | -1.8296 | 2.43274e-09 | clementine0.9_004044m | AT5G13820.1 | TBP1 | telomeric DNA binding protein 1 |
| 1. clementina_scaffold_42:327080-328924 | -1.82906 | 0.000942684 | clementine0.9_008123m | AT5G03555.1 |  | permease. cytosine/purines. uracil. thiamine. allantoin family protein |
| 1. clementina_scaffold_20:731951-733916 | -1.82811 | 1.23412e-07 | clementine0.9_034961m | AT2G15080.1 | RLP19 | receptor like protein 19 |
| 1. clementina_scaffold_6:270484-277860 | -1.82129 | 1.22362e-08 | clementine0.9_001336m | AT2G41790.1 |  | Insulinase (Peptidase family M16) family protein |
| 1. clementina_scaffold_32:159459-160316 | -1.82049 | 0.000842036 | clementine0.9_028119m | AT1G55190.1 | PRA7 | PRA1 (Prenylated rab acceptor) family protein |
| 1. clementina_scaffold_33:12184-15759 | -1.81761 | 8.9073e-07 | clementine0.9_000349m | AT1G15520.1 | PDR12 | pleiotropic drug resistance 12 |
| 1. clementina_scaffold_4:504363-505846 | -1.81722 | 4.22963e-05 | clementine0.9_028030m | AT3G14470.1 |  | NB-ARC domain-containing disease resistance protein |
| 1. clementina_scaffold_49:986536-988375 | -1.81541 | 2.13286e-05 | clementine0.9_035601m | AT2G04160.1 | AIR3 | Subtilisin-like serine endopeptidase family protein |
| 1. clementina_scaffold_9:1331631-1339840 | -1.81503 | 0.000718037 | clementine0.9_025315m |  |  |  |
| 1. clementina_scaffold_74:67251-78045 | -1.81344 | 6.47594e-10 | clementine0.9_002280m | AT3G10550.1 | MTM1 | Myotubularin-like phosphatases II superfamily |
| 1. clementina_scaffold_8:3858811-3860359 | -1.81266 | 1.56682e-05 | clementine0.9_017203m | AT5G22920.1 |  | CHY-type/CTCHY-type/RING-type Zinc finger protein |
| 1. clementina_scaffold_116:457558-459040 | -1.80871 | 0.000344308 | clementine0.9_004947m | AT2G31820.1 |  | Ankyrin repeat family protein |
| 1. clementina_scaffold_21:523160-524220 | -1.80871 | 5.07343e-11 | clementine0.9_026022m | AT2G05540.1 |  | Glycine-rich protein family |
| 1. clementina_scaffold_47:558717-560595 | -1.80762 | 6.33193e-05 | clementine0.9_001491m | AT2G40840.1 | DPE2 | disproportionating enzyme 2 |
| 1. clementina_scaffold_39:1111533-1113257 | -1.80648 | 0.000613054 | clementine0.9_003180m | AT1G17690.1 | NOF1 |  |
| 1. clementina_scaffold_1:8761409-8768759 | -1.79769e+308 | 0.000781998 | clementine0.9_001597m | AT1G09060.3 |  | Zinc finger. RING-type;Transcription factor jumonji/aspartyl beta-hydroxylase |
| 1. clementina_scaffold_1:11376262-11379751 | -1.79769e+308 | 0.000351192 | clementine0.9_018395m | AT1G28200.1 | FIP1 | FH interacting protein 1 |
| 1. clementina_scaffold_10:1064827-1066062 | -1.79769e+308 | 0.00197388 | clementine0.9_000076m | AT3G01460.1 | MBD9 | methyl-CPG-binding domain 9 |
| 1. clementina_scaffold_10:5714582-5717034 | -1.79769e+308 | 0.000639514 | clementine0.9_000313m | AT1G08600.2 | ATRX | P-loop containing nucleoside triphosphate hydrolases superfamily protein |
| 1. clementina_scaffold_10:1110888-1114308 | -1.79769e+308 | 7.90706e-05 | clementine0.9_005777m | AT1G10580.1 |  | Transducin/WD40 repeat-like superfamily protein |
| 1. clementina_scaffold_12:970121-976625 | -1.79769e+308 | 0.000327769 | clementine0.9_002909m | AT5G63320.1 | NPX1 | nuclear protein X1 |
| 1. clementina_scaffold_12:3096067-3096473 | -1.79769e+308 | 0.000417739 | clementine0.9_000141m | AT1G63490.1 |  | transcription factor jumonji (jmjC) domain-containing protein |
| 1. clementina_scaffold_121:353680-356286 | -1.79769e+308 | 0.00263688 | clementine0.9_007012m | AT1G02150.1 |  | Tetratricopeptide repeat (TPR)-like superfamily protein |
| 1. clementina_scaffold_13:1807646-1808035 | -1.79769e+308 | 0.000417739 | clementine0.9_020077m | AT5G39250.1 |  | F-box family protein |
| 1. clementina_scaffold_19:2631258-2634289 | -1.79769e+308 | 0.00129192 | clementine0.9_008778m | AT5G46100.1 |  | Pentatricopeptide repeat (PPR) superfamily protein |
| 1. clementina_scaffold_2:3696851-3697922 | -1.79769e+308 | 0.00168522 | clementine0.9_000149m | AT4G16340.1 | SPK1 | guanyl-nucleotide exchange factors;GTPase binding;GTP binding |
| 1. clementina_scaffold_2:1220207-1223253 | -1.79769e+308 | 0.00265844 | clementine0.9_006334m | AT2G40070.1 |  |  |
| 1. clementina_scaffold_20:1122308-1123620 | -1.79769e+308 | 0.000979823 | clementine0.9_000164m | AT2G13370.1 | CHR5 | chromatin remodeling 5 |
| 1. clementina_scaffold_23:3094182-3098081 | -1.79769e+308 | 0.000554692 |  |  |  |  |
| 1. clementina_scaffold_25:2183559-2184784 | -1.79769e+308 | 0.000836537 | clementine0.9_000275m | AT3G51120.1 |  | DNA binding;zinc ion binding;nucleic acid binding;nucleic acid binding |
| 1. clementina_scaffold_3:316673-317319 | -1.79769e+308 | 0.00168522 | clementine0.9_000037m | AT1G02080.2 |  | transcription regulators |
| 1. clementina_scaffold_3:4147886-4148572 | -1.79769e+308 | 0.00259673 | clementine0.9_003251m | AT1G02660.1 |  | alpha/beta-Hydrolases superfamily protein |
| 1. clementina_scaffold_3:6381757-6383081 | -1.79769e+308 | 0.00168522 | clementine0.9_004547m | AT1G18390.1 |  | Protein kinase superfamily protein |
| 1. clementina_scaffold_3:6600859-6605562 | -1.79769e+308 | 0.00176828 | clementine0.9_017765m | AT4G02580.1 |  | NADH-ubiquinone oxidoreductase 24 kDa subunit. putative |
| 1. clementina_scaffold_34:207383-207978 | -1.79769e+308 | 0.00259673 | clementine0.9_000070m | AT1G77460.1 |  | Armadillo/beta-catenin-like repeat ; C2 calcium/lipid-binding domain (CaLB) protein |
| 1. clementina_scaffold_34:486377-487024 | -1.79769e+308 | 0.000836537 | clementine0.9_000068m | AT1G21580.1 |  | Zinc finger C-x8-C-x5-C-x3-H type family protein |
| 1. clementina_scaffold_34:1208012-1212110 | -1.79769e+308 | 0.00010804 | clementine0.9_014727m | AT1G43700.1 | VIP1 | VIRE2-interacting protein 1 |
| 1. clementina_scaffold_4:2116323-2116872 | -1.79769e+308 | 0.000639514 | clementine0.9_003169m | AT2G18700.1 | TPS11 | trehalose phosphatase/synthase 11 |
| 1. clementina_scaffold_4:4501714-4505019 | -1.79769e+308 | 0.000962281 | clementine0.9_022126m | AT2G26340.1 |  |  |
| 1. clementina_scaffold_4:6260395-6260939 | -1.79769e+308 | 0.000979823 | clementine0.9_000008m | AT1G55860.2 | UPL1 | ubiquitin-protein ligase 1 |
| 1. clementina_scaffold_5:1895464-1898247 | -1.79769e+308 | 6.92005e-05 | clementine0.9_035466m | AT3G42170.1 |  | BED zinc finger ;hAT family dimerisation domain |
| 1. clementina_scaffold_51:29347-32248 | -1.79769e+308 | 0.00200596 | clementine0.9_009520m | AT3G24040.1 |  | Core-2/I-branching beta-1.6-N-acetylglucosaminyltransferase family protein |
| 1. clementina_scaffold_53:1219851-1222283 | -1.79769e+308 | 0.00264903 | clementine0.9_027391m |  |  |  |
| 1. clementina_scaffold_6:4961537-4964097 | -1.79769e+308 | 0.00152096 | clementine0.9_023333m | AT4G25740.1 |  | RNA binding Plectin/S10 domain-containing protein |
| 1. clementina_scaffold_63:542514-543493 | -1.79769e+308 | 0.00128442 | clementine0.9_000021m | AT4G31570.1 |  |  |
| 1. clementina_scaffold_67:127818-128230 | -1.79769e+308 | 0.00259673 | clementine0.9_005129m | AT4G18250.1 |  | receptor serine/threonine kinase. putative |
| 1. clementina_scaffold_7:2711020-2713030 | -1.79769e+308 | 0.0014225 | clementine0.9_023559m | AT3G58090.1 |  | Disease resistance-responsive (dirigent-like protein) family protein |
| 1. clementina_scaffold_7:5047038-5048086 | -1.79769e+308 | 0.00187043 | clementine0.9_023183m | AT3G06840.1 |  |  |
| 1. clementina_scaffold_74:767328-769824 | -1.79769e+308 | 0.000832837 | clementine0.9_016996m | AT5G04490.1 | VTE5 | vitamin E pathway gene 5 |
| 1. clementina_scaffold_8:3061131-3062679 | -1.79769e+308 | 0.00232926 | clementine0.9_006410m | AT3G18670.1 |  | Ankyrin repeat family protein |
| 1. clementina_scaffold_80:168341-169017 | -1.79769e+308 | 0.00259673 | clementine0.9_029972m | AT5G16180.1 | CRS1 | ortholog of maize chloroplast splicing factor CRS1 |
| 1. clementina_scaffold_81:804328-806127 | -1.79769e+308 | 0.000659792 | clementine0.9_009584m | AT1G16870.1 |  | mitochondrial 28S ribosomal protein S29-related |
| 1. clementina_scaffold_8:2238328-2239065 | -1.7932 | 0.00184184 | clementine0.9_001900m | AT3G45850.1 |  | P-loop containing nucleoside triphosphate hydrolases superfamily protein |
| 1. clementina_scaffold_78:445065-452193 | -1.7882 | 2.55007e-08 | clementine0.9_014900m | AT3G15140.1 |  | Polynucleotidyl transferase. ribonuclease H-like superfamily protein |
| 1. clementina_scaffold_3:3372776-3374371 | -1.78316 | 1.22349e-05 | clementine0.9_001507m | AT4G01800.1 | AGY1 | Albino or Glassy Yellow 1 |
| 1. clementina_scaffold_39:548836-550241 | -1.77985 | 0.000545233 | clementine0.9_020666m | AT5G54890.1 |  | RNA-binding CRS1 / YhbY (CRM) domain-containing protein |
| 1. clementina_scaffold_8:2805922-2809448 | -1.77958 | 8.20917e-05 | clementine0.9_002322m | AT5G60410.3 | SIZ1 | DNA-binding protein with MIZ/SP-RING zinc finger. PHD-finger and SAP domain |
| 1. clementina_scaffold_15:3112211-3114423 | -1.77242 | 0.00103674 | clementine0.9_000241m | AT1G30410.1 | MRP13 | multidrug resistance-associated protein 13 |
| 1. clementina_scaffold_16:2220702-2221277 | -1.77052 | 0.000426328 | clementine0.9_007267m | AT4G31940.1 | CYP82C4 | cytochrome P450. family 82. subfamily C. polypeptide 4 |
| 1. clementina_scaffold_3:7647122-7650195 | -1.76987 | 5.8727e-05 | clementine0.9_002560m | AT2G20320.1 |  | DENN (AEX-3) domain-containing protein |
| 1. clementina_scaffold_1:6123319-6126895 | -1.7679 | 0.000483579 | clementine0.9_000837m | AT4G32640.1 |  | Sec23/Sec24 protein transport family protein |
| 1. clementina_scaffold_1:7381042-7383049 | -1.76721 | 0.00163098 | clementine0.9_017086m | AT1G54780.1 | TLP18.3 | thylakoid lumen 18.3 kDa protein |
| 1. clementina_scaffold_16:1559604-1562032 | -1.7668 | 0.000706416 | clementine0.9_007945m | AT3G12570.1 | FYD | FYD |
| 1. clementina_scaffold_1:3621466-3623257 | -1.76522 | 0.000551892 | clementine0.9_033661m | AT1G62720.1 |  | Pentatricopeptide repeat (PPR-like) superfamily protein |
| 1. clementina_scaffold_17:3258331-3259316 | -1.76428 | 0.00212215 | clementine0.9_030736m | AT1G17860.1 |  | Kunitz family trypsin and protease inhibitor protein |
| 1. clementina_scaffold_7:3222629-3224643 | -1.76114 | 0.00231688 | clementine0.9_002969m | AT5G26240.1 | CLC-D | chloride channel D |
| 1. clementina_scaffold_8:4243874-4246253 | -1.75865 | 0.000868685 | clementine0.9_001499m |  |  |  |
| 1. clementina_scaffold_33:2409025-2411152 | -1.74604 | 0.00285836 | clementine0.9_000151m | AT3G14270.1 | FAB1B | phosphatidylinositol-4-phosphate 5-kinase family protein |
| 1. clementina_scaffold_1:9513492-9516384 | -1.73637 | 1.11022e-15 | clementine0.9_004851m | AT2G47430.1 | CKI1 | Signal transduction histidine kinase |
| 1. clementina_scaffold_101:659419-662508 | -1.73637 | 0.00175055 | clementine0.9_001751m | AT2G37050.1 |  | Leucine-rich repeat protein kinase family protein |
| 1. clementina_scaffold_95:295007-297299 | -1.72311 | 0.000143442 | clementine0.9_001216m | AT5G13980.1 |  | Glycosyl hydrolase family 38 protein |
| 1. clementina_scaffold_6:2520886-2521141 | -1.72197 | 3.77565e-10 | clementine0.9_033636m | AT2G17950.1 | WUS | Homeodomain-like superfamily protein |
| 1. clementina_scaffold_19:2392627-2395832 | -1.71926 | 0.0025599 | clementine0.9_018324m | AT1G28960.1 | NUDX15 | nudix hydrolase homolog 15 |
| 1. clementina_scaffold_41:613061-617557 | -1.71365 | 4.86677e-05 | clementine0.9_001601m | AT1G06840.1 |  | Leucine-rich repeat protein kinase family protein |
| 1. clementina_scaffold_4:2965459-2967771 | -1.70849 | 0.00264327 | clementine0.9_001801m | AT5G57250.1 |  | Pentatricopeptide repeat (PPR) superfamily protein |
| 1. clementina_scaffold_19:1024358-1027532 | -1.70801 | 1.80717e-09 | clementine0.9_002425m | AT1G11720.2 | SS3 | starch synthase 3 |
| 1. clementina_scaffold_121:417980-422272 | -1.69866 | 2.11408e-05 | clementine0.9_001069m | AT3G48110.1 | EDD1 | glycine-tRNA ligases |
| 1. clementina_scaffold_3:2312316-2315694 | -1.69431 | 5.08926e-13 | clementine0.9_014342m | AT3G61740.1 | SDG14 | SET domain protein 14 |
| 1. clementina_scaffold_91:222998-224778 | -1.68926 | 0.00256281 | clementine0.9_002120m | AT1G17070.1 |  | GC-rich sequence DNA-binding factor-like protein with Tuftelin interacting domain |
| 1. clementina_scaffold_2:184792-187493 | -1.68881 | 0.00 | clementine0.9_024148m | AT5G02020.1 | SIS |  |
| 1. clementina_scaffold_98:650276-652054 | -1.68606 | 0.00146963 | clementine0.9_030400m | AT3G04160.1 |  |  |
| 1. clementina_scaffold_19:66512-68984 | -1.68355 | 0.00184916 | clementine0.9_008551m | AT5G47810.1 | PFK2 | phosphofructokinase 2 |
| 1. clementina_scaffold_21:2191938-2194798 | -1.6757 | 1.04456e-07 | clementine0.9_018006m | AT3G07310.1 |  | Protein of unknown function (DUF760) |
| 1. clementina_scaffold_7:4114350-4116215 | -1.66753 | 2.80964e-05 | clementine0.9_028703m | AT4G15530.5 | PPDK | pyruvate orthophosphate dikinase |
| 1. clementina_scaffold_19:1642060-1645000 | -1.66709 | 0.000485529 | clementine0.9_000582m | AT4G18050.1 | PGP9 | P-glycoprotein 9 |
| 1. clementina_scaffold_12:932971-937804 | -1.6615 | 2.21047e-08 | clementine0.9_004615m | AT1G67580.1 |  | Protein kinase superfamily protein |
| 1. clementina_scaffold_19:1589850-1592076 | -1.65865 | 3.31275e-09 | clementine0.9_009102m | AT5G46410.2 | SSP4 | SCP1-like small phosphatase 4 |
| 1. clementina_scaffold_1:9382748-9389894 | -1.655 | 6.86791e-10 | clementine0.9_002443m | AT3G05670.1 |  | RING/U-box protein |
| 1. clementina_scaffold_111:37969-43950 | -1.65464 | 0.000661041 | clementine0.9_004223m | AT3G58640.1 |  | Mitogen activated protein kinase kinase kinase-related |
| 1. clementina_scaffold_12:1582389-1584124 | -1.65441 | 3.72083e-06 | clementine0.9_005528m | AT5G63120.2 |  | P-loop containing nucleoside triphosphate hydrolases superfamily protein |
| 1. clementina_scaffold_32:167257-170046 | -1.65284 | 2.07694e-08 | clementine0.9_019175m | AT4G33000.1 | CBL10 | calcineurin B-like protein 10 |
| 1. clementina_scaffold_1:8060806-8060958 | -1.65057 | 0.000472913 | clementine0.9_008522m | AT2G01170.1 | BAT1 | bidirectional amino acid transporter 1 |
| 1. clementina_scaffold_27:2023233-2025739 | -1.64949 | 0.000141734 | clementine0.9_035940m | AT4G28880.1 | ckl3 | casein kinase I-like 3 |
| 1. clementina_scaffold_96:258956-260655 | -1.64381 | 2.7751e-06 | clementine0.9_025256m |  |  |  |
| 1. clementina_scaffold_7:2905947-2907090 | -1.64319 | 1.83441e-05 | clementine0.9_000778m | AT4G26090.1 | RPS2 | NB-ARC domain-containing disease resistance protein |
| 1. clementina_scaffold_8:1135144-1137850 | -1.6411 | 0.0001942 | clementine0.9_023823m | AT5G02020.1 | SIS |  |
| 1. clementina_scaffold_115:143032-143360 | -1.64089 | 4.38551e-05 | clementine0.9_031335m | AT3G08500.1 | MYB83 | myb domain protein 83 |
| 1. clementina_scaffold_32:1122647-1125890 | -1.63826 | 0.000367338 | clementine0.9_001311m | AT1G53350.1 |  | Disease resistance protein (CC-NBS-LRR class) family |
| 1. clementina_scaffold_24:240795-241797 | -1.63494 | 5.4879e-06 | clementine0.9_010360m | AT4G24230.6 | ACBP3 | acyl-CoA-binding domain 3 |
| 1. clementina_scaffold_19:2256342-2259485 | -1.6341 | 1.489e-06 | clementine0.9_018911m | AT1G68810.1 |  | basic helix-loop-helix (bHLH) DNA-binding superfamily protein |
| 1. clementina_scaffold_1:9327626-9331954 | -1.63103 | 0.00129132 | clementine0.9_000869m | AT5G27030.1 | TPR3 | TOPLESS-related 3 |
| 1. clementina_scaffold_7:3566059-3567443 | -1.62887 | 1.80749e-06 | clementine0.9_012314m | AT5G19120.1 |  | Eukaryotic aspartyl protease family protein |
| 1. clementina_scaffold_1:4485933-4495728 | -1.62386 | 0.000272165 | clementine0.9_012707m | AT4G19420.1 |  | Pectinacetylesterase family protein |
| 1. clementina_scaffold_1:2041284-2044527 | -1.62248 | 0.000440022 | clementine0.9_032289m | AT3G02100.1 |  | UDP-Glycosyltransferase superfamily protein |
| 1. clementina_scaffold_87:273747-276293 | -1.62156 | 0.00 | clementine0.9_023069m | AT1G05870.1 |  | Protein of unknown function (DUF1685) |
| 1. clementina_scaffold_11:3936138-3937396 | -1.60616 | 0.0006572 | clementine0.9_005226m | AT5G51920.1 |  | Pyridoxal phosphate (PLP)-dependent transferases superfamily protein |
| 1. clementina_scaffold_3:3520307-3522714 | -1.60537 | 8.56777e-07 | clementine0.9_003838m | AT4G01870.1 |  | tolB protein-related |
| 1. clementina_scaffold_33:1766133-1768954 | -1.59166 | 1.99894e-07 | clementine0.9_002420m | AT1G52150.1 | ATHB-15 | Homeobox-leucine zipper family protein / lipid-binding START domain-containing protein |
| 1. clementina_scaffold_3:4823909-4826856 | -1.59131 | 6.75111e-06 | clementine0.9_000644m | AT1G02890.1 |  | AAA-type ATPase family protein |
| 1. clementina_scaffold_33:1617102-1619089 | -1.5903 | 0.00109263 | clementine0.9_032337m | AT3G16270.1 |  | ENTH/VHS family protein |
| 1. clementina_scaffold_11:2451865-2452326 | -1.58917 | 0.00134353 | clementine0.9_001221m | AT5G17680.1 |  | disease resistance protein (TIR-NBS-LRR class). putative |
| 1. clementina_scaffold_8:1307162-1317801 | -1.58671 | 4.46262e-06 | clementine0.9_031086m | AT2G29210.1 |  | splicing factor PWI domain-containing protein |
| 1. clementina_scaffold_12:532073-537717 | -1.57837 | 0.000158095 | clementine0.9_000013m | AT4G17140.3 |  | pleckstrin homology (PH) domain-containing protein |
| 1. clementina_scaffold_59:1264755-1264993 | -1.57714 | 3.05591e-09 | clementine0.9_000177m | AT4G00990.1 |  | Transcription factor jumonji (jmjC) domain-containing protein |
| 1. clementina_scaffold_63:405865-408370 | -1.5762 | 0.000977221 | clementine0.9_011674m | AT4G31430.1 |  |  |
| 1. clementina_scaffold_54:194170-199996 | -1.56296 | 0.000191174 | clementine0.9_029676m | AT5G04290.1 | KTF1 | kow domain-containing transcription factor 1 |
| 1. clementina_scaffold_85:103502-110790 | -1.56293 | 0.000368259 | clementine0.9_005121m | AT5G49720.1 | GH9A1 | glycosyl hydrolase 9A1 |
| 1. clementina_scaffold_65:814821-817062 | -1.55656 | 6.10016e-07 | clementine0.9_001207m | AT1G76390.1 |  | ARM repeat superfamily protein |
| 1. clementina_scaffold_21:1408652-1412755 | -1.55526 | 3.71698e-08 | clementine0.9_004846m | AT2G32540.1 | CSLB04 | cellulose synthase-like B4 |
| 1. clementina_scaffold_49:460808-465177 | -1.55341 | 0.00147097 | clementine0.9_019379m | AT3G07565.1 |  | Protein of unknown function (DUF3755) |
| 1. clementina_scaffold_3:2881809-2884970 | -1.55173 | 9.09704e-05 | clementine0.9_000096m | AT4G00800.1 |  | transducin family protein / WD-40 repeat family protein |
| 1. clementina_scaffold_9:1165980-1172881 | -1.55127 | 6.64757e-06 | clementine0.9_000426m | AT5G11700.2 |  |  |
| 1. clementina_scaffold_3:7755347-7763402 | -1.55005 | 0.000447529 | clementine0.9_002034m | AT1G04080.1 | PRP39 | Tetratricopeptide repeat (TPR)-like superfamily protein |
| 1. clementina_scaffold_4:3133134-3137982 | -1.54934 | 0.000112979 | clementine0.9_002474m | AT5G57160.1 | ATLIG4 | DNA ligase IV |
| 1. clementina_scaffold_10:1435219-1438102 | -1.54609 | 6.12521e-06 | clementine0.9_000314m | AT1G10760.1 | SEX1 | Pyruvate phosphate dikinase. PEP/pyruvate binding domain |
| 1. clementina_scaffold_8:3857203-3858710 | -1.54309 | 4.28768e-13 | clementine0.9_017203m | AT5G22920.1 |  | CHY-type/CTCHY-type/RING-type Zinc finger protein |
| 1. clementina_scaffold_8:3087901-3088797 | -1.54122 | 5.67303e-09 | clementine0.9_023877m | AT5G60680.1 |  | Protein of unknown function. DUF584 |
| 1. clementina_scaffold_10:1346051-1347385 | -1.54067 | 1.01246e-07 | clementine0.9_011893m | AT1G23390.1 |  | Kelch repeat-containing F-box family protein |
| 1. clementina_scaffold_21:559332-560862 | -1.53356 | 0.000511564 | clementine0.9_024808m | AT2G04520.1 |  | Nucleic acid-binding. OB-fold-like protein |
| 1. clementina_scaffold_117:196520-201702 | -1.53227 | 0.00 | clementine0.9_035799m | AT2G05160.1 |  | CCCH-type zinc fingerfamily protein with RNA-binding domain |
| 1. clementina_scaffold_64:1244721-1247474 | -1.52661 | 5.17161e-05 | clementine0.9_029769m | AT5G35450.1 |  | Disease resistance protein (CC-NBS-LRR class) family |
| 1. clementina_scaffold_2:240971-241818 | -1.52633 | 0.0020007 | clementine0.9_005134m | AT3G54010.1 | PAS1 | FKBP-type peptidyl-prolyl cis-trans isomerase family protein |
| 1. clementina_scaffold_4:6140620-6144262 | -1.52219 | 2.44878e-10 | clementine0.9_005458m | AT5G47750.1 | D6PKL2 | D6 protein kinase like 2 |
| 1. clementina_scaffold_3:8248167-8249899 | -1.51985 | 3.81944e-10 | clementine0.9_011989m | AT3G07120.1 |  | RING/U-box superfamily protein |
| 1. clementina_scaffold_4:1041824-1043877 | -1.51942 | 0.000440573 | clementine0.9_000310m | AT3G46920.1 |  | Protein kinase superfamily protein with octicosapeptide/Phox/Bem1p domain |
| 1. clementina_scaffold_3:7863107-7866408 | -1.5181 | 1.37365e-10 | clementine0.9_000393m | AT2G20190.1 | CLASP | CLIP-associated protein |
| 1. clementina_scaffold_3:6291418-6296210 | -1.51701 | 8.41549e-13 | clementine0.9_022116m | AT4G14713.1 | PPD1 | TIFY domain/Divergent CCT motif family protein |
| 1. clementina_scaffold_26:2861467-2863075 | -1.51331 | 6.17635e-08 |  |  |  |  |
| 1. clementina_scaffold_24:770628-772477 | -1.51057 | 0.000953434 |  |  |  |  |
| 1. clementina_scaffold_25:3268046-3271916 | -1.50968 | 0.000111878 | clementine0.9_000004m | AT2G17930.1 |  | Phosphatidylinositol 3- and 4-kinase family protein with FAT domain |
| 1. clementina_scaffold_47:1006583-1012321 | -1.50404 | 2.45619e-10 | clementine0.9_008624m | AT1G66330.1 |  | senescence-associated family protein |
| 1. clementina_scaffold_26:2944508-2945862 | -1.50157 | 0.00121051 |  |  |  |  |
| 1. clementina_scaffold_49:988766-994172 | -1.50037 | 7.41993e-08 | clementine0.9_035601m | AT2G04160.1 | AIR3 | Subtilisin-like serine endopeptidase family protein |
| 1. clementina_scaffold_22:2454455-2459837 | -1.49764 | 9.11348e-09 | clementine0.9_011310m | AT1G47270.1 | TLP6 | tubby like protein 6 |
| 1. clementina_scaffold_14:4064938-4067839 | -1.49203 | 1.29243e-09 | clementine0.9_001048m | AT3G42170.1 |  | BED zinc finger ;hAT family dimerisation domain |
| 1. clementina_scaffold_30:2441964-2457839 | -1.4865 | 0.000260135 | clementine0.9_000243m | AT5G04140.1 | GLU1 | glutamate synthase 1 |
| 1. clementina_scaffold_14:1161391-1161551 | -1.48409 | 2.37648e-08 | clementine0.9_020970m | AT4G18230.1 |  |  |
| 1. clementina_scaffold_20:734053-739837 | -1.4838 | 2.22045e-16 | clementine0.9_031627m | AT3G28890.1 | RLP43 | receptor like protein 43 |
| 1. clementina_scaffold_11:3708568-3712378 | -1.48333 | 0.00112693 | clementine0.9_002199m | AT1G31480.1 | SGR2 | shoot gravitropism 2 (SGR2) |
| 1. clementina_scaffold_22:141962-145996 | -1.48273 | 5.53446e-07 | clementine0.9_034245m | AT1G76630.2 |  | Tetratricopeptide repeat (TPR)-like superfamily protein |
| 1. clementina_scaffold_22:634327-636836 | -1.47997 | 1.79265e-09 | clementine0.9_030638m | AT5G42340.1 | PUB15 | Plant U-Box 15 |
| 1. clementina_scaffold_3:5735827-5736981 | -1.47904 | 7.46842e-07 | clementine0.9_011390m | AT5G63380.1 |  | AMP-dependent synthetase and ligase family protein |
| 1. clementina_scaffold_1:9632015-9634503 | -1.4788 | 4.67493e-12 | clementine0.9_000637m | AT4G27220.1 |  | NB-ARC domain-containing disease resistance protein |
| 1. clementina_scaffold_5:2085255-2085415 | -1.47478 | 9.58069e-08 | clementine0.9_003758m | AT5G45380.1 | DUR3 | solute:sodium symporters;urea transmembrane transporters |
| 1. clementina_scaffold_1:12560457-12562175 | -1.47142 | 0.00137233 | clementine0.9_002794m | AT1G21980.1 | PIP5K1 | phosphatidylinositol-4-phosphate 5-kinase 1 |
| 1. clementina_scaffold_1:2692811-2694398 | -1.47099 | 0.000637314 | clementine0.9_005416m | AT1G31920.1 |  | Tetratricopeptide repeat (TPR)-like superfamily protein |
| 1. clementina_scaffold_79:992658-992813 | -1.46789 | 1.09609e-06 | clementine0.9_030691m | AT1G16760.1 |  | Protein kinase protein with adenine nucleotide alpha hydrolases-like domain |
| 1. clementina_scaffold_47:1507043-1510085 | -1.46486 | 0.0024817 | clementine0.9_015549m | AT5G37930.1 |  | Protein with RING/U-box and TRAF-like domains |
| 1. clementina_scaffold_1:12640292-12641158 | -1.4638 | 0.00107315 | clementine0.9_023792m | AT4G36020.1 | CSDP1 | cold shock domain protein 1 |
| 1. clementina_scaffold_80:704989-706002 | -1.46334 | 0.000681491 | clementine0.9_028744m | AT1G70630.1 |  | Nucleotide-diphospho-sugar transferase family protein |
| 1. clementina_scaffold_53:621853-629813 | -1.45695 | 4.51653e-05 | clementine0.9_002997m | AT3G01180.1 | SS2 | starch synthase 2 |
| 1. clementina_scaffold_112:470153-473823 | -1.45426 | 3.91686e-05 | clementine0.9_000384m | AT1G64570.1 | DUO3 | Homeodomain-like superfamily protein |
| 1. clementina_scaffold_44:1143095-1149642 | -1.45244 | 6.48045e-07 | clementine0.9_000162m | AT2G16485.1 |  | nucleic acid binding;zinc ion binding;DNA binding |
| 1. clementina_scaffold_9:1014315-1018670 | -1.44749 | 6.18757e-08 | clementine0.9_004689m | AT1G79750.1 | NADP-ME4 | NADP-malic enzyme 4 |
| 1. clementina_scaffold_56:824841-832742 | -1.44706 | 7.14435e-07 | clementine0.9_002184m | AT2G01460.1 |  | P-loop containing nucleoside triphosphate hydrolases superfamily protein |
| 1. clementina_scaffold_22:105079-108278 | -1.44665 | 0.00227496 | clementine0.9_000619m | AT5G42390.1 |  | Insulinase (Peptidase family M16) family protein |
| 1. clementina_scaffold_34:488473-491826 | -1.4309 | 0.000110186 | clementine0.9_000068m | AT1G21580.1 |  | Zinc finger C-x8-C-x5-C-x3-H type family protein |
| 1. clementina_scaffold_5:3197561-3201495 | -1.42722 | 0.000231636 | clementine0.9_011480m | AT2G20650.1 |  | RING/U-box superfamily protein |
| 1. clementina_scaffold_12:857181-860900 | -1.4272 | 3.57647e-12 | clementine0.9_003039m | AT5G14210.1 |  | Leucine-rich repeat protein kinase family protein |
| 1. clementina_scaffold_48:154606-157077 | -1.42456 | 4.17274e-05 | clementine0.9_003189m | AT1G68690.1 |  | Protein kinase superfamily protein |
| 1. clementina_scaffold_63:1063231-1065052 | -1.41996 | 6.63989e-06 | clementine0.9_009733m | AT5G10930.1 | CIPK5 | CBL-interacting protein kinase 5 |
| 1. clementina_scaffold_33:1906430-1909735 | -1.40753 | 4.07241e-05 | clementine0.9_010089m | AT1G51940.1 |  | protein kinase family protein / peptidoglycan-binding LysM domain-containing protein |
| 1. clementina_scaffold_41:1916230-1917887 | -1.40516 | 4.19732e-07 | clementine0.9_006228m | AT5G63950.1 | CHR24 | chromatin remodeling 24 |
| 1. clementina_scaffold_47:671351-676810 | -1.40291 | 0.000947446 | clementine0.9_005033m | AT1G68570.1 |  | Major facilitator superfamily protein |
| 1. clementina_scaffold_80:256553-258316 | -1.40239 | 0.000401086 | clementine0.9_018467m | AT1G73830.1 | BEE3 | BR enhanced expression 3 |
| 1. clementina_scaffold_1:6035520-6046136 | -1.39928 | 0.000928948 | clementine0.9_016545m | AT3G04880.1 | DRT102 | DNA-damage-repair/toleration protein (DRT102) |
| 1. clementina_scaffold_7:3237248-3241775 | -1.39672 | 9.76862e-05 | clementine0.9_012811m | AT4G19600.1 | CYCT1;4 | Cyclin family protein |
| 1. clementina_scaffold_12:2992189-2995525 | -1.39404 | 0.00270732 | clementine0.9_020601m | AT3G47670.1 |  | Plant invertase/pectin methylesterase inhibitor superfamily protein |
| 1. clementina_scaffold_82:139269-140533 | -1.39115 | 0.000401014 | clementine0.9_017886m | AT4G32060.1 |  | calcium-binding EF hand family protein |
| 1. clementina_scaffold_71:631738-640239 | -1.38779 | 0.000289647 | clementine0.9_010029m | AT4G17020.1 |  | transcription factor-related |
| 1. clementina_scaffold_20:573238-577117 | -1.38292 | 1.41399e-07 | clementine0.9_006051m | AT3G46730.1 |  | NB-ARC domain-containing disease resistance protein |
| 1. clementina_scaffold_27:346651-349141 | -1.38028 | 0.000213471 | clementine0.9_004573m | AT5G55860.1 |  | Plant protein of unknown function (DUF827) |
| 1. clementina_scaffold_132:448490-451891 | -1.37836 | 6.80715e-05 | clementine0.9_003644m | AT2G27810.1 | NAT12 | nucleobase-ascorbate transporter 12 |
| 1. clementina_scaffold_113:196395-199216 | -1.37694 | 0.000643609 | clementine0.9_025257m |  |  |  |
| 1. clementina_scaffold_67:627707-634676 | -1.37469 | 0.00186632 | clementine0.9_002745m | AT5G23450.1 | LCBK1 | long-chain base (LCB) kinase 1 |
| 1. clementina_scaffold_63:511312-512982 | -1.37097 | 0.000243643 | clementine0.9_017035m | AT5G24890.1 |  |  |
| 1. clementina_scaffold_3:5975624-5977375 | -1.36961 | 6.29275e-05 | clementine0.9_000558m | AT3G22380.2 | TIC | time for coffee |
| 1. clementina_scaffold_1:10148785-10150301 | -1.36813 | 8.58202e-12 | clementine0.9_018899m | AT3G03990.1 |  | alpha/beta-Hydrolases superfamily protein |
| 1. clementina_scaffold_8:3660879-3662275 | -1.36146 | 1.2373e-06 | clementine0.9_027597m | AT4G38460.1 | GGR | geranylgeranyl reductase |
| 1. clementina_scaffold_34:1268956-1276907 | -1.36075 | 0.000631127 | clementine0.9_001915m | AT1G76850.1 | SEC5A | exocyst complex component sec5 |
| 1. clementina_scaffold_3:4457823-4463486 | -1.35872 | 3.56427e-09 | clementine0.9_002649m | AT4G02280.1 | SUS3 | sucrose synthase 3 |
| 1. clementina_scaffold_1:8341257-8344315 | -1.35713 | 1.12257e-06 | clementine0.9_028642m | AT3G14470.1 |  | NB-ARC domain-containing disease resistance protein |
| 1. clementina_scaffold_22:2313177-2317329 | -1.35696 | 2.83511e-07 | clementine0.9_001988m | AT1G19720.1 |  | Pentatricopeptide repeat (PPR-like) superfamily protein |
| 1. clementina_scaffold_19:1920425-1923390 | -1.35337 | 2.37376e-06 | clementine0.9_002222m | AT1G06410.1 | TPS7 | trehalose-phosphatase/synthase 7 |
| 1. clementina_scaffold_16:511987-515937 | -1.34807 | 6.28963e-08 | clementine0.9_008802m | AT5G03040.1 | iqd2 | IQ-domain 2 |
| 1. clementina_scaffold_1:10553692-10554920 | -1.33889 | 1.04133e-06 | clementine0.9_024405m | AT4G14960.1 | TUA6 | Tubulin/FtsZ family protein |
| 1. clementina_scaffold_21:660229-663826 | -1.33348 | 0.00103859 | clementine0.9_003126m | AT2G32900.1 | ATZW10 | centromere/kinetochore protein. putative (ZW10) |
| 1. clementina_scaffold_20:1325349-1328509 | -1.33208 | 6.55583e-07 | clementine0.9_010239m | AT5G17540.1 |  | HXXXD-type acyl-transferase family protein |
| 1. clementina_scaffold_9:1079753-1084906 | -1.33197 | 2.53975e-07 | clementine0.9_005249m | AT4G32850.5 | nPAP | nuclear poly(a) polymerase |
| 1. clementina_scaffold_3:5489769-5496709 | -1.32742 | 3.56981e-12 | clementine0.9_000033m | AT2G48060.1 |  |  |
| 1. clementina_scaffold_148:253994-258884 | -1.32721 | 3.17331e-11 | clementine0.9_003816m | AT1G10240.1 | FRS11 | FAR1-related sequence 11 |
| 1. clementina_scaffold_30:2649573-2653152 | -1.32654 | 3.48325e-08 | clementine0.9_017712m | AT2G41250.1 |  | Haloacid dehalogenase-like hydrolase (HAD) superfamily protein |
| 1. clementina_scaffold_23:1638490-1639848 | -1.32092 | 0.00087037 |  |  |  |  |
| 1. clementina_scaffold_40:155895-160864 | -1.32007 | 1.67949e-09 | clementine0.9_003023m | AT4G26140.1 | BGAL12 | beta-galactosidase 12 |
| 1. clementina_scaffold_53:222970-226768 | -1.31997 | 0.000657193 | clementine0.9_000154m | AT5G15540.1 | EMB2773 | PHD finger family protein |
| 1. clementina_scaffold_88:224888-227865 | -1.31823 | 0.000970587 | clementine0.9_014402m | AT1G52630.2 |  | O-fucosyltransferase family protein |
| 1. clementina_scaffold_108:391958-393397 | -1.3178 | 0.00149437 | clementine0.9_030019m | AT4G10780.1 |  | LRR and NB-ARC domains-containing disease resistance protein |
| 1. clementina_scaffold_34:400277-405917 | -1.31176 | 8.01581e-14 | clementine0.9_002053m | AT1G21640.1 | NADK2 | NAD kinase 2 |
| 1. clementina_scaffold_7:4526851-4528700 | -1.30537 | 0.000530401 | clementine0.9_018431m | AT5G18610.1 |  | Protein kinase superfamily protein |
| 1. clementina_scaffold_10:4252365-4254703 | -1.2979 | 0.000357833 | clementine0.9_012670m | AT1G25550.1 |  | myb-like transcription factor family protein |
| 1. clementina_scaffold_10:4075500-4081326 | -1.29746 | 6.6974e-06 | clementine0.9_001419m | AT3G25690.1 | CHUP1 | Hydroxyproline-rich glycoprotein family protein |
| 1. clementina_scaffold_8:3309459-3316658 | -1.29318 | 1.11022e-15 | clementine0.9_029776m | AT2G28250.1 | NCRK | Protein kinase superfamily protein |
| 1. clementina_scaffold_137:134921-136434 | -1.2905 | 2.0855e-05 | clementine0.9_009591m | AT1G03940.1 |  | HXXXD-type acyl-transferase family protein |
| 1. clementina_scaffold_114:295052-297463 | -1.28531 | 0.000517784 | clementine0.9_034208m | AT5G14990.1 |  |  |
| 1. clementina_scaffold_7:3181463-3184549 | -1.28504 | 1.5887e-07 | clementine0.9_000746m | AT3G22380.2 | TIC | time for coffee |
| 1. clementina_scaffold_16:1486740-1497206 | -1.27956 | 1.57013e-05 | clementine0.9_000650m | AT2G39580.1 |  |  |
| 1. clementina_scaffold_98:257354-259964 | -1.27942 | 1.54264e-06 | clementine0.9_022127m | AT1G08570.1 | ACHT4 | atypical CYS HIS rich thioredoxin 4 |
| 1. clementina_scaffold_67:708155-719044 | -1.27894 | 2.18711e-05 | clementine0.9_003391m | AT1G65580.1 | FRA3 | Endonuclease/exonuclease/phosphatase family protein |
| 1. clementina_scaffold_126:347475-348536 | -1.27704 | 5.85759e-06 | clementine0.9_028562m | AT3G23990.1 | HSP60 | heat shock protein 60 |
| 1. clementina_scaffold_23:3459210-3461980 | -1.27196 | 0.00207898 |  |  |  |  |
| 1. clementina_scaffold_96:410108-411929 | -1.2703 | 0.00280917 | clementine0.9_001853m | AT3G25500.1 | AFH1 | formin homology 1 |
| 1. clementina_scaffold_1:2713568-2717722 | -1.27002 | 0.00152401 | clementine0.9_006941m | AT1G32130.1 | IWS1 | Transcription elongation factor (TFIIS) family protein |
| 1. clementina_scaffold_8:533739-540682 | -1.26577 | 0.00035002 | clementine0.9_005735m | AT3G47340.1 | ASN1 | glutamine-dependent asparagine synthase 1 |
| 1. clementina_scaffold_15:2745998-2746559 | -1.26285 | 0.00141061 | clementine0.9_016328m | AT4G18370.1 | DEG5 | DEGP protease 5 |
| 1. clementina_scaffold_111:322772-323777 | -1.26184 | 0.00172036 | clementine0.9_005561m | AT3G59040.1 |  | Tetratricopeptide repeat (TPR)-like superfamily protein |
| 1. clementina_scaffold_47:197644-200621 | -1.25775 | 0.00101336 | clementine0.9_002649m | AT4G02280.1 | SUS3 | sucrose synthase 3 |
| 1. clementina_scaffold_44:1351450-1354505 | -1.25765 | 0.000224188 | clementine0.9_004942m | AT1G12700.1 |  | ATP binding;nucleic acid binding;helicases |
| 1. clementina_scaffold_7:4487677-4489637 | -1.25355 | 0.0027342 | clementine0.9_003620m | AT5G18590.1 |  | Galactose oxidase/kelch repeat superfamily protein |
| 1. clementina_scaffold_3:5436349-5440842 | -1.25149 | 1.93539e-10 | clementine0.9_004551m | AT2G48010.1 | RKF3 | receptor-like kinase in in flowers 3 |
| 1. clementina_scaffold_3:5320353-5325210 | -1.25135 | 9.19225e-06 | clementine0.9_001652m | AT4G03090.1 |  | sequence-specific DNA binding;sequence-specific DNA binding transcription factors |
| 1. clementina_scaffold_4:611938-614274 | -1.25112 | 0.000229794 | clementine0.9_034714m | AT3G14470.1 |  | NB-ARC domain-containing disease resistance protein |
| 1. clementina_scaffold_25:1808161-1810818 | -1.25069 | 2.8007e-09 | clementine0.9_010580m | AT5G66460.1 | MAN7 | Glycosyl hydrolase superfamily protein |
| 1. clementina_scaffold_22:647681-653627 | -1.24908 | 0.00 | clementine0.9_002290m | AT2G30600.1 |  | BTB/POZ domain-containing protein |
| 1. clementina_scaffold_1:8967791-8971149 | -1.24729 | 0.000257737 | clementine0.9_020031m | AT4G15920.1 | SWEET17 | Nodulin MtN3 family protein |
| 1. clementina_scaffold_47:158304-160340 | -1.24548 | 0.000804521 | clementine0.9_006243m | AT1G65890.1 | AAE12 | acyl activating enzyme 12 |
| 1. clementina_scaffold_2:5761372-5762653 | -1.24255 | 3.83799e-06 | clementine0.9_014909m | AT2G27310.1 |  | F-box family protein |
| 1. clementina_scaffold_15:3252607-3262009 | -1.23854 | 0.000424698 | clementine0.9_002422m | AT1G30470.1 |  | SIT4 phosphatase-associated family protein |
| 1. clementina_scaffold_2:4402058-4403365 | -1.23733 | 0.000549388 | clementine0.9_002177m | AT3G56150.1 | EIF3C | eukaryotic translation initiation factor 3C |
| 1. clementina_scaffold_6:1056886-1058235 | -1.23642 | 0.000226177 | clementine0.9_026320m | AT2G42310.1 |  |  |
| 1. clementina_scaffold_1:9006192-9009035 | -1.23163 | 2.65836e-10 | clementine0.9_033815m | AT4G15920.1 | SWEET17 | Nodulin MtN3 family protein |
| 1. clementina_scaffold_19:3753092-3754230 | -1.23162 | 6.0528e-05 | clementine0.9_022492m | AT4G27310.1 |  | B-box type zinc finger family protein |
| 1. clementina_scaffold_24:1456888-1462684 | -1.23087 | 0.000125161 |  |  |  |  |
| 1. clementina_scaffold_6:5899767-5905792 | -1.22785 | 1.19233e-05 | clementine0.9_000053m | AT5G53460.1 | GLT1 | NADH-dependent glutamate synthase 1 |
| 1. clementina_scaffold_56:12639-17194 | -1.22543 | 3.25758e-06 | clementine0.9_002230m | AT1G68020.2 | ATTPS6 | UDP-Glycosyltransferase / trehalose-phosphatase family protein |
| 1. clementina_scaffold_12:4320772-4322164 | -1.22464 | 3.58842e-06 | clementine0.9_013711m | AT1G64500.1 |  | Glutaredoxin family protein |
| 1. clementina_scaffold_3:5693440-5694049 | -1.22304 | 3.33069e-05 | clementine0.9_028671m | AT3G63088.1 | RTFL14 | ROTUNDIFOLIA like 14 |
| 1. clementina_scaffold_2:906355-909095 | -1.22163 | 2.45425e-05 | clementine0.9_008089m | AT2G38110.1 | GPAT6 | glycerol-3-phosphate acyltransferase 6 |
| 1. clementina_scaffold_43:876505-880738 | -1.21928 | 2.74947e-05 | clementine0.9_004947m | AT2G31820.1 |  | Ankyrin repeat family protein |
| 1. clementina_scaffold_7:1448240-1453749 | -1.21857 | 0.00114155 | clementine0.9_002165m | AT2G37520.1 |  | Acyl-CoA N-acyltransferase with RING/FYVE/PHD-type zinc finger domain |
| 1. clementina_scaffold_1:7383513-7386513 | -1.21609 | 2.22045e-16 | clementine0.9_016418m | AT5G27390.1 |  | Mog1/PsbP/DUF1795-like photosystem II reaction center PsbP family protein |
| 1. clementina_scaffold_10:1480639-1483875 | -1.21237 | 4.01598e-09 | clementine0.9_006055m | AT3G26570.2 | PHT2;1 | phosphate transporter 2;1 |
| 1. clementina_scaffold_23:1449430-1452834 | -1.21152 | 3.70501e-06 | clementine0.9_009917m | AT1G47240.1 | NRAMP2 | NRAMP metal ion transporter 2 |
| 1. clementina_scaffold_4:2309561-2313359 | -1.21116 | 0.000552782 | clementine0.9_010552m | AT4G25770.1 |  | alpha/beta-Hydrolases superfamily protein |
| 1. clementina_scaffold_7:4936375-4939016 | -1.21072 | 0.00193902 | clementine0.9_003521m | AT3G06860.1 | MFP2 | multifunctional protein 2 |
| 1. clementina_scaffold_38:1855930-1860061 | -1.20984 | 0.00232875 | clementine0.9_002300m | AT5G64580.1 |  | AAA-type ATPase family protein |
| 1. clementina_scaffold_7:6329093-6334231 | -1.20747 | 1.05337e-10 | clementine0.9_033245m | AT3G53480.1 | PDR9 | pleiotropic drug resistance 9 |
| 1. clementina_scaffold_2:3455520-3460678 | -1.20727 | 6.66223e-12 | clementine0.9_002742m | AT2G32250.1 | FRS2 | FAR1-related sequence 2 |
| 1. clementina_scaffold_46:1438664-1447201 | -1.20649 | 8.30336e-12 | clementine0.9_009527m | AT2G30710.1 |  | Ypt/Rab-GAP domain of gyp1p superfamily protein |
| 1. clementina_scaffold_9:3042071-3045179 | -1.20546 | 8.24797e-06 | clementine0.9_009018m | AT2G22590.1 |  | UDP-Glycosyltransferase superfamily protein |
| 1. clementina_scaffold_2:6625536-6630044 | -1.20049 | 0.00265955 | clementine0.9_001878m | AT1G12430.1 | ARK3 | armadillo repeat kinesin 3 |
| 1. clementina_scaffold_4:7020134-7023260 | -1.2001 | 0.000974942 | clementine0.9_032549m | AT5G55125.1 |  | Ribosomal protein L31 |
| 1. clementina_scaffold_18:3465160-3472657 | -1.1975 | 0.000525615 | clementine0.9_000851m | AT3G17740.1 |  |  |
| 1. clementina_scaffold_14:332488-338854 | -1.1974 | 2.49818e-05 | clementine0.9_001638m | AT1G72180.1 |  | Leucine-rich receptor-like protein kinase family protein |
| 1. clementina_scaffold_51:961029-964197 | -1.19539 | 4.50877e-08 | clementine0.9_005927m | AT3G23920.1 | BAM1 | beta-amylase 1 |
| 1. clementina_scaffold_4:5237994-5244269 | -1.19265 | 6.40921e-05 | clementine0.9_008372m | AT1G19170.1 |  | Pectin lyase-like superfamily protein |
| 1. clementina_scaffold_121:87454-89745 | -1.19255 | 0.00078789 | clementine0.9_026680m |  |  |  |
| 1. clementina_scaffold_10:307368-312617 | -1.19048 | 0.000607807 | clementine0.9_019318m | AT2G02710.1 | PLPB | PAS/LOV protein B |
| 1. clementina_scaffold_21:3882164-3884089 | -1.19022 | 0.00224161 | clementine0.9_008540m | AT2G43020.1 | PAO2 | polyamine oxidase 2 |
| 1. clementina_scaffold_19:3583939-3589141 | -1.18567 | 1.67646e-05 | clementine0.9_005812m | AT3G62980.1 | TIR1 | F-box/RNI-like superfamily protein |
| 1. clementina_scaffold_16:968363-972461 | -1.18509 | 1.49697e-07 | clementine0.9_006578m | AT1G28060.1 |  | Pre-mRNA-splicing factor 3 |
| 1. clementina_scaffold_3:894212-901309 | -1.18386 | 2.38139e-06 | clementine0.9_000277m | AT2G45540.1 |  | WD-40 repeat family protein / beige-related |
| 1. clementina_scaffold_1:10684671-10685806 | -1.18201 | 0.000833289 | clementine0.9_015318m | AT2G22670.4 | IAA8 | indoleacetic acid-induced protein 8 |
| 1. clementina_scaffold_1:4049593-4051035 | -1.18019 | 0.000167291 | clementine0.9_012636m | AT1G31350.1 | KUF1 | KAR-UP F-box 1 |
| 1. clementina_scaffold_91:739130-743123 | -1.17992 | 1.92211e-05 | clementine0.9_029474m |  |  |  |
| 1. clementina_scaffold_63:84767-86704 | -1.17801 | 0.0017706 | clementine0.9_022763m | AT4G31130.1 |  | Protein of unknown function (DUF1218) |
| 1. clementina_scaffold_87:254875-260951 | -1.17401 | 3.10862e-15 | clementine0.9_016006m | AT2G43330.1 | INT1 | inositol transporter 1 |
| 1. clementina_scaffold_82:304186-306450 | -1.17181 | 2.30502e-05 | clementine0.9_004016m | AT4G32190.1 |  | Myosin heavy chain-related protein |
| 1. clementina_scaffold_16:1321444-1328031 | -1.17092 | 0.000816461 | clementine0.9_000429m | AT3G55320.1 | PGP20 | P-glycoprotein 20 |
| 1. clementina_scaffold_118:248273-251637 | -1.16878 | 1.62637e-09 | clementine0.9_001548m | AT3G14470.1 |  | NB-ARC domain-containing disease resistance protein |
| 1. clementina_scaffold_8:5924771-5929370 | -1.16527 | 0.000264967 | clementine0.9_018172m | AT2G32520.1 |  | alpha/beta-Hydrolases superfamily protein |
| 1. clementina_scaffold_104:78977-82491 | -1.1645 | 2.16523e-05 | clementine0.9_006412m | AT3G07130.1 | PAP15 | purple acid phosphatase 15 |
| 1. clementina_scaffold_28:493753-499048 | -1.16392 | 0.000110749 | clementine0.9_001064m | AT4G34830.1 | MRL1 | Pentatricopeptide repeat (PPR) superfamily protein |
| 1. clementina_scaffold_52:609083-610976 | -1.16153 | 0.000423388 | clementine0.9_033094m | AT5G59810.1 | SBT5.4 | Subtilase family protein |
| 1. clementina_scaffold_31:1266889-1268167 | -1.15753 | 0.000208839 | clementine0.9_013041m | AT3G12120.1 | FAD2 | fatty acid desaturase 2 |
| 1. clementina_scaffold_12:2357711-2363384 | -1.15725 | 1.66102e-08 | clementine0.9_006271m | AT5G62570.2 |  | Calmodulin binding protein-like |
| 1. clementina_scaffold_12:701904-706547 | -1.15634 | 9.89392e-05 | clementine0.9_000355m | AT5G47020.1 |  |  |
| 1. clementina_scaffold_4:7434635-7438809 | -1.15206 | 8.1176e-07 | clementine0.9_005938m | AT4G03500.1 |  | Ankyrin repeat family protein |
| 1. clementina_scaffold_2:5198671-5203707 | -1.14964 | 0.000232107 | clementine0.9_010995m | AT2G24280.1 |  | alpha/beta-Hydrolases superfamily protein |
| 1. clementina_scaffold_3:1419871-1421166 | -1.14903 | 0.00169838 | clementine0.9_010067m | AT3G49050.1 |  | alpha/beta-Hydrolases superfamily protein |
| 1. clementina_scaffold_2:4262996-4266898 | -1.14682 | 0.000711003 | clementine0.9_006555m | AT2G42520.1 |  | P-loop containing nucleoside triphosphate hydrolases superfamily protein |
| 1. clementina_scaffold_20:1208325-1211209 | -1.14677 | 3.1862e-05 | clementine0.9_010239m | AT5G17540.1 |  | HXXXD-type acyl-transferase family protein |
| 1. clementina_scaffold_3:2990934-2997457 | -1.14661 | 7.04987e-08 | clementine0.9_004559m | AT1G01060.1 | LHY | Homeodomain-like superfamily protein |
| 1. clementina_scaffold_19:2325179-2327744 | -1.14653 | 0.000166044 | clementine0.9_013830m | AT5G45910.1 |  | GDSL-like Lipase/Acylhydrolase superfamily protein |
| 1. clementina_scaffold_3:4112624-4119046 | -1.14652 | 1.08296e-10 | clementine0.9_002988m | AT1G02640.1 | BXL2 | beta-xylosidase 2 |
| 1. clementina_scaffold_8:4658972-4660695 | -1.1464 | 1.81191e-05 | clementine0.9_022442m | AT2G27830.1 |  |  |
| 1. clementina_scaffold_9:1160975-1165629 | -1.14587 | 3.73682e-07 | clementine0.9_000426m | AT5G11700.2 |  |  |
| 1. clementina_scaffold_4:4583615-4585241 | -1.14556 | 0.00128191 | clementine0.9_019258m | AT5G21940.1 |  |  |
| 1. clementina_scaffold_67:465069-466841 | -1.14531 | 3.44993e-05 | clementine0.9_009653m | AT1G25440.1 |  | B-box type zinc finger protein with CCT domain |
| 1. clementina_scaffold_60:685529-687036 | -1.14384 | 0.00218431 | clementine0.9_006707m | AT3G53130.1 | LUT1 | Cytochrome P450 superfamily protein |
| 1. clementina_scaffold_1:11121693-11130157 | -1.14335 | 3.11339e-05 | clementine0.9_003881m | AT1G58200.1 | MSL3 | MSCS-like 3 |
| 1. clementina_scaffold_32:1127458-1130323 | -1.14221 | 0.000112781 | clementine0.9_002347m | AT5G45540.1 |  | Protein of unknown function (DUF594) |
| 1. clementina_scaffold_148:112955-117094 | -1.12862 | 0.00128496 | clementine0.9_004740m | AT5G49890.1 | CLC-C | chloride channel C |
| 1. clementina_scaffold_30:479207-481520 | -1.12582 | 1.10649e-10 | clementine0.9_013949m | AT5G54160.1 | OMT1 | O-methyltransferase 1 |
| 1. clementina_scaffold_28:174361-175173 | -1.12244 | 0.000110148 | clementine0.9_026150m | AT1G75580.1 |  | SAUR-like auxin-responsive protein family |
| 1. clementina_scaffold_25:512630-514546 | -1.12191 | 1.24538e-09 |  |  |  |  |
| 1. clementina_scaffold_143:153786-158903 | -1.11826 | 7.29318e-05 | clementine0.9_004308m | AT1G48870.1 |  | Transducin/WD40 repeat-like superfamily protein |
| 1. clementina_scaffold_61:955544-960764 | -1.11716 | 1.91029e-05 | clementine0.9_006394m | AT4G18020.1 | APRR2 | CheY-like two-component responsive regulator family protein |
| 1. clementina_scaffold_25:1481263-1486601 | -1.11619 | 5.40235e-11 | clementine0.9_009016m | AT3G50670.1 | U1-70K | U1 small nuclear ribonucleoprotein-70K |
| 1. clementina_scaffold_6:532875-536085 | -1.11167 | 2.65678e-05 | clementine0.9_003844m | AT3G42170.1 |  | BED zinc finger ;hAT family dimerisation domain |
| 1. clementina_scaffold_38:1279752-1283970 | -1.11048 | 0.000266526 | clementine0.9_033794m | AT5G02930.1 |  | F-box/RNI-like superfamily protein |
| 1. clementina_scaffold_84:842599-849527 | -1.10796 | 1.52729e-09 | clementine0.9_001292m | AT5G13980.1 |  | Glycosyl hydrolase family 38 protein |
| 1. clementina_scaffold_2:1186297-1193548 | -1.10777 | 3.76936e-05 | clementine0.9_003170m | AT5G01270.2 | CPL2 | carboxyl-terminal domain (ctd) phosphatase-like 2 |
| 1. clementina_scaffold_4:2776979-2779882 | -1.1063 | 8.30954e-05 | clementine0.9_001613m | AT4G30190.1 | HA2 | H(+)-ATPase 2 |
| 1. clementina_scaffold_16:2539312-2541218 | -1.10619 | 8.10744e-05 | clementine0.9_024865m | AT2G41430.1 | ERD15 | dehydration-induced protein (ERD15) |
| 1. clementina_scaffold_16:2731553-2736135 | -1.10532 | 0.0026798 | clementine0.9_035822m | AT5G20420.1 | CHR42 | chromatin remodeling 42 |
| 1. clementina_scaffold_96:257384-258749 | -1.10528 | 9.02955e-05 | clementine0.9_034591m | AT5G17680.1 |  | disease resistance protein (TIR-NBS-LRR class). putative |
| 1. clementina_scaffold_8:3175870-3181950 | -1.09776 | 0.00156616 | clementine0.9_002134m | AT1G07990.1 |  | SIT4 phosphatase-associated family protein |
| 1. clementina_scaffold_10:2168065-2172009 | -1.09747 | 0.000325467 | clementine0.9_008422m | AT3G26000.1 |  | Ribonuclease inhibitor |
| 1. clementina_scaffold_1:9362139-9365621 | -1.09456 | 6.74252e-05 | clementine0.9_010936m | AT1G09160.1 |  | Protein phosphatase 2C family protein |
| 1. clementina_scaffold_3:3438416-3440098 | -1.09438 | 3.36553e-06 | clementine0.9_026209m | AT1G75250.1 | RL6 | RAD-like 6 |
| 1. clementina_scaffold_39:1422570-1424769 | -1.09432 | 0.000543011 | clementine0.9_016755m | AT5G13170.1 | SAG29 | senescence-associated gene 29 |
| 1. clementina_scaffold_19:3351405-3356034 | -1.09216 | 0.000128993 | clementine0.9_001095m | AT1G61210.1 |  | Transducin/WD40 repeat-like superfamily protein |
| 1. clementina_scaffold_101:732290-739987 | -1.09179 | 6.39311e-12 | clementine0.9_001362m | AT3G45630.1 |  | RNA binding (RRM/RBD/RNP motifs) family protein |
| 1. clementina_scaffold_3:204085-212691 | -1.08973 | 2.45679e-05 | clementine0.9_003101m | AT2G45030.1 |  | Translation elongation factor EFG/EF2 protein |
| 1. clementina_scaffold_12:3081655-3088810 | -1.08959 | 0.000100607 | clementine0.9_011063m | AT5G11800.1 | KEA6 | K+ efflux antiporter 6 |
| 1. clementina_scaffold_19:3872070-3873742 | -1.0883 | 0.000149273 | clementine0.9_018428m | AT1G11440.1 |  |  |
| 1. clementina_scaffold_85:824673-827089 | -1.08738 | 0.000455709 | clementine0.9_002849m | AT3G01610.1 | CDC48C | cell division cycle 48C |
| 1. clementina_scaffold_26:2060841-2068173 | -1.08717 | 5.28466e-14 | clementine0.9_004135m | AT4G04850.2 | KEA3 | K+ efflux antiporter 3 |
| 1. clementina_scaffold_4:6745600-6749638 | -1.08679 | 4.51451e-08 | clementine0.9_009075m | AT3G07810.1 |  | RNA-binding (RRM/RBD/RNP motifs) family protein |
| 1. clementina_scaffold_19:3632083-3632679 | -1.08578 | 0.0016971 | clementine0.9_026700m | AT4G05070.1 |  | Wound-responsive family protein |
| 1. clementina_scaffold_4:1713554-1728461 | -1.0843 | 5.71433e-05 | clementine0.9_000137m | AT4G28080.1 |  | Tetratricopeptide repeat (TPR)-like superfamily protein |
| 1. clementina_scaffold_80:1009671-1014859 | -1.0838 | 0.00134598 | clementine0.9_003616m | AT5G38880.1 |  |  |
| 1. clementina_scaffold_8:2323738-2333672 | -1.08325 | 2.42173e-07 | clementine0.9_001343m | AT3G45780.1 | PHOT1 | phototropin 1 |
| 1. clementina_scaffold_80:1024015-1025740 | -1.08236 | 4.39562e-05 | clementine0.9_014637m | AT5G15780.1 |  | Pollen Ole e 1 allergen and extensin family protein |
| 1. clementina_scaffold_113:92915-95628 | -1.08123 | 0.000175782 | clementine0.9_010976m | AT3G15030.1 | TCP4 | TCP family transcription factor 4 |
| 1. clementina_scaffold_16:2211397-2213083 | -1.08085 | 0.000365927 | clementine0.9_007256m | AT4G31940.1 | CYP82C4 | cytochrome P450. family 82. subfamily C. polypeptide 4 |
| 1. clementina_scaffold_18:1573650-1581329 | -1.07778 | 9.37472e-08 | clementine0.9_024797m | AT3G49550.1 |  |  |
| 1. clementina_scaffold_6:3423952-3427734 | -1.07511 | 1.32535e-06 | clementine0.9_034737m | AT5G51690.1 | ACS12 | 1-amino-cyclopropane-1-carboxylate synthase 12 |
| 1. clementina_scaffold_1:12676049-12679815 | -1.07461 | 1.63586e-05 | clementine0.9_000975m | AT1G77680.1 |  | Ribonuclease II/R family protein |
| 1. clementina_scaffold_139:276983-281538 | -1.07349 | 1.11887e-05 | clementine0.9_000849m | AT4G12010.1 |  | Disease resistance protein (TIR-NBS-LRR class) family |
| 1. clementina_scaffold_8:651253-652457 | -1.07203 | 0.00101017 | clementine0.9_016533m | AT5G01410.1 | RSR4 | Aldolase-type TIM barrel family protein |
| 1. clementina_scaffold_72:1127396-1130425 | -1.07144 | 4.05511e-05 | clementine0.9_006214m | AT1G09460.1 |  | Carbohydrate-binding X8 domain superfamily protein |
| 1. clementina_scaffold_4:5437307-5439756 | -1.06964 | 1.01943e-06 | clementine0.9_020008m | AT5G21940.1 |  |  |
| 1. clementina_scaffold_95:182493-190140 | -1.06947 | 1.20202e-09 | clementine0.9_028921m | AT5G13950.3 |  |  |
| 1. clementina_scaffold_7:3271113-3278796 | -1.06785 | 2.82726e-05 | clementine0.9_003286m | AT5G19330.1 | ARIA | ARM repeat protein interacting with ABF2 |
| 1. clementina_scaffold_3:3311345-3316412 | -1.06734 | 8.84873e-07 | clementine0.9_013658m | AT1G09530.1 | PIF3 | phytochrome interacting factor 3 |
| 1. clementina_scaffold_7:5471924-5474534 | -1.06715 | 0.00211508 | clementine0.9_003181m | AT1G10240.1 | FRS11 | FAR1-related sequence 11 |
| 1. clementina_scaffold_7:5523021-5529429 | -1.06678 | 3.92366e-09 | clementine0.9_002914m | AT5G49360.1 | BXL1 | beta-xylosidase 1 |
| 1. clementina_scaffold_5:587304-589289 | -1.06659 | 0.000227548 | clementine0.9_016174m | AT3G27010.1 | TCP20 | TEOSINTE BRANCHED 1. cycloidea. PCF (TCP)-domain family protein 20 |
| 1. clementina_scaffold_10:2460242-2464039 | -1.06609 | 1.31238e-10 | clementine0.9_016294m | AT1G67480.1 |  | Galactose oxidase/kelch repeat superfamily protein |
| 1. clementina_scaffold_103:24455-25196 | -1.06561 | 0.000249951 | clementine0.9_032120m | AT3G04550.1 |  |  |
| 1. clementina_scaffold_4:7696470-7696578 | -1.06413 | 9.64918e-05 | clementine0.9_032838m | AT2G34930.1 |  | disease resistance family protein / LRR family protein |
| 1. clementina_scaffold_25:2998851-3001753 | -1.06222 | 1.27504e-06 | clementine0.9_012211m | AT3G51370.1 |  | Protein phosphatase 2C family protein |
| 1. clementina_scaffold_91:204983-214621 | -1.05715 | 1.90963e-08 | clementine0.9_002518m | AT5G35980.1 | YAK1 | yeast YAK1-related gene 1 |
| 1. clementina_scaffold_4:4555590-4558341 | -1.05672 | 0.000594391 | clementine0.9_001629m | AT2G26330.1 | ER | Leucine-rich receptor-like protein kinase family protein |
| 1. clementina_scaffold_3:340930-346620 | -1.05593 | 1.0922e-06 | clementine0.9_009547m | AT4G00050.1 | UNE10 | basic helix-loop-helix (bHLH) DNA-binding superfamily protein |
| 1. clementina_scaffold_1:11185445-11187198 | -1.0537 | 5.4623e-14 | clementine0.9_023360m | AT4G33580.1 | BCA5 | beta carbonic anhydrase 5 |
| 1. clementina_scaffold_20:2066684-2071963 | -1.05058 | 4.87122e-08 | clementine0.9_002770m | AT5G17920.1 | ATMS1 | Cobalamin-independent synthase family protein |
| 1. clementina_scaffold_4:1656558-1660089 | -1.05053 | 0.000994788 | clementine0.9_001808m | AT4G30825.1 |  | Tetratricopeptide repeat (TPR)-like superfamily protein |
| 1. clementina_scaffold_23:3031813-3035805 | -1.04892 | 0.000308961 | clementine0.9_023614m | AT5G08480.2 |  | VQ motif-containing protein |
| 1. clementina_scaffold_9:990239-991005 | -1.04767 | 0.00121806 | clementine0.9_022942m | AT4G29110.1 |  |  |
| 1. clementina_scaffold_1:10062661-10065714 | -1.04662 | 0.000149674 | clementine0.9_005593m | AT1G49890.1 | QWRF2 | Family of unknown function (DUF566) |
| 1. clementina_scaffold_8:1776592-1779185 | -1.04116 | 0.00153641 | clementine0.9_009675m | AT3G11340.1 |  | UDP-Glycosyltransferase superfamily protein |
| 1. clementina_scaffold_43:1256049-1257583 | -1.03729 | 0.00052428 | clementine0.9_008798m | AT1G05530.1 | UGT75B2 | UDP-glucosyl transferase 75B2 |
| 1. clementina_scaffold_8:2486922-2496108 | -1.03727 | 4.82563e-09 | clementine0.9_001155m | AT3G45630.1 |  | RNA binding (RRM/RBD/RNP motifs) family protein |
| 1. clementina_scaffold_10:1499902-1507614 | -1.03631 | 5.33169e-05 | clementine0.9_003802m | AT1G70610.1 | TAP1 | transporter associated with antigen processing protein 1 |
| 1. clementina_scaffold_12:2811394-2813633 | -1.03439 | 0.00030823 | clementine0.9_015194m | AT3G01470.1 | HB-1 | homeobox 1 |
| 1. clementina_scaffold_2:2505595-2511169 | -1.03415 | 5.30562e-09 | clementine0.9_002044m | AT3G51620.2 |  | PAP/OAS1 substrate-binding domain superfamily |
| 1. clementina_scaffold_3:1724826-1728431 | -1.03354 | 2.16497e-08 | clementine0.9_008792m | AT1G01540.2 |  | Protein kinase superfamily protein |
| 1. clementina_scaffold_91:749618-753463 | -1.03336 | 0.00184987 | clementine0.9_010868m | AT5G17640.1 |  | Protein of unknown function (DUF1005) |
| 1. clementina_scaffold_53:363979-368638 | -1.03295 | 0.000101143 | clementine0.9_003680m | AT5G15410.1 | DND1 | Cyclic nucleotide-regulated ion channel family protein |
| 1. clementina_scaffold_3:5002027-5007409 | -1.03141 | 1.68808e-05 | clementine0.9_033314m | AT2G47830.1 |  | Cation efflux family protein |
| 1. clementina_scaffold_16:4134252-4137935 | -1.03138 | 0.000158737 | clementine0.9_003224m | AT1G07530.1 | SCL14 | SCARECROW-like 14 |
| 1. clementina_scaffold_29:387885-392363 | -1.02443 | 0.00191297 | clementine0.9_002125m | AT2G39260.1 |  | binding;RNA binding |
| 1. clementina_scaffold_23:1554557-1559762 | -1.02405 | 0.000881283 | clementine0.9_016531m | AT5G67370.1 |  | Protein of unknown function (DUF1230) |
| 1. clementina_scaffold_9:2425602-2428898 | -1.02279 | 0.000631578 | clementine0.9_006059m | AT2G26690.1 |  | Major facilitator superfamily protein |
| 1. clementina_scaffold_7:6375599-6379635 | -1.02191 | 0.00228258 | clementine0.9_008253m | AT5G18500.1 |  | Protein kinase superfamily protein |
| 1. clementina_scaffold_2:789559-791588 | -1.02143 | 1.91073e-05 | clementine0.9_006494m | AT2G38080.1 | IRX12 | Laccase/Diphenol oxidase family protein |
| 1. clementina_scaffold_2:5142283-5146891 | -1.02008 | 4.88203e-06 | clementine0.9_014096m | AT5G06260.1 |  | TLD-domain containing nucleolar protein |
| 1. clementina_scaffold_25:1573548-1576208 | -1.01979 | 4.58056e-07 | clementine0.9_030108m |  |  |  |
| 1. clementina_scaffold_7:4451517-4457162 | -1.019 | 4.32943e-12 | clementine0.9_006480m | AT5G18570.1 | EMB269 | GTP1/OBG family protein |
| 1. clementina_scaffold_23:3762684-3768357 | -1.01791 | 4.31223e-05 | clementine0.9_036062m | AT3G21300.1 |  | RNA methyltransferase family protein |
| 1. clementina_scaffold_26:3193822-3195430 | -1.01557 | 0.00108143 |  |  |  |  |
| 1. clementina_scaffold_12:1213863-1218258 | -1.01537 | 0.000126527 | clementine0.9_003709m | AT5G63190.1 |  | MA3 domain-containing protein |
| 1. clementina_scaffold_1:3670635-3675067 | -1.01523 | 4.72893e-06 | clementine0.9_032445m | AT4G19110.1 |  | Protein kinase superfamily protein |
| 1. clementina_scaffold_34:1311226-1314204 | -1.01323 | 0.000116996 | clementine0.9_005316m | AT1G76890.2 | GT2 | Duplicated homeodomain-like superfamily protein |
| 1. clementina_scaffold_15:3938857-3947022 | -1.01218 | 2.58327e-05 | clementine0.9_000078m | AT2G35630.1 | MOR1 | ARM repeat superfamily protein |
| 1. clementina_scaffold_2:134373-136227 | -1.01202 | 0.000844667 | clementine0.9_007027m | AT3G53950.1 |  | glyoxal oxidase-related protein |
| 1. clementina_scaffold_17:4131414-4142427 | -1.01152 | 4.96027e-05 | clementine0.9_029287m | AT2G36810.1 |  | ARM repeat superfamily protein |
| 1. clementina_scaffold_52:815463-820534 | -1.01145 | 0.00 | clementine0.9_015695m | AT1G60600.1 | ABC4 | UbiA prenyltransferase family protein |
| 1. clementina_scaffold_80:566531-569230 | -1.01096 | 0.00161652 | clementine0.9_005285m | AT5G38600.1 |  | Proline-rich spliceosome-associated (PSP) family protein / zinc knuckle (CCHC-type) family protein |
| 1. clementina_scaffold_3:2222745-2229195 | -1.01057 | 1.33216e-07 | clementine0.9_032831m | AT3G61690.1 |  | nucleotidyltransferases |
| 1. clementina_scaffold_22:789900-791487 | -1.01054 | 7.22608e-05 | clementine0.9_021181m | AT4G35190.1 |  | Putative lysine decarboxylase family protein |
| 1. clementina_scaffold_20:3532153-3536761 | -1.01031 | 3.03557e-05 | clementine0.9_010243m | AT5G14420.1 | RGLG2 | RING domain ligase2 |
| 1. clementina_scaffold_23:1598712-1601992 | -1.00748 | 2.90193e-06 | clementine0.9_005089m | AT5G67385.1 |  | Phototropic-responsive NPH3 family protein |
| 1. clementina_scaffold_8:128668-131245 | -1.00716 | 0.0016947 | clementine0.9_011600m | AT3G13062.1 |  | Polyketide cyclase/dehydrase and lipid transport superfamily protein |
| 1. clementina_scaffold_28:433655-434234 | -1.0051 | 0.00101291 | clementine0.9_026575m | AT2G21210.1 |  | SAUR-like auxin-responsive protein family |
| 1. clementina_scaffold_44:1058286-1063232 | -1.00507 | 0.000291558 | clementine0.9_002313m | AT2G21300.1 |  | ATP binding microtubule motor family protein |
| 1. clementina_scaffold_22:1081639-1083230 | -1.00342 | 0.00149619 | clementine0.9_016142m | AT5G42180.1 |  | Peroxidase superfamily protein |
| 1. clementina_scaffold_128:391802-403422 | -1.00236 | 0.000696555 | clementine0.9_000910m | AT5G06600.1 | UBP12 | ubiquitin-specific protease 12 |
| 1. clementina_scaffold_4:1915814-1918081 | -1.00177 | 0.000328561 | clementine0.9_010764m | AT4G30440.1 | GAE1 | UDP-D-glucuronate 4-epimerase 1 |
| 1. clementina_scaffold_146:295954-302000 | -0.997662 | 0.000400702 | clementine0.9_011603m | AT3G48730.1 | GSA2 | glutamate-1-semialdehyde 2.1-aminomutase 2 |
| 1. clementina_scaffold_104:272642-274616 | -0.993868 | 0.00204716 | clementine0.9_025322m | AT3G05870.1 | APC11 | anaphase-promoting complex/cyclosome 11 |
| 1. clementina_scaffold_7:270175-279970 | -0.991066 | 3.09352e-07 | clementine0.9_003450m | AT1G06150.1 | EMB1444 | basic helix-loop-helix (bHLH) DNA-binding superfamily protein |
| 1. clementina_scaffold_7:2721600-2722817 | -0.990846 | 0.00070328 | clementine0.9_018026m | AT5G02750.1 |  | RING/U-box superfamily protein |
| 1. clementina_scaffold_19:1690363-1692585 | -0.98805 | 0.000489325 | clementine0.9_022749m | AT3G14770.1 | SWEET2 | Nodulin MtN3 family protein |
| 1. clementina_scaffold_22:1899047-1903833 | -0.98743 | 0.000297422 |  |  |  |  |
| 1. clementina_scaffold_112:622847-624079 | -0.987082 | 0.000215394 | clementine0.9_027043m | AT4G28088.1 |  | Low temperature and salt responsive protein family |
| 1. clementina_scaffold_9:4431693-4431811 | -0.986907 | 0.000191542 | clementine0.9_020310m | AT4G26220.1 |  | S-adenosyl-L-methionine-dependent methyltransferases superfamily protein |
| 1. clementina_scaffold_60:426277-428580 | -0.985322 | 0.000344225 | clementine0.9_004300m | AT5G10530.1 |  | Concanavalin A-like lectin protein kinase family protein |
| 1. clementina_scaffold_9:2353955-2360047 | -0.985203 | 0.00174731 | clementine0.9_015173m | AT1G15410.1 |  | aspartate-glutamate racemase family |
| 1. clementina_scaffold_20:3075042-3081459 | -0.980238 | 6.76153e-06 | clementine0.9_002884m | AT5G40390.1 | SIP1 | Raffinose synthase family protein |
| 1. clementina_scaffold_17:1737262-1742521 | -0.979033 | 4.08846e-11 | clementine0.9_010982m | AT3G14720.1 | MPK19 | MAP kinase 19 |
| 1. clementina_scaffold_12:4012895-4016296 | -0.976826 | 0.00019077 | clementine0.9_014208m | AT4G01100.1 | ADNT1 | adenine nucleotide transporter 1 |
| 1. clementina_scaffold_8:3542582-3543505 | -0.976792 | 1.32555e-05 | clementine0.9_028735m | AT1G49290.1 |  |  |
| 1. clementina_scaffold_3:6693281-6698476 | -0.976621 | 0.000912956 | clementine0.9_010369m | AT2G30580.1 | DRIP2 | DREB2A-interacting protein 2 |
| 1. clementina_scaffold_29:2401989-2403657 | -0.976569 | 1.54839e-10 | clementine0.9_025567m | AT1G18730.1 | NDF6 | NDH dependent flow 6 |
| 1. clementina_scaffold_12:1245033-1247142 | -0.976082 | 9.33324e-06 | clementine0.9_011921m | AT4G24780.1 |  | Pectin lyase-like superfamily protein |
| 1. clementina_scaffold_16:3186062-3193607 | -0.97394 | 0.000247323 | clementine0.9_003556m | AT5G05680.1 | MOS7 | nuclear pore complex protein-related |
| 1. clementina_scaffold_79:681211-682245 | -0.971573 | 0.000796267 | clementine0.9_019650m | AT5G54680.1 | ILR3 | basic helix-loop-helix (bHLH) DNA-binding superfamily protein |
| 1. clementina_scaffold_1:6242883-6249945 | -0.968562 | 0.000245756 | clementine0.9_001575m | AT1G08720.1 | EDR1 | Protein kinase superfamily protein |
| 1. clementina_scaffold_82:136797-139152 | -0.968337 | 0.000402616 | clementine0.9_013137m | AT4G32060.1 |  | calcium-binding EF hand family protein |
| 1. clementina_scaffold_3:1462349-1470449 | -0.962242 | 3.70023e-05 | clementine0.9_000050m | AT4G00450.1 | CCT | RNA polymerase II transcription mediators |
| 1. clementina_scaffold_82:781308-784299 | -0.961602 | 0.000186641 | clementine0.9_013635m | AT5G11420.1 |  | Protein of unknown function. DUF642 |
| 1. clementina_scaffold_16:2185285-2187230 | -0.960732 | 0.000243467 | clementine0.9_007273m | AT4G31940.1 | CYP82C4 | cytochrome P450. family 82. subfamily C. polypeptide 4 |
| 1. clementina_scaffold_53:352698-357405 | -0.95555 | 2.08513e-05 | clementine0.9_001339m | AT5G15400.1 |  | U-box domain-containing protein |
| 1. clementina_scaffold_9:1370957-1376879 | -0.955211 | 0.000919719 | clementine0.9_004265m | AT2G25970.1 |  | KH domain-containing protein |
| 1. clementina_scaffold_54:774886-777831 | -0.951927 | 0.000568276 | clementine0.9_020006m | AT2G01890.1 | PAP8 | purple acid phosphatase 8 |
| 1. clementina_scaffold_10:4446072-4449606 | -0.951799 | 0.00123565 | clementine0.9_005906m | AT1G68570.1 |  | Major facilitator superfamily protein |
| 1. clementina_scaffold_1:1017929-1021956 | -0.946531 | 7.74244e-05 | clementine0.9_005187m | AT3G20770.1 | EIN3 | Ethylene insensitive 3 family protein |
| 1. clementina_scaffold_83:705051-709476 | -0.946483 | 1.38811e-05 | clementine0.9_008678m | AT1G04680.1 |  | Pectin lyase-like superfamily protein |
| 1. clementina_scaffold_45:1738571-1744431 | -0.946243 | 3.54992e-05 | clementine0.9_036016m | AT2G35840.1 |  | Sucrose-6F-phosphate phosphohydrolase family protein |
| 1. clementina_scaffold_94:243085-247506 | -0.944731 | 0.00013197 | clementine0.9_008636m | AT3G49260.1 | iqd21 | IQ-domain 21 |
| 1. clementina_scaffold_23:1375088-1377073 | -0.944691 | 0.000367796 |  |  |  |  |
| 1. clementina_scaffold_7:4505988-4506706 | -0.942641 | 0.000682588 | clementine0.9_026220m | AT5G18600.1 |  | Thioredoxin superfamily protein |
| 1. clementina_scaffold_9:652142-654595 | -0.94251 | 0.000269308 | clementine0.9_035940m | AT4G28880.1 | ckl3 | casein kinase I-like 3 |
| 1. clementina_scaffold_35:656834-658028 | -0.941973 | 2.43857e-09 | clementine0.9_024394m |  |  |  |
| 1. clementina_scaffold_4:3883839-3887285 | -0.941402 | 0.000359557 | clementine0.9_002789m | AT5G20300.1 |  | Avirulence induced gene (AIG1) family protein |
| 1. clementina_scaffold_9:2474281-2477190 | -0.941389 | 0.000411797 | clementine0.9_014927m | AT3G25910.1 |  | Protein of unknown function (DUF1644) |
| 1. clementina_scaffold_47:1082104-1086882 | -0.940487 | 0.000515263 | clementine0.9_005676m | AT5G16610.1 |  |  |
| 1. clementina_scaffold_10:2941039-2949121 | -0.939024 | 0.000947196 | clementine0.9_000263m | AT1G24300.1 |  | GYF domain-containing protein |
| 1. clementina_scaffold_1:6228439-6230303 | -0.938403 | 0.00155846 | clementine0.9_025020m | AT5G27990.1 |  | Pre-rRNA-processing protein TSR2. conserved region |
| 1. clementina_scaffold_12:4101744-4107467 | -0.938139 | 0.000418396 | clementine0.9_010307m | AT4G23820.1 |  | Pectin lyase-like superfamily protein |
| 1. clementina_scaffold_28:867646-870213 | -0.937941 | 3.05626e-06 | clementine0.9_005826m | AT2G21140.1 | PRP2 | proline-rich protein 2 |
| 1. clementina_scaffold_32:522403-525140 | -0.937823 | 0.000491788 | clementine0.9_010651m | AT5G57020.1 | NMT1 | myristoyl-CoA:protein N-myristoyltransferase |
| 1. clementina_scaffold_20:1993426-2000683 | -0.937106 | 0.00108499 | clementine0.9_019827m | AT3G56850.1 | AREB3 | ABA-responsive element binding protein 3 |
| 1. clementina_scaffold_19:1595337-1600472 | -0.936427 | 6.49311e-07 | clementine0.9_000871m | AT4G18130.1 | PHYE | phytochrome E |
| 1. clementina_scaffold_4:1593806-1597918 | -0.935665 | 0.000280731 | clementine0.9_000813m | AT4G30790.1 |  |  |
| 1. clementina_scaffold_1:9650624-9659845 | -0.934708 | 7.25644e-05 | clementine0.9_003166m | AT4G26090.1 | RPS2 | NB-ARC domain-containing disease resistance protein |
| 1. clementina_scaffold_26:1396452-1397864 | -0.934704 | 0.000528638 |  |  |  |  |
| 1. clementina_scaffold_10:2764906-2767370 | -0.933043 | 0.000150376 | clementine0.9_024325m | AT1G27290.1 |  |  |
| 1. clementina_scaffold_10:3656125-3662010 | -0.932504 | 1.04158e-06 | clementine0.9_000265m | AT1G68890.1 |  | magnesium ion binding;thiamin pyrophosphate binding;hydro-lyases;catalytics;2-succinyl-5-enolpyruvyl-6-hydroxy-3-cyclohexene-1-carboxylic-acid synthases |
| 1. clementina_scaffold_2:2416344-2419653 | -0.93144 | 0.00101538 | clementine0.9_023416m | AT3G51730.1 |  | saposin B domain-containing protein |
| 1. clementina_scaffold_12:4295762-4300154 | -0.930959 | 0.00058188 | clementine0.9_000588m | AT4G10930.1 |  |  |
| 1. clementina_scaffold_7:6211602-6217907 | -0.929514 | 6.82143e-06 | clementine0.9_000818m | AT3G06480.1 |  | DEAD box RNA helicase family protein |
| 1. clementina_scaffold_49:1518559-1523360 | -0.929064 | 4.62042e-11 | clementine0.9_005806m | AT5G35790.1 | G6PD1 | glucose-6-phosphate dehydrogenase 1 |
| 1. clementina_scaffold_34:522095-523258 | -0.928571 | 0.000573488 | clementine0.9_023779m | AT1G21550.1 |  | Calcium-binding EF-hand family protein |
| 1. clementina_scaffold_96:23794-25234 | -0.928325 | 0.00201404 | clementine0.9_020160m | AT3G59780.1 |  | Rhodanese/Cell cycle control phosphatase superfamily protein |
| 1. clementina_scaffold_34:258536-263276 | -0.927865 | 7.23835e-07 | clementine0.9_006038m | AT1G21700.1 | SWI3C | SWITCH/sucrose nonfermenting 3C |
| 1. clementina_scaffold_2:2730105-2733374 | -0.926682 | 0.00202474 | clementine0.9_008978m | AT3G08720.1 | S6K2 | serine/threonine protein kinase 2 |
| 1. clementina_scaffold_33:2287806-2290540 | -0.925151 | 0.000436233 | clementine0.9_007621m | AT3G14360.1 |  | alpha/beta-Hydrolases superfamily protein |
| 1. clementina_scaffold_69:980923-982252 | -0.924822 | 0.000928745 | clementine0.9_019400m | AT4G27450.1 |  | Aluminium induced protein with YGL and LRDR motifs |
| 1. clementina_scaffold_47:1046027-1051325 | -0.923858 | 4.51048e-11 | clementine0.9_021994m | AT5G37720.1 | ALY4 | ALWAYS EARLY 4 |
| 1. clementina_scaffold_105:131655-134960 | -0.922546 | 0.000457632 | clementine0.9_025878m |  |  |  |
| 1. clementina_scaffold_19:703190-704485 | -0.922373 | 0.000913348 | clementine0.9_014517m | AT4G22190.1 |  |  |
| 1. clementina_scaffold_120:302684-307563 | -0.921265 | 6.31745e-10 | clementine0.9_016671m | AT5G37360.1 |  |  |
| 1. clementina_scaffold_24:3184948-3191663 | -0.919996 | 0.00226504 |  |  |  |  |
| 1. clementina_scaffold_4:3441934-3445705 | -0.919777 | 8.26428e-05 | clementine0.9_000984m | AT5G20730.2 | NPH4 | Transcriptional factor B3 family protein / auxin-responsive factor AUX/IAA-related |
| 1. clementina_scaffold_6:6091754-6094590 | -0.919505 | 0.000921287 | clementine0.9_034132m | AT1G14610.1 | TWN2 | valyl-tRNA synthetase / valine--tRNA ligase (VALRS) |
| 1. clementina_scaffold_51:835521-845210 | -0.915845 | 0.00112974 | clementine0.9_006169m | AT3G23980.1 | BLI | BLISTER |
| 1. clementina_scaffold_1:12220952-12227518 | -0.913579 | 1.38673e-06 | clementine0.9_004368m | AT1G77990.1 | AST56 | STAS domain / Sulfate transporter family |
| 1. clementina_scaffold_32:1046116-1051550 | -0.912864 | 0.00288934 | clementine0.9_004304m | AT3G14075.1 |  | Mono-/di-acylglycerol lipase. N-terminal;Lipase. class 3 |
| 1. clementina_scaffold_2:3447726-3454115 | -0.911056 | 3.49637e-05 | clementine0.9_002742m | AT2G32250.1 | FRS2 | FAR1-related sequence 2 |
| 1. clementina_scaffold_7:6491065-6492323 | -0.910626 | 0.00100105 | clementine0.9_017888m | AT1G48300.1 |  |  |
| 1. clementina_scaffold_207:10044-11869 | -0.91034 | 0.00104978 | clementine0.9_009767m | AT1G65450.1 |  | HXXXD-type acyl-transferase family protein |
| 1. clementina_scaffold_4:5131952-5139650 | -0.909051 | 0.000521161 | clementine0.9_006028m | AT1G74960.1 | FAB1 | fatty acid biosynthesis 1 |
| 1. clementina_scaffold_12:4856279-4865340 | -0.908448 | 0.0027568 | clementine0.9_011676m | AT1G32860.1 |  | Glycosyl hydrolase superfamily protein |
| 1. clementina_scaffold_1:7418356-7421043 | -0.908395 | 0.000574356 | clementine0.9_017163m | AT1G54740.1 |  | Protein of unknown function (DUF3049) |
| 1. clementina_scaffold_32:201994-206063 | -0.908235 | 0.000514376 | clementine0.9_008296m | AT3G13730.1 | CYP90D1 | cytochrome P450. family 90. subfamily D. polypeptide 1 |
| 1. clementina_scaffold_3:390148-393884 | -0.906803 | 6.61056e-07 | clementine0.9_005816m |  |  |  |
| 1. clementina_scaffold_1:7445339-7450581 | -0.906264 | 3.7581e-12 | clementine0.9_007659m | AT3G05165.1 |  | Major facilitator superfamily protein |
| 1. clementina_scaffold_1:11864405-11869999 | -0.905592 | 1.36665e-05 | clementine0.9_001791m | AT1G22310.2 | MBD8 | methyl-CPG-binding domain 8 |
| 1. clementina_scaffold_68:915133-925946 | -0.905534 | 6.11395e-10 | clementine0.9_005971m | AT1G15740.1 |  | Leucine-rich repeat family protein |
| 1. clementina_scaffold_3:5059879-5063350 | -0.905335 | 7.35014e-06 | clementine0.9_002283m | AT4G03200.1 |  | catalytics |
| 1. clementina_scaffold_113:380913-385885 | -0.904915 | 0.00137356 | clementine0.9_028564m | AT3G15010.1 |  | RNA-binding (RRM/RBD/RNP motifs) family protein |
| 1. clementina_scaffold_12:2300923-2306227 | -0.903217 | 0.000567009 | clementine0.9_000231m | AT3G48050.1 |  | BAH domain ;TFIIS helical bundle-like domain |
| 1. clementina_scaffold_6:4536509-4541989 | -0.902809 | 6.74319e-05 | clementine0.9_012189m | AT5G23870.3 |  | Pectinacetylesterase family protein |
| 1. clementina_scaffold_7:4359370-4362075 | -0.902224 | 0.00102202 | clementine0.9_006769m | AT5G18670.1 | BMY3 | beta-amylase 3 |
| 1. clementina_scaffold_51:81253-84071 | -0.902078 | 0.00042004 | clementine0.9_006681m | AT3G22670.1 |  | Pentatricopeptide repeat (PPR) superfamily protein |
| 1. clementina_scaffold_5:7312529-7313204 | -0.901348 | 0.00185373 | clementine0.9_026833m | AT4G10270.1 |  | Wound-responsive family protein |
| 1. clementina_scaffold_23:1495212-1496898 | -0.900855 | 0.00180075 |  |  |  |  |
| 1. clementina_scaffold_23:3735077-3739544 | -0.900736 | 0.000320235 |  |  |  |  |
| 1. clementina_scaffold_120:269973-275904 | -0.899887 | 3.81315e-11 | clementine0.9_011457m | AT5G16810.1 |  | Protein kinase superfamily protein |
| 1. clementina_scaffold_4:6683213-6687405 | -0.89955 | 6.83899e-05 | clementine0.9_009450m | AT5G55580.1 |  | Mitochondrial transcription termination factor family protein |
| 1. clementina_scaffold_20:2001918-2008667 | -0.898685 | 2.84959e-08 | clementine0.9_010457m | AT2G26980.4 | CIPK3 | CBL-interacting protein kinase 3 |
| 1. clementina_scaffold_10:3794208-3797101 | -0.898367 | 2.85231e-10 | clementine0.9_014838m | AT3G57030.1 |  | Calcium-dependent phosphotriesterase superfamily protein |
| 1. clementina_scaffold_3:5701583-5706850 | -0.897135 | 2.20345e-06 | clementine0.9_007607m | AT1G03270.1 |  | CBS domain-containing protein with a domain of unknown function (DUF21) |
| 1. clementina_scaffold_85:461262-462176 | -0.897083 | 0.00111716 | clementine0.9_026078m |  |  |  |
| 1. clementina_scaffold_27:1083382-1090063 | -0.89519 | 4.46476e-07 | clementine0.9_000996m | AT5G43630.1 | TZP | zinc knuckle (CCHC-type) family protein |
| 1. clementina_scaffold_29:2620503-2628905 | -0.895059 | 1.98488e-06 | clementine0.9_001014m | AT5G52230.1 | MBD13 | methyl-CPG-binding domain protein 13 |
| 1. clementina_scaffold_70:297165-301830 | -0.893554 | 0.00101421 | clementine0.9_016126m | AT4G09970.1 |  |  |
| 1. clementina_scaffold_27:697031-699359 | -0.8934 | 1.9139e-05 |  |  |  |  |
| 1. clementina_scaffold_32:2238200-2242040 | -0.893245 | 4.71441e-09 | clementine0.9_006912m | AT5G42690.1 |  | Protein of unknown function. DUF547 |
| 1. clementina_scaffold_31:655030-667189 | -0.891467 | 0.000322019 | clementine0.9_007134m | AT1G55250.3 | HUB2 | histone mono-ubiquitination 2 |
| 1. clementina_scaffold_7:5763801-5769751 | -0.88932 | 1.04086e-05 | clementine0.9_002866m | AT3G06620.1 |  | PAS domain-containing protein tyrosine kinase family protein |
| 1. clementina_scaffold_22:2166810-2167976 | -0.88892 | 0.000591215 |  |  |  |  |
| 1. clementina_scaffold_11:4304081-4306788 | -0.888763 | 0.000550132 | clementine0.9_027342m |  |  |  |
| 1. clementina_scaffold_10:3222502-3226525 | -0.887007 | 9.1953e-06 | clementine0.9_018747m | AT1G13700.1 | PGL1 | 6-phosphogluconolactonase 1 |
| 1. clementina_scaffold_47:1421666-1423703 | -0.886097 | 0.00206211 | clementine0.9_021138m | AT1G66480.1 |  | plastid movement impaired 2 |
| 1. clementina_scaffold_8:3193554-3198978 | -0.88604 | 0.000647799 | clementine0.9_003537m | AT2G28350.1 | ARF10 | auxin response factor 10 |
| 1. clementina_scaffold_6:2464814-2466892 | -0.883811 | 0.00195719 | clementine0.9_009342m | AT3G29590.1 | AT5MAT | HXXXD-type acyl-transferase family protein |
| 1. clementina_scaffold_15:2983918-2987763 | -0.881648 | 0.000223231 | clementine0.9_001909m | AT5G37020.1 | ARF8 | auxin response factor 8 |
| 1. clementina_scaffold_4:3275708-3292220 | -0.881516 | 1.53179e-05 | clementine0.9_001065m | AT5G57110.1 | ACA8 | autoinhibited Ca2+ -ATPase. isoform 8 |
| 1. clementina_scaffold_26:1142336-1144472 | -0.88099 | 0.000185523 | clementine0.9_032594m | AT2G20142.1 |  | Toll-Interleukin-Resistance (TIR) domain family protein |
| 1. clementina_scaffold_4:987483-994619 | -0.880733 | 6.41367e-05 | clementine0.9_003041m | AT5G25270.1 |  | Ubiquitin-like superfamily protein |
| 1. clementina_scaffold_15:2688691-2689458 | -0.880379 | 0.00123647 | clementine0.9_025134m | AT4G21870.1 |  | HSP20-like chaperones superfamily protein |
| 1. clementina_scaffold_90:991953-993672 | -0.880378 | 0.000818352 | clementine0.9_012930m | AT2G19810.1 |  | CCCH-type zinc finger family protein |
| 1. clementina_scaffold_53:1085071-1086872 | -0.876761 | 0.000889608 | clementine0.9_026116m |  |  |  |
| 1. clementina_scaffold_2:3240789-3246900 | -0.876604 | 0.00241768 | clementine0.9_011790m | AT3G02690.1 |  | nodulin MtN21 /EamA-like transporter family protein |
| 1. clementina_scaffold_10:153643-155774 | -0.87656 | 5.67843e-05 | clementine0.9_017421m | AT1G69780.1 | ATHB13 | Homeobox-leucine zipper protein family |
| 1. clementina_scaffold_1:10756031-10761146 | -0.871038 | 0.000739288 | clementine0.9_010182m | AT4G24400.1 | CIPK8 | CBL-interacting protein kinase 8 |
| 1. clementina_scaffold_6:4668781-4674060 | -0.870866 | 4.70601e-06 | clementine0.9_004061m | AT1G63700.1 | YDA | Protein kinase superfamily protein |
| 1. clementina_scaffold_23:3016339-3020438 | -0.87069 | 1.95854e-05 | clementine0.9_009833m | AT4G40050.1 |  | Protein of unknown function (DUF3550/UPF0682) |
| 1. clementina_scaffold_7:4290661-4297583 | -0.87045 | 4.02693e-09 | clementine0.9_000011m | AT2G28290.1 | SYD | P-loop containing nucleoside triphosphate hydrolases superfamily protein |
| 1. clementina_scaffold_4:1271950-1277323 | -0.869854 | 0.000163169 | clementine0.9_000714m | AT5G58040.1 | FIP1[V] | homolog of yeast FIP1 [V] |
| 1. clementina_scaffold_20:2391716-2397828 | -0.868908 | 0.000925645 | clementine0.9_005071m | AT5G14880.1 |  | Potassium transporter family protein |
| 1. clementina_scaffold_128:352081-358086 | -0.867416 | 6.57851e-05 | clementine0.9_030353m | AT3G44050.1 |  | P-loop containing nucleoside triphosphate hydrolases superfamily protein |
| 1. clementina_scaffold_11:2453573-2455760 | -0.86727 | 0.00214211 | clementine0.9_001221m | AT5G17680.1 |  | disease resistance protein (TIR-NBS-LRR class). putative |
| 1. clementina_scaffold_121:189822-194460 | -0.866628 | 0.000778374 | clementine0.9_005137m | AT3G60190.1 | DL1E | DYNAMIN-like 1E |
| 1. clementina_scaffold_63:369160-374220 | -0.865667 | 0.000580824 | clementine0.9_004173m | AT4G31390.1 |  | Protein kinase superfamily protein |
| 1. clementina_scaffold_6:4863590-4869098 | -0.865422 | 2.86542e-07 | clementine0.9_000872m | AT5G52530.1 |  | dentin sialophosphoprotein-related |
| 1. clementina_scaffold_4:5026150-5031709 | -0.861447 | 2.53131e-13 | clementine0.9_033873m | AT3G43190.1 | SUS4 | sucrose synthase 4 |
| 1. clementina_scaffold_16:554088-558959 | -0.857237 | 0.0011043 | clementine0.9_002375m | AT2G35800.1 |  | mitochondrial substrate carrier family protein |
| 1. clementina_scaffold_22:613060-626976 | -0.856656 | 0.00157395 |  |  |  |  |
| 1. clementina_scaffold_15:3298437-3302133 | -0.856369 | 0.00248582 | clementine0.9_002251m | AT2G34710.1 | PHB | Homeobox-leucine zipper family protein / lipid-binding START domain-containing protein |
| 1. clementina_scaffold_81:41707-45410 | -0.854677 | 0.000159212 | clementine0.9_005489m | AT5G58350.1 | WNK4 | with no lysine (K) kinase 4 |
| 1. clementina_scaffold_8:2988027-2992985 | -0.854474 | 0.00221866 | clementine0.9_020664m | AT5G60590.2 |  | DHBP synthase RibB-like alpha/beta domain |
| 1. clementina_scaffold_2:4478390-4489232 | -0.854078 | 7.05332e-07 | clementine0.9_000928m | AT5G06600.2 | UBP12 | ubiquitin-specific protease 12 |
| 1. clementina_scaffold_19:2298768-2304515 | -0.852662 | 0.00158431 | clementine0.9_013631m | AT1G29050.1 | TBL38 | TRICHOME BIREFRINGENCE-LIKE 38 |
| 1. clementina_scaffold_151:97174-103947 | -0.851786 | 1.63811e-05 | clementine0.9_000095m | AT1G05570.1 | CALS1 | callose synthase 1 |
| 1. clementina_scaffold_21:1451834-1454457 | -0.851628 | 0.00232923 | clementine0.9_027442m | AT1G11910.1 | APA1 | aspartic proteinase A1 |
| 1. clementina_scaffold_34:62734-65521 | -0.850367 | 2.14426e-07 | clementine0.9_006911m | AT1G44446.1 | CH1 | Pheophorbide a oxygenase family protein with Rieske [2Fe-2S] domain |
| 1. clementina_scaffold_79:564681-573898 | -0.849527 | 7.50769e-08 | clementine0.9_000035m | AT4G15180.1 | SDG2 | SET domain protein 2 |
| 1. clementina_scaffold_44:860768-865107 | -0.849411 | 0.00151576 | clementine0.9_005548m | AT4G38900.2 |  | Basic-leucine zipper (bZIP) transcription factor family protein |
| 1. clementina_scaffold_88:879496-881113 | -0.848185 | 0.00218883 | clementine0.9_009410m | AT1G80310.1 |  | sulfate transmembrane transporters |
| 1. clementina_scaffold_7:6355533-6361951 | -0.847438 | 3.57536e-12 | clementine0.9_001169m | AT2G47070.1 | SPL1 | squamosa promoter binding protein-like 1 |
| 1. clementina_scaffold_44:1711109-1717786 | -0.846657 | 0.000395701 | clementine0.9_009374m | AT2G16405.1 |  | Transducin/WD40 repeat-like superfamily protein |
| 1. clementina_scaffold_16:1189701-1194598 | -0.845663 | 0.00186236 | clementine0.9_002373m | AT3G55270.1 | MKP1 | mitogen-activated protein kinase phosphatase 1 |
| 1. clementina_scaffold_134:46941-50056 | -0.843607 | 0.00255268 | clementine0.9_034279m | AT1G79940.1 | ATERDJ2A | DnaJ / Sec63 Brl domains-containing protein |
| 1. clementina_scaffold_4:1367132-1374285 | -0.843439 | 0.000479203 | clementine0.9_001654m | AT5G57990.1 | UBP23 | ubiquitin-specific protease 23 |
| 1. clementina_scaffold_3:3986823-3993482 | -0.843025 | 1.00377e-05 | clementine0.9_002796m | AT4G02020.1 | SWN | SET domain-containing protein |
| 1. clementina_scaffold_80:1051328-1057859 | -0.84052 | 0.00158084 | clementine0.9_000002m | AT3G02260.1 | BIG | auxin transport protein (BIG) |
| 1. clementina_scaffold_3:2124787-2129674 | -0.840161 | 0.00159728 | clementine0.9_008364m | AT1G01430.1 | TBL25 | TRICHOME BIREFRINGENCE-LIKE 25 |
| 1. clementina_scaffold_7:572516-574469 | -0.839886 | 0.00124468 | clementine0.9_025217m | AT5G02160.1 |  |  |
| 1. clementina_scaffold_15:3140837-3146581 | -0.839684 | 0.00144491 | clementine0.9_035456m | AT1G30450.1 | CCC1 | cation-chloride co-transporter 1 |
| 1. clementina_scaffold_8:471990-481854 | -0.83904 | 2.90498e-08 | clementine0.9_003848m | AT1G07110.1 | F2KP | fructose-2.6-bisphosphatase |
| 1. clementina_scaffold_19:2645730-2653817 | -0.838559 | 2.02192e-06 | clementine0.9_000186m | AT1G28420.1 | HB-1 | homeobox-1 |
| 1. clementina_scaffold_64:1287150-1290654 | -0.836965 | 1.19426e-05 | clementine0.9_001010m | AT4G27220.1 |  | NB-ARC domain-containing disease resistance protein |
| 1. clementina_scaffold_6:3057037-3057883 | -0.836511 | 0.00137044 | clementine0.9_036063m | AT5G62360.1 |  | Plant invertase/pectin methylesterase inhibitor superfamily protein |
| 1. clementina_scaffold_58:1366189-1375584 | -0.835454 | 0.00067996 | clementine0.9_035784m | AT4G29060.1 | emb2726 | elongation factor Ts family protein |
| 1. clementina_scaffold_4:7651248-7656724 | -0.833925 | 0.00238094 | clementine0.9_007655m | AT3G20410.1 | CPK9 | calmodulin-domain protein kinase 9 |
| 1. clementina_scaffold_53:400264-412698 | -0.833871 | 0.000502239 | clementine0.9_007255m | AT3G29290.1 | emb2076 | Pentatricopeptide repeat (PPR) superfamily protein |
| 1. clementina_scaffold_79:739000-744028 | -0.829182 | 3.99938e-06 | clementine0.9_008320m | AT4G15240.1 |  | Protein of unknown function (DUF604) |
| 1. clementina_scaffold_9:1146361-1153257 | -0.828986 | 0.000116784 | clementine0.9_000079m | AT4G32820.1 |  | Tetratricopeptide repeat (TPR)-like superfamily protein |
| 1. clementina_scaffold_2:2168116-2172035 | -0.828839 | 0.00158814 | clementine0.9_000289m | AT2G38440.1 | SCAR2 | SCAR homolog 2 |
| 1. clementina_scaffold_12:5052557-5057446 | -0.828838 | 1.81588e-05 | clementine0.9_002756m | AT1G76890.2 | GT2 | Duplicated homeodomain-like superfamily protein |
| 1. clementina_scaffold_26:3132139-3137688 | -0.828277 | 0.00214655 | clementine0.9_001664m | AT4G35270.1 |  | Plant regulator RWP-RK family protein |
| 1. clementina_scaffold_24:1567671-1572547 | -0.828025 | 0.00196374 |  |  |  |  |
| 1. clementina_scaffold_53:384835-387772 | -0.827987 | 3.26654e-05 | clementine0.9_009952m | AT3G01060.1 |  |  |
| 1. clementina_scaffold_3:2765165-2769793 | -0.826571 | 5.18465e-05 | clementine0.9_010325m | AT1G01140.1 | CIPK9 | CBL-interacting protein kinase 9 |
| 1. clementina_scaffold_16:3781691-3786556 | -0.826389 | 6.71487e-06 | clementine0.9_009059m | AT3G55580.1 |  | Regulator of chromosome condensation (RCC1) family protein |
| 1. clementina_scaffold_9:196989-202296 | -0.826156 | 0.00160612 | clementine0.9_000085m | AT2G25660.1 | emb2410 | embryo defective 2410 |
| 1. clementina_scaffold_17:2392790-2397299 | -0.826048 | 0.000147572 | clementine0.9_001273m | AT1G53440.1 |  | Leucine-rich repeat transmembrane protein kinase |
| 1. clementina_scaffold_67:1220667-1225584 | -0.824828 | 7.71406e-05 | clementine0.9_002742m | AT2G32250.1 | FRS2 | FAR1-related sequence 2 |
| 1. clementina_scaffold_12:1810103-1814612 | -0.824546 | 1.67545e-06 | clementine0.9_000378m | AT4G24680.1 | MOS1 | modifier of snc1 |
| 1. clementina_scaffold_10:300946-306684 | -0.824096 | 0.00218368 | clementine0.9_002193m | AT3G17900.1 |  |  |
| 1. clementina_scaffold_8:120118-126463 | -0.822396 | 0.000206168 | clementine0.9_009719m | AT3G13060.2 | ECT5 | evolutionarily conserved C-terminal region 5 |
| 1. clementina_scaffold_15:3094279-3102705 | -0.820801 | 0.000578464 | clementine0.9_031858m | AT2G34660.1 | MRP2 | multidrug resistance-associated protein 2 |
| 1. clementina_scaffold_8:3983081-3988654 | -0.820322 | 1.72802e-05 | clementine0.9_029271m | AT2G27980.1 |  | Acyl-CoA N-acyltransferase with RING/FYVE/PHD-type zinc finger domain |
| 1. clementina_scaffold_15:3723368-3727757 | -0.819912 | 0.00184543 | clementine0.9_018090m | AT5G44450.1 |  | methyltransferases |
| 1. clementina_scaffold_9:3874380-3879336 | -0.819087 | 0.00108414 | clementine0.9_022856m | AT5G48930.1 | HCT | hydroxycinnamoyl-CoA shikimate/quinate hydroxycinnamoyl transferase |
| 1. clementina_scaffold_25:2303492-2310443 | -0.81877 | 4.55801e-06 | clementine0.9_002218m | AT2G44710.1 |  | RNA-binding (RRM/RBD/RNP motifs) family protein |
| 1. clementina_scaffold_136:169461-175831 | -0.818421 | 0.00236239 | clementine0.9_000012m | AT1G50030.1 | TOR | target of rapamycin |
| 1. clementina_scaffold_38:782344-791916 | -0.817745 | 2.79914e-05 | clementine0.9_008723m | AT5G09300.1 |  | Thiamin diphosphate-binding fold (THDP-binding) superfamily protein |
| 1. clementina_scaffold_12:5042461-5049637 | -0.816601 | 0.000512515 | clementine0.9_013816m | AT4G10430.1 |  | TMPIT-like protein |
| 1. clementina_scaffold_3:2329736-2343143 | -0.816101 | 1.77522e-06 | clementine0.9_000119m | AT1G01320.2 |  | Tetratricopeptide repeat (TPR)-like superfamily protein |
| 1. clementina_scaffold_12:3211708-3215970 | -0.815993 | 0.00030394 | clementine0.9_000799m | AT1G63300.1 |  | Myosin heavy chain-related protein |
| 1. clementina_scaffold_6:3149485-3158535 | -0.815475 | 5.59378e-06 | clementine0.9_027573m | AT4G25290.1 |  | DNA photolyases;DNA photolyases |
| 1. clementina_scaffold_33:2378816-2382260 | -0.814785 | 4.89438e-07 | clementine0.9_005845m | AT3G14310.1 | PME3 | pectin methylesterase 3 |
| 1. clementina_scaffold_2:5935974-5943412 | -0.814631 | 6.85673e-06 | clementine0.9_000448m | AT2G36380.1 | PDR6 | pleiotropic drug resistance 6 |
| 1. clementina_scaffold_98:72294-81549 | -0.814563 | 0.00149938 | clementine0.9_001616m | AT2G06990.1 | HEN2 | RNA helicase. ATP-dependent. SK12/DOB1 protein |
| 1. clementina_scaffold_26:1398063-1405147 | -0.814513 | 3.12789e-06 | clementine0.9_004479m | AT2G14680.1 | MEE13 | myosin heavy chain-related |
| 1. clementina_scaffold_94:9881-13823 | -0.813945 | 0.000765762 | clementine0.9_005431m | AT5G53370.1 | PMEPCRF | pectin methylesterase PCR fragment F |
| 1. clementina_scaffold_4:2360996-2368619 | -0.813551 | 0.000491997 | clementine0.9_002458m | AT5G57590.1 | BIO1 | adenosylmethionine-8-amino-7-oxononanoate transaminases |
| 1. clementina_scaffold_12:4221527-4224255 | -0.81214 | 0.000969051 | clementine0.9_022190m | AT4G23895.1 |  | Pleckstrin homology (PH) domain-containing protein |
| 1. clementina_scaffold_31:1208993-1217286 | -0.811829 | 0.000201642 | clementine0.9_000097m | AT5G13000.1 | GSL12 | glucan synthase-like 12 |
| 1. clementina_scaffold_33:523582-525550 | -0.811824 | 0.00237338 | clementine0.9_005107m | AT3G47570.1 |  | Leucine-rich repeat protein kinase family protein |
| 1. clementina_scaffold_1:12089626-12094965 | -0.811024 | 0.00279817 | clementine0.9_010115m | AT3G13340.1 |  | Transducin/WD40 repeat-like superfamily protein |
| 1. clementina_scaffold_6:262033-268325 | -0.808849 | 2.87764e-06 | clementine0.9_001336m | AT2G41790.1 |  | Insulinase (Peptidase family M16) family protein |
| 1. clementina_scaffold_7:3060758-3065057 | -0.808476 | 0.00107662 | clementine0.9_016469m | AT3G09600.1 |  | Homeodomain-like superfamily protein |
| 1. clementina_scaffold_13:1533851-1536939 | -0.807899 | 0.000850093 | clementine0.9_000715m | AT5G04020.1 |  | calmodulin binding |
| 1. clementina_scaffold_25:459280-464615 | -0.807846 | 0.000160598 | clementine0.9_023228m | AT3G50630.1 | KRP2 | KIP-related protein 2 |
| 1. clementina_scaffold_56:1210878-1216716 | -0.807661 | 0.000449141 | clementine0.9_002068m | AT1G15130.1 |  | Endosomal targeting BRO1-like domain-containing protein |
| 1. clementina_scaffold_55:796286-801766 | -0.80601 | 0.000501211 | clementine0.9_002414m | AT1G80930.1 |  | MIF4G domain-containing protein / MA3 domain-containing protein |
| 1. clementina_scaffold_11:3295243-3297276 | -0.805302 | 0.000311793 | clementine0.9_034441m | AT1G19260.1 |  | TTF-type zinc finger protein with HAT dimerisation domain |
| 1. clementina_scaffold_23:1473576-1476830 | -0.804596 | 0.000796676 | clementine0.9_002441m | AT2G23140.1 |  | RING/U-box superfamily protein with ARM repeat domain |
| 1. clementina_scaffold_139:354138-360671 | -0.804317 | 0.000741302 | clementine0.9_009705m | AT1G73875.1 |  | DNAse I-like superfamily protein |
| 1. clementina_scaffold_44:1082772-1086118 | -0.804129 | 0.000285295 | clementine0.9_022987m | AT4G38960.1 |  | B-box type zinc finger family protein |
| 1. clementina_scaffold_23:2646539-2649197 | -0.80384 | 3.42781e-05 | clementine0.9_013765m | AT4G37970.1 | CAD6 | cinnamyl alcohol dehydrogenase 6 |
| 1. clementina_scaffold_24:1927820-1931801 | -0.803737 | 0.000242652 |  |  |  |  |
| 1. clementina_scaffold_29:921917-926846 | -0.802843 | 3.63872e-05 | clementine0.9_010733m | AT1G73980.1 |  | Phosphoribulokinase / Uridine kinase family |
| 1. clementina_scaffold_21:1469495-1473143 | -0.802037 | 2.12719e-07 | clementine0.9_017404m | AT2G32500.1 |  | Stress responsive alpha-beta barrel domain protein |
| 1. clementina_scaffold_84:186740-190725 | -0.801894 | 0.00101049 | clementine0.9_007564m | AT5G51150.1 |  | Mitochondrial import inner membrane translocase subunit Tim17/Tim22/Tim23 family protein |
| 1. clementina_scaffold_1:9423221-9427907 | -0.800287 | 0.000415501 | clementine0.9_010806m | AT1G09195.2 |  |  |
| 1. clementina_scaffold_3:2922426-2925432 | -0.799907 | 0.000230256 | clementine0.9_011463m | AT3G61950.1 |  | basic helix-loop-helix (bHLH) DNA-binding superfamily protein |
| 1. clementina_scaffold_39:590229-590772 | -0.799432 | 0.00235361 | clementine0.9_026065m | AT1G51400.1 |  | Photosystem II 5 kD protein |
| 1. clementina_scaffold_36:2105461-2112114 | -0.799275 | 0.000321444 | clementine0.9_000725m | AT1G22930.1 |  | T-complex protein 11 |
| 1. clementina_scaffold_2:2316364-2324495 | -0.799173 | 0.00217342 | clementine0.9_000627m | AT3G08850.1 | RAPTOR1 | HEAT repeat ;WD domain. G-beta repeat protein protein |
| 1. clementina_scaffold_3:1263895-1266528 | -0.79796 | 4.77304e-05 | clementine0.9_033771m | AT3G61220.1 |  | NAD(P)-binding Rossmann-fold superfamily protein |
| 1. clementina_scaffold_3:5672466-5678369 | -0.797787 | 9.22339e-06 | clementine0.9_000454m | AT2G48160.1 |  | Tudor/PWWP/MBT domain-containing protein |
| 1. clementina_scaffold_110:209034-212359 | -0.79762 | 2.9894e-07 | clementine0.9_032757m | AT5G07010.1 | ST2A | sulfotransferase 2A |
| 1. clementina_scaffold_10:268963-274651 | -0.797051 | 0.002338 | clementine0.9_003185m | AT1G69830.1 | AMY3 | alpha-amylase-like 3 |
| 1. clementina_scaffold_3:4552454-4555695 | -0.793406 | 0.000208773 | clementine0.9_031143m | AT4G02340.1 |  | alpha/beta-Hydrolases superfamily protein |
| 1. clementina_scaffold_4:5168358-5172929 | -0.792395 | 0.00281549 | clementine0.9_000850m | AT1G19220.1 | ARF19 | auxin response factor 19 |
| 1. clementina_scaffold_3:6384233-6390207 | -0.791597 | 0.00273967 | clementine0.9_007230m | AT1G03060.1 | SPI | Beige/BEACH domain ;WD domain. G-beta repeat protein |
| 1. clementina_scaffold_43:186540-191006 | -0.791545 | 0.000660701 | clementine0.9_002438m | AT2G47390.1 |  | Prolyl oligopeptidase family protein |
| 1. clementina_scaffold_93:479015-504679 | -0.790394 | 9.53336e-06 | clementine0.9_013952m | AT5G08710.1 |  | Regulator of chromosome condensation (RCC1) family protein |
| 1. clementina_scaffold_24:3200841-3205642 | -0.789759 | 1.61053e-05 |  |  |  |  |
| 1. clementina_scaffold_12:4571262-4574919 | -0.789319 | 6.65242e-06 | clementine0.9_003708m | AT4G10760.1 | MTA | mRNAadenosine methylase |
| 1. clementina_scaffold_4:3024751-3031371 | -0.788774 | 8.73996e-07 | clementine0.9_003246m | AT4G30020.1 |  | PA-domain containing subtilase family protein |
| 1. clementina_scaffold_34:1424288-1429933 | -0.786376 | 4.17588e-06 | clementine0.9_023334m |  |  |  |
| 1. clementina_scaffold_3:7765247-7771405 | -0.784416 | 0.00123046 |  |  |  |  |
| 1. clementina_scaffold_22:1217846-1221997 | -0.784033 | 2.43727e-07 | clementine0.9_000638m | AT1G27940.1 | PGP13 | P-glycoprotein 13 |
| 1. clementina_scaffold_39:1219531-1223690 | -0.783498 | 0.000208001 | clementine0.9_017060m | AT1G28100.1 |  |  |
| 1. clementina_scaffold_71:337469-341415 | -0.782795 | 2.3127e-07 | clementine0.9_005318m | AT5G21222.1 |  | protein kinase family protein |
| 1. clementina_scaffold_4:4900075-4903417 | -0.781958 | 1.22181e-05 | clementine0.9_007052m | AT5G20860.1 |  | Plant invertase/pectin methylesterase inhibitor superfamily |
| 1. clementina_scaffold_71:143272-149182 | -0.779035 | 8.83599e-08 | clementine0.9_008631m | AT1G67300.1 |  | Major facilitator superfamily protein |
| 1. clementina_scaffold_136:148643-153001 | -0.778797 | 0.00160757 | clementine0.9_007219m | AT3G18890.1 |  | NAD(P)-binding Rossmann-fold superfamily protein |
| 1. clementina_scaffold_23:1572648-1576176 | -0.77744 | 0.000695086 |  |  |  |  |
| 1. clementina_scaffold_12:1367313-1371521 | -0.777362 | 5.6083e-08 | clementine0.9_009477m | AT5G14420.1 | RGLG2 | RING domain ligase2 |
| 1. clementina_scaffold_4:4187311-4190656 | -0.77656 | 0.000311329 | clementine0.9_006612m | AT1G72770.1 | HAB1 | homology to ABI1 |
| 1. clementina_scaffold_33:1970565-1977821 | -0.769156 | 0.00227607 | clementine0.9_003844m | AT3G42170.1 |  | BED zinc finger ;hAT family dimerisation domain |
| 1. clementina_scaffold_36:444049-446704 | -0.768404 | 9.53303e-05 | clementine0.9_003492m | AT5G43470.1 | RPP8 | Disease resistance protein (CC-NBS-LRR class) family |
| 1. clementina_scaffold_1:9405106-9409031 | -0.767063 | 8.11984e-07 | clementine0.9_007209m | AT3G06720.1 | IMPA-1 | importin alpha isoform 1 |
| 1. clementina_scaffold_39:1828313-1830309 | -0.766017 | 0.00118159 | clementine0.9_019739m | AT1G69530.1 | EXPA1 | expansin A1 |
| 1. clementina_scaffold_16:404013-412728 | -0.764543 | 2.44205e-06 | clementine0.9_000169m | AT3G52250.1 |  | Duplicated homeodomain-like superfamily protein |
| 1. clementina_scaffold_26:1970168-1974876 | -0.762986 | 0.000135296 |  |  |  |  |
| 1. clementina_scaffold_16:544297-550104 | -0.761408 | 0.000132027 | clementine0.9_004300m | AT5G10530.1 |  | Concanavalin A-like lectin protein kinase family protein |
| 1. clementina_scaffold_23:1878443-1883406 | -0.759588 | 0.0016474 | clementine0.9_033178m | AT5G67570.1 | DG1 | Tetratricopeptide repeat (TPR)-like superfamily protein |
| 1. clementina_scaffold_11:1664402-1673093 | -0.759143 | 1.05091e-06 | clementine0.9_001234m | AT4G10120.1 | ATSPS4F | Sucrose-phosphate synthase family protein |
| 1. clementina_scaffold_4:6230296-6233682 | -0.757734 | 0.00270199 | clementine0.9_003499m | AT1G55850.1 | CSLE1 | cellulose synthase like E1 |
| 1. clementina_scaffold_8:2811012-2816869 | -0.754439 | 0.00101611 | clementine0.9_002322m | AT5G60410.3 | SIZ1 | DNA-binding protein with MIZ/SP-RING zinc finger. PHD-finger and SAP domain |
| 1. clementina_scaffold_4:5514120-5518455 | -0.751617 | 0.000248068 | clementine0.9_006525m | AT5G56270.1 | WRKY2 | WRKY DNA-binding protein 2 |
| 1. clementina_scaffold_80:993311-998502 | -0.751498 | 6.99097e-05 | clementine0.9_009052m | AT3G02300.1 |  | Regulator of chromosome condensation (RCC1) family protein |
| 1. clementina_scaffold_7:3014848-3020475 | -0.750755 | 0.000863786 | clementine0.9_002263m | AT5G02830.1 |  | Tetratricopeptide repeat (TPR)-like superfamily protein |
| 1. clementina_scaffold_1:11899040-11903950 | -0.750517 | 2.8576e-06 | clementine0.9_005030m | AT4G08920.1 | CRY1 | cryptochrome 1 |
| 1. clementina_scaffold_12:4401650-4404927 | -0.75021 | 0.000361977 | clementine0.9_001837m | AT4G24020.1 | NLP7 | NIN like protein 7 |
| 1. clementina_scaffold_2:4801058-4807507 | -0.750075 | 0.00014271 | clementine0.9_001854m | AT2G35920.1 |  | RNA helicase family protein |
| 1. clementina_scaffold_85:804449-812804 | -0.748867 | 9.97215e-05 | clementine0.9_011302m | AT3G07300.1 |  | NagB/RpiA/CoA transferase-like superfamily protein |
| 1. clementina_scaffold_28:2132589-2135943 | -0.747949 | 2.97924e-09 | clementine0.9_018461m | AT3G57490.1 |  | Ribosomal protein S5 family protein |
| 1. clementina_scaffold_4:2572298-2575603 | -0.747062 | 0.00241977 | clementine0.9_015313m | AT2G18860.1 |  | Syntaxin/t-SNARE family protein |
| 1. clementina_scaffold_1:12471793-12477277 | -0.746293 | 6.82565e-05 | clementine0.9_008060m | AT4G08500.1 | MEKK1 | MAPK/ERK kinase kinase 1 |
| 1. clementina_scaffold_80:187538-193286 | -0.74384 | 0.000130277 | clementine0.9_011181m | AT5G16150.1 | PGLCT | plastidic GLC translocator |
| 1. clementina_scaffold_34:1726764-1733071 | -0.742045 | 0.000250327 | clementine0.9_033630m | AT1G71696.2 | SOL1 | carboxypeptidase D. putative |
| 1. clementina_scaffold_58:1132404-1137123 | -0.742039 | 0.000848786 | clementine0.9_006703m | AT2G44970.1 |  | alpha/beta-Hydrolases superfamily protein |
| 1. clementina_scaffold_31:1764124-1768258 | -0.741215 | 0.00240363 | clementine0.9_004305m | AT3G20780.1 | TOP6B | topoisomerase 6 subunit B |
| 1. clementina_scaffold_8:3636947-3641140 | -0.740616 | 0.000315367 | clementine0.9_005517m | AT5G60790.1 | GCN1 | ABC transporter family protein |
| 1. clementina_scaffold_1:12459088-12464197 | -0.739789 | 0.000833409 | clementine0.9_005367m | AT1G36990.1 |  |  |
| 1. clementina_scaffold_4:6022942-6028022 | -0.739051 | 0.000846707 | clementine0.9_001450m | AT3G13225.2 |  | WW domain-containing protein |
| 1. clementina_scaffold_23:3169993-3176633 | -0.737081 | 0.000363796 | clementine0.9_008369m | AT5G65380.1 |  | MATE efflux family protein |
| 1. clementina_scaffold_32:2562714-2566778 | -0.736445 | 0.00134389 | clementine0.9_012889m | AT1G75020.1 | LPAT4 | lysophosphatidyl acyltransferase 4 |
| 1. clementina_scaffold_121:65024-68039 | -0.736016 | 0.0012534 | clementine0.9_015623m | AT5G58220.1 | TTL | transthyretin-like protein |
| 1. clementina_scaffold_1:901468-903355 | -0.736012 | 8.03908e-06 | clementine0.9_011452m | AT5G23120.1 | HCF136 | photosystem II stability/assembly factor. chloroplast (HCF136) |
| 1. clementina_scaffold_13:4668522-4679200 | -0.735792 | 0.000136794 | clementine0.9_001449m | AT2G41740.1 | VLN2 | villin 2 |
| 1. clementina_scaffold_55:606556-609566 | -0.735067 | 0.00156665 | clementine0.9_010823m | AT4G34530.1 | CIB1 | cryptochrome-interacting basic-helix-loop-helix 1 |
| 1. clementina_scaffold_9:453673-456877 | -0.734253 | 0.000565561 | clementine0.9_004818m | AT5G25630.1 |  | Tetratricopeptide repeat (TPR)-like superfamily protein |
| 1. clementina_scaffold_54:1253037-1263571 | -0.73363 | 0.000833673 | clementine0.9_023581m | AT1G06470.1 |  | Nucleotide/sugar transporter family protein |
| 1. clementina_scaffold_5:3958901-3973997 | -0.733599 | 1.02773e-05 | clementine0.9_003267m | AT4G03560.1 | TPC1 | two-pore channel 1 |
| 1. clementina_scaffold_17:1270505-1274529 | -0.73331 | 0.000860551 | clementine0.9_007626m | AT3G14690.1 | CYP72A15 | cytochrome P450. family 72. subfamily A. polypeptide 15 |
| 1. clementina_scaffold_25:3039808-3044965 | -0.730556 | 0.000101331 | clementine0.9_000098m | AT3G50370.1 |  |  |
| 1. clementina_scaffold_12:1073250-1077685 | -0.728845 | 2.49353e-10 | clementine0.9_020373m |  |  |  |
| 1. clementina_scaffold_55:1310624-1313366 | -0.727602 | 0.00169073 | clementine0.9_022827m | AT4G36720.1 | HVA22K | HVA22-like protein K |
| 1. clementina_scaffold_106:34184-40092 | -0.72733 | 0.000198995 | clementine0.9_003511m | AT4G19040.1 | EDR2 | ENHANCED DISEASE RESISTANCE 2 |
| 1. clementina_scaffold_42:915187-922794 | -0.725231 | 0.00134707 | clementine0.9_000508m | AT2G36910.1 | ABCB1 | ATP binding cassette subfamily B1 |
| 1. clementina_scaffold_20:1941337-1949859 | -0.723393 | 0.000864678 | clementine0.9_001789m | AT2G27040.1 | ago/04 | Argonaute family protein |
| 1. clementina_scaffold_10:2869731-2875649 | -0.722475 | 0.000991312 | clementine0.9_010520m | AT1G27980.1 | DPL1 | dihydrosphingosine phosphate lyase |
| 1. clementina_scaffold_13:2803889-2812332 | -0.71898 | 0.000378084 | clementine0.9_000279m | AT3G57300.1 | INO80 | INO80 ortholog |
| 1. clementina_scaffold_3:1519387-1524444 | -0.718263 | 2.49846e-05 | clementine0.9_013442m | AT3G61415.1 | SK21 | SKP1-like 21 |
| 1. clementina_scaffold_123:448626-456578 | -0.714658 | 0.00192476 | clementine0.9_003422m | AT1G17980.1 | PAPS1 | poly(A) polymerase 1 |
| 1. clementina_scaffold_47:560778-566987 | -0.713937 | 1.13128e-06 | clementine0.9_001491m | AT2G40840.1 | DPE2 | disproportionating enzyme 2 |
| 1. clementina_scaffold_4:6251532-6260341 | -0.713078 | 0.000217862 | clementine0.9_000005m | AT1G55860.2 | UPL1 | ubiquitin-protein ligase 1 |
| 1. clementina_scaffold_4:3113129-3117051 | -0.712579 | 2.07454e-09 | clementine0.9_012148m | AT4G25990.2 | CIL | CCT motif family protein |
| 1. clementina_scaffold_31:1175937-1186660 | -0.711322 | 0.000376945 | clementine0.9_000102m | AT3G07160.1 | GSL10 | glucan synthase-like 10 |
| 1. clementina_scaffold_85:58671-73554 | -0.710464 | 0.000648051 | clementine0.9_000707m | AT1G33410.2 | SAR1 | SUPPRESSOR OF AUXIN RESISTANCE1 |
| 1. clementina_scaffold_7:148129-157782 | -0.709168 | 2.5591e-06 | clementine0.9_032523m | AT3G55610.1 | P5CS2 | delta 1-pyrroline-5-carboxylate synthase 2 |
| 1. clementina_scaffold_7:4823828-4828147 | -0.708154 | 0.00280296 | clementine0.9_002552m | AT3G16630.1 | KINESIN-13A | P-loop containing nucleoside triphosphate hydrolases superfamily protein |
| 1. clementina_scaffold_56:819059-824502 | -0.707561 | 0.000245243 | clementine0.9_002184m | AT2G01460.1 |  | P-loop containing nucleoside triphosphate hydrolases superfamily protein |
| 1. clementina_scaffold_3:1157115-1163116 | -0.707381 | 0.000200133 | clementine0.9_002366m | AT4G00730.1 | ANL2 | Homeobox-leucine zipper family protein / lipid-binding START domain-containing protein |
| 1. clementina_scaffold_68:777996-783044 | -0.706575 | 0.00105191 | clementine0.9_010024m | AT1G80530.1 |  | Major facilitator superfamily protein |
| 1. clementina_scaffold_10:5473278-5479699 | -0.705527 | 7.90702e-06 | clementine0.9_004355m | AT3G19830.1 | NTMC2T5.2 | Calcium-dependent lipid-binding (CaLB domain) family protein |
| 1. clementina_scaffold_6:3933553-3938785 | -0.703912 | 4.0684e-05 | clementine0.9_019667m | AT5G51970.1 |  | GroES-like zinc-binding alcohol dehydrogenase family protein |
| 1. clementina_scaffold_46:618791-629975 | -0.703315 | 0.0015669 | clementine0.9_001592m | AT2G43410.1 | FPA | RNA binding |
| 1. clementina_scaffold_22:528270-531592 | -0.701757 | 0.000626954 | clementine0.9_008467m | AT2G01860.1 | EMB975 | Tetratricopeptide repeat (TPR)-like superfamily protein |
| 1. clementina_scaffold_34:1445085-1452493 | -0.699339 | 0.00118956 | clementine0.9_001006m | AT2G21770.1 | CESA9 | cellulose synthase A9 |
| 1. clementina_scaffold_3:3417215-3419008 | -0.697739 | 0.00200636 | clementine0.9_010939m | AT4G01840.1 | KCO5 | Ca2+ activated outward rectifying K+ channel 5 |
| 1. clementina_scaffold_105:211231-214899 | -0.6954 | 1.0562e-05 | clementine0.9_017292m |  |  |  |
| 1. clementina_scaffold_1:10049085-10052426 | -0.694773 | 1.17072e-06 | clementine0.9_026514m | AT5G43150.1 |  |  |
| 1. clementina_scaffold_32:1340984-1346008 | -0.694478 | 0.000554845 | clementine0.9_035277m | AT3G14470.1 |  | NB-ARC domain-containing disease resistance protein |
| 1. clementina_scaffold_12:3816753-3821361 | -0.694143 | 0.00107874 | clementine0.9_004059m | AT1G64140.1 |  |  |
| 1. clementina_scaffold_19:2753035-2755015 | -0.693841 | 0.00167899 | clementine0.9_026762m | AT2G33830.2 |  | Dormancy/auxin associated family protein |
| 1. clementina_scaffold_6:483334-488656 | -0.692988 | 0.00151647 | clementine0.9_002956m | AT3G57520.1 | SIP2 | seed imbibition 2 |
| 1. clementina_scaffold_47:291907-297177 | -0.692749 | 4.02514e-05 | clementine0.9_009124m | AT2G41060.1 |  | RNA-binding (RRM/RBD/RNP motifs) family protein |
| 1. clementina_scaffold_4:4176517-4183507 | -0.692696 | 5.48039e-06 | clementine0.9_006549m | AT4G14350.1 |  | AGC (cAMP-dependent. cGMP-dependent and protein kinase C) kinase family protein |
| 1. clementina_scaffold_21:2472234-2475915 | -0.691459 | 0.00178969 | clementine0.9_004846m | AT2G32540.1 | CSLB04 | cellulose synthase-like B4 |
| 1. clementina_scaffold_7:6205170-6209457 | -0.691323 | 0.000556763 | clementine0.9_008788m | AT1G48110.1 | ECT7 | evolutionarily conserved C-terminal region 7 |
| 1. clementina_scaffold_3:5025893-5035129 | -0.690928 | 0.000184577 | clementine0.9_000196m | AT3G62900.1 |  | CW-type Zinc Finger |
| 1. clementina_scaffold_1:6214522-6223827 | -0.689961 | 0.00276089 | clementine0.9_000227m | AT5G27970.1 |  | ARM repeat superfamily protein |
| 1. clementina_scaffold_3:5408007-5414745 | -0.688394 | 4.57003e-07 | clementine0.9_001399m | AT1G03380.1 | ATG18G | homolog of yeast autophagy 18 (ATG18) G |
| 1. clementina_scaffold_23:1651384-1657835 | -0.687921 | 1.12568e-05 | clementine0.9_010252m | AT4G37560.1 |  | Acetamidase/Formamidase family protein |
| 1. clementina_scaffold_22:1415235-1419607 | -0.687285 | 0.00225731 | clementine0.9_003160m | AT1G75730.1 |  |  |
| 1. clementina_scaffold_11:3760440-3766646 | -0.686809 | 3.81061e-09 | clementine0.9_027511m | AT4G10070.1 |  | KH domain-containing protein |
| 1. clementina_scaffold_85:113389-120215 | -0.686544 | 0.00221864 | clementine0.9_003872m | AT5G49730.1 | FRO6 | ferric reduction oxidase 6 |
| 1. clementina_scaffold_1:11501229-11506366 | -0.686401 | 0.000882848 | clementine0.9_014473m | AT2G36690.1 |  | 2-oxoglutarate (2OG) and Fe(II)-dependent oxygenase superfamily protein |
| 1. clementina_scaffold_8:2417117-2421842 | -0.686135 | 4.7929e-06 | clementine0.9_010384m | AT4G30950.1 | FAD6 | fatty acid desaturase 6 |
| 1. clementina_scaffold_35:1557581-1569110 | -0.685245 | 0.000171382 | clementine0.9_011788m | AT3G02830.1 | ZFN1 | zinc finger protein 1 |
| 1. clementina_scaffold_47:623567-628079 | -0.684641 | 1.4815e-07 | clementine0.9_010373m | AT3G25230.1 | ROF1 | rotamase FKBP 1 |
| 1. clementina_scaffold_60:1128460-1134614 | -0.684582 | 7.09771e-06 | clementine0.9_011057m | AT1G05055.1 | GTF2H2 | general transcription factor II H2 |
| 1. clementina_scaffold_3:2217923-2221885 | -0.684273 | 2.16417e-05 | clementine0.9_032831m | AT3G61690.1 |  | nucleotidyltransferases |
| 1. clementina_scaffold_12:4237091-4242186 | -0.68421 | 8.43769e-15 | clementine0.9_005277m | AT1G64430.1 |  | Pentatricopeptide repeat (PPR) superfamily protein |
| 1. clementina_scaffold_10:5786597-5791529 | -0.679761 | 0.00285515 | clementine0.9_006644m | AT5G18480.1 | PGSIP6 | plant glycogenin-like starch initiation protein 6 |
| 1. clementina_scaffold_131:276428-279145 | -0.679388 | 0.00156286 | clementine0.9_024809m | AT1G32540.2 | LOL1 | lsd one like 1 |
| 1. clementina_scaffold_26:2659185-2667663 | -0.679361 | 1.27221e-07 |  |  |  |  |
| 1. clementina_scaffold_156:128534-134075 | -0.678801 | 1.63691e-05 | clementine0.9_002887m | AT5G26742.2 | emb1138 | DEAD box RNA helicase (RH3) |
| 1. clementina_scaffold_7:5632837-5638679 | -0.675169 | 0.00252966 | clementine0.9_000359m | AT5G49430.1 |  | WD40/YVTN repeat-like-containing domain;Bromodomain |
| 1. clementina_scaffold_36:855107-863157 | -0.673293 | 0.000734164 | clementine0.9_003525m | AT5G08720.1 |  |  |
| 1. clementina_scaffold_4:4822585-4826284 | -0.672604 | 0.000891965 | clementine0.9_014673m | AT4G33140.1 |  | Haloacid dehalogenase-like hydrolase (HAD) superfamily protein |
| 1. clementina_scaffold_19:234626-243272 | -0.672313 | 0.00107022 | clementine0.9_000290m | AT5G47690.3 |  | binding |
| 1. clementina_scaffold_4:1378693-1382192 | -0.668946 | 0.0016548 | clementine0.9_002238m | AT2G24230.1 |  | Leucine-rich repeat protein kinase family protein |
| 1. clementina_scaffold_32:2571680-2577926 | -0.667247 | 0.000154885 | clementine0.9_002198m | AT5G42620.2 |  | metalloendopeptidases;zinc ion binding |
| 1. clementina_scaffold_3:3458608-3464481 | -0.667048 | 2.80312e-06 | clementine0.9_003596m | AT3G17450.1 |  | hAT dimerisation domain-containing protein |
| 1. clementina_scaffold_130:268891-276995 | -0.666886 | 0.00106602 | clementine0.9_003732m | AT1G53050.1 |  | Protein kinase superfamily protein |
| 1. clementina_scaffold_15:3114907-3119621 | -0.666579 | 0.00261794 | clementine0.9_031858m | AT2G34660.1 | MRP2 | multidrug resistance-associated protein 2 |
| 1. clementina_scaffold_25:3353096-3358040 | -0.663058 | 0.000203816 | clementine0.9_005677m | AT2G17650.1 |  | AMP-dependent synthetase and ligase family protein |
| 1. clementina_scaffold_26:3371374-3384210 | -0.662372 | 7.50869e-05 | clementine0.9_017467m | AT3G12500.1 | HCHIB | basic chitinase |
| 1. clementina_scaffold_21:2603797-2613425 | -0.660543 | 3.33346e-09 | clementine0.9_019889m | AT3G58760.1 |  | Integrin-linked protein kinase family |
| 1. clementina_scaffold_40:118142-122904 | -0.658536 | 0.000187749 | clementine0.9_007670m | AT5G56850.1 |  |  |
| 1. clementina_scaffold_25:2176567-2183110 | -0.657513 | 0.000333762 | clementine0.9_000251m | AT3G51120.1 |  | DNA binding;zinc ion binding;nucleic acid binding;nucleic acid binding |
| 1. clementina_scaffold_27:3254607-3257889 | -0.657104 | 7.20059e-05 | clementine0.9_016582m | AT1G60000.1 |  | RNA-binding (RRM/RBD/RNP motifs) family protein |
| 1. clementina_scaffold_1:12192081-12196204 | -0.656934 | 0.00247514 | clementine0.9_000048m | AT1G36160.1 | ACC1 | acetyl-CoA carboxylase 1 |
| 1. clementina_scaffold_22:1092826-1095082 | -0.654945 | 0.000380377 |  |  |  |  |
| 1. clementina_scaffold_27:3259458-3268219 | -0.654612 | 8.31859e-05 | clementine0.9_011309m | AT1G04530.1 |  | Tetratricopeptide repeat (TPR)-like superfamily protein |
| 1. clementina_scaffold_23:379639-384248 | -0.654453 | 0.000517392 | clementine0.9_008361m | AT5G27730.1 |  | Protein of unknown function (DUF1624) |
| 1. clementina_scaffold_9:536624-543512 | -0.653937 | 0.000746374 | clementine0.9_004163m | AT4G32760.1 |  | ENTH/VHS/GAT family protein |
| 1. clementina_scaffold_22:447878-451353 | -0.653825 | 0.000210678 | clementine0.9_001885m | AT5G24360.1 | IRE1-1 | inositol requiring 1-1 |
| 1. clementina_scaffold_3:6354098-6360295 | -0.652771 | 0.000134271 | clementine0.9_009594m | AT5G19840.2 |  | 2-oxoglutarate (2OG) and Fe(II)-dependent oxygenase superfamily protein |
| 1. clementina_scaffold_15:2263469-2274794 | -0.652732 | 0.00047815 | clementine0.9_003645m | AT3G54500.1 |  |  |
| 1. clementina_scaffold_22:3913009-3919296 | -0.65188 | 7.2828e-06 |  |  |  |  |
| 1. clementina_scaffold_9:242679-248586 | -0.651533 | 0.000466085 | clementine0.9_031487m | AT3G24480.1 |  | Leucine-rich repeat (LRR) family protein |
| 1. clementina_scaffold_22:1225808-1229447 | -0.650663 | 0.00185566 | clementine0.9_021324m | AT1G20070.1 |  |  |
| 1. clementina_scaffold_95:172799-181816 | -0.650527 | 6.69418e-05 | clementine0.9_014413m | AT3G19340.1 |  | Protein of unknown function (DUF3754) |
| 1. clementina_scaffold_8:2239121-2243717 | -0.650216 | 8.02874e-05 | clementine0.9_001127m | AT3G45850.1 |  | P-loop containing nucleoside triphosphate hydrolases superfamily protein |
| 1. clementina_scaffold_23:2806671-2815773 | -0.649993 | 0.000336027 | clementine0.9_003539m | AT5G10490.1 | MSL2 | MSCS-like 2 |
| 1. clementina_scaffold_14:2670254-2673887 | -0.647765 | 0.00191612 | clementine0.9_013774m | AT2G43230.2 |  | Protein kinase superfamily protein |
| 1. clementina_scaffold_23:3447585-3454964 | -0.64746 | 0.00055559 | clementine0.9_005649m | AT5G08500.1 |  | Transmembrane CLPTM1 family protein |
| 1. clementina_scaffold_10:4406792-4418064 | -0.647434 | 3.95706e-05 | clementine0.9_002011m | AT3G25840.1 |  | Protein kinase superfamily protein |
| 1. clementina_scaffold_3:1598829-1604120 | -0.647174 | 0.00211849 | clementine0.9_000093m | AT2G46020.2 | BRM | transcription regulatory protein SNF2. putative |
| 1. clementina_scaffold_2:4678883-4684162 | -0.647013 | 9.71789e-05 | clementine0.9_004487m | AT5G06440.2 |  |  |
| 1. clementina_scaffold_1:9234729-9238634 | -0.64413 | 9.18986e-05 | clementine0.9_004724m | AT5G27920.1 |  | F-box family protein |
| 1. clementina_scaffold_119:283112-286883 | -0.64292 | 0.00111214 | clementine0.9_016683m | AT4G33580.1 | BCA5 | beta carbonic anhydrase 5 |
| 1. clementina_scaffold_16:841226-850306 | -0.639986 | 8.28237e-05 | clementine0.9_002149m | AT3G55020.1 |  | Ypt/Rab-GAP domain of gyp1p superfamily protein |
| 1. clementina_scaffold_38:625606-630835 | -0.639001 | 0.00234173 | clementine0.9_000461m | AT5G20960.1 | AO1 | aldehyde oxidase 1 |
| 1. clementina_scaffold_4:1074010-1078073 | -0.638839 | 0.000689329 | clementine0.9_003283m | AT3G09850.1 |  | D111/G-patch domain-containing protein |
| 1. clementina_scaffold_6:5934028-5944736 | -0.638396 | 0.00023893 | clementine0.9_004328m | AT5G53450.1 | ORG1 | OBP3-responsive gene 1 |
| 1. clementina_scaffold_4:5940178-5946279 | -0.637078 | 0.00198845 | clementine0.9_007684m | AT1G55810.1 | UKL3 | uridine kinase-like 3 |
| 1. clementina_scaffold_2:5276252-5284879 | -0.636369 | 0.00125327 | clementine0.9_001781m | AT3G11540.1 | SPY | Tetratricopeptide repeat (TPR)-like superfamily protein |
| 1. clementina_scaffold_3:7525197-7534854 | -0.634317 | 3.84226e-12 |  |  |  |  |
| 1. clementina_scaffold_6:157081-160556 | -0.633972 | 6.31078e-06 | clementine0.9_001882m | AT3G47570.1 |  | Leucine-rich repeat protein kinase family protein |
| 1. clementina_scaffold_71:155029-159472 | -0.631396 | 0.00253423 | clementine0.9_013086m | AT4G28990.1 |  | RNA-binding protein-related |
| 1. clementina_scaffold_53:260287-263159 | -0.631296 | 0.00237503 | clementine0.9_012172m | AT2G03470.1 |  | ELM2 domain-containing protein |
| 1. clementina_scaffold_1:1340110-1345846 | -0.630775 | 0.000112064 | clementine0.9_010873m | AT4G19120.1 | ERD3 | S-adenosyl-L-methionine-dependent methyltransferases superfamily protein |
| 1. clementina_scaffold_49:1147855-1149727 | -0.629303 | 0.000893899 | clementine0.9_024236m | AT2G04240.1 | XERICO | RING/U-box superfamily protein |
| 1. clementina_scaffold_96:138364-154933 | -0.628178 | 1.80769e-08 | clementine0.9_001931m | AT5G49960.1 |  |  |
| 1. clementina_scaffold_6:211538-221601 | -0.628145 | 5.8344e-09 | clementine0.9_001544m | AT2G41790.1 |  | Insulinase (Peptidase family M16) family protein |
| 1. clementina_scaffold_7:4875352-4881576 | -0.627993 | 5.17736e-05 | clementine0.9_000511m | AT3G06880.2 |  | Transducin/WD40 repeat-like superfamily protein |
| 1. clementina_scaffold_128:64749-70207 | -0.627234 | 0.000487628 | clementine0.9_010506m | AT3G22190.1 | IQD5 | IQ-domain 5 |
| 1. clementina_scaffold_20:3927837-3934187 | -0.626298 | 0.000186025 | clementine0.9_000879m | AT5G44180.2 |  | Homeodomain-like transcriptional regulator |
| 1. clementina_scaffold_80:618943-622809 | -0.625143 | 0.0020244 | clementine0.9_017230m | AT1G67170.1 |  |  |
| 1. clementina_scaffold_8:1916859-1921203 | -0.62201 | 4.39896e-06 | clementine0.9_011275m | AT2G39570.1 |  | ACT domain-containing protein |
| 1. clementina_scaffold_22:1622065-1627485 | -0.62163 | 0.000718948 |  |  |  |  |
| 1. clementina_scaffold_3:3464757-3468321 | -0.621412 | 0.000279718 | clementine0.9_004854m | AT3G62200.1 |  | Putative endonuclease or glycosyl hydrolase |
| 1. clementina_scaffold_17:488600-496268 | -0.620598 | 0.00173388 | clementine0.9_004493m | AT3G14400.1 | UBP25 | ubiquitin-specific protease 25 |
| 1. clementina_scaffold_12:2612087-2615401 | -0.618501 | 0.00104613 | clementine0.9_023209m | AT5G62610.1 |  | basic helix-loop-helix (bHLH) DNA-binding superfamily protein |
| 1. clementina_scaffold_14:3576932-3582193 | -0.618017 | 0.000542113 | clementine0.9_015887m | AT5G05100.1 |  | Single-stranded nucleic acid binding R3H protein |
| 1. clementina_scaffold_4:2680904-2685959 | -0.616067 | 4.79196e-05 | clementine0.9_003663m | AT4G30200.3 | VEL1 | vernalization5/VIN3-like |
| 1. clementina_scaffold_43:1804056-1809543 | -0.615933 | 0.00252593 | clementine0.9_006380m | AT3G06980.1 |  | DEA(D/H)-box RNA helicase family protein |
| 1. clementina_scaffold_78:659251-666305 | -0.615474 | 0.00126008 | clementine0.9_000466m | AT5G13530.1 | KEG | protein kinases;ubiquitin-protein ligases |
| 1. clementina_scaffold_16:1826998-1833432 | -0.613865 | 0.000750812 | clementine0.9_000899m | AT3G14172.1 |  |  |
| 1. clementina_scaffold_2:2311423-2316136 | -0.613354 | 3.22379e-05 | clementine0.9_005110m | AT5G01760.1 |  | ENTH/VHS/GAT family protein |
| 1. clementina_scaffold_4:1103710-1115979 | -0.612693 | 3.26103e-05 | clementine0.9_003145m | AT5G35180.2 |  | Protein of unknown function (DUF1336) |
| 1. clementina_scaffold_3:3148655-3156067 | -0.609777 | 0.000627021 | clementine0.9_018270m | AT2G46910.1 |  | Plastid-lipid associated protein PAP / fibrillin family protein |
| 1. clementina_scaffold_37:1674650-1681181 | -0.609508 | 8.43902e-07 | clementine0.9_028947m | AT3G14470.1 |  | NB-ARC domain-containing disease resistance protein |
| 1. clementina_scaffold_12:4556914-4560508 | -0.609217 | 3.54239e-05 | clementine0.9_005131m | AT5G41990.1 | WNK8 | with no lysine (K) kinase 8 |
| 1. clementina_scaffold_8:52892-59099 | -0.608955 | 0.00105617 | clementine0.9_005522m | AT1G16720.1 | HCF173 | high chlorophyll fluorescence phenotype 173 |
| 1. clementina_scaffold_8:960437-965439 | -0.608746 | 3.83017e-05 | clementine0.9_002940m | AT1G07380.1 |  | Neutral/alkaline non-lysosomal ceramidase |
| 1. clementina_scaffold_34:479386-483710 | -0.607573 | 0.000917062 | clementine0.9_002987m | AT1G77280.1 |  | Protein kinase protein with adenine nucleotide alpha hydrolases-like domain |
| 1. clementina_scaffold_79:904240-909025 | -0.607128 | 0.000330491 | clementine0.9_011509m | AT3G21820.1 | ATXR2 | histone-lysine N-methyltransferase ATXR2 |
| 1. clementina_scaffold_2:2733507-2736593 | -0.605004 | 5.54868e-05 | clementine0.9_030133m | AT5G01920.1 | STN8 | Protein kinase superfamily protein |
| 1. clementina_scaffold_20:1468873-1470852 | -0.602676 | 0.000254965 | clementine0.9_021950m | AT4G29070.1 |  | Phospholipase A2 family protein |
| 1. clementina_scaffold_2:7431743-7439641 | -0.602376 | 4.82585e-06 | clementine0.9_000333m | AT5G47490.1 |  | RGPR-related |
| 1. clementina_scaffold_10:3118115-3124195 | -0.601824 | 1.32584e-05 | clementine0.9_029046m | AT1G13750.1 |  | Purple acid phosphatases superfamily protein |
| 1. clementina_scaffold_68:904437-913563 | -0.601281 | 0.000172832 | clementine0.9_000827m | AT1G15750.1 | TPL | Transducin family protein / WD-40 repeat family protein |
| 1. clementina_scaffold_4:7135500-7139635 | -0.599448 | 0.000566545 | clementine0.9_002116m | AT5G55100.2 |  | SWAP (Suppressor-of-White-APricot)/surp domain-containing protein |
| 1. clementina_scaffold_29:338209-343940 | -0.599195 | 7.21042e-05 | clementine0.9_009551m | AT3G18500.3 |  | DNAse I-like superfamily protein |
| 1. clementina_scaffold_29:2336950-2339895 | -0.596921 | 0.00264906 | clementine0.9_021622m | AT1G74410.1 |  | RING/U-box superfamily protein |
| 1. clementina_scaffold_27:717524-720545 | -0.596768 | 0.00120924 |  |  |  |  |
| 1. clementina_scaffold_1:7438766-7444646 | -0.595671 | 0.00101182 | clementine0.9_031979m | AT5G27350.1 | SFP1 | Major facilitator superfamily protein |
| 1. clementina_scaffold_1:3724400-3737334 | -0.595301 | 0.00151916 | clementine0.9_001417m | AT3G23640.1 | HGL1 | heteroglycan glucosidase 1 |
| 1. clementina_scaffold_3:1390706-1393194 | -0.592753 | 0.00225751 | clementine0.9_013747m | AT3G61320.1 |  | Bestrophin-like protein |
| 1. clementina_scaffold_18:427209-432638 | -0.592629 | 8.46011e-05 | clementine0.9_017349m | AT3G49430.2 | SRp34a | SER/ARG-rich protein 34A |
| 1. clementina_scaffold_102:62705-70233 | -0.592036 | 7.44439e-05 | clementine0.9_008396m | AT3G18000.1 | XPL1 | S-adenosyl-L-methionine-dependent methyltransferases superfamily protein |
| 1. clementina_scaffold_3:4637111-4641967 | -0.591593 | 1.59129e-05 | clementine0.9_006765m | AT2G47600.1 | MHX | magnesium/proton exchanger |
| 1. clementina_scaffold_7:2907202-2911848 | -0.590851 | 6.91043e-06 | clementine0.9_000778m | AT4G26090.1 | RPS2 | NB-ARC domain-containing disease resistance protein |
| 1. clementina_scaffold_11:742264-745645 | -0.590442 | 0.000824096 | clementine0.9_004288m | AT5G17680.1 |  | disease resistance protein (TIR-NBS-LRR class). putative |
| 1. clementina_scaffold_44:1589398-1594968 | -0.58982 | 0.00038491 | clementine0.9_000683m | AT1G62020.1 |  | Coatomer. alpha subunit |
| 1. clementina_scaffold_47:613573-616615 | -0.589796 | 0.00127834 | clementine0.9_010373m | AT3G25230.1 | ROF1 | rotamase FKBP 1 |
| 1. clementina_scaffold_34:929837-937871 | -0.589582 | 0.00142888 | clementine0.9_001160m | AT4G08350.1 | GTA2 | global transcription factor group A2 |
| 1. clementina_scaffold_3:5894090-5899483 | -0.589305 | 0.000101269 | clementine0.9_029132m | AT1G03160.1 | FZL | FZO-like |
| 1. clementina_scaffold_67:213844-219283 | -0.587544 | 0.00213702 | clementine0.9_000659m | AT3G18290.1 | BTS | zinc finger protein-related |
| 1. clementina_scaffold_22:97125-104478 | -0.587495 | 1.86282e-07 | clementine0.9_000619m | AT5G42390.1 |  | Insulinase (Peptidase family M16) family protein |
| 1. clementina_scaffold_25:2409313-2417487 | -0.586615 | 0.0021877 |  |  |  |  |
| 1. clementina_scaffold_10:4513838-4518460 | -0.584651 | 0.000442155 | clementine0.9_002017m | AT1G68560.1 | XYL1 | alpha-xylosidase 1 |
| 1. clementina_scaffold_7:4752966-4757125 | -0.5846 | 0.00080186 | clementine0.9_007633m | AT3G16560.1 |  | Protein phosphatase 2C family protein |
| 1. clementina_scaffold_4:5475524-5484373 | -0.584147 | 0.000320843 | clementine0.9_003287m | AT5G56290.1 | PEX5 | peroxin 5 |
| 1. clementina_scaffold_15:2632749-2639494 | -0.584144 | 0.00201965 | clementine0.9_000528m | AT1G61850.2 |  | phospholipases;galactolipases |
| 1. clementina_scaffold_53:1331566-1335903 | -0.583767 | 0.00165687 | clementine0.9_000554m | AT5G15020.2 | SNL2 | SIN3-like 2 |
| 1. clementina_scaffold_14:4746695-4756343 | -0.583648 | 0.00211235 | clementine0.9_002728m | AT5G04810.1 |  | pentatricopeptide (PPR) repeat-containing protein |
| 1. clementina_scaffold_3:2905113-2910478 | -0.582528 | 5.31692e-06 | clementine0.9_009736m | AT2G46670.1 |  | CCT motif family protein |
| 1. clementina_scaffold_124:474248-484598 | -0.581766 | 0.00148678 | clementine0.9_001549m | AT2G42600.1 | PPC2 | phosphoenolpyruvate carboxylase 2 |
| 1. clementina_scaffold_3:2785055-2789885 | -0.580323 | 4.6348e-05 | clementine0.9_008965m | AT4G00830.1 |  | RNA-binding (RRM/RBD/RNP motifs) family protein |
| 1. clementina_scaffold_3:394721-397142 | -0.579767 | 0.000194421 | clementine0.9_023372m | AT1G12244.1 |  | Polynucleotidyl transferase. ribonuclease H-like superfamily protein |
| 1. clementina_scaffold_34:1300422-1303697 | -0.578392 | 0.000421547 | clementine0.9_005040m | AT1G76880.1 |  | Duplicated homeodomain-like superfamily protein |
| 1. clementina_scaffold_7:5924488-5927408 | -0.570311 | 0.000206055 | clementine0.9_023058m | AT1G02380.1 |  |  |
| 1. clementina_scaffold_7:6513238-6516307 | -0.569395 | 0.00140026 | clementine0.9_021901m | AT5G09995.2 |  |  |
| 1. clementina_scaffold_1:1025194-1029171 | -0.569229 | 0.00211683 | clementine0.9_005238m | AT3G20770.1 | EIN3 | Ethylene insensitive 3 family protein |
| 1. clementina_scaffold_3:3186148-3198571 | -0.56837 | 0.00157739 | clementine0.9_000633m | AT3G62010.1 |  |  |
| 1. clementina_scaffold_4:4922088-4930702 | -0.565927 | 0.000405488 | clementine0.9_000178m | AT3G14270.1 | FAB1B | phosphatidylinositol-4-phosphate 5-kinase family protein |
| 1. clementina_scaffold_23:2139907-2144900 | -0.563688 | 0.000302743 | clementine0.9_000937m | AT5G65770.1 | LINC4 | little nuclei4 |
| 1. clementina_scaffold_33:1672725-1679630 | -0.562748 | 0.000966385 | clementine0.9_002669m | AT3G16290.1 | EMB2083 | AAA-type ATPase family protein |
| 1. clementina_scaffold_29:2583827-2590200 | -0.561439 | 0.00049334 | clementine0.9_003138m | AT1G12800.1 |  | Nucleic acid-binding. OB-fold-like protein |
| 1. clementina_scaffold_56:868569-881251 | -0.561022 | 7.05931e-05 | clementine0.9_031655m | AT2G01440.1 |  | DEAD/DEAH box RNA helicase family protein |
| 1. clementina_scaffold_3:8652483-8665514 | -0.556711 | 0.000440633 | clementine0.9_004686m | AT3G03710.1 | RIF10 | polyribonucleotide nucleotidyltransferase. putative |
| 1. clementina_scaffold_12:1405015-1408741 | -0.556683 | 6.49789e-07 | clementine0.9_010689m | AT4G24740.1 | FC2 | FUS3-complementing gene 2 |
| 1. clementina_scaffold_4:1669135-1675293 | -0.555542 | 0.00287989 | clementine0.9_003914m | AT4G30720.1 |  | FAD/NAD(P)-binding oxidoreductase family protein |
| 1. clementina_scaffold_48:382687-387134 | -0.554378 | 0.00035235 | clementine0.9_028482m | AT3G55080.1 |  | SET domain-containing protein |
| 1. clementina_scaffold_84:439983-448351 | -0.554293 | 0.00043832 | clementine0.9_001246m | AT5G07350.1 | Tudor1 | TUDOR-SN protein 1 |
| 1. clementina_scaffold_18:1496283-1503834 | -0.553127 | 0.0016968 | clementine0.9_002305m | AT5G54090.1 |  | DNA mismatch repair protein MutS. type 2 |
| 1. clementina_scaffold_53:554385-558719 | -0.550964 | 0.000235445 | clementine0.9_007446m | AT5G15270.1 |  | RNA-binding KH domain-containing protein |
| 1. clementina_scaffold_56:133516-138236 | -0.547529 | 0.0022401 | clementine0.9_005167m | AT1G68060.1 | MAP70-1 | microtubule-associated proteins 70-1 |
| 1. clementina_scaffold_9:1473602-1477706 | -0.542709 | 0.00155177 | clementine0.9_019175m | AT4G33000.1 | CBL10 | calcineurin B-like protein 10 |
| 1. clementina_scaffold_6:2578167-2582965 | -0.54008 | 0.000211126 | clementine0.9_014729m | AT5G51410.1 |  | LUC7 N_terminus domain-containing protein |
| 1. clementina_scaffold_7:1173518-1179781 | -0.535575 | 0.00199501 | clementine0.9_031069m | AT5G02310.1 | PRT6 | proteolysis 6 |
| 1. clementina_scaffold_16:918166-924048 | -0.53483 | 0.00243528 | clementine0.9_028520m | AT5G13590.1 |  |  |
| 1. clementina_scaffold_43:89524-92102 | -0.530112 | 0.000449346 | clementine0.9_021361m | AT2G44065.1 |  | Ribosomal protein L2 family |
| 1. clementina_scaffold_26:2620427-2624216 | -0.528609 | 2.35578e-05 | clementine0.9_010271m | AT4G14210.1 | PDS3 | phytoene desaturase 3 |
| 1. clementina_scaffold_84:400024-405516 | -0.523963 | 0.000387707 | clementine0.9_001513m | AT5G51060.1 | RHD2 | NADPH/respiratory burst oxidase protein D |
| 1. clementina_scaffold_2:1419783-1421923 | -0.523442 | 0.000314281 | clementine0.9_016534m | AT5G01410.1 | RSR4 | Aldolase-type TIM barrel family protein |
| 1. clementina_scaffold_7:4354732-4359073 | -0.521424 | 1.90061e-05 | clementine0.9_012087m | AT2G47900.1 | TLP3 | tubby like protein 3 |
| 1. clementina_scaffold_8:2830865-2836689 | -0.518059 | 0.00112154 | clementine0.9_003726m | AT5G60450.1 | ARF4 | auxin response factor 4 |
| 1. clementina_scaffold_3:7826558-7832421 | -0.51685 | 7.75781e-05 | clementine0.9_001410m | AT4G28760.1 |  | Protein of unknown function (DUF3741) |
| 1. clementina_scaffold_65:478909-481533 | -0.506734 | 0.00017509 | clementine0.9_004872m | AT5G13400.1 |  | Major facilitator superfamily protein |
| 1. clementina_scaffold_19:2734214-2738946 | -0.506613 | 0.00270927 | clementine0.9_005052m | AT1G28340.1 | RLP4 | receptor like protein 4 |
| 1. clementina_scaffold_6:6694031-6699849 | -0.502991 | 0.00276111 | clementine0.9_010015m | AT1G54350.1 |  | ABC transporter family protein |
| 1. clementina_scaffold_6:989949-995239 | -0.501606 | 0.00238703 | clementine0.9_004705m | AT3G20860.1 | NEK5 | NIMA-related kinase 5 |
| 1. clementina_scaffold_20:2551629-2555468 | -0.501397 | 0.000194862 | clementine0.9_010542m | AT3G01480.1 | CYP38 | cyclophilin 38 |
| 1. clementina_scaffold_2:4861458-4865792 | -0.500427 | 0.000686663 | clementine0.9_003949m | AT2G35940.1 | BLH1 | BEL1-like homeodomain 1 |
| 1. clementina_scaffold_21:2668746-2675909 | -0.499198 | 0.000346313 | clementine0.9_021967m | AT5G48545.1 | HINT3 | histidine triad nucleotide-binding 3 |
| 1. clementina_scaffold_26:1516819-1523119 | -0.498213 | 0.00159295 | clementine0.9_019288m | AT3G19640.1 | MGT4 | magnesium transporter 4 |
| 1. clementina_scaffold_1:1396719-1405135 | -0.497034 | 0.00070483 | clementine0.9_032445m | AT4G19110.1 |  | Protein kinase superfamily protein |
| 1. clementina_scaffold_7:1431337-1434178 | -0.496351 | 0.000184858 | clementine0.9_020293m |  |  |  |
| 1. clementina_scaffold_20:2880370-2887371 | -0.491171 | 0.000428865 | clementine0.9_003798m | AT5G14610.2 |  | DEAD box RNA helicase family protein |
| 1. clementina_scaffold_21:61889-64540 | -0.490162 | 0.0007106 |  |  |  |  |
| 1. clementina_scaffold_4:627220-633592 | -0.48545 | 0.00253347 | clementine0.9_001008m | AT1G09620.1 |  | ATP binding;leucine-tRNA ligases;aminoacyl-tRNA ligases;nucleotide binding;ATP binding;aminoacyl-tRNA ligases |
| 1. clementina_scaffold_3:2250522-2255268 | -0.483642 | 1.32235e-05 | clementine0.9_017028m | AT2G46420.1 |  | Plant protein 1589 of unknown function |
| 1. clementina_scaffold_25:1802523-1806540 | -0.481676 | 0.00188949 | clementine0.9_019450m | AT5G66470.1 |  | RNA binding;GTP binding |
| 1. clementina_scaffold_133:468209-473478 | -0.481033 | 0.00248833 | clementine0.9_001334m | AT2G32730.1 |  | 26S proteasome regulatory complex. non-ATPase subcomplex. Rpn2/Psmd1 subunit |
| 1. clementina_scaffold_20:113231-119643 | -0.480184 | 7.06433e-05 | clementine0.9_004414m | AT5G19620.1 | OEP80 | outer envelope protein of 80 kDa |
| 1. clementina_scaffold_1:3198930-3206458 | -0.479033 | 0.000374818 | clementine0.9_011690m | AT1G32230.1 | RCD1 | WWE protein-protein interaction domain protein family |
| 1. clementina_scaffold_2:3393465-3396953 | -0.477906 | 0.000290782 | clementine0.9_008497m | AT4G14605.1 |  | Mitochondrial transcription termination factor family protein |
| 1. clementina_scaffold_1:11843251-11848951 | -0.474406 | 0.000802016 | clementine0.9_003898m | AT1G78240.1 | TSD2 | S-adenosyl-L-methionine-dependent methyltransferases superfamily protein |
| 1. clementina_scaffold_25:2946843-2951793 | -0.47425 | 0.00213148 | clementine0.9_011278m | AT4G35920.1 | MCA1 | PLAC8 family protein |
| 1. clementina_scaffold_33:1941818-1947478 | -0.473854 | 0.00155571 | clementine0.9_004146m | AT4G32850.5 | nPAP | nuclear poly(a) polymerase |
| 1. clementina_scaffold_81:670926-678856 | -0.471958 | 0.00211488 | clementine0.9_034047m | AT1G06950.1 | TIC110 | translocon at the inner envelope membrane of chloroplasts 110 |
| 1. clementina_scaffold_8:2353043-2359719 | -0.46578 | 0.000137848 | clementine0.9_003609m | AT5G02810.1 | PRR7 | pseudo-response regulator 7 |
| 1. clementina_scaffold_1:7431890-7437803 | -0.454232 | 0.000764419 | clementine0.9_008722m | AT3G05165.1 |  | Major facilitator superfamily protein |
| 1. clementina_scaffold_9:1291619-1296356 | -0.453787 | 0.000161141 | clementine0.9_002259m | AT3G49601.1 |  |  |
| 1. clementina_scaffold_30:2458139-2465303 | -0.452981 | 0.000837753 | clementine0.9_000243m | AT5G04140.1 | GLU1 | glutamate synthase 1 |
| 1. clementina_scaffold_4:2623963-2629635 | -0.452797 | 0.000884817 | clementine0.9_003654m | AT4G30210.2 | ATR2 | P450 reductase 2 |
| 1. clementina_scaffold_150:153823-157682 | -0.450287 | 0.00184556 | clementine0.9_011731m | AT4G17830.1 |  | Peptidase M20/M25/M40 family protein |
| 1. clementina_scaffold_22:2178912-2182064 | -0.449391 | 0.000845557 |  |  |  |  |
| 1. clementina_scaffold_18:470833-474341 | -0.446507 | 0.000850082 | clementine0.9_012848m | AT1G64720.1 | CP5 | Polyketide cyclase/dehydrase and lipid transport superfamily protein |
| 1. clementina_scaffold_90:596188-598812 | -0.443444 | 0.0011083 | clementine0.9_020310m | AT4G26220.1 |  | S-adenosyl-L-methionine-dependent methyltransferases superfamily protein |
| 1. clementina_scaffold_10:3598759-3603180 | -0.437515 | 0.00208058 | clementine0.9_003992m | AT1G68910.2 | WIT2 | WPP domain-interacting protein 2 |
| 1. clementina_scaffold_137:86426-92187 | -0.433673 | 0.00214712 | clementine0.9_008629m | AT2G20900.2 | DGK5 | diacylglycerol kinase 5 |
| 1. clementina_scaffold_32:764136-766716 | -0.431347 | 0.00118787 | clementine0.9_016745m | AT1G72640.1 |  | NAD(P)-binding Rossmann-fold superfamily protein |
| 1. clementina_scaffold_25:1385626-1390988 | -0.428549 | 0.000111074 | clementine0.9_005650m | AT4G36690.1 | ATU2AF65A | U2 snRNP auxilliary factor. large subunit. splicing factor |
| 1. clementina_scaffold_9:3620774-3628323 | -0.420789 | 0.00218279 | clementine0.9_016066m | AT1G73060.1 | LPA3 | Low PSII Accumulation 3 |
| 1. clementina_scaffold_7:192197-194303 | -0.416737 | 0.00220011 | clementine0.9_015945m | AT2G37770.2 |  | NAD(P)-linked oxidoreductase superfamily protein |
| 1. clementina_scaffold_1:9791237-9795499 | -0.394241 | 0.000721842 | clementine0.9_013487m | AT1G09340.1 | CRB | chloroplast RNA binding |
| 1. clementina_scaffold_49:292757-296642 | -0.387833 | 0.000782159 | clementine0.9_005800m | AT5G48385.1 |  | FRIGIDA-like protein |
| 1. clementina_scaffold_9:3854179-3862931 | -0.383234 | 0.000278538 | clementine0.9_026922m |  |  |  |
| 1. clementina_scaffold_8:836868-841947 | -0.370609 | 0.00280594 | clementine0.9_005391m | AT3G47000.1 |  | Glycosyl hydrolase family protein |
| 1. clementina_scaffold_3:6682342-6686003 | -0.366083 | 0.000829965 | clementine0.9_017168m | AT5G09690.2 | MGT7 | magnesium transporter 7 |
| 1. clementina_scaffold_63:66829-70252 | -0.347496 | 0.00208527 | clementine0.9_020091m | AT4G31115.1 |  | Protein of unknown function (DUF1997) |

*The fold change values (P ≤ 0.001) obtained from of each treated sample compared to Etanol control.

**Identification number of *Citrus clementina* transcripts present in the locus - http://www.phytozome.org/search.php

***Identification number of the *Arabidopsis thaliana* ortholog of down-regulated citrus gene in response to SA treatment (The Arabidopsis Genome Initiative).
