# Supplementary material for: Transcriptional profile of sweet orange in response to chitosan and salicylic acid
Source: BMC Genomics. 2015 Apr 12;16(1):288. doi: 10.1186/s12864-015-1440-5 (PMC4415254; doi:10.1186/s12864-015-1440-5)
Supplement: Additional file 7: Table S6-1. — Differentially expressed genes that were upregulated in CHI-treated plants. [file 12864_2015_1440_MOESM7_ESM.docx]

**Table S6-1** Differentially expressed genes that were up-regulated in CHI-treated plants.

| **Locus** | **Log2**  **(fold_change)*** | **p_value** | **ID**** | ***Arabidopsis thaliana****** | **Gene** | **Description** |
| --- | --- | --- | --- | --- | --- | --- |
| 1. clementina_scaffold_94:494005-496091 | 498.942 | 1.90E+00 | clementine0.9_002941m | AT1G15290.1 |  | Tetratricopeptide repeat (TPR)-like superfamily protein |
| 1. clementina_scaffold_13:3509916-3510940 | 395.692 | 2.78E+00 | clementine0.9_011520m | AT2G41640.1 |  | Glycosyltransferase family 61 protein |
| 1. clementina_scaffold_3:5722564-5723552 | 383.047 | 1.79E-04 | clementine0.9_014388m | AT3G63110.1 | IPT3 | isopentenyltransferase 3 |
| 1. clementina_scaffold_4:6361177-6366835 | 365.907 | 0.0010215 | clementine0.9_004530m | AT4G26630.1 |  | DEK domain-containing chromatin associated protein |
| 1. clementina_scaffold_3:6371007-6373562 | 308.233 | 2.84E-08 | clementine0.9_033817m | AT1G03060.1 | SPI | Beige/BEACH domain ;WD domain. G-beta repeat protein |
| 1. clementina_scaffold_56:199085-199564 | 306.431 | 0 | clementine0.9_030644m | AT5G50220.1 |  | F-box family protein |
| 1. clementina_scaffold_3:3143825-3145081 | 294.736 | 4.39E-01 | clementine0.9_008987m | AT1G18010.1 |  | Major facilitator superfamily protein |
| 1. clementina_scaffold_60:1028067-1028691 | 290.583 | 0.000343068 | clementine0.9_002758m | AT5G58450.1 |  | Tetratricopeptide repeat (TPR)-like superfamily protein |
| 1. clementina_scaffold_32:1542469-1542876 | 273.443 | 0.000602427 | clementine0.9_023796m | AT1G35430.1 |  |  |
| 1. clementina_scaffold_34:2264118-2265429 | 270.787 | 2.66E-01 | clementine0.9_008182m | AT2G44020.1 |  | Mitochondrial transcription termination factor family protein |
| 1. clementina_scaffold_28:1526826-1527791 | 262.073 | 0.00024613 | clementine0.9_035476m | AT1G18265.1 |  | Protein of unknown function. DUF593 |
| 1. clementina_scaffold_3:2277515-2282266 | 257.716 | 0.000104038 | clementine0.9_007661m | AT3G61710.1 | ATG6 | AUTOPHAGY 6 |
| 1. clementina_scaffold_4:3363847-3365496 | 254.381 | 0.00063971 | clementine0.9_034647m | AT2G39930.1 | ISA1 | isoamylase 1 |
| 1. clementina_scaffold_19:245245-246595 | 237.771 | 6.35E-02 | clementine0.9_000215m | AT5G47690.3 |  | binding |
| 1. clementina_scaffold_2:2494069-2497399 | 237.728 | 2.33E+00 | clementine0.9_005216m | AT3G51630.1 | WNK5 | with no lysine (K) kinase 5 |
| 1. clementina_scaffold_7:4056379-4057223 | 236.112 | 0.000166679 | clementine0.9_033410m | AT2G20790.1 |  | clathrin adaptor complexes medium subunit family protein |
| 1. clementina_scaffold_39:1241689-1243483 | 230.718 | 0.000717235 | clementine0.9_000665m | AT2G33540.1 | CPL3 | C-terminal domain phosphatase-like 3 |
| 1. clementina_scaffold_83:303572-304323 | 227.574 | 0.00166741 | clementine0.9_000192m | AT3G48770.1 |  | DNA binding;ATP binding |
| 1. clementina_scaffold_47:943314-943387 | 220.466 | 0.000111088 | clementine0.9_026474m | AT1G72020.1 |  |  |
| 1. clementina_scaffold_2:6700415-6702985 | 219.543 | 1.68E-03 | clementine0.9_025093m | AT1G12390.1 |  | Cornichon family protein |
| 1. clementina_scaffold_2:3874675-3875723 | 215.208 | 0.00148021 | clementine0.9_007414m | AT3G11980.1 | MS2 | Jojoba acyl CoA reductase-related male sterility protein |
| 1. clementina_scaffold_1:6919073-6921932 | 214.623 | 0.000172358 | clementine0.9_022905m | AT1G08845.2 |  | Ribosomal L18p/L5e family protein |
| 1. clementina_scaffold_75:262887-264298 | 210.084 | 0 | clementine0.9_003425m | AT1G76140.1 |  | Prolyl oligopeptidase family protein |
| 1. clementina_scaffold_2:5544772-5546524 | 206.136 | 1.82E-01 | clementine0.9_026362m | AT3G52730.1 |  | ubiquinol-cytochrome C reductase UQCRX/QCR9-like family protein |
| 1. clementina_scaffold_100:816764-818826 | 202.836 | 4.53E-05 | clementine0.9_012490m |  |  |  |
| 1. clementina_scaffold_16:4269243-4270188 | 201.111 | 4.36E-05 | clementine0.9_027390m | ATCG00640.1 | RPL33 | ribosomal protein L33 |
| 1. clementina_scaffold_45:570077-575754 | 199.934 | 0.00110424 | clementine0.9_006634m | AT5G46840.1 |  | RNA-binding (RRM/RBD/RNP motifs) family protein |
| 1. clementina_scaffold_29:1125172-1126120 | 191.106 | 0.0012382 | clementine0.9_008017m | AT5G15740.1 |  | O-fucosyltransferase family protein |
| 1. clementina_scaffold_66:1159160-1159277 | 190.723 | 0 | clementine0.9_035745m | AT3G28890.1 | RLP43 | receptor like protein 43 |
| 1. clementina_scaffold_116:113008-117976 | 190.586 | 0.000321295 | clementine0.9_017398m | AT4G16100.1 |  | Protein of unknown function (DUF789) |
| 1. clementina_scaffold_98:381878-385151 | 189.669 | 0.00154075 | clementine0.9_018230m | AT1G08530.1 |  |  |
| 1. clementina_scaffold_3:5093129-5093784 | 188.927 | 5.39E+00 | clementine0.9_027783m | AT1G03020.1 |  | Thioredoxin superfamily protein |
| 1. clementina_scaffold_44:184160-185480 | 184.797 | 0.000590424 | clementine0.9_034560m | AT2G19680.1 |  | Mitochondrial ATP synthase subunit G protein |
| 1. clementina_scaffold_144:252818-253937 | 179.971 | 1.11E-10 | clementine0.9_021335m | AT1G17860.1 |  | Kunitz family trypsin and protease inhibitor protein |
| 1. clementina_scaffold_1:8766524-8768697 | 177.024 | 1.11E+00 | clementine0.9_002437m | AT1G09060.3 |  | Zinc finger. RING-type;Transcription factor jumonji/aspartyl beta-hydroxylase |
| 1. clementina_scaffold_7:5487449-5489185 | 175.711 | 0.000532214 | clementine0.9_015096m | AT5G49330.1 | MYB111 | myb domain protein 111 |
| 1. clementina_scaffold_53:1407971-1408951 | 175.379 | 0.000154636 | clementine0.9_000119m | AT1G01320.2 |  | Tetratricopeptide repeat (TPR)-like superfamily protein |
| 1. clementina_scaffold_92:256722-257656 | 173.174 | 0.000700045 | clementine0.9_028981m | AT1G35710.1 |  | Protein kinase family protein with leucine-rich repeat domain |
| 1. clementina_scaffold_11:935270-937751 | 167.252 | 0.00103042 | clementine0.9_024632m | AT4G10300.1 |  | RmlC-like cupins superfamily protein |
| 1. clementina_scaffold_53:303513-305512 | 166.886 | 0.000236002 | clementine0.9_023676m | AT5G15350.1 | ENODL17 | early nodulin-like protein 17 |
| 1. clementina_scaffold_76:1140051-1141102 | 162.134 | 0.000178949 | clementine0.9_032715m | AT2G01810.1 |  | RING/FYVE/PHD zinc finger superfamily protein |
| 1. clementina_scaffold_20:2888293-2889717 | 158.431 | 1.03E-03 | clementine0.9_014656m | AT3G49340.1 |  | Cysteine proteinases superfamily protein |
| 1. clementina_scaffold_25:1268702-1269844 | 158.317 | 0.000915668 | clementine0.9_008439m | AT3G50740.1 | UGT72E1 | UDP-glucosyl transferase 72E1 |
| 1. clementina_scaffold_25:1834465-1835911 | 158.087 | 0.000706317 | clementine0.9_014708m | AT3G11480.1 | BSMT1 | S-adenosyl-L-methionine-dependent methyltransferases superfamily protein |
| 1. clementina_scaffold_4:2789062-2791348 | 157.905 | 9.23E+00 | clementine0.9_020720m | AT4G25910.1 | NFU3 | NFU domain protein 3 |
| 1. clementina_scaffold_29:2049394-2050290 | 157.409 | 0.000442897 | clementine0.9_025307m |  |  |  |
| 1. clementina_scaffold_23:1563526-1565172 | 153.972 | 0.00138083 | clementine0.9_032813m | AT4G33910.1 |  | 2-oxoglutarate (2OG) and Fe(II)-dependent oxygenase superfamily protein |
| 1. clementina_scaffold_132:256564-256857 | 152.863 | 2.07E+00 | clementine0.9_026001m | AT3G44590.1 |  | 60S acidic ribosomal protein family |
| 1. clementina_scaffold_82:704216-706183 | 150.682 | 0.000136869 | clementine0.9_019299m | AT1G52700.1 |  | alpha/beta-Hydrolases superfamily protein |
| 1. clementina_scaffold_67:1122040-1126652 | 150.432 | 7.11E-02 | clementine0.9_009279m | AT4G20070.1 | AAH | allantoate amidohydrolase |
| 1. clementina_scaffold_3:8580370-8584051 | 146.699 | 6.08E-07 | clementine0.9_035959m | AT3G14470.1 |  | NB-ARC domain-containing disease resistance protein |
| 1. clementina_scaffold_10:3210806-3212672 | 146.223 | 0.00065203 | clementine0.9_028712m | AT1G26500.1 |  | Pentatricopeptide repeat (PPR) superfamily protein |
| 1. clementina_scaffold_9:4514150-4515581 | 143.892 | 4.15E+00 | clementine0.9_007762m | AT5G07990.1 | TT7 | Cytochrome P450 superfamily protein |
| 1. clementina_scaffold_20:4021552-4024487 | 143.509 | 0.000402366 | clementine0.9_020296m | AT4G28510.1 | PHB1 | prohibitin 1 |
| 1. clementina_scaffold_8:2606539-2608318 | 143.261 | 0.000415314 | clementine0.9_024894m | AT5G02960.1 |  | Ribosomal protein S12/S23 family protein |
| 1. clementina_scaffold_23:3469565-3470970 | 142.572 | 0.000344267 | clementine0.9_009387m | AT4G09570.1 | CPK4 | calcium-dependent protein kinase 4 |
| 1. clementina_scaffold_79:525156-525814 | 138.247 | 0.00103043 | clementine0.9_028446m | AT3G49950.1 |  | GRAS family transcription factor |
| 1. clementina_scaffold_2:630762-631879 | 136.075 | 2.26E-02 | clementine0.9_022857m |  |  |  |
| 1. clementina_scaffold_2:6863909-6866324 | 135.944 | 0.00016011 | clementine0.9_007258m | AT4G12300.1 | CYP706A4 | cytochrome P450. family 706. subfamily A. polypeptide 4 |
| 1. clementina_scaffold_1:1284914-1286824 | 134.986 | 5.36E+00 | clementine0.9_007657m | AT4G31500.1 | CYP83B1 | cytochrome P450. family 83. subfamily B. polypeptide 1 |
| 1. clementina_scaffold_8:4836476-4837728 | 134.598 | 0.000575717 | clementine0.9_002076m | AT4G08850.1 |  | Leucine-rich repeat receptor-like protein kinase family protein |
| 1. clementina_scaffold_39:80148-80499 | 133.607 | 0.00124509 | clementine0.9_019193m | AT3G62870.1 |  | Ribosomal protein L7Ae/L30e/S12e/Gadd45 family protein |
| 1. clementina_scaffold_12:2464076-2466468 | 133.106 | 0 | clementine0.9_022368m | AT3G48100.1 | RR5 | response regulator 5 |
| 1. clementina_scaffold_131:171845-173372 | 131.778 | 0.000623964 | clementine0.9_014457m | AT5G46590.1 | NAC096 | NAC domain containing protein 96 |
| 1. clementina_scaffold_13:3937-5500 | 130.453 | 2.69E-01 | clementine0.9_020037m | AT3G57040.1 | ARR9 | response regulator 9 |
| 1. clementina_scaffold_2:6509238-6511069 | 127.897 | 1.05E-01 | clementine0.9_018490m | AT1G12520.1 | CCS | copper chaperone for SOD1 |
| 1. clementina_scaffold_56:117139-121470 | 127.469 | 0.000521477 | clementine0.9_005046m | AT1G14830.1 | DL1C | DYNAMIN-like 1C |
| 1. clementina_scaffold_3:5108781-5109505 | 126.784 | 1.08E-01 | clementine0.9_026296m | AT2G30540.1 |  | Thioredoxin superfamily protein |
| 1. clementina_scaffold_62:1178693-1179246 | 123.921 | 0.000625772 | clementine0.9_002380m | AT1G65800.1 | RK2 | receptor kinase 2 |
| 1. clementina_scaffold_1:1809149-1810601 | 123.022 | 1.20E-04 | clementine0.9_022372m | AT3G49340.1 |  | Cysteine proteinases superfamily protein |
| 1. clementina_scaffold_29:390814-392306 | 122.923 | 2.29E+00 | clementine0.9_002125m | AT2G39260.1 |  | binding;RNA binding |
| 1. clementina_scaffold_15:4289956-4290381 | 121.806 | 0.00144916 | clementine0.9_002968m | AT1G10180.1 |  |  |
| 1. clementina_scaffold_27:2640975-2642766 | 121.741 | 0.000213347 | clementine0.9_016673m | AT1G17020.1 | SRG1 | senescence-related gene 1 |
| 1. clementina_scaffold_8:639969-644738 | 117.801 | 0.0016722 | clementine0.9_005470m | AT5G58700.1 | PLC4 | phosphatidylinositol-speciwc phospholipase C4 |
| 1. clementina_scaffold_4:229810-231253 | 117.689 | 0.000468233 | clementine0.9_029145m | AT3G14460.1 |  | LRR and NB-ARC domains-containing disease resistance protein |
| 1. clementina_scaffold_25:547820-550076 | 117.616 | 1.83E-01 | clementine0.9_003751m | AT5G66810.1 |  |  |
| 1. clementina_scaffold_6:4439125-4441393 | 114.933 | 0.000920727 | clementine0.9_028841m | AT1G76570.1 |  | Chlorophyll A-B binding family protein |
| 1. clementina_scaffold_80:96737-97300 | 114.575 | 1.01E+00 | clementine0.9_026346m | AT3G18280.1 |  | Bifunctional inhibitor/lipid-transfer protein/seed storage 2S albumin superfamily protein |
| 1. clementina_scaffold_6:1529625-1531073 | 114.553 | 5.50E-01 | clementine0.9_014789m | AT3G49340.1 |  | Cysteine proteinases superfamily protein |
| 1. clementina_scaffold_102:725080-726517 | 112.536 | 0.0016268 | clementine0.9_005340m | AT3G18060.1 |  | transducin family protein / WD-40 repeat family protein |
| 1. clementina_scaffold_2:6370735-6372607 | 112.322 | 0.000941775 | clementine0.9_003735m | AT4G11860.1 |  | Protein of unknown function (DUF544) |
| 1. clementina_scaffold_38:406763-411186 | 111.684 | 1.01E-06 | clementine0.9_026268m | AT3G04650.1 |  | FAD/NAD(P)-binding oxidoreductase family protein |
| 1. clementina_scaffold_25:242121-243588 | 111.504 | 3.30E-01 | clementine0.9_019723m | AT4G34050.1 | CCoAOMT1 | S-adenosyl-L-methionine-dependent methyltransferases superfamily protein |
| 1. clementina_scaffold_3:6270824-6273265 | 106.869 | 9.08E-01 | clementine0.9_020125m | AT1G03070.1 |  | Bax inhibitor-1 family protein |
| 1. clementina_scaffold_60:482031-486470 | 106.363 | 0.00167709 | clementine0.9_020198m | AT2G44870.1 |  |  |
| 1. clementina_scaffold_4:2582175-2582666 | 106.068 | 1.81E+00 | clementine0.9_026231m | AT3G46320.1 |  | Histone superfamily protein |
| 1. clementina_scaffold_1:6757781-6759035 | 104.846 | 0.00174603 | clementine0.9_020612m | AT2G28190.1 | CSD2 | copper/zinc superoxide dismutase 2 |
| 1. clementina_scaffold_1:5091980-5094107 | 103.834 | 7.74E+00 | clementine0.9_027338m |  |  |  |
| 1. clementina_scaffold_13:3711024-3713767 | 102.773 | 0.000652834 | clementine0.9_003883m | AT3G21090.1 |  | ABC-2 type transporter family protein |
| 1. clementina_scaffold_4:2235256-2238662 | 102.503 | 1.33E-03 | clementine0.9_026695m | AT2G18740.1 |  | Small nuclear ribonucleoprotein family protein |
| 1. clementina_scaffold_34:127038-127959 | 101.742 | 0.000611399 | clementine0.9_014924m | AT1G21750.1 | PDIL1-1 | PDI-like 1-1 |
| 1. clementina_scaffold_12:2621456-2623235 | 100.988 | 0.000547341 | clementine0.9_017973m | AT5G20110.1 |  | Dynein light chain type 1 family protein |
| 1. clementina_scaffold_20:3403442-3405926 | 19.374 | 7.43E-01 | clementine0.9_014376m | AT3G27560.1 | ATN1 | Protein kinase superfamily protein |
| 1. clementina_scaffold_27:173216-174988 | 18.271 | 0.00017738 | clementine0.9_017288m | AT3G22550.1 |  | Protein of unknown function (DUF581) |
| 1. clementina_scaffold_82:443271-443493 | 13.544 | 5.35E-03 | clementine0.9_003736m | AT5G58270.1 | ATM3 | ABC transporter of the mitochondrion 3 |
| 1. clementina_scaffold_42:922200-922810 | 13.096 | 0.000358665 | clementine0.9_000508m | AT2G36910.1 | ABCB1 | ATP binding cassette subfamily B1 |
| 1. clementina_scaffold_80:936590-938252 | 11.132 | 0.000346269 | clementine0.9_009510m | AT5G38970.1 | BR6OX1 | brassinosteroid-6-oxidase 1 |
| 1. clementina_scaffold_63:727044-728233 | 1.587 | 0.00112869 | clementine0.9_006639m | AT4G26690.1 | SHV3 | PLC-like phosphodiesterase family protein |
| 1. clementina_scaffold_48:882575-883056 | 1.79 | 5.35E+00 | clementine0.9_011252m | AT5G61190.1 |  | putative endonuclease or glycosyl hydrolase with C2H2-type zinc finger domain |
| 1. clementina_scaffold_2:645196-646114 | 0.974451 | 0.000240907 | clementine0.9_022857m |  |  |  |
| 1. clementina_scaffold_144:42894-43373 | 0.971699 | 0.00037837 | clementine0.9_024216m | ATCG00020.1 | PSBA | photosystem II reaction center protein A |
| 1. clementina_scaffold_3:2125514-2129674 | 0.971502 | 0.000399959 | clementine0.9_008364m | AT1G01430.1 | TBL25 | TRICHOME BIREFRINGENCE-LIKE 25 |
| 1. clementina_scaffold_29:1207728-1211228 | 0.971237 | 3.99E-01 | clementine0.9_015994m | AT5G08100.1 |  | N-terminal nucleophile aminohydrolases (Ntn hydrolases) superfamily protein |
| 1. clementina_scaffold_8:998819-1002214 | 0.970327 | 3.05E-02 | clementine0.9_008486m | AT5G59010.1 |  | Protein kinase protein with tetratricopeptide repeat domain |
| 1. clementina_scaffold_34:1874733-1879634 | 0.963733 | 0.00129859 | clementine0.9_006545m | AT1G71800.1 | CSTF64 | cleavage stimulating factor 64 |
| 1. clementina_scaffold_20:3605205-3606523 | 0.955231 | 0.00117582 | clementine0.9_017983m | AT3G01660.1 |  | S-adenosyl-L-methionine-dependent methyltransferases superfamily protein |
| 1. clementina_scaffold_16:1354066-1355346 | 0.948456 | 0.00068586 | clementine0.9_017239m | AT4G01470.1 | TIP1;3 | tonoplast intrinsic protein 1;3 |
| 1. clementina_scaffold_14:1248469-1249551 | 0.94293 | 8.07E+00 | clementine0.9_020424m | AT1G17860.1 |  | Kunitz family trypsin and protease inhibitor protein |
| 1. clementina_scaffold_55:257072-258800 | 0.931637 | 2.98E-03 | clementine0.9_026137m | AT2G21580.2 |  | Ribosomal protein S25 family protein |
| 1. clementina_scaffold_49:863437-864164 | 0.928703 | 5.46E+00 | clementine0.9_007503m | AT5G48300.1 | ADG1 | ADP glucose pyrophosphorylase 1 |
| 1. clementina_scaffold_15:2263418-2268143 | 0.919848 | 0.00106612 | clementine0.9_003645m | AT3G54500.1 |  |  |
| 1. clementina_scaffold_20:1796302-1804163 | 0.908075 | 0.000312316 | clementine0.9_031057m | AT5G19500.1 |  | Tryptophan/tyrosine permease |
| 1. clementina_scaffold_10:681427-682915 | 0.903872 | 5.18E+00 | clementine0.9_018252m | AT1G10470.1 | ARR4 | response regulator 4 |
| 1. clementina_scaffold_2:7224957-7228062 | 0.903811 | 2.03E+00 | clementine0.9_010130m | AT1G12110.1 | NRT1.1 | nitrate transporter 1.1 |
| 1. clementina_scaffold_36:2013354-2013712 | 0.900522 | 5.57E+00 | clementine0.9_024832m | AT1G60950.1 | FED A | 2Fe-2S ferredoxin-like superfamily protein |
| 1. clementina_scaffold_12:4081836-4082738 | 0.880625 | 0.000391153 | clementine0.9_024650m |  |  |  |
| 1. clementina_scaffold_19:346372-347323 | 0.873085 | 0.00109783 | clementine0.9_022534m | AT4G16410.1 |  |  |
| 1. clementina_scaffold_11:950850-951259 | 0.868806 | 0.000107965 | clementine0.9_026407m | AT4G10300.1 |  | RmlC-like cupins superfamily protein |
| 1. clementina_scaffold_14:1226635-1227507 | 0.863845 | 7.25E+00 | clementine0.9_020626m | AT1G17860.1 |  | Kunitz family trypsin and protease inhibitor protein |
| 1. clementina_scaffold_1:9785614-9787210 | 0.858858 | 0.00100771 | clementine0.9_023159m | AT1G09330.1 |  |  |
| 1. clementina_scaffold_10:2889952-2890910 | 0.858334 | 0.00116516 | clementine0.9_018750m | AT5G13090.1 |  |  |
| 1. clementina_scaffold_51:1270620-1272848 | 0.852443 | 0.00142984 | clementine0.9_014318m | AT4G35160.1 |  | O-methyltransferase family protein |
| 1. clementina_scaffold_86:18812-20126 | 0.852157 | 0.000274084 | clementine0.9_005681m | AT1G53210.1 |  | sodium/calcium exchanger family protein / calcium-binding EF hand family protein |
| 1. clementina_scaffold_14:1375756-1376343 | 0.849274 | 0.00116963 | clementine0.9_024135m | ATCG00020.1 | PSBA | photosystem II reaction center protein A |
| 1. clementina_scaffold_16:238809-243896 | 0.845236 | 0.000321232 | clementine0.9_009706m | AT2G39220.1 | PLP6 | PATATIN-like protein 6 |
| 1. clementina_scaffold_34:522192-523151 | 0.842934 | 0.000106102 | clementine0.9_023779m | AT1G21550.1 |  | Calcium-binding EF-hand family protein |
| 1. clementina_scaffold_79:992632-992815 | 0.830754 | 0.000121473 | clementine0.9_030691m | AT1G16760.1 |  | Protein kinase protein with adenine nucleotide alpha hydrolases-like domain |
| 1. clementina_scaffold_19:1614663-1616755 | 0.830058 | 0.00165747 | clementine0.9_025221m | AT4G18100.1 |  | Ribosomal protein L32e |
| 1. clementina_scaffold_6:5188001-5190207 | 0.828655 | 3.07E-01 | clementine0.9_017725m | AT1G64090.1 | RTNLB3 | Reticulan like protein B3 |
| 1. clementina_scaffold_3:1980061-1982800 | 0.825512 | 0.00132202 | clementine0.9_004635m | AT5G41620.1 |  |  |
| 1. clementina_scaffold_22:1639365-1641333 | 0.820882 | 0.00144269 | clementine0.9_008057m | AT4G31500.1 | CYP83B1 | cytochrome P450. family 83. subfamily B. polypeptide 1 |
| 1. clementina_scaffold_3:705667-707290 | 0.816566 | 3.58E+00 | clementine0.9_015395m | AT2G45400.1 | BEN1 | NAD(P)-binding Rossmann-fold superfamily protein |
| 1. clementina_scaffold_1:6620855-6623509 | 0.81127 | 3.34E-03 | clementine0.9_024343m | AT1G08830.1 | CSD1 | copper/zinc superoxide dismutase 1 |
| 1. clementina_scaffold_1:4886161-4888538 | 0.81106 | 0.000330384 | clementine0.9_006943m | AT1G30700.1 |  | FAD-binding Berberine family protein |
| 1. clementina_scaffold_76:722903-727322 | 0.810663 | 1.45E-01 | clementine0.9_014291m | ATCG00270.1 | PSBD | photosystem II reaction center protein D |
| 1. clementina_scaffold_95:82370-83923 | 0.810252 | 0.000227338 | clementine0.9_012455m | AT5G13930.1 | TT4 | Chalcone and stilbene synthase family protein |
| 1. clementina_scaffold_9:2224223-2226210 | 0.80957 | 0.000830806 | clementine0.9_025426m | AT5G20160.1 |  | Ribosomal protein L7Ae/L30e/S12e/Gadd45 family protein |
| 1. clementina_scaffold_26:2954238-2954540 | 0.806704 | 0.000312733 | clementine0.9_025545m | AT3G53740.2 |  | Ribosomal protein L36e family protein |
| 1. clementina_scaffold_73:67163-68643 | 0.795495 | 0.00111456 | clementine0.9_016394m | AT5G22390.1 |  | Protein of unknown function (DUF3049) |
| 1. clementina_scaffold_10:2572161-2573573 | 0.794352 | 3.53E-01 | clementine0.9_020757m | AT1G67360.1 |  | Rubber elongation factor protein (REF) |
| 1. clementina_scaffold_4:5173149-5175021 | 0.793636 | 2.31E+00 | clementine0.9_019565m | AT5G20720.1 | CPN20 | chaperonin 20 |
| 1. clementina_scaffold_2:652248-653424 | 0.7904 | 0.000252025 | clementine0.9_024080m |  |  |  |
| 1. clementina_scaffold_125:259540-263042 | 0.790382 | 6.10E+00 | clementine0.9_005112m | AT5G10250.1 | DOT3 | Phototropic-responsive NPH3 family protein |
| 1. clementina_scaffold_9:4945248-4950838 | 0.789533 | 0.00134092 | clementine0.9_000822m | AT5G04895.1 |  | DEA(D/H)-box RNA helicase family protein |
| 1. clementina_scaffold_22:3380613-3381964 | 0.785807 | 0.000899444 | clementine0.9_035136m |  |  |  |
| 1. clementina_scaffold_68:327089-328668 | 0.777721 | 0.00126585 | clementine0.9_009967m | AT2G19070.1 | SHT | spermidine hydroxycinnamoyl transferase |
| 1. clementina_scaffold_31:545653-547163 | 0.7743 | 0.000145557 | clementine0.9_014566m | AT3G49340.1 |  | Cysteine proteinases superfamily protein |
| 1. clementina_scaffold_38:1178574-1180106 | 0.76801 | 0.000826321 | clementine0.9_020719m |  |  |  |
| 1. clementina_scaffold_3:1753176-1755261 | 0.762753 | 2.30E+00 | clementine0.9_018749m | AT4G01310.1 |  | Ribosomal L5P family protein |
| 1. clementina_scaffold_2:852589-854115 | 0.75575 | 0.00120559 | clementine0.9_016532m | AT5G05780.1 | RPN8A | RP non-ATPase subunit 8A |
| 1. clementina_scaffold_27:1439915-1440770 | 0.75432 | 0.000649728 | clementine0.9_026253m | AT1G04330.1 |  |  |
| 1. clementina_scaffold_25:1737172-1741321 | 0.749433 | 0.000255302 | clementine0.9_003255m | AT1G08070.1 | OTP82 | Tetratricopeptide repeat (TPR)-like superfamily protein |
| 1. clementina_scaffold_50:843869-848808 | 0.732652 | 5.38E+00 | clementine0.9_026813m |  |  |  |
| 1. clementina_scaffold_34:820760-821814 | 0.723667 | 2.62E+00 | clementine0.9_020916m | AT4G03520.1 | ATHM2 | Thioredoxin superfamily protein |
| 1. clementina_scaffold_10:3958175-3958888 | 0.723391 | 0.000841701 | clementine0.9_000715m | AT5G04020.1 |  | calmodulin binding |
| 1. clementina_scaffold_73:166028-167277 | 0.719147 | 0.000248622 | clementine0.9_023347m | AT2G27385.1 |  | Pollen Ole e 1 allergen and extensin family protein |
| 1. clementina_scaffold_99:767018-770093 | 0.707194 | 0.00155286 | clementine0.9_007527m | AT5G23960.2 | TPS21 | terpene synthase 21 |
| 1. clementina_scaffold_39:65512-69959 | 0.704578 | 0.00130849 | clementine0.9_005508m | AT3G48710.1 |  | DEK domain-containing chromatin associated protein |
| 1. clementina_scaffold_5:2085248-2085398 | 0.703606 | 0.00113653 | clementine0.9_020970m | AT4G18230.1 |  |  |
| 1. clementina_scaffold_124:352434-355804 | 0.70312 | 0.000165751 | clementine0.9_011406m | AT2G31130.1 |  |  |
| 1. clementina_scaffold_19:756424-760439 | 0.690591 | 0.000383642 | clementine0.9_003593m | AT4G22130.1 | SRF8 | STRUBBELIG-receptor family 8 |
| 1. clementina_scaffold_26:1023074-1026378 | 0.688852 | 0.00157244 | clementine0.9_017555m | AT4G34270.1 |  | TIP41-like family protein |
| 1. clementina_scaffold_78:107123-108877 | 0.684332 | 9.81E-01 | clementine0.9_007241m | AT1G65930.1 | cICDH | cytosolic NADP+-dependent isocitrate dehydrogenase |
| 1. clementina_scaffold_30:2657339-2660368 | 0.68413 | 0.000111613 | clementine0.9_013929m | AT2G41290.1 | SSL2 | strictosidine synthase-like 2 |
| 1. clementina_scaffold_19:135228-136920 | 0.682873 | 0.000947218 | clementine0.9_010242m | AT1G64810.2 | APO1 | Arabidopsis thaliana protein of unknown function (DUF794) |
| 1. clementina_scaffold_23:1582853-1584640 | 0.675085 | 0.00093453 | clementine0.9_026980m | AT2G23090.1 |  | Uncharacterised protein family SERF |
| 1. clementina_scaffold_63:785744-787693 | 0.674851 | 0.000120772 | clementine0.9_009056m | AT5G10770.1 |  | Eukaryotic aspartyl protease family protein |
| 1. clementina_scaffold_36:269937-271517 | 0.673956 | 4.81E-02 | clementine0.9_021738m | AT1G61520.2 | LHCA3 | photosystem I light harvesting complex gene 3 |
| 1. clementina_scaffold_1:4270801-4274294 | 0.660514 | 3.39E+00 | clementine0.9_005983m | AT5G45275.1 |  | Major facilitator superfamily protein |
| 1. clementina_scaffold_53:627540-632024 | 0.659685 | 0.000207328 | clementine0.9_002997m | AT3G01180.1 | SS2 | starch synthase 2 |
| 1. clementina_scaffold_128:428513-429217 | 0.652673 | 0.000352681 | clementine0.9_025653m | AT5G20935.1 |  |  |
| 1. clementina_scaffold_31:50307-55534 | 0.64285 | 0.000907122 | clementine0.9_017070m | AT2G32080.2 | PUR ALPHA-1 | purin-rich alpha 1 |
| 1. clementina_scaffold_25:3459927-3464150 | 0.642055 | 0.000952047 | clementine0.9_011123m | AT2G17500.1 |  | Auxin efflux carrier family protein |
| 1. clementina_scaffold_4:7222354-7227299 | 0.632562 | 0.000197804 | clementine0.9_010277m | AT5G55060.1 |  |  |
| 1. clementina_scaffold_27:1891729-1895671 | 0.617634 | 0.000410588 | clementine0.9_015365m | AT5G43330.1 |  | Lactate/malate dehydrogenase family protein |
| 1. clementina_scaffold_8:3927932-3932578 | 0.616662 | 5.99E+00 | clementine0.9_020612m | AT2G28190.1 | CSD2 | copper/zinc superoxide dismutase 2 |
| 1. clementina_scaffold_7:1318792-1321007 | 0.605288 | 0.000487483 | clementine0.9_013104m | AT2G37620.1 | ACT1 | actin 1 |
| 1. clementina_scaffold_91:776917-780480 | 0.604101 | 0.000872954 | clementine0.9_005402m | AT1G08540.1 | SIG2 | RNApolymerase sigma subunit 2 |
| 1. clementina_scaffold_9:1568483-1570530 | 0.597726 | 0.000585907 | clementine0.9_016221m | AT5G56860.1 | GNC | GATA type zinc finger transcription factor family protein |
| 1. clementina_scaffold_126:56306-60310 | 0.597675 | 0.000897173 | clementine0.9_001984m | AT4G03230.1 |  | S-locus lectin protein kinase family protein |
| 1. clementina_scaffold_2:4696917-4699108 | 0.597302 | 0.000406026 | clementine0.9_025176m | AT3G11750.1 | FOLB1 | Dihydroneopterin aldolase |
| 1. clementina_scaffold_8:1415938-1420728 | 0.592059 | 0.00144088 | clementine0.9_018371m | AT3G55530.1 | SDIR1 | RING/U-box superfamily protein |
| 1. clementina_scaffold_9:962016-965084 | 0.588201 | 0.00102594 | clementine0.9_012940m | AT5G11650.1 |  | alpha/beta-Hydrolases superfamily protein |
| 1. clementina_scaffold_2:5317145-5325460 | 0.586263 | 0.000233946 | clementine0.9_031396m | AT2G36145.1 |  |  |
| 1. clementina_scaffold_21:3877890-3884141 | 0.57473 | 0.000106063 | clementine0.9_012806m | AT2G43020.1 | PAO2 | polyamine oxidase 2 |
| 1. clementina_scaffold_6:4571109-4574182 | 0.541937 | 0.000392901 | clementine0.9_003839m | AT5G23890.1 |  |  |
| 1. clementina_scaffold_114:219604-222526 | 0.506043 | 0.00171532 | clementine0.9_003933m | AT4G30080.1 | ARF16 | auxin response factor 16 |
| 1. clementina_scaffold_26:2349108-2353996 | 0.470276 | 0.00114289 | clementine0.9_017729m | AT4G35000.1 | APX3 | ascorbate peroxidase 3 |
| 1. clementina_scaffold_15:3058865-3059977 | 0.467441 | 0.000138765 | clementine0.9_025401m | AT1G30380.1 | PSAK | photosystem I subunit K |
| 1. clementina_scaffold_7:6397729-6400369 | 0.459035 | 6.53E+00 | clementine0.9_013519m | AT3G06483.1 | PDK | pyruvate dehydrogenase kinase |
| 1. clementina_scaffold_4:1246117-1249857 | 0.443011 | 0.000672525 | clementine0.9_008326m | AT2G24270.4 | ALDH11A3 | aldehyde dehydrogenase 11A3 |
| 1. clementina_scaffold_3:2866197-2869131 | 0.434702 | 0.000495553 | clementine0.9_005293m | AT1G32900.1 |  | UDP-Glycosyltransferase superfamily protein |
| 1. clementina_scaffold_23:3258334-3263126 | 0.41588 | 0.000416395 | clementine0.9_020406m | AT5G10350.2 |  | RNA-binding (RRM/RBD/RNP motifs) family protein |
| 1. clementina_scaffold_8:836926-841918 | 0.411267 | 0.000403569 | clementine0.9_005391m | AT3G47000.1 |  | Glycosyl hydrolase family protein |

*The fold change values (P ≤ 0.001) obtained from of each treated sample compared to HCl control.

** Identification number of *Citrus clementina* transcripts present in the locus - http://www.phytozome.org/search.php

***Identification number of the *Arabidopsis thaliana* ortholog of up-regulated citrus gene in response to CHI treatment (The Arabidopsis Genome Initiative).
