# Supplementary material for: Transcriptional profile of sweet orange in response to chitosan and salicylic acid
Source: BMC Genomics. 2015 Apr 12;16(1):288. doi: 10.1186/s12864-015-1440-5 (PMC4415254; doi:10.1186/s12864-015-1440-5)
Supplement: Additional file 8: Table S6-2. — Differentially expressed genes that were downregulated in CHI-treated plants. [file 12864_2015_1440_MOESM8_ESM.docx]

**Table S6-2** Differentially expressed genes that were down-regulated in CHI-treated plants.

| **Locus** | **Log2**  **(fold change)*** | **p_value** | **ID**** | ***Arabidopsis thaliana****** | **Gene** | **Description** |
| --- | --- | --- | --- | --- | --- | --- |
| 1. clementina_scaffold_54:376165-376812 | -518.527 | 4.38E-04 | clementine0.9_002853m | AT5G59810.1 | SBT5.4 | Subtilase family protein |
| 1. clementina_scaffold_19:1589839-1592076 | -383.642 | 0 | clementine0.9_009102m | AT5G46410.2 | SSP4 | SCP1-like small phosphatase 4 |
| 1. clementina_scaffold_20:1574884-1575844 | -372.426 | 0.0007505 | clementine0.9_000025m | AT4G30990.1 |  | ARM repeat superfamily protein |
| 1. clementina_scaffold_76:289148-291511 | -331.697 | 6.49E+00 | clementine0.9_006222m | AT2G40890.1 | CYP98A3 | cytochrome P450. family 98. subfamily A. polypeptide 3 |
| 1. clementina_scaffold_10:3958175-3958888 | -329.865 | 1.87E-06 | clementine0.9_000715m | AT5G04020.1 |  | calmodulin binding |
| 1. clementina_scaffold_121:481737-486503 | -312.822 | 3.36E-01 | clementine0.9_002847m | AT3G60320.1 |  | Protein of unknown function (DUF630 and DUF632) |
| 1. clementina_scaffold_3:5735744-5736973 | -306.695 | 1.28E-08 | clementine0.9_011390m | AT5G63380.1 |  | AMP-dependent synthetase and ligase family protein |
| 1. clementina_scaffold_27:372356-376080 | -306.514 | 5.71E-04 | clementine0.9_018179m | AT4G14740.1 |  | Plant protein of unknown function (DUF828) with plant pleckstrin homology-like region |
| 1. clementina_scaffold_7:4290690-4298480 | -296.434 | 0 | clementine0.9_000011m | AT2G28290.1 | SYD | P-loop containing nucleoside triphosphate hydrolases superfamily protein |
| 1. clementina_scaffold_24:1927791-1931628 | -276.453 | 0 | clementine0.9_009149m | AT5G12080.1 | MSL10 | mechanosensitive channel of small conductance-like 10 |
| 1. clementina_scaffold_28:810541-812990 | -271.411 | 0.0012857 | clementine0.9_000733m | AT4G21710.1 | NRPB2 | DNA-directed RNA polymerase family protein |
| 1. clementina_scaffold_3:2408486-2408885 | -271.277 | 0.0012801 | clementine0.9_010047m | AT4G00980.1 |  | zinc knuckle (CCHC-type) family protein |
| 1. clementina_scaffold_22:201858-202934 | -266.054 | 0.0001632 | clementine0.9_003225m | AT1G20980.1 | SPL14 | squamosa promoter binding protein-like 14 |
| 1. clementina_scaffold_25:464248-465288 | -265.387 | 0.0016618 | clementine0.9_000771m | AT3G26560.1 |  | ATP-dependent RNA helicase. putative |
| 1. clementina_scaffold_34:929662-930144 | -263.115 | 0.0006017 | clementine0.9_001536m | AT4G08350.1 | GTA2 | global transcription factor group A2 |
| 1. clementina_scaffold_3:8223944-8228572 | -257.046 | 0.000914 | clementine0.9_000075m | AT3G24870.1 |  | Helicase/SANT-associated. DNA binding protein |
| 1. clementina_scaffold_2:7424591-7425665 | -249.489 | 0.0004194 | clementine0.9_006327m | AT4G17300.1 | NS1 | Class II aminoacyl-tRNA and biotin synthetases superfamily protein |
| 1. clementina_scaffold_19:99778-100597 | -248.507 | 0.0001697 | clementine0.9_000480m | AT3G13300.1 | VCS | Transducin/WD40 repeat-like superfamily protein |
| 1. clementina_scaffold_17:1270743-1270929 | -247.401 | 0.0007403 | clementine0.9_007626m | AT3G14690.1 | CYP72A15 | cytochrome P450. family 72. subfamily A. polypeptide 15 |
| 1. clementina_scaffold_108:299472-300429 | -246.123 | 5.68E-04 | clementine0.9_029016m | AT4G10780.1 |  | LRR and NB-ARC domains-containing disease resistance protein |
| 1. clementina_scaffold_15:3088650-3092763 | -243.497 | 2.25E-01 | clementine0.9_031858m | AT2G34660.1 | MRP2 | multidrug resistance-associated protein 2 |
| 1. clementina_scaffold_2:5236018-5237329 | -243.299 | 0.0004123 | clementine0.9_002197m | AT3G11560.2 |  | LETM1-like protein |
| 1. clementina_scaffold_137:82193-86352 | -240.678 | 0.0007235 | clementine0.9_017083m | AT2G20890.1 | PSB29 | photosystem II reaction center PSB29 protein |
| 1. clementina_scaffold_11:2451918-2452359 | -236.572 | 2.37E+00 | clementine0.9_001221m | AT5G17680.1 |  | disease resistance protein (TIR-NBS-LRR class). putative |
| 1. clementina_scaffold_9:2128065-2130516 | -230.262 | 9.92E+00 | clementine0.9_017518m | AT2G26660.1 | SPX2 | SPX domain gene 2 |
| 1. clementina_scaffold_9:2946983-2948515 | -229.845 | 4.41E+00 | clementine0.9_012288m | AT4G13400.1 |  | 2-oxoglutarate (2OG) and Fe(II)-dependent oxygenase superfamily protein |
| 1. clementina_scaffold_3:3161546-3163374 | -228.474 | 0.0003022 | clementine0.9_031483m | AT2G46915.1 |  | Protein of unknown function (DUF3754) |
| 1. clementina_scaffold_44:1332514-1334748 | -227.664 | 3.78E-02 | clementine0.9_028481m | AT1G12775.1 |  | Pentatricopeptide repeat (PPR) superfamily protein |
| 1. clementina_scaffold_8:2833241-2835196 | -227.067 | 2.40E-05 | clementine0.9_002618m | AT5G60450.1 | ARF4 | auxin response factor 4 |
| 1. clementina_scaffold_1:1340210-1345843 | -226.751 | 0.000123 | clementine0.9_010873m | AT4G19120.1 | ERD3 | S-adenosyl-L-methionine-dependent methyltransferases superfamily protein |
| 1. clementina_scaffold_9:1103118-1103885 | -225.615 | 8.31E-01 | clementine0.9_014449m | AT2G25850.4 | PAPS2 | poly(A) polymerase 2 |
| 1. clementina_scaffold_18:15874-18027 | -225.612 | 0.000153 | clementine0.9_015842m | AT5G24400.1 | EMB2024 | NagB/RpiA/CoA transferase-like superfamily protein |
| 1. clementina_scaffold_12:705528-711043 | -222.387 | 3.30E+00 | clementine0.9_000355m | AT5G47020.1 |  |  |
| 1. clementina_scaffold_94:117428-119903 | -221.144 | 0.0009909 | clementine0.9_000177m | AT4G00990.1 |  | Transcription factor jumonji (jmjC) domain-containing protein |
| 1. clementina_scaffold_139:354156-354774 | -219.592 | 0.0002054 | clementine0.9_009705m | AT1G73875.1 |  | DNAse I-like superfamily protein |
| 1. clementina_scaffold_16:266588-269806 | -218.697 | 8.97E-02 | clementine0.9_001834m | AT2G28880.1 | emb1997 | para-aminobenzoate (PABA) synthase family protein |
| 1. clementina_scaffold_34:1350855-1351393 | -216.905 | 0.0011977 | clementine0.9_032923m | AT1G74190.1 | RLP15 | receptor like protein 15 |
| 1. clementina_scaffold_16:522116-524717 | -216.661 | 5.76E-01 | clementine0.9_012701m | AT4G02120.1 |  | CTP synthase family protein |
| 1. clementina_scaffold_39:1388507-1391652 | -216.392 | 0.0001926 | clementine0.9_034690m | AT1G31150.1 |  | Domain of unknown function (DUF1985) |
| 1. clementina_scaffold_3:4854051-4855731 | -215.875 | 0.0006 | clementine0.9_010996m | AT2G47760.1 | ALG3 | asparagine-linked glycosylation 3 |
| 1. clementina_scaffold_23:1508945-1513203 | -213.381 | 5.09E+00 | clementine0.9_036075m | AT4G37460.1 | SRFR1 | Tetratricopeptide repeat (TPR)-like superfamily protein |
| 1. clementina_scaffold_10:2308521-2309866 | -213.125 | 0.0014568 | clementine0.9_001782m | AT1G67560.1 |  | PLAT/LH2 domain-containing lipoxygenase family protein |
| 1. clementina_scaffold_68:737362-738927 | -210.893 | 0.001687 | clementine0.9_001973m | AT5G63020.1 |  | Disease resistance protein (CC-NBS-LRR class) family |
| 1. clementina_scaffold_9:4089136-4089338 | -210.893 | 0.001687 | clementine0.9_011501m | AT4G30780.1 |  |  |
| 1. clementina_scaffold_3:162492-162987 | -209.866 | 6.99E+00 | clementine0.9_003470m | AT2G44950.1 | HUB1 | histone mono-ubiquitination 1 |
| 1. clementina_scaffold_3:319586-324284 | -209.768 | 0.0004341 | clementine0.9_000037m | AT1G02080.2 |  | transcription regulators |
| 1. clementina_scaffold_115:18825-20565 | -209.675 | 0.0008796 | clementine0.9_006328m | AT5G59190.1 |  | subtilase family protein |
| 1. clementina_scaffold_10:3014402-3022848 | -209.515 | 0.0002178 | clementine0.9_003004m | AT1G69220.1 | SIK1 | Protein kinase superfamily protein |
| 1. clementina_scaffold_54:1522999-1523660 | -208.926 | 0.000928 | clementine0.9_023410m | AT3G07470.1 |  | Protein of unknown function. DUF538 |
| 1. clementina_scaffold_26:2938298-2939688 | -206.221 | 6.58E-02 | clementine0.9_015646m | AT3G12500.1 | HCHIB | basic chitinase |
| 1. clementina_scaffold_7:2907208-2908013 | -205.783 | 0.0006204 | clementine0.9_000778m | AT4G26090.1 | RPS2 | NB-ARC domain-containing disease resistance protein |
| 1. clementina_scaffold_25:2199503-2201501 | -204.619 | 7.75E-01 | clementine0.9_032993m | AT4G35740.1 | RecQl3 | DEAD/DEAH box RNA helicase family protein |
| 1. clementina_scaffold_38:1088877-1091119 | -203.761 | 2.99E-02 | clementine0.9_029560m | AT3G14460.1 |  | LRR and NB-ARC domains-containing disease resistance protein |
| 1. clementina_scaffold_6:6633204-6635567 | -202.719 | 0.001313 | clementine0.9_000491m | AT1G79280.1 | NUA | nuclear pore anchor |
| 1. clementina_scaffold_7:6329323-6330056 | -202.052 | 0.0002143 | clementine0.9_033245m | AT3G53480.1 | PDR9 | pleiotropic drug resistance 9 |
| 1. clementina_scaffold_7:6360242-6361857 | -201.998 | 6.55E-02 | clementine0.9_003888m | AT3G60030.1 | SPL12 | squamosa promoter-binding protein-like 12 |
| 1. clementina_scaffold_21:872604-874739 | -201.966 | 0.0003496 | clementine0.9_008928m | AT2G05790.1 |  | O-Glycosyl hydrolases family 17 protein |
| 1. clementina_scaffold_1:10105434-10106626 | -201.922 | 0.0007387 | clementine0.9_000808m | AT1G27750.1 |  | nucleic acid binding |
| 1. clementina_scaffold_1:9644279-9647912 | -200.166 | 2.19E+00 | clementine0.9_032351m | AT4G27190.1 |  | NB-ARC domain-containing disease resistance protein |
| 1. clementina_scaffold_85:873138-877085 | -198.862 | 0.0009813 | clementine0.9_000684m | AT2G02560.2 | CAND1 | cullin-associated and neddylation dissociated |
| 1. clementina_scaffold_25:3165709-3168103 | -198.313 | 0.0009104 | clementine0.9_032993m | AT4G35740.1 | RecQl3 | DEAD/DEAH box RNA helicase family protein |
| 1. clementina_scaffold_3:2312336-2314133 | -197.199 | 6.04E-05 | clementine0.9_014342m | AT3G61740.1 | SDG14 | SET domain protein 14 |
| 1. clementina_scaffold_3:55057-56590 | -196.937 | 0.0002083 | clementine0.9_004130m | AT2G44900.1 | ARABIDILLO-1 | ARABIDILLO-1 |
| 1. clementina_scaffold_18:3014387-3016540 | -194.679 | 8.90E-01 | clementine0.9_001060m | AT1G48410.1 | ago/01 | Stabilizer of iron transporter SufD / Polynucleotidyl transferase |
| 1. clementina_scaffold_12:2694626-2697160 | -194.558 | 0.0014037 | clementine0.9_011395m | AT1G74780.1 |  | Nodulin-like / Major Facilitator Superfamily protein |
| 1. clementina_scaffold_58:1248418-1251482 | -194.437 | 3.70E-01 | clementine0.9_001030m | AT2G20050.1 |  | protein serine/threonine phosphatases;protein kinases;catalytics;cAMP-dependent protein kinase regulators;ATP binding;protein serine/threonine phosphatases |
| 1. clementina_scaffold_7:3181440-3184602 | -192.983 | 1.19E-06 | clementine0.9_000746m | AT3G22380.2 | TIC | time for coffee |
| 1. clementina_scaffold_1:11735669-11738463 | -191.926 | 1.45E-04 | clementine0.9_020234m | AT4G08980.1 | FBW2 | F-BOX WITH WD-40 2 |
| 1. clementina_scaffold_3:6033908-6034391 | -190.601 | 0.0015532 | clementine0.9_010825m | AT4G02740.1 |  | F-box/RNI-like superfamily protein |
| 1. clementina_scaffold_10:1072584-1076211 | -188.634 | 0.0001001 | clementine0.9_002981m | AT1G60200.1 |  | splicing factor PWI domain-containing protein / RNA recognition motif (RRM)-containing protein |
| 1. clementina_scaffold_10:3988515-3990846 | -187.894 | 0.0010268 | clementine0.9_001096m | AT1G68750.1 | PPC4 | phosphoenolpyruvate carboxylase 4 |
| 1. clementina_scaffold_8:2201139-2202371 | -187.563 | 2.08E+00 | clementine0.9_001112m | AT4G00900.1 | ECA2 | ER-type Ca2+-ATPase 2 |
| 1. clementina_scaffold_19:3110118-3110666 | -185.431 | 0.0008972 | clementine0.9_002930m | AT3G14067.1 |  | Subtilase family protein |
| 1. clementina_scaffold_67:708147-711668 | -185.335 | 0.001251 | clementine0.9_003391m | AT1G65580.1 | FRA3 | Endonuclease/exonuclease/phosphatase family protein |
| 1. clementina_scaffold_10:2979936-2980864 | -183.281 | 0.0014167 | clementine0.9_007994m | AT1G26560.1 | BGLU40 | beta glucosidase 40 |
| 1. clementina_scaffold_84:932248-933125 | -182.936 | 2.66E-01 | clementine0.9_002282m | AT5G62000.1 | ARF2 | auxin response factor 2 |
| 1. clementina_scaffold_22:14868-16887 | -182.729 | 2.19E-01 | clementine0.9_014291m | ATCG00270.1 | PSBD | photosystem II reaction center protein D |
| 1. clementina_scaffold_8:2862204-2865461 | -181.635 | 5.59E-01 | clementine0.9_006033m | AT1G61560.1 | MLO6 | Seven transmembrane MLO family protein |
| 1. clementina_scaffold_12:1984339-1986031 | -181.535 | 3.67E+00 | clementine0.9_002445m | AT4G32180.2 | PANK2 | pantothenate kinase 2 |
| 1. clementina_scaffold_15:4026250-4028999 | -180.988 | 2.76E-02 | clementine0.9_001130m | AT1G56130.1 |  | Leucine-rich repeat transmembrane protein kinase |
| 1. clementina_scaffold_12:14795-17925 | -180.541 | 0.0015483 | clementine0.9_002941m | AT1G15290.1 |  | Tetratricopeptide repeat (TPR)-like superfamily protein |
| 1. clementina_scaffold_19:1278293-1278654 | -180.172 | 1.92E-08 | clementine0.9_016154m | AT5G46250.2 |  | RNA-binding protein |
| 1. clementina_scaffold_8:2427136-2428673 | -180.026 | 0.0005013 | clementine0.9_001522m | AT5G58160.1 |  | actin binding |
| 1. clementina_scaffold_1:10912355-10913738 | -178.955 | 5.44E-01 | clementine0.9_000019m | AT1G70320.1 | UPL2 | ubiquitin-protein ligase 2 |
| 1. clementina_scaffold_10:4033077-4036688 | -177.918 | 0.0008401 | clementine0.9_031455m | AT1G67230.1 | LINC1 | little nuclei1 |
| 1. clementina_scaffold_151:127583-130073 | -177.402 | 0.0005228 | clementine0.9_035651m | AT5G06710.1 | HAT14 | homeobox from Arabidopsis thaliana |
| 1. clementina_scaffold_7:3231450-3234018 | -176.964 | 0.0006049 | clementine0.9_031794m | AT5G27600.1 | LACS7 | long-chain acyl-CoA synthetase 7 |
| 1. clementina_scaffold_10:4722955-4724175 | -176.906 | 0.0010513 | clementine0.9_010413m | AT3G19970.1 |  | alpha/beta-Hydrolases superfamily protein |
| 1. clementina_scaffold_19:2298735-2304472 | -175.805 | 1.09E-07 | clementine0.9_013631m | AT1G29050.1 | TBL38 | TRICHOME BIREFRINGENCE-LIKE 38 |
| 1. clementina_scaffold_12:3359554-3360644 | -175.682 | 0.0016641 | clementine0.9_003637m | AT1G63700.1 | YDA | Protein kinase superfamily protein |
| 1. clementina_scaffold_10:1439239-1441513 | -175.539 | 0.0010881 | clementine0.9_000452m | AT1G10760.1 | SEX1 | Pyruvate phosphate dikinase. PEP/pyruvate binding domain |
| 1. clementina_scaffold_67:100058-101501 | -175.073 | 0.0003386 | clementine0.9_031429m | AT1G18390.2 |  | Protein kinase superfamily protein |
| 1. clementina_scaffold_84:814835-818291 | -174.592 | 3.27E+00 | clementine0.9_002227m | AT5G61960.1 | ML1 | MEI2-like protein 1 |
| 1. clementina_scaffold_8:1096580-1097174 | -173.686 | 0.000243 | clementine0.9_028184m | AT5G59030.1 | COPT1 | copper transporter 1 |
| 1. clementina_scaffold_28:217262-217625 | -172.426 | 0.0013607 | clementine0.9_005419m | AT1G74250.1 |  | DNAJ heat shock N-terminal domain-containing protein |
| 1. clementina_scaffold_8:3542610-3543510 | -172.151 | 2.31E-04 | clementine0.9_028735m | AT1G49290.1 |  |  |
| 1. clementina_scaffold_7:4395137-4396675 | -169.569 | 0.0007001 | clementine0.9_005815m | AT5G41070.1 | DRB5 | dsRNA-binding protein 5 |
| 1. clementina_scaffold_88:929307-930507 | -168.602 | 0.0003174 | clementine0.9_000348m | AT1G15520.1 | PDR12 | pleiotropic drug resistance 12 |
| 1. clementina_scaffold_8:1315022-1317714 | -167.279 | 2.89E+00 | clementine0.9_031086m | AT2G29210.1 |  | splicing factor PWI domain-containing protein |
| 1. clementina_scaffold_32:657980-660841 | -167.153 | 5.51E-01 | clementine0.9_004816m | AT3G14010.1 | CID4 | CTC-interacting domain 4 |
| 1. clementina_scaffold_9:1165990-1166857 | -166.916 | 0.0013812 | clementine0.9_000426m | AT5G11700.2 |  |  |
| 1. clementina_scaffold_33:2325536-2326647 | -165.456 | 0.0013684 | clementine0.9_005759m | AT1G53800.1 |  |  |
| 1. clementina_scaffold_3:3018718-3019782 | -164.775 | 0.0010683 | clementine0.9_000089m | AT1G01040.1 | DCL1 | dicer-like 1 |
| 1. clementina_scaffold_25:3102807-3104561 | -164.038 | 1.03E+00 | clementine0.9_004676m | AT4G35790.2 | PLDDELTA | phospholipase D delta |
| 1. clementina_scaffold_195:13298-16341 | -161.766 | 6.26E-01 | clementine0.9_018591m | AT2G37340.1 | RSZ33 | arginine/serine-rich zinc knuckle-containing protein 33 |
| 1. clementina_scaffold_2:240966-242136 | -160.945 | 0.0009661 | clementine0.9_005134m | AT3G54010.1 | PAS1 | FKBP-type peptidyl-prolyl cis-trans isomerase family protein |
| 1. clementina_scaffold_4:1579481-1581868 | -160.135 | 2.03E-01 | clementine0.9_001159m | AT5G17420.1 | IRX3 | Cellulose synthase family protein |
| 1. clementina_scaffold_39:548901-549764 | -159.873 | 0.0006546 | clementine0.9_020666m | AT5G54890.1 |  | RNA-binding CRS1 / YhbY (CRM) domain-containing protein |
| 1. clementina_scaffold_2:4402088-4403335 | -159.236 | 3.63E-01 | clementine0.9_002177m | AT3G56150.1 | EIF3C | eukaryotic translation initiation factor 3C |
| 1. clementina_scaffold_32:361224-362963 | -158.577 | 0.0003811 | clementine0.9_003390m | AT1G72990.1 | BGAL17 | beta-galactosidase 17 |
| 1. clementina_scaffold_15:1656889-1660685 | -157.702 | 7.66E+00 | clementine0.9_010121m | AT3G55850.1 | LAF3 | Amidohydrolase family |
| 1. clementina_scaffold_7:4486537-4489635 | -157.634 | 1.05E+00 | clementine0.9_003620m | AT5G18590.1 |  | Galactose oxidase/kelch repeat superfamily protein |
| 1. clementina_scaffold_10:636965-637879 | -156.881 | 8.27E+00 | clementine0.9_000382m | AT1G70060.1 | SNL4 | SIN3-like 4 |
| 1. clementina_scaffold_10:1076311-1079070 | -156.268 | 2.13E-08 | clementine0.9_001718m | AT1G60200.1 |  | splicing factor PWI domain-containing protein / RNA recognition motif (RRM)-containing protein |
| 1. clementina_scaffold_123:172874-176570 | -156.157 | 4.14E-01 | clementine0.9_011496m | AT2G37025.1 | TRFL8 | TRF-like 8 |
| 1. clementina_scaffold_1:10910258-10912110 | -155.004 | 0.0007994 | clementine0.9_000019m | AT1G70320.1 | UPL2 | ubiquitin-protein ligase 2 |
| 1. clementina_scaffold_21:1796236-1797807 | -154.131 | 0.0002396 | clementine0.9_007512m | AT3G21690.1 |  | MATE efflux family protein |
| 1. clementina_scaffold_34:670782-671860 | -152.724 | 0.0014618 | clementine0.9_005898m | AT2G34300.1 |  | S-adenosyl-L-methionine-dependent methyltransferases superfamily protein |
| 1. clementina_scaffold_23:2472923-2479183 | -152.299 | 0 | clementine0.9_005395m | AT2G22660.2 |  | Protein of unknown function (duplicated DUF1399) |
| 1. clementina_scaffold_9:1014282-1016865 | -151.787 | 6.52E-05 | clementine0.9_005184m | AT5G25880.1 | NADP-ME3 | NADP-malic enzyme 3 |
| 1. clementina_scaffold_47:450439-453399 | -150.171 | 0.0001014 | clementine0.9_016594m | AT2G40830.1 | RHC1A | RING-H2 finger C1A |
| 1. clementina_scaffold_4:1720661-1725428 | -148.386 | 6.54E-03 | clementine0.9_000137m | AT4G28080.1 |  | Tetratricopeptide repeat (TPR)-like superfamily protein |
| 1. clementina_scaffold_53:224778-227623 | -147.974 | 0.0008478 | clementine0.9_000154m | AT5G15540.1 | EMB2773 | PHD finger family protein |
| 1. clementina_scaffold_71:1298489-1299290 | -147.263 | 2.58E+00 | clementine0.9_028648m | AT4G18670.1 |  | Leucine-rich repeat (LRR) family protein |
| 1. clementina_scaffold_29:55409-58105 | -145.633 | 3.70E+00 | clementine0.9_006000m | AT1G49050.1 |  | Eukaryotic aspartyl protease family protein |
| 1. clementina_scaffold_18:1577411-1581334 | -145.385 | 8.89E-03 | clementine0.9_026434m |  |  |  |
| 1. clementina_scaffold_1:6217939-6220647 | -144.863 | 0.000197 | clementine0.9_000227m | AT5G27970.1 |  | ARM repeat superfamily protein |
| 1. clementina_scaffold_4:3530378-3534686 | -142.143 | 7.51E-06 | clementine0.9_029607m | AT2G07050.1 | CAS1 | cycloartenol synthase 1 |
| 1. clementina_scaffold_51:1449224-1449998 | -141.688 | 0.0010802 | clementine0.9_009230m | AT4G13430.1 | IIL1 | isopropyl malate isomerase large subunit 1 |
| 1. clementina_scaffold_136:221454-222357 | -141.589 | 0.0009921 | clementine0.9_025412m | AT2G26500.1 |  | cytochrome b6f complex subunit (petM). putative |
| 1. clementina_scaffold_16:678375-680603 | -141.195 | 0.0002109 | clementine0.9_034548m | AT3G54970.1 |  | D-aminoacid aminotransferase-like PLP-dependent enzymes superfamily protein |
| 1. clementina_scaffold_29:924912-927898 | -140.229 | 0.0010335 | clementine0.9_010733m | AT1G73980.1 |  | Phosphoribulokinase / Uridine kinase family |
| 1. clementina_scaffold_12:4937675-4938720 | -139.284 | 0.0002025 | clementine0.9_027411m | AT1G33055.1 |  |  |
| 1. clementina_scaffold_3:7863689-7866329 | -139.276 | 8.22E-02 | clementine0.9_000393m | AT2G20190.1 | CLASP | CLIP-associated protein |
| 1. clementina_scaffold_3:8685786-8687536 | -139.035 | 0.0010458 | clementine0.9_004419m | AT1G58030.1 | CAT2 | cationic amino acid transporter 2 |
| 1. clementina_scaffold_1:7402189-7404386 | -138.123 | 0.0014252 | clementine0.9_006091m | AT5G27380.1 | GSH2 | glutathione synthetase 2 |
| 1. clementina_scaffold_68:932028-932756 | -137.567 | 0.0012194 | clementine0.9_009980m | AT1G80480.1 | PTAC17 | plastid transcriptionally active 17 |
| 1. clementina_scaffold_80:248370-250980 | -136.756 | 0.0008752 | clementine0.9_022745m | AT5G16130.1 |  | Ribosomal protein S7e family protein |
| 1. clementina_scaffold_47:845638-850322 | -136.488 | 1.66E+00 | clementine0.9_012131m | AT2G26800.2 |  | Aldolase superfamily protein |
| 1. clementina_scaffold_33:1677248-1679109 | -136.366 | 7.11E+00 | clementine0.9_002669m | AT3G16290.1 | EMB2083 | AAA-type ATPase family protein |
| 1. clementina_scaffold_9:1371006-1375325 | -135.993 | 8.90E-02 | clementine0.9_004265m | AT2G25970.1 |  | KH domain-containing protein |
| 1. clementina_scaffold_3:1522827-1524444 | -135.594 | 0.00031 | clementine0.9_013957m | AT3G61415.1 | SK21 | SKP1-like 21 |
| 1. clementina_scaffold_10:5543485-5545394 | -135.128 | 0.0005312 | clementine0.9_013904m | AT1G50300.1 | TAF15 | TBP-associated factor 15 |
| 1. clementina_scaffold_6:4692274-4694159 | -134.575 | 0.0015909 | clementine0.9_000745m | AT4G09980.1 | EMB1691 | Methyltransferase MT-A70 family protein |
| 1. clementina_scaffold_9:2027501-2028515 | -134.339 | 4.20E+00 | clementine0.9_004918m | AT2G25490.1 | EBF1 | EIN3-binding F box protein 1 |
| 1. clementina_scaffold_34:217753-220117 | -133.888 | 0.000322 | clementine0.9_016936m | AT1G01720.1 | ATAF1 | NAC (No Apical Meristem) domain transcriptional regulator superfamily protein |
| 1. clementina_scaffold_3:4823909-4826875 | -133.084 | 0.0001366 | clementine0.9_000644m | AT1G02890.1 |  | AAA-type ATPase family protein |
| 1. clementina_scaffold_25:2300707-2301735 | -132.394 | 7.67E-01 | clementine0.9_022677m | AT2G18050.1 | HIS1-3 | histone H1-3 |
| 1. clementina_scaffold_34:875164-876822 | -132.378 | 0.0004911 | clementine0.9_001684m | AT1G43850.1 | SEU | SEUSS transcriptional co-regulator |
| 1. clementina_scaffold_8:960441-965237 | -132.222 | 0.0008973 | clementine0.9_002940m | AT1G07380.1 |  | Neutral/alkaline non-lysosomal ceramidase |
| 1. clementina_scaffold_25:3439964-3442630 | -132.015 | 5.03E-02 | clementine0.9_023762m | AT4G35750.1 |  | SEC14 cytosolic factor family protein / phosphoglyceride transfer family protein |
| 1. clementina_scaffold_91:739240-743104 | -131.945 | 7.89E-01 | clementine0.9_029474m |  |  |  |
| 1. clementina_scaffold_11:4653804-4654070 | -131.878 | 0.0002881 | clementine0.9_000677m | AT5G35750.1 | HK2 | histidine kinase 2 |
| 1. clementina_scaffold_19:1024377-1027388 | -131.678 | 1.09E-01 | clementine0.9_002425m | AT1G11720.2 | SS3 | starch synthase 3 |
| 1. clementina_scaffold_32:2249419-2250836 | -130.922 | 2.54E+00 | clementine0.9_013333m | AT2G26530.1 | AR781 | Protein of unknown function (DUF1645) |
| 1. clementina_scaffold_20:2066685-2070238 | -129.537 | 9.65E-01 | clementine0.9_002770m | AT5G17920.1 | ATMS1 | Cobalamin-independent synthase family protein |
| 1. clementina_scaffold_54:428061-428993 | -128.103 | 0.0007466 | clementine0.9_012019m | AT2G39140.1 | SVR1 | pseudouridine synthase family protein |
| 1. clementina_scaffold_22:1126791-1129071 | -126.183 | 0.0005136 | clementine0.9_002964m | AT1G20160.1 | ATSBT5.2 | Subtilisin-like serine endopeptidase family protein |
| 1. clementina_scaffold_10:5409318-5413346 | -125.856 | 4.63E-01 | clementine0.9_000365m | AT3G13530.1 | MAPKKK7 | mitogen-activated protein kinase kinase kinase 7 |
| 1. clementina_scaffold_2:2416391-2419767 | -124.771 | 4.87E+00 | clementine0.9_023416m | AT3G51730.1 |  | saposin B domain-containing protein |
| 1. clementina_scaffold_37:1674589-1676132 | -124.521 | 0.0010018 | clementine0.9_028947m | AT3G14470.1 |  | NB-ARC domain-containing disease resistance protein |
| 1. clementina_scaffold_2:4181169-4184071 | -123.717 | 0.0014573 | clementine0.9_032071m | AT3G12010.1 |  |  |
| 1. clementina_scaffold_2:9010797-9014396 | -123.694 | 0.0007029 | clementine0.9_000915m | AT4G27190.1 |  | NB-ARC domain-containing disease resistance protein |
| 1. clementina_scaffold_26:785451-786065 | -123.581 | 0.0001477 | clementine0.9_019592m | AT4G34215.1 |  | Domain of unknown function (DUF303) |
| 1. clementina_scaffold_28:493928-497320 | -123.551 | 0.0010902 | clementine0.9_001064m | AT4G34830.1 | MRL1 | Pentatricopeptide repeat (PPR) superfamily protein |
| 1. clementina_scaffold_56:998449-1000736 | -123.521 | 0.000433 | clementine0.9_000257m | AT1G71010.1 | FAB1C | FORMS APLOID AND BINUCLEATE CELLS 1C |
| 1. clementina_scaffold_20:1811245-1815379 | -123.087 | 0.0001273 | clementine0.9_034742m | AT1G51740.1 | SYP81 | syntaxin of plants 81 |
| 1. clementina_scaffold_118:248273-251629 | -122.947 | 2.19E+00 | clementine0.9_001548m | AT3G14470.1 |  | NB-ARC domain-containing disease resistance protein |
| 1. clementina_scaffold_23:2356379-2358337 | -122.266 | 0.0008101 | clementine0.9_009560m | AT2G22720.3 |  | SPT2 chromatin protein |
| 1. clementina_scaffold_10:4467980-4474952 | -121.122 | 0 | clementine0.9_012487m | AT3G02540.1 | RAD23C | Rad23 UV excision repair protein family |
| 1. clementina_scaffold_9:1344189-1345329 | -120.848 | 0.0003843 | clementine0.9_025435m | AT5G11760.1 |  |  |
| 1. clementina_scaffold_33:2030371-2033588 | -120.359 | 0.001104 | clementine0.9_004496m | AT1G51965.1 | ABO5 | ABA Overly-Sensitive 5 |
| 1. clementina_scaffold_20:2805896-2810557 | -120.157 | 0.0003923 | clementine0.9_015833m | AT3G27820.1 | MDAR4 | monodehydroascorbate reductase 4 |
| 1. clementina_scaffold_9:1588525-1592437 | -119.332 | 0.0003358 | clementine0.9_031805m | AT2G26100.1 |  | Galactosyltransferase family protein |
| 1. clementina_scaffold_21:2767987-2770632 | -119.255 | 0.0007421 | clementine0.9_002102m | AT1G29400.1 | ML5 | MEI2-like protein 5 |
| 1. clementina_scaffold_15:4271476-4275914 | -119.225 | 0.0015763 | clementine0.9_002391m | AT5G45160.1 |  | Root hair defective 3 GTP-binding protein (RHD3) |
| 1. clementina_scaffold_8:535852-537417 | -117.773 | 2.31E-03 | clementine0.9_005735m | AT3G47340.1 | ASN1 | glutamine-dependent asparagine synthase 1 |
| 1. clementina_scaffold_6:2609945-2610602 | -116.072 | 0.001602 | clementine0.9_002381m | AT5G51430.1 | EYE | conserved oligomeric Golgi complex component-related / COG complex component-related |
| 1. clementina_scaffold_22:978618-982899 | -115.538 | 2.51E+00 | clementine0.9_012088m | AT4G38470.1 |  | ACT-like protein tyrosine kinase family protein |
| 1. clementina_scaffold_23:2516126-2518779 | -115.403 | 0.0010137 | clementine0.9_003268m | AT5G65620.1 |  | Zincin-like metalloproteases family protein |
| 1. clementina_scaffold_8:5792149-5806553 | -115.221 | 4.89E-03 | clementine0.9_017601m | AT4G33520.2 | PAA1 | P-type ATP-ase 1 |
| 1. clementina_scaffold_44:1143179-1145789 | -114.369 | 9.43E+00 | clementine0.9_000162m | AT2G16485.1 |  | nucleic acid binding;zinc ion binding;DNA binding |
| 1. clementina_scaffold_20:573352-577098 | -113.329 | 2.82E-02 | clementine0.9_006051m | AT3G46730.1 |  | NB-ARC domain-containing disease resistance protein |
| 1. clementina_scaffold_39:855621-861480 | -113.327 | 0.0001796 | clementine0.9_002871m | AT5G13690.1 | CYL1 | alpha-N-acetylglucosaminidase family / NAGLU family |
| 1. clementina_scaffold_31:358838-360590 | -113.192 | 0.0010792 | clementine0.9_000925m | AT5G19820.1 | emb2734 | ARM repeat superfamily protein |
| 1. clementina_scaffold_1:2197864-2199459 | -112.338 | 4.27E+00 | clementine0.9_005932m | AT1G31970.1 | STRS1 | DEA(D/H)-box RNA helicase family protein |
| 1. clementina_scaffold_2:5938684-5943377 | -112.148 | 2.94E-05 | clementine0.9_000337m | AT2G36380.1 | PDR6 | pleiotropic drug resistance 6 |
| 1. clementina_scaffold_8:534687-535742 | -111.919 | 0.0009666 | clementine0.9_005735m | AT3G47340.1 | ASN1 | glutamine-dependent asparagine synthase 1 |
| 1. clementina_scaffold_15:3257896-3261993 | -111.829 | 1.02E+00 | clementine0.9_002492m | AT1G30470.1 |  | SIT4 phosphatase-associated family protein |
| 1. clementina_scaffold_3:6257617-6261135 | -110.849 | 4.57E+00 | clementine0.9_005329m | AT3G04810.1 | NEK2 | NIMA-related kinase 2 |
| 1. clementina_scaffold_1:8469667-8473026 | -110.576 | 6.59E-01 | clementine0.9_007233m | AT3G05350.1 |  | Metallopeptidase M24 family protein |
| 1. clementina_scaffold_24:2815108-2819723 | -109.973 | 0.0001217 | clementine0.9_019087m | AT2G22120.2 |  | RING/FYVE/PHD zinc finger superfamily protein |
| 1. clementina_scaffold_40:83155-86513 | -109.716 | 0.0009256 | clementine0.9_011552m | AT2G19560.1 | EER5 | proteasome family protein |
| 1. clementina_scaffold_47:561261-566279 | -109.397 | 8.60E-01 | clementine0.9_001491m | AT2G40840.1 | DPE2 | disproportionating enzyme 2 |
| 1. clementina_scaffold_96:257339-258743 | -109.314 | 0.0005069 | clementine0.9_034591m | AT5G17680.1 |  | disease resistance protein (TIR-NBS-LRR class). putative |
| 1. clementina_scaffold_5:1024600-1025458 | -108.786 | 0.0006832 | clementine0.9_002864m | AT3G27170.1 | CLC-B | chloride channel B |
| 1. clementina_scaffold_1:10553690-10554919 | -107.375 | 3.11E-01 | clementine0.9_024405m | AT4G14960.1 | TUA6 | Tubulin/FtsZ family protein |
| 1. clementina_scaffold_1:8761355-8764101 | -107.222 | 0.0003498 | clementine0.9_017437m | AT3G05410.2 |  | Photosystem II reaction center PsbP family protein |
| 1. clementina_scaffold_21:523164-524316 | -107.067 | 1.41E-01 | clementine0.9_026022m | AT2G05540.1 |  | Glycine-rich protein family |
| 1. clementina_scaffold_3:4649166-4651705 | -106.848 | 0.0005915 | clementine0.9_025337m | AT3G62880.1 | ATOEP16-4 | Mitochondrial import inner membrane translocase subunit Tim17/Tim22/Tim23 family protein |
| 1. clementina_scaffold_12:1986182-1987853 | -106.843 | 0.0007968 | clementine0.9_001756m | AT4G32180.1 | PANK2 | pantothenate kinase 2 |
| 1. clementina_scaffold_92:311855-314392 | -106.364 | 8.47E-01 | clementine0.9_021119m | AT4G11600.1 | GPX6 | glutathione peroxidase 6 |
| 1. clementina_scaffold_4:5137634-5139652 | -106.167 | 0.0004068 | clementine0.9_006028m | AT1G74960.1 | FAB1 | fatty acid biosynthesis 1 |
| 1. clementina_scaffold_26:1970120-1971716 | -106.063 | 0.0003104 | clementine0.9_019247m | AT2G14520.1 |  | CBS domain-containing protein with a domain of unknown function (DUF21) |
| 1. clementina_scaffold_4:3901014-3903423 | -105.335 | 0.0001988 | clementine0.9_006101m | AT1G05500.1 | NTMC2T2.1 | Calcium-dependent lipid-binding (CaLB domain) family protein |
| 1. clementina_scaffold_59:716276-723803 | -105.092 | 0.0010758 | clementine0.9_006488m | AT5G43490.1 |  |  |
| 1. clementina_scaffold_55:67583-70462 | -104.712 | 0.0001796 | clementine0.9_005060m | AT2G21520.1 |  | Sec14p-like phosphatidylinositol transfer family protein |
| 1. clementina_scaffold_4:1713548-1720553 | -104.381 | 1.39E-01 | clementine0.9_000137m | AT4G28080.1 |  | Tetratricopeptide repeat (TPR)-like superfamily protein |
| 1. clementina_scaffold_32:666552-669513 | -104.192 | 0.0001718 | clementine0.9_004290m | AT5G13640.1 | PDAT | phospholipid:diacylglycerol acyltransferase |
| 1. clementina_scaffold_58:363360-364372 | -104.094 | 2.13E-03 | clementine0.9_023328m | AT5G56550.1 | OXS3 | oxidative stress 3 |
| 1. clementina_scaffold_136:72840-74963 | -103.614 | 0.0008251 | clementine0.9_032826m | AT3G18930.1 |  | RING/U-box superfamily protein |
| 1. clementina_scaffold_25:1102419-1106683 | -102.733 | 0.0001149 | clementine0.9_000372m | AT2G23740.2 |  | nucleic acid binding;sequence-specific DNA binding transcription factors;zinc ion binding |
| 1. clementina_scaffold_7:5920523-5923053 | -102.506 | 0.0003407 | clementine0.9_008225m | AT3G06580.1 | GALK | Mevalonate/galactokinase family protein |
| 1. clementina_scaffold_82:139262-140541 | -101.898 | 0.0007708 | clementine0.9_017886m | AT4G32060.1 |  | calcium-binding EF hand family protein |
| 1. clementina_scaffold_21:2114078-2116069 | -101.711 | 0.0002736 | clementine0.9_007324m | AT1G08680.4 | ZIGA4 | ARF GAP-like zinc finger-containing protein ZIGA4 |
| 1. clementina_scaffold_3:6320146-6326376 | -101.402 | 0.0015532 | clementine0.9_001033m | AT3G63340.1 |  | Protein phosphatase 2C family protein |
| 1. clementina_scaffold_34:2343812-2345127 | -101.162 | 0.0013433 | clementine0.9_006944m | AT4G09160.1 |  | SEC14 cytosolic factor family protein / phosphoglyceride transfer family protein |
| 1. clementina_scaffold_7:3220462-3224575 | -100.947 | 0.0003376 | clementine0.9_002969m | AT5G26240.1 | CLC-D | chloride channel D |
| 1. clementina_scaffold_2:4700058-4705911 | -100.558 | 2.29E-05 | clementine0.9_003815m | AT5G22030.1 | UBP8 | ubiquitin-specific protease 8 |
| 1. clementina_scaffold_1:12220894-12222537 | -100.544 | 0.000252 | clementine0.9_004368m | AT1G77990.1 | AST56 | STAS domain / Sulfate transporter family |
| 1. clementina_scaffold_25:1076757-1078928 | -100.432 | 8.97E-02 | clementine0.9_002423m | AT2G23760.1 | BLH4 | BEL1-like homeodomain 4 |
| 1. clementina_scaffold_1:11183867-11185134 | -100.081 | 0.00153 | clementine0.9_019112m | AT3G56770.1 |  | basic helix-loop-helix (bHLH) DNA-binding superfamily protein |
| 1. clementina_scaffold_69:151503-153707 | -100.052 | 0.0011102 | clementine0.9_029441m | AT1G09660.1 |  | RNA-binding KH domain-containing protein |
| 1. clementina_scaffold_1:12437232-12441280 | -25.039 | 2.63E-01 | clementine0.9_000385m | AT1G77800.1 |  | PHD finger family protein |
| 1. clementina_scaffold_8:673742-675996 | -23.989 | 8.56E+00 | clementine0.9_008471m | AT5G58720.1 |  | smr (Small MutS Related) domain-containing protein |
| 1. clementina_scaffold_3:894192-900190 | -21.493 | 9.30E-04 | clementine0.9_000277m | AT2G45540.1 |  | WD-40 repeat family protein / beige-related |
| 1. clementina_scaffold_25:940535-942594 | -19.269 | 0.000167 | clementine0.9_007386m | AT2G23470.1 | RUS4 | Protein of unknown function. DUF647 |
| 1. clementina_scaffold_16:1486772-1493638 | -16.442 | 0.0001105 | clementine0.9_000650m | AT2G39580.1 |  |  |
| 1. clementina_scaffold_11:4870691-4872456 | -15.925 | 1.92E+00 | clementine0.9_000904m | AT1G74170.1 | RLP13 | receptor like protein 13 |
| 1. clementina_scaffold_15:3065555-3069554 | -15.523 | 2.63E-01 | clementine0.9_001011m | AT4G05420.1 | DDB1A | damaged DNA binding protein 1A |
| 1. clementina_scaffold_2:4398254-4401191 | -15.098 | 0.000585 | clementine0.9_002177m | AT3G56150.1 | EIF3C | eukaryotic translation initiation factor 3C |
| 1. clementina_scaffold_28:2409800-2410309 | -15.043 | 0.0008623 | clementine0.9_032818m |  |  |  |
| 1. clementina_scaffold_10:988651-991414 | -13.514 | 0.000155 | clementine0.9_002470m | AT1G23870.1 | TPS9 | trehalose-phosphatase/synthase 9 |
| 1. clementina_scaffold_35:1126885-1129861 | -12.831 | 2.89E-01 | clementine0.9_033600m | AT3G24080.1 |  | KRR1 family protein |
| 1. clementina_scaffold_3:6291511-6293524 | -12.423 | 2.73E-03 | clementine0.9_019639m | AT4G14720.1 | PPD2 | TIFY domain/Divergent CCT motif family protein |
| 1. clementina_scaffold_9:3009367-3016164 | -12.186 | 0.0013292 | clementine0.9_012702m | AT3G24430.1 | HCF101 | ATP binding |
| 1. clementina_scaffold_20:2745285-2749244 | -11.066 | 0.0008366 | clementine0.9_006241m | AT5G14720.1 |  | Protein kinase superfamily protein |
| 1. clementina_scaffold_15:4029088-4031926 | -10.995 | 7.76E+00 | clementine0.9_001130m | AT1G56130.1 |  | Leucine-rich repeat transmembrane protein kinase |
| 1. clementina_scaffold_38:432390-434527 | -10.298 | 2.59E-02 | clementine0.9_008687m | AT3G04650.1 |  | FAD/NAD(P)-binding oxidoreductase family protein |
| 1. clementina_scaffold_41:546406-548923 | -10.102 | 0.0003848 | clementine0.9_017276m | AT2G30530.1 |  |  |
| 1. clementina_scaffold_1:9332399-9334731 | -3.475 | 4.54E-05 | clementine0.9_000869m | AT5G27030.1 | TPR3 | TOPLESS-related 3 |
| 1. clementina_scaffold_6:111818-119133 | -1.442 | 1.94E+00 | clementine0.9_000654m | AT5G46330.1 | FLS2 | Leucine-rich receptor-like protein kinase family protein |
| 1. clementina_scaffold_7:3019564-3020573 | -1.224 | 0.0002625 | clementine0.9_002263m | AT5G02830.1 |  | Tetratricopeptide repeat (TPR)-like superfamily protein |
| 1. clementina_scaffold_74:1193244-1197818 | -0.987121 | 3.92E+00 | clementine0.9_002185m | AT5G04460.1 |  | RING/U-box superfamily protein |
| 1. clementina_scaffold_25:367735-370567 | -0.976513 | 0.0001214 | clementine0.9_019108m | AT4G36960.1 |  | RNA-binding (RRM/RBD/RNP motifs) family protein |
| 1. clementina_scaffold_3:5435653-5440891 | -0.974186 | 3.53E-05 | clementine0.9_004551m | AT2G48010.1 | RKF3 | receptor-like kinase in in flowers 3 |
| 1. clementina_scaffold_23:2353097-2355604 | -0.974052 | 1.09E+00 | clementine0.9_009560m | AT2G22720.3 |  | SPT2 chromatin protein |
| 1. clementina_scaffold_22:1219057-1221990 | -0.9657 | 2.38E-08 | clementine0.9_000638m | AT1G27940.1 | PGP13 | P-glycoprotein 13 |
| 1. clementina_scaffold_34:1236936-1244282 | -0.96171 | 1.71E-02 | clementine0.9_000401m | AT1G76810.1 |  | eukaryotic translation initiation factor 2 (eIF-2) family protein |
| 1. clementina_scaffold_85:808591-812796 | -0.9593 | 3.33E-10 | clementine0.9_011401m | AT3G07300.1 |  | NagB/RpiA/CoA transferase-like superfamily protein |
| 1. clementina_scaffold_3:1724844-1728366 | -0.957674 | 6.27E-06 | clementine0.9_008792m | AT1G01540.2 |  | Protein kinase superfamily protein |
| 1. clementina_scaffold_133:36476-40022 | -0.952398 | 0.0002344 | clementine0.9_001091m | AT4G27220.1 |  | NB-ARC domain-containing disease resistance protein |
| 1. clementina_scaffold_15:3298464-3302146 | -0.951732 | 3.44E+00 | clementine0.9_002251m | AT2G34710.1 | PHB | Homeobox-leucine zipper family protein / lipid-binding START domain-containing protein |
| 1. clementina_scaffold_12:516277-519727 | -0.943158 | 2.92E-01 | clementine0.9_006579m | AT4G17090.1 | CT-BMY | chloroplast beta-amylase |
| 1. clementina_scaffold_134:30736-33602 | -0.942551 | 0.0005772 | clementine0.9_021594m | AT4G31040.1 |  | CemA-like proton extrusion protein-related |
| 1. clementina_scaffold_10:2139308-2143564 | -0.938685 | 0.0007745 | clementine0.9_007919m | AT3G26020.1 |  | Protein phosphatase 2A regulatory B subunit family protein |
| 1. clementina_scaffold_3:5060868-5062385 | -0.936763 | 1.22E-01 | clementine0.9_003979m | AT4G03200.2 |  | catalytics |
| 1. clementina_scaffold_19:2256342-2259486 | -0.936319 | 9.75E-02 | clementine0.9_018911m | AT1G68810.1 |  | basic helix-loop-helix (bHLH) DNA-binding superfamily protein |
| 1. clementina_scaffold_2:9208804-9213532 | -0.935573 | 0.0010136 | clementine0.9_005398m | AT4G24290.2 |  | MAC/Perforin domain-containing protein |
| 1. clementina_scaffold_8:2805783-2816853 | -0.931578 | 0.0003522 | clementine0.9_002322m | AT5G60410.3 | SIZ1 | DNA-binding protein with MIZ/SP-RING zinc finger. PHD-finger and SAP domain |
| 1. clementina_scaffold_14:2878463-2879963 | -0.931276 | 2.01E+00 | clementine0.9_032568m | AT1G66240.1 | ATX1 | homolog of anti-oxidant 1 |
| 1. clementina_scaffold_104:78840-82786 | -0.929498 | 1.77E-02 | clementine0.9_006412m | AT3G07130.1 | PAP15 | purple acid phosphatase 15 |
| 1. clementina_scaffold_4:7696470-7696578 | -0.925246 | 2.65E+00 | clementine0.9_032838m | AT2G34930.1 |  | disease resistance family protein / LRR family protein |
| 1. clementina_scaffold_96:851652-854578 | -0.923559 | 0.001348 | clementine0.9_000436m | AT3G14460.1 |  | LRR and NB-ARC domains-containing disease resistance protein |
| 1. clementina_scaffold_82:838812-841190 | -0.916478 | 0.0014853 | clementine0.9_010052m | AT5G11330.1 |  | FAD/NAD(P)-binding oxidoreductase family protein |
| 1. clementina_scaffold_1:9383047-9389856 | -0.908722 | 2.99E+00 | clementine0.9_002443m | AT3G05670.1 |  | RING/U-box protein |
| 1. clementina_scaffold_134:46953-50039 | -0.9077 | 0.0012502 | clementine0.9_034279m | AT1G79940.1 | ATERDJ2A | DnaJ / Sec63 Brl domains-containing protein |
| 1. clementina_scaffold_2:4335657-4337024 | -0.903065 | 0.0002116 | clementine0.9_014053m | AT2G44130.1 |  | Galactose oxidase/kelch repeat superfamily protein |
| 1. clementina_scaffold_45:570077-575754 | -0.898833 | 3.75E+00 | clementine0.9_006634m | AT5G46840.1 |  | RNA-binding (RRM/RBD/RNP motifs) family protein |
| 1. clementina_scaffold_1:436849-439663 | -0.89789 | 0.0004582 | clementine0.9_002614m | AT2G34300.1 |  | S-adenosyl-L-methionine-dependent methyltransferases superfamily protein |
| 1. clementina_scaffold_24:3202710-3205455 | -0.896604 | 6.64E-04 | clementine0.9_003542m | AT4G39680.1 |  | SAP domain-containing protein |
| 1. clementina_scaffold_14:1452450-1457147 | -0.881643 | 0.0006372 | clementine0.9_000670m | AT5G49220.1 |  | Protein of unknown function (DUF789) |
| 1. clementina_scaffold_12:916815-921809 | -0.875853 | 0.0008943 | clementine0.9_011390m | AT5G63380.1 |  | AMP-dependent synthetase and ligase family protein |
| 1. clementina_scaffold_7:2065715-2069055 | -0.87504 | 1.66E-02 | clementine0.9_021434m | AT2G22540.1 | SVP | K-box region and MADS-box transcription factor family protein |
| 1. clementina_scaffold_9:3362149-3366101 | -0.868475 | 0.0009959 | clementine0.9_001692m | AT4G27190.1 |  | NB-ARC domain-containing disease resistance protein |
| 1. clementina_scaffold_2:959206-960913 | -0.862437 | 0.0011498 | clementine0.9_009519m | AT2G38120.1 | AUX1 | Transmembrane amino acid transporter family protein |
| 1. clementina_scaffold_10:4223131-4224551 | -0.85965 | 0.000779 | clementine0.9_013116m | AT1G68840.1 | RAV2 | related to ABI3/VP1 2 |
| 1. clementina_scaffold_10:3445451-3449355 | -0.859385 | 0.0002284 | clementine0.9_017593m | AT2G03780.1 |  | Translin family protein |
| 1. clementina_scaffold_33:335716-339035 | -0.859067 | 6.78E-03 | clementine0.9_011137m | AT1G80400.1 |  | RING/U-box superfamily protein |
| 1. clementina_scaffold_151:141189-143916 | -0.85616 | 0.0001187 | clementine0.9_015308m | AT2G44420.1 |  | protein N-terminal asparagine amidohydrolase family protein |
| 1. clementina_scaffold_80:1024018-1025710 | -0.854876 | 7.90E+00 | clementine0.9_014637m | AT5G15780.1 |  | Pollen Ole e 1 allergen and extensin family protein |
| 1. clementina_scaffold_1:9179550-9182234 | -0.852908 | 7.80E+00 | clementine0.9_024629m | AT3G18760.1 |  | Translation elongation factor EF1B/ribosomal protein S6 family protein |
| 1. clementina_scaffold_4:6441693-6446825 | -0.852245 | 9.79E-01 | clementine0.9_004054m | AT5G55600.1 |  | agenet domain-containing protein / bromo-adjacent homology (BAH) domain-containing protein |
| 1. clementina_scaffold_4:6044243-6046870 | -0.847488 | 0.0011308 | clementine0.9_017261m | AT5G55970.1 |  | RING/U-box superfamily protein |
| 1. clementina_scaffold_79:904206-909113 | -0.843709 | 2.00E-02 | clementine0.9_011509m | AT3G21820.1 | ATXR2 | histone-lysine N-methyltransferase ATXR2 |
| 1. clementina_scaffold_1:7439081-7442015 | -0.840363 | 0.0012477 | clementine0.9_031979m | AT5G27350.1 | SFP1 | Major facilitator superfamily protein |
| 1. clementina_scaffold_25:2158761-2160889 | -0.833989 | 6.45E-02 | clementine0.9_007698m | AT4G36220.1 | FAH1 | ferulic acid 5-hydroxylase 1 |
| 1. clementina_scaffold_1:4049702-4051005 | -0.833704 | 0.0005369 | clementine0.9_012636m | AT1G31350.1 | KUF1 | KAR-UP F-box 1 |
| 1. clementina_scaffold_4:3024748-3032246 | -0.833464 | 2.15E-06 | clementine0.9_003246m | AT4G30020.1 |  | PA-domain containing subtilase family protein |
| 1. clementina_scaffold_21:1408651-1412961 | -0.832204 | 0.0002127 | clementine0.9_004846m | AT2G32540.1 | CSLB04 | cellulose synthase-like B4 |
| 1. clementina_scaffold_5:786275-786592 | -0.830282 | 0.0001597 | clementine0.9_027281m | AT5G50460.1 |  | secE/sec61-gamma protein transport protein |
| 1. clementina_scaffold_9:4081884-4089022 | -0.828299 | 2.00E-01 | clementine0.9_021480m | AT1G80940.1 |  |  |
| 1. clementina_scaffold_20:3868102-3871885 | -0.826861 | 9.40E-02 | clementine0.9_011826m | AT2G20570.1 | GPRI1 | GBF\'s pro-rich region-interacting factor 1 |
| 1. clementina_scaffold_65:814999-818765 | -0.823231 | 0.0002249 | clementine0.9_001207m | AT1G76390.1 |  | ARM repeat superfamily protein |
| 1. clementina_scaffold_71:146754-149125 | -0.817632 | 0.0016919 | clementine0.9_012620m | AT1G67300.2 |  | Major facilitator superfamily protein |
| 1. clementina_scaffold_20:629499-632839 | -0.817552 | 0.0009853 | clementine0.9_003883m | AT3G21090.1 |  | ABC-2 type transporter family protein |
| 1. clementina_scaffold_3:6729370-6730701 | -0.817418 | 0.0005505 | clementine0.9_017412m | AT2G30620.2 |  | winged-helix DNA-binding transcription factor family protein |
| 1. clementina_scaffold_19:3106423-3109956 | -0.813998 | 0.0006692 | clementine0.9_029820m | AT2G47960.1 |  |  |
| 1. clementina_scaffold_64:189858-192983 | -0.812797 | 0.0009532 | clementine0.9_001787m | AT5G54730.1 | G18F | homolog of yeast autophagy 18 (ATG18) F |
| 1. clementina_scaffold_1:4485923-4491718 | -0.810742 | 0.0002354 | clementine0.9_018898m | AT4G19420.1 |  | Pectinacetylesterase family protein |
| 1. clementina_scaffold_21:879175-882512 | -0.809089 | 4.27E+00 | clementine0.9_004499m | AT2G32970.2 |  |  |
| 1. clementina_scaffold_25:2303442-2306461 | -0.808595 | 0.0004969 | clementine0.9_009908m | AT1G64080.1 |  |  |
| 1. clementina_scaffold_3:5407826-5412733 | -0.803461 | 5.31E+00 | clementine0.9_001399m | AT1G03380.1 | ATG18G | homolog of yeast autophagy 18 (ATG18) G |
| 1. clementina_scaffold_3:208819-212707 | -0.80209 | 4.03E+00 | clementine0.9_014112m | AT3G60510.1 |  | ATP-dependent caseinolytic (Clp) protease/crotonase family protein |
| 1. clementina_scaffold_33:1961752-1962902 | -0.80103 | 0.0016004 | clementine0.9_022514m | AT5G56550.1 | OXS3 | oxidative stress 3 |
| 1. clementina_scaffold_112:564413-572693 | -0.800902 | 5.53E-03 | clementine0.9_035821m | AT3G47570.1 |  | Leucine-rich repeat protein kinase family protein |
| 1. clementina_scaffold_12:1460661-1462514 | -0.800319 | 0.0009815 | clementine0.9_015981m | AT4G24730.2 |  | Calcineurin-like metallo-phosphoesterase superfamily protein |
| 1. clementina_scaffold_22:613033-617489 | -0.79789 | 0.0003886 | clementine0.9_030267m | AT4G35090.1 | CAT2 | catalase 2 |
| 1. clementina_scaffold_18:782599-785844 | -0.796239 | 1.09E-01 | clementine0.9_029196m | AT5G54160.1 | OMT1 | O-methyltransferase 1 |
| 1. clementina_scaffold_1:9513551-9516389 | -0.794713 | 0.0001948 | clementine0.9_004851m | AT2G47430.1 | CKI1 | Signal transduction histidine kinase |
| 1. clementina_scaffold_80:703080-705997 | -0.794469 | 6.06E-02 | clementine0.9_011156m | AT1G13570.1 |  | F-box/RNI-like superfamily protein |
| 1. clementina_scaffold_15:2880394-2884731 | -0.793837 | 0.0006835 | clementine0.9_001574m | AT1G17210.1 | ILP1 | IAP-like protein 1 |
| 1. clementina_scaffold_20:114926-119640 | -0.791631 | 1.29E-02 | clementine0.9_004414m | AT5G19620.1 | OEP80 | outer envelope protein of 80 kDa |
| 1. clementina_scaffold_91:849151-853332 | -0.780761 | 0.000781 | clementine0.9_000074m | AT3G54280.1 | RGD3 | DNA binding;ATP binding;nucleic acid binding;binding;helicases;ATP binding;DNA binding;helicases |
| 1. clementina_scaffold_33:1925423-1928000 | -0.780433 | 5.42E+00 | clementine0.9_019699m | AT3G16500.1 | PAP1 | phytochrome-associated protein 1 |
| 1. clementina_scaffold_84:811300-814729 | -0.778229 | 0.0006668 | clementine0.9_002227m | AT5G61960.1 | ML1 | MEI2-like protein 1 |
| 1. clementina_scaffold_26:2958946-2960236 | -0.777007 | 0.0013979 | clementine0.9_017463m | AT3G12500.1 | HCHIB | basic chitinase |
| 1. clementina_scaffold_19:205945-209397 | -0.775084 | 0.0012812 | clementine0.9_005306m | AT5G47750.1 | D6PKL2 | D6 protein kinase like 2 |
| 1. clementina_scaffold_7:5942791-5946021 | -0.773423 | 8.29E+00 | clementine0.9_030642m | AT1G16060.1 | ADAP | ARIA-interacting double AP2 domain protein |
| 1. clementina_scaffold_3:326411-333544 | -0.77151 | 5.31E+00 | clementine0.9_000037m | AT1G02080.2 |  | transcription regulators |
| 1. clementina_scaffold_114:258891-266324 | -0.756997 | 4.65E-01 | clementine0.9_003624m | AT5G20660.1 |  | Zn-dependent exopeptidases superfamily protein |
| 1. clementina_scaffold_20:3182114-3186320 | -0.756363 | 0.0006203 | clementine0.9_000017m | AT5G40450.1 |  |  |
| 1. clementina_scaffold_28:867632-870276 | -0.755929 | 0.0003635 | clementine0.9_005826m | AT2G21140.1 | PRP2 | proline-rich protein 2 |
| 1. clementina_scaffold_4:1845272-1847722 | -0.755749 | 4.06E+00 | clementine0.9_008414m | AT4G28070.2 |  | AFG1-like ATPase family protein |
| 1. clementina_scaffold_54:194206-199044 | -0.755436 | 0.0005273 | clementine0.9_029676m | AT5G04290.1 | KTF1 | kow domain-containing transcription factor 1 |
| 1. clementina_scaffold_34:1311146-1314157 | -0.755078 | 0.0005279 | clementine0.9_005316m | AT1G76890.2 | GT2 | Duplicated homeodomain-like superfamily protein |
| 1. clementina_scaffold_36:2077644-2081907 | -0.749339 | 0.0015118 | clementine0.9_014393m | AT1G10940.1 |  | Protein kinase superfamily protein |
| 1. clementina_scaffold_2:3746180-3748518 | -0.748464 | 1.04E+00 | clementine0.9_024385m | AT2G42220.1 |  | Rhodanese/Cell cycle control phosphatase superfamily protein |
| 1. clementina_scaffold_17:1737245-1741562 | -0.748421 | 5.65E-01 | clementine0.9_005153m | AT2G42880.1 | MPK20 | MAP kinase 20 |
| 1. clementina_scaffold_15:1444163-1447261 | -0.741385 | 0.0010172 | clementine0.9_004748m | AT2G02080.1 | IDD4 | indeterminate(ID)-domain 4 |
| 1. clementina_scaffold_4:2624000-2629633 | -0.739866 | 0.0007628 | clementine0.9_003654m | AT4G30210.2 | ATR2 | P450 reductase 2 |
| 1. clementina_scaffold_4:1581966-1583821 | -0.739851 | 7.29E-01 | clementine0.9_001159m | AT5G17420.1 | IRX3 | Cellulose synthase family protein |
| 1. clementina_scaffold_31:529971-531514 | -0.739383 | 0.0006195 | clementine0.9_014881m | AT1G15670.1 |  | Galactose oxidase/kelch repeat superfamily protein |
| 1. clementina_scaffold_36:2105465-2112967 | -0.738229 | 2.54E-07 | clementine0.9_000725m | AT1G22930.1 |  | T-complex protein 11 |
| 1. clementina_scaffold_2:5761372-5762683 | -0.738208 | 0.0006468 | clementine0.9_014909m | AT2G27310.1 |  | F-box family protein |
| 1. clementina_scaffold_25:1385616-1391045 | -0.737607 | 4.93E-06 | clementine0.9_005650m | AT4G36690.1 | ATU2AF65A | U2 snRNP auxilliary factor. large subunit. splicing factor |
| 1. clementina_scaffold_39:1601197-1604990 | -0.735412 | 5.82E+00 | clementine0.9_010045m | AT1G13960.1 | WRKY4 | WRKY DNA-binding protein 4 |
| 1. clementina_scaffold_7:5403239-5403977 | -0.734284 | 0.0007857 | clementine0.9_002609m | AT3G48110.1 | EDD1 | glycine-tRNA ligases |
| 1. clementina_scaffold_22:2259494-2263388 | -0.727716 | 0.0006339 | clementine0.9_004283m | AT2G16250.1 |  | Leucine-rich repeat protein kinase family protein |
| 1. clementina_scaffold_86:541793-542659 | -0.724262 | 0.0011637 | clementine0.9_021895m | AT3G15210.1 | ERF4 | ethylene responsive element binding factor 4 |
| 1. clementina_scaffold_79:738966-743739 | -0.721675 | 0.0005228 | clementine0.9_008320m | AT4G15240.1 |  | Protein of unknown function (DUF604) |
| 1. clementina_scaffold_2:3456389-3460675 | -0.721151 | 1.25E-03 | clementine0.9_002742m | AT2G32250.1 | FRS2 | FAR1-related sequence 2 |
| 1. clementina_scaffold_8:4691629-4694247 | -0.715695 | 0.0001501 | clementine0.9_004476m | AT5G22640.1 | emb1211 | MORN (Membrane Occupation and Recognition Nexus) repeat-containing protein |
| 1. clementina_scaffold_9:1160975-1165629 | -0.713118 | 0.0013707 | clementine0.9_000426m | AT5G11700.2 |  |  |
| 1. clementina_scaffold_5:3958939-3970130 | -0.712482 | 1.21E-05 | clementine0.9_027203m |  |  |  |
| 1. clementina_scaffold_4:6875881-6884253 | -0.711047 | 3.24E-03 | clementine0.9_002475m | AT5G55310.1 | TOP1BETA | DNA topoisomerase 1 beta |
| 1. clementina_scaffold_1:12789985-12793087 | -0.710428 | 0.0004248 | clementine0.9_012129m | AT4G08310.1 |  |  |
| 1. clementina_scaffold_15:4491956-4497012 | -0.710255 | 0.000524 | clementine0.9_000457m | AT2G35110.2 | GRL | transcription activators |
| 1. clementina_scaffold_4:5475521-5485099 | -0.707934 | 0.0004053 | clementine0.9_003287m | AT5G56290.1 | PEX5 | peroxin 5 |
| 1. clementina_scaffold_27:1083360-1089967 | -0.706178 | 1.53E-05 | clementine0.9_000996m | AT5G43630.1 | TZP | zinc knuckle (CCHC-type) family protein |
| 1. clementina_scaffold_61:841267-842646 | -0.702727 | 8.00E+00 | clementine0.9_026688m |  |  |  |
| 1. clementina_scaffold_32:167204-170153 | -0.700213 | 5.10E-07 | clementine0.9_019175m | AT4G33000.1 | CBL10 | calcineurin B-like protein 10 |
| 1. clementina_scaffold_12:4232302-4236618 | -0.694108 | 6.16E+00 | clementine0.9_005277m | AT1G64430.1 |  | Pentatricopeptide repeat (PPR) superfamily protein |
| 1. clementina_scaffold_12:3184704-3188696 | -0.690727 | 0.001745 | clementine0.9_006996m | AT5G23850.1 |  | Arabidopsis thaliana protein of unknown function (DUF821) |
| 1. clementina_scaffold_8:3110387-3113838 | -0.685992 | 9.20E-02 | clementine0.9_009364m | AT2G28380.1 | DRB2 | dsRNA-binding protein 2 |
| 1. clementina_scaffold_20:731923-739806 | -0.684736 | 3.70E-03 | clementine0.9_031627m | AT3G28890.1 | RLP43 | receptor like protein 43 |
| 1. clementina_scaffold_29:2249456-2254799 | -0.679822 | 0.0009323 | clementine0.9_006409m | AT5G61380.1 | TOC1 | CCT motif -containing response regulator protein |
| 1. clementina_scaffold_8:3127952-3132995 | -0.671541 | 0.0001458 | clementine0.9_002294m | AT5G60690.1 | REV | Homeobox-leucine zipper family protein / lipid-binding START domain-containing protein |
| 1. clementina_scaffold_40:157416-160879 | -0.671381 | 0.0007673 | clementine0.9_003023m | AT4G26140.1 | BGAL12 | beta-galactosidase 12 |
| 1. clementina_scaffold_4:5514758-5518243 | -0.671012 | 0.0002751 | clementine0.9_006525m | AT5G56270.1 | WRKY2 | WRKY DNA-binding protein 2 |
| 1. clementina_scaffold_53:1089038-1091631 | -0.668615 | 0.0009962 | clementine0.9_001489m | AT2G27880.1 | ago/05 | Argonaute family protein |
| 1. clementina_scaffold_10:3889885-3895575 | -0.66795 | 0.0014815 | clementine0.9_003189m | AT1G68690.1 |  | Protein kinase superfamily protein |
| 1. clementina_scaffold_27:3111007-3114821 | -0.666413 | 6.05E+00 | clementine0.9_011981m | AT2G33410.1 |  | RNA-binding (RRM/RBD/RNP motifs) family protein |
| 1. clementina_scaffold_1:1025190-1028703 | -0.665404 | 1.01E-01 | clementine0.9_005238m | AT3G20770.1 | EIN3 | Ethylene insensitive 3 family protein |
| 1. clementina_scaffold_4:3688509-3691427 | -0.664532 | 0.0001246 | clementine0.9_011813m | AT3G13050.1 |  | Major facilitator superfamily protein |
| 1. clementina_scaffold_10:1491850-1497092 | -0.66352 | 0.0012664 | clementine0.9_003831m | AT1G70620.3 |  | cyclin-related |
| 1. clementina_scaffold_8:4438524-4440457 | -0.659951 | 4.66E-01 | clementine0.9_003007m | AT4G03230.1 |  | S-locus lectin protein kinase family protein |
| 1. clementina_scaffold_12:5052585-5057572 | -0.65879 | 2.43E+00 | clementine0.9_002756m | AT1G76890.2 | GT2 | Duplicated homeodomain-like superfamily protein |
| 1. clementina_scaffold_11:2812758-2815779 | -0.658265 | 0.0012823 | clementine0.9_000739m | AT5G23630.1 | PDR2 | phosphate deficiency response 2 |
| 1. clementina_scaffold_22:2327863-2330990 | -0.655313 | 5.66E-02 | clementine0.9_033353m |  |  |  |
| 1. clementina_scaffold_2:2681769-2686652 | -0.653949 | 7.36E+00 | clementine0.9_005082m | AT3G52850.1 | VSR1 | vacuolar sorting receptor homolog 1 |
| 1. clementina_scaffold_23:2139858-2146952 | -0.652177 | 4.05E-01 | clementine0.9_000937m | AT5G65770.1 | LINC4 | little nuclei4 |
| 1. clementina_scaffold_9:168936-173308 | -0.649137 | 0.0002044 | clementine0.9_002111m | AT5G11430.1 |  | SPOC domain / Transcription elongation factor S-II protein |
| 1. clementina_scaffold_4:3559425-3567999 | -0.642772 | 4.35E-03 | clementine0.9_003027m | AT2G07050.1 | CAS1 | cycloartenol synthase 1 |
| 1. clementina_scaffold_60:1128514-1133327 | -0.641169 | 3.57E+00 | clementine0.9_011057m | AT1G05055.1 | GTF2H2 | general transcription factor II H2 |
| 1. clementina_scaffold_7:272264-276583 | -0.638165 | 0.000227 | clementine0.9_003450m | AT1G06150.1 | EMB1444 | basic helix-loop-helix (bHLH) DNA-binding superfamily protein |
| 1. clementina_scaffold_41:636021-639374 | -0.63173 | 5.51E+00 | clementine0.9_006837m | AT2G30520.1 | RPT2 | Phototropic-responsive NPH3 family protein |
| 1. clementina_scaffold_16:1158814-1166433 | -0.627763 | 0.0007548 | clementine0.9_003787m | AT5G13550.1 | SULTR4;1 | sulfate transporter 4.1 |
| 1. clementina_scaffold_53:1332052-1335849 | -0.62448 | 0.0010263 | clementine0.9_000554m | AT5G15020.2 | SNL2 | SIN3-like 2 |
| 1. clementina_scaffold_3:3510756-3512912 | -0.620902 | 0.0012356 | clementine0.9_025516m | AT3G15580.1 | APG8H | Ubiquitin-like superfamily protein |
| 1. clementina_scaffold_53:227887-231345 | -0.61983 | 0.0008 | clementine0.9_000154m | AT5G15540.1 | EMB2773 | PHD finger family protein |
| 1. clementina_scaffold_22:2289382-2291613 | -0.617874 | 0.0006822 | clementine0.9_008842m | AT1G47330.1 |  | CBS domain-containing protein with a domain of unknown function (DUF21) |
| 1. clementina_scaffold_4:3279797-3292024 | -0.612919 | 6.07E+00 | clementine0.9_001065m | AT5G57110.1 | ACA8 | autoinhibited Ca2+ -ATPase. isoform 8 |
| 1. clementina_scaffold_3:4552548-4555712 | -0.610381 | 0.000262 | clementine0.9_031143m | AT4G02340.1 |  | alpha/beta-Hydrolases superfamily protein |
| 1. clementina_scaffold_3:4112632-4118921 | -0.608391 | 0.0016846 | clementine0.9_002988m | AT1G02640.1 | BXL2 | beta-xylosidase 2 |
| 1. clementina_scaffold_5:7283449-7286104 | -0.597674 | 0.0007334 | clementine0.9_026941m | AT4G28240.1 |  | Wound-responsive family protein |
| 1. clementina_scaffold_151:98166-104763 | -0.596605 | 0.0010052 | clementine0.9_000095m | AT1G05570.1 | CALS1 | callose synthase 1 |
| 1. clementina_scaffold_1:7445346-7450688 | -0.595702 | 2.43E-03 | clementine0.9_007659m | AT3G05165.1 |  | Major facilitator superfamily protein |
| 1. clementina_scaffold_3:3192111-3198508 | -0.590693 | 0.0008294 | clementine0.9_000633m | AT3G62010.1 |  |  |
| 1. clementina_scaffold_9:2474011-2477080 | -0.588866 | 5.87E+00 | clementine0.9_014927m | AT3G25910.1 |  | Protein of unknown function (DUF1644) |
| 1. clementina_scaffold_9:513137-517014 | -0.58836 | 0.0013798 | clementine0.9_017392m | AT5G11520.1 | ASP3 | aspartate aminotransferase 3 |
| 1. clementina_scaffold_56:815322-818771 | -0.587429 | 0.0002035 | clementine0.9_011822m | AT2G01470.1 | STL2P | SEC12P-like 2 protein |
| 1. clementina_scaffold_3:8580370-8584051 | -0.586841 | 4.58E+00 | clementine0.9_035959m | AT3G14470.1 |  | NB-ARC domain-containing disease resistance protein |
| 1. clementina_scaffold_9:254744-260484 | -0.577744 | 1.49E+00 | clementine0.9_016107m | AT5G25560.1 |  | CHY-type/CTCHY-type/RING-type Zinc finger protein |
| 1. clementina_scaffold_4:6080196-6083476 | -0.576889 | 7.62E+00 | clementine0.9_019837m | AT3G13224.1 |  | RNA-binding (RRM/RBD/RNP motifs) family protein |
| 1. clementina_scaffold_29:1798783-1808516 | -0.576034 | 0.0002432 | clementine0.9_028435m | AT5G61150.1 | VIP4 | leo1-like family protein |
| 1. clementina_scaffold_37:1234206-1240621 | -0.575495 | 1.36E+00 | clementine0.9_022760m | AT1G10890.1 |  |  |
| 1. clementina_scaffold_12:1522477-1526971 | -0.575016 | 7.28E-01 | clementine0.9_002593m | AT4G24690.1 |  | ubiquitin-associated (UBA)/TS-N domain-containing protein / octicosapeptide/Phox/Bemp1 (PB1) domain-containing protein |
| 1. clementina_scaffold_24:209260-213970 | -0.569282 | 0.0003997 | clementine0.9_004520m | AT3G18770.1 |  | Autophagy-related protein 13 |
| 1. clementina_scaffold_47:142996-145838 | -0.566031 | 0.0007448 | clementine0.9_012412m | AT3G02720.1 |  | Class I glutamine amidotransferase-like superfamily protein |
| 1. clementina_scaffold_8:2323751-2333402 | -0.560131 | 0.000384 | clementine0.9_001343m | AT3G45780.1 | PHOT1 | phototropin 1 |
| 1. clementina_scaffold_24:764418-768714 | -0.55918 | 0.0004535 | clementine0.9_002601m | AT5G05170.1 | CEV1 | Cellulose synthase family protein |
| 1. clementina_scaffold_120:270024-275905 | -0.549456 | 6.79E-02 | clementine0.9_011457m | AT5G16810.1 |  | Protein kinase superfamily protein |
| 1. clementina_scaffold_3:6293633-6296203 | -0.548968 | 0.0016912 | clementine0.9_022116m | AT4G14713.1 | PPD1 | TIFY domain/Divergent CCT motif family protein |
| 1. clementina_scaffold_2:6161962-6164581 | -0.543483 | 0.0002684 | clementine0.9_016017m | AT5G42820.1 | U2AF35B | Zinc finger C-x8-C-x5-C-x3-H type family protein |
| 1. clementina_scaffold_15:2810591-2814550 | -0.540066 | 0.0012548 | clementine0.9_005967m | AT1G30220.1 | INT2 | inositol transporter 2 |
| 1. clementina_scaffold_8:1518170-1523872 | -0.536805 | 0.001641 | clementine0.9_003934m | AT1G07540.1 | TRFL2 | TRF-like 2 |
| 1. clementina_scaffold_26:3286581-3291766 | -0.531699 | 0.0008623 | clementine0.9_005045m | AT4G35290.1 | GLUR2 | glutamate receptor 2 |
| 1. clementina_scaffold_12:1811347-1815030 | -0.529423 | 3.42E+00 | clementine0.9_000378m | AT4G24680.1 | MOS1 | modifier of snc1 |
| 1. clementina_scaffold_80:1082798-1086282 | -0.525648 | 0.000117 | clementine0.9_004799m | AT2G03890.1 | PI4K GAMMA 7 | phosphoinositide 4-kinase gamma 7 |
| 1. clementina_scaffold_7:4264193-4269833 | -0.52469 | 0.0001014 | clementine0.9_013697m | AT3G06330.3 |  | RING/U-box superfamily protein |
| 1. clementina_scaffold_39:609757-615091 | -0.522038 | 2.82E+00 | clementine0.9_005556m | AT3G63520.1 | CCD1 | carotenoid cleavage dioxygenase 1 |
| 1. clementina_scaffold_25:530115-535185 | -0.519668 | 0.0007748 | clementine0.9_014280m | AT4G37040.1 | MAP1D | methionine aminopeptidase 1D |
| 1. clementina_scaffold_2:184962-187511 | -0.517577 | 0.0014772 | clementine0.9_024148m | AT5G02020.1 | SIS |  |
| 1. clementina_scaffold_12:3924335-3927301 | -0.515655 | 8.59E+00 | clementine0.9_016818m | AT4G23730.1 |  | Galactose mutarotase-like superfamily protein |
| 1. clementina_scaffold_34:1300458-1303697 | -0.51408 | 0.0003886 | clementine0.9_005040m | AT1G76880.1 |  | Duplicated homeodomain-like superfamily protein |
| 1. clementina_scaffold_23:3428645-3433179 | -0.506655 | 0.0003657 | clementine0.9_003739m | AT1G33420.1 |  | RING/FYVE/PHD zinc finger superfamily protein |
| 1. clementina_scaffold_15:3412613-3418082 | -0.500106 | 2.01E-01 | clementine0.9_014227m | AT4G20930.1 |  | 6-phosphogluconate dehydrogenase family protein |
| 1. clementina_scaffold_37:1678192-1681163 | -0.498306 | 0.0001049 | clementine0.9_027103m | AT5G24690.1 |  | Protein of unknown function (DUF3411) |
| 1. clementina_scaffold_12:4236984-4242181 | -0.496297 | 6.89E-02 | clementine0.9_005277m | AT1G64430.1 |  | Pentatricopeptide repeat (PPR) superfamily protein |
| 1. clementina_scaffold_22:520497-522727 | -0.495996 | 6.11E+00 | clementine0.9_024662m | AT1G20696.3 | HMGB3 | high mobility group B3 |
| 1. clementina_scaffold_56:868943-881195 | -0.493551 | 0.0003753 | clementine0.9_031655m | AT2G01440.1 |  | DEAD/DEAH box RNA helicase family protein |
| 1. clementina_scaffold_156:128518-134027 | -0.492945 | 5.95E-01 | clementine0.9_002887m | AT5G26742.2 | emb1138 | DEAD box RNA helicase (RH3) |
| 1. clementina_scaffold_20:3313169-3316575 | -0.489136 | 0.0006629 | clementine0.9_019837m | AT3G13224.1 |  | RNA-binding (RRM/RBD/RNP motifs) family protein |
| 1. clementina_scaffold_63:153085-156085 | -0.487748 | 0.0008211 | clementine0.9_012489m | AT4G31240.1 |  | protein kinase C-like zinc finger protein |
| 1. clementina_scaffold_87:657600-663508 | -0.482322 | 0.0002964 | clementine0.9_001317m | AT2G43160.1 |  | ENTH/VHS family protein |
| 1. clementina_scaffold_56:378887-382230 | -0.47343 | 0.0002374 | clementine0.9_014335m | AT3G17860.1 | JAZ3 | jasmonate-zim-domain protein 3 |
| 1. clementina_scaffold_10:3794205-3797130 | -0.47327 | 0.0005243 | clementine0.9_014838m | AT3G57030.1 |  | Calcium-dependent phosphotriesterase superfamily protein |
| 1. clementina_scaffold_47:795968-799553 | -0.47225 | 0.0011825 | clementine0.9_007841m | AT5G12250.1 | TUB6 | beta-6 tubulin |
| 1. clementina_scaffold_56:851266-855447 | -0.46531 | 0.0011784 | clementine0.9_033260m | AT2G01450.1 | MPK17 | MAP kinase 17 |
| 1. clementina_scaffold_20:3383817-3387759 | -0.455209 | 0.000133 | clementine0.9_023698m | AT3G01590.1 |  | Galactose mutarotase-like superfamily protein |
| 1. clementina_scaffold_41:1257148-1262105 | -0.447317 | 0.0005364 | clementine0.9_006770m | AT4G34240.1 | ALDH3I1 | aldehyde dehydrogenase 3I1 |
| 1. clementina_scaffold_1:2041282-2044591 | -0.443291 | 0.0011527 | clementine0.9_032289m | AT3G02100.1 |  | UDP-Glycosyltransferase superfamily protein |
| 1. clementina_scaffold_67:1285856-1292075 | -0.440485 | 0.0004006 | clementine0.9_002783m | AT2G35060.1 | KUP11 | K+ uptake permease 11 |
| 1. clementina_scaffold_3:4637114-4641780 | -0.440156 | 0.0001427 | clementine0.9_006765m | AT2G47600.1 | MHX | magnesium/proton exchanger |
| 1. clementina_scaffold_1:1396709-1405131 | -0.437364 | 0.0005982 | clementine0.9_032445m | AT4G19110.1 |  | Protein kinase superfamily protein |
| 1. clementina_scaffold_29:338115-343943 | -0.433393 | 0.0004961 | clementine0.9_009551m | AT3G18500.3 |  | DNAse I-like superfamily protein |
| 1. clementina_scaffold_18:1496287-1503835 | -0.432668 | 0.0004409 | clementine0.9_002305m | AT5G54090.1 |  | DNA mismatch repair protein MutS. type 2 |
| 1. clementina_scaffold_1:7432607-7437819 | -0.4311 | 9.45E+00 | clementine0.9_008722m | AT3G05165.1 |  | Major facilitator superfamily protein |
| 1. clementina_scaffold_24:723368-731000 | -0.430152 | 0.0011544 | clementine0.9_005739m | AT5G09880.1 |  | Splicing factor. CC1-like |
| 1. clementina_scaffold_96:136282-148974 | -0.426671 | 2.31E-01 | clementine0.9_004311m | AT3G59780.1 |  | Rhodanese/Cell cycle control phosphatase superfamily protein |
| 1. clementina_scaffold_12:2899124-2901805 | -0.413843 | 0.0006517 | clementine0.9_025092m | AT2G02760.1 | UBC2 | ubiquiting-conjugating enzyme 2 |
| 1. clementina_scaffold_49:1151871-1159707 | -0.407375 | 0.0006089 | clementine0.9_001985m | AT2G04270.2 | RNEE/G | RNAse E/G-like |
| 1. clementina_scaffold_41:607992-617557 | -0.403116 | 0.0001599 | clementine0.9_001601m | AT1G06840.1 |  | Leucine-rich repeat protein kinase family protein |
| 1. clementina_scaffold_3:5200082-5204185 | -0.366489 | 0.0006378 | clementine0.9_016985m | AT1G48910.1 | YUC10 | Flavin-containing monooxygenase family protein |
| 1. clementina_scaffold_136:148620-152018 | -0.263317 | 0.0008717 | clementine0.9_007219m | AT3G18890.1 |  | NAD(P)-binding Rossmann-fold superfamily protein |

*The fold change values (P ≤ 0.001) obtained from of each treated sample compared to HCl control.

** Identification number of *Citrus clementina* transcripts present in the locus - http://www.phytozome.org/search.php

***Identification number of the *Arabidopsis thaliana* ortholog of down-regulated citrus gene in response to CHI treatment (The Arabidopsis Genome Initiative).
